# Supplementary material for: Waffle Method: A general and flexible approach for improving throughput in FIB-milling
Source: Nat Commun. 2022 Apr 6;13:1857. doi: 10.1038/s41467-022-29501-3 (PMC8987090; doi:10.1038/s41467-022-29501-3)
Supplement: Supplementary file 1 — Supplementary Information [file 41467_2022_29501_MOESM1_ESM.pdf]

Supplementary Information

Supplementary Table 1: Required and recommended Waffle Method hardware

**Hardware requirements**	
**Equipment**	**Details**
Planchette hat polishing equipment	Sandpapers with multiple grits, fine-grain polishing solution, and Kimwipes.
1-hexadecene	Facilitates separation of grids from planchette hats.
5 µL of sample	Used for waffling.
Sputtering device	Used for sputtering carbon onto EM grids.
High-pressure freezer (HPF)	Used for vitrifying samples onto EM grids.
Cryo-transfer system	For transferring waffle grids between equipment. Preferably includes vacuum control between transfer steps.
High-vacuum sputtering device	For conductive platinum deposition at cryo-temperatures.
Tilted cryo-FIB/SEM grid holder	Allows for shallow lamella milling.
Cryo-FIB/SEM	Used to mill lamellae.
GIS system	Used for platinum GIS coating.
Common cryo-EM equipment	Carbon/gold EM grids, glow discharge cleaner or plasma cleaner, HPF planchette hats, tweezers, liquid nitrogen and dewars, cryo-gloves, eye protection (goggles/face mask), pipettes, and ethanol for cleaning
**Hardware recommendations**	
Humidity-controlled environment	Preferably set to ~10% RH to minimize contamination.
2-methylpentane	May help achieve full vitrification.

EM grid spacer ring	May be used to adjust waffle thickness for thicker specimens.
Magnifying glass and/or camera	May help with loading grids in proper orientations.
Flat cryo-FIB/SEM grid holder	May allow for easier trench milling, if necessary.
Cryo-FLM system	May help with screening waffles before/during milling and analyzing waffled lamellae after milling and/or after cryo-ET collection.
Fluorescently-tagged regions of interest in the specimen	May allow for FLM identification and localization of objects of interest.

Supplementary Table 1 | Lists of required and recommended hardware for the Waffle Method.

Supplementary Figure 1: Planchette hat sanding

a) New planchette hat (500x, 2kx mag)

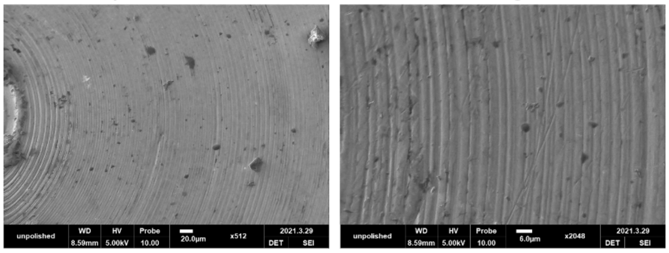

b) After 1,200 grit sandpaper (500x, 2kx mag)

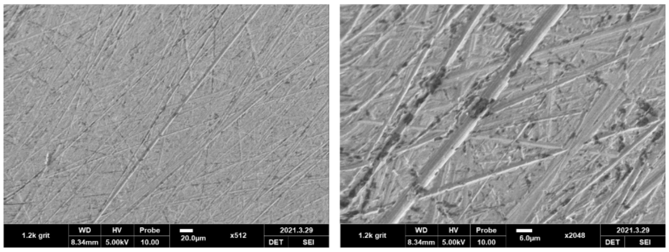

c) After 15,000 grit sandpaper (500x, 2kx mag)

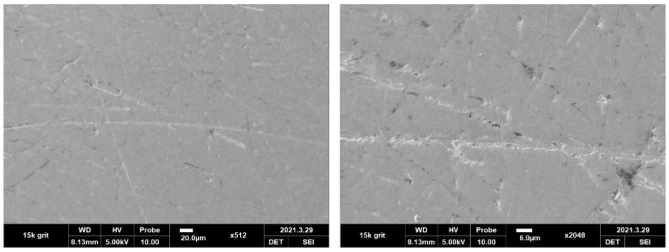

d) After 50,000 grit sandpaper (500x, 2kx mag)

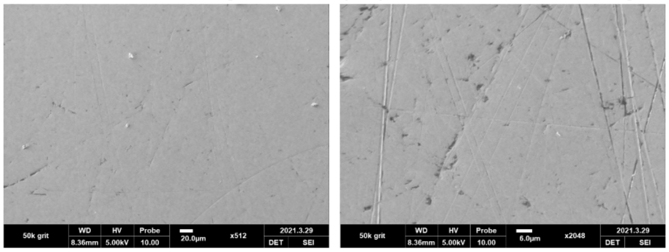

e)

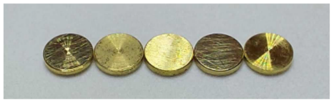

New planchette hats

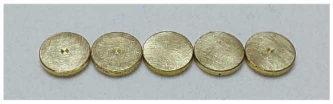

After 1,200 grit sandpaper

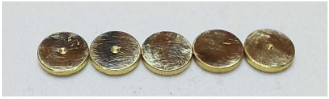

After 15,000 grit sandpaper

f)

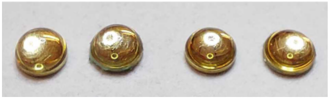

Incubate 15 min with 1-hexadecane

Supplementary Figure 1 | Images of a planchette hats at different sandpaper steps to illustrate surface smoothness. **(a)** SEM image of a section of a fresh planchette hat. 500x magnification image on the left and 2,000x magnification image of the center of the 500x image on the right. **(b)** After sanding with 1,200 grit sandpaper. **(c)** After additional sanding with 15,000 grit sandpaper. **(d)** After additional sanding with 50,000 grit sandpaper. **(e)** Low-magnification images of a series of planchette hats before sanding (top), after 1,200 grit sandpaper (middle), and after 15,000 grit sandpaper (bottom). **(f)** After sanding and metal polishing, the planchette hats are placed grid-facing side up on filter paper wetted with 1-hexadecane and with a drop of 1-hexadecane on the grid-facing side of each hat and incubated for 15 minutes before immediately assembling waffles for high-pressure freezing. Notice that the smoothness difference between (c) and (d) is negligible. SEM images (a-d) were collected on a Cube II Mini-SEM (EMCRAFTS, Korea). n = 1 experiment for each panel in (a-d).

Supplementary Figure 2: Waffle assembly

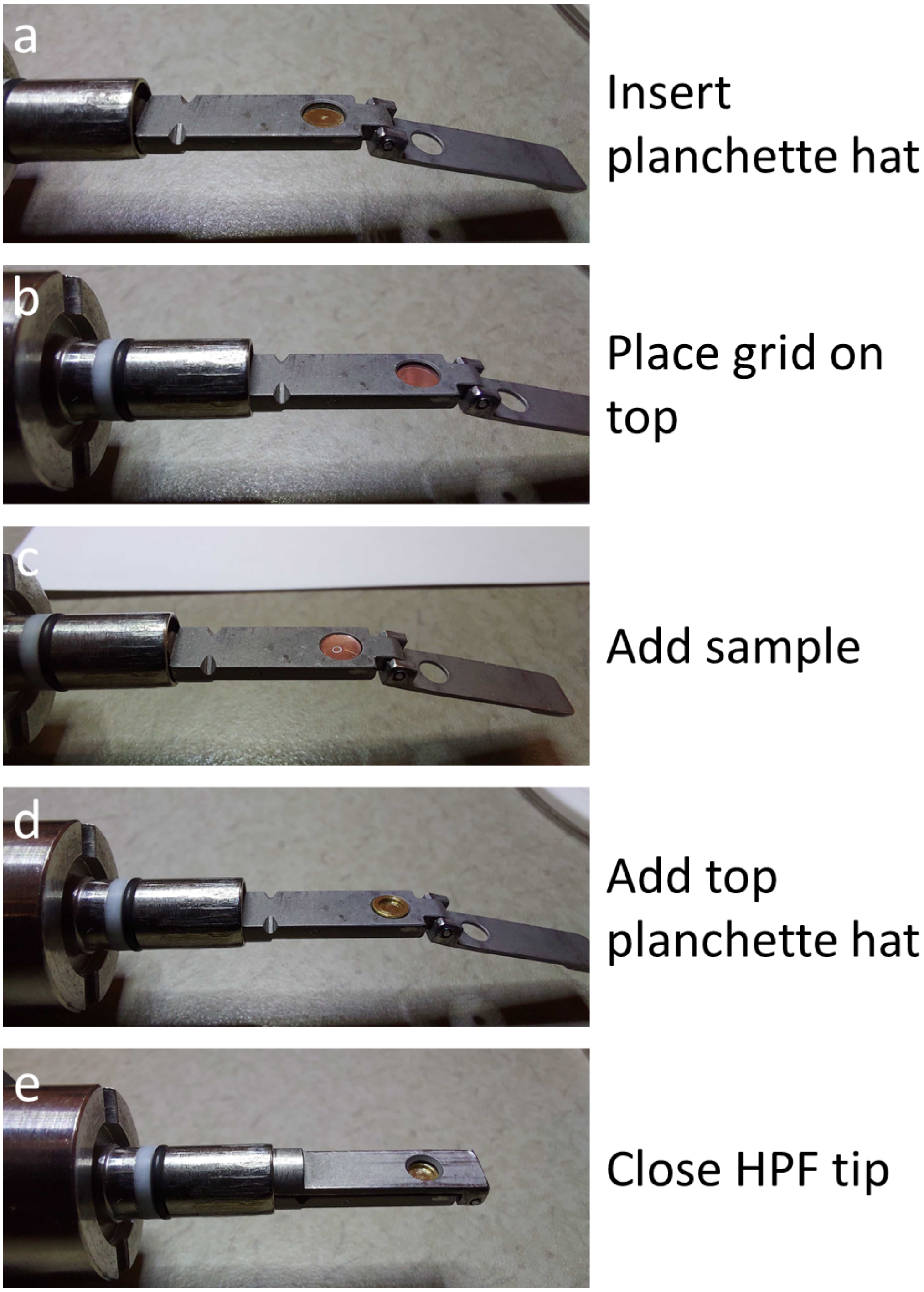

Supplementary Figure 2 | Images of the waffle assembly. Here, a waffle is assembled inside of a Wohlwend high-pressure freezer (HPF) tip. First a polished planchette hat with a fresh coat of 1-hexadecane is placed in the freshly-cleaned HPF tip **(a)**, then a freshly glow-discharged EM grid with 25 nm of extra carbon is placed on top with the grid bar side facing up **(b)**, several µL of sample is added on top of the grid **(c)**, the top polished planchette hat is added to the top **(d)**, and the HPF tip is closed before freezing **(e)**. Excess sample may need to be removed prior to freezing.

Supplementary Figure 3: Waffle milling details

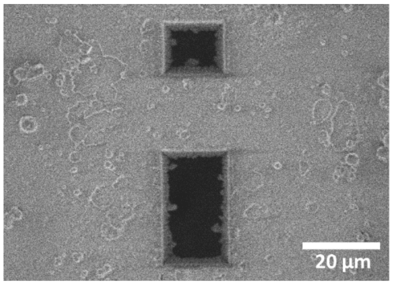

Trench milling
- ~90° to the grid
- Near the square center
- Milling current: 9 – 15 nA
- ~30 µm dimensions,
  20 – 30 µm separation

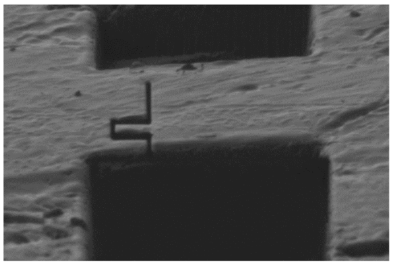

Notch preparation
- ≤20° to the grid
- Notch current: ~0.3 nA
- Notch width: 200 – 300 nm

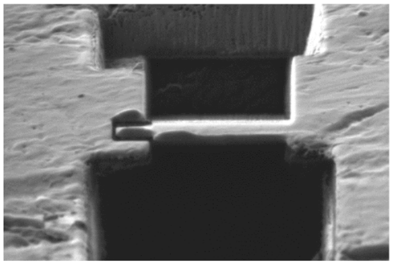

Coarse milling
- ≤20° to the grid
- Milling current: ≤1 nA
- Milled to 1 – 3 µm thick

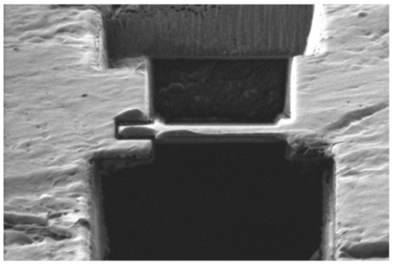

Medium milling
- Same angle as coarse milling
- Milling current: ≤0.5 nA
- Milled to 0.8 – 2 µm thick

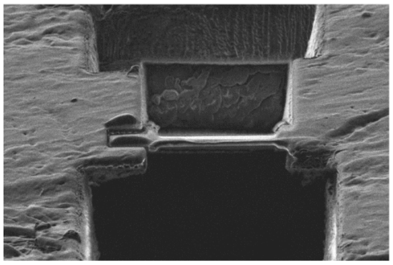

Fine milling
- Same angle as coarse milling
- Milling current: ≤0.3 nA
- Milled to 0.4 – 1 µm thick
- Possibly with 0.5 – 1° overtilt

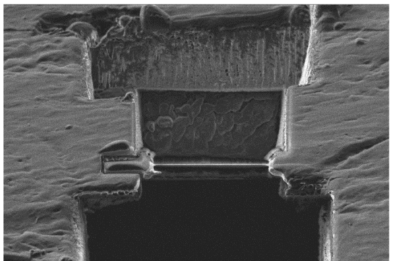

Lamellae polishing
- Same angle as coarse milling
- Milling current: ≤0.05 nA
- Milled to 0 – 0.2 µm thick
  (defined by milling software;
  not actual thickness)

Supplementary Figure 3 | FIB images of waffle milling with details of the current protocol from trench milling to polishing. Trench milling is performed manually and lamella milling is performed with automation software. Typically we mill the trench closer to the FIB beam to be about twice as long as the other trench to allow for easier navigation while milling at shallow angles. Note: Milling thicknesses are defined by the milling software and may not represent the actual thicknesses. n > 100 independent experiments with similar results.

Supplementary Figure 4: Waffle milling workflow

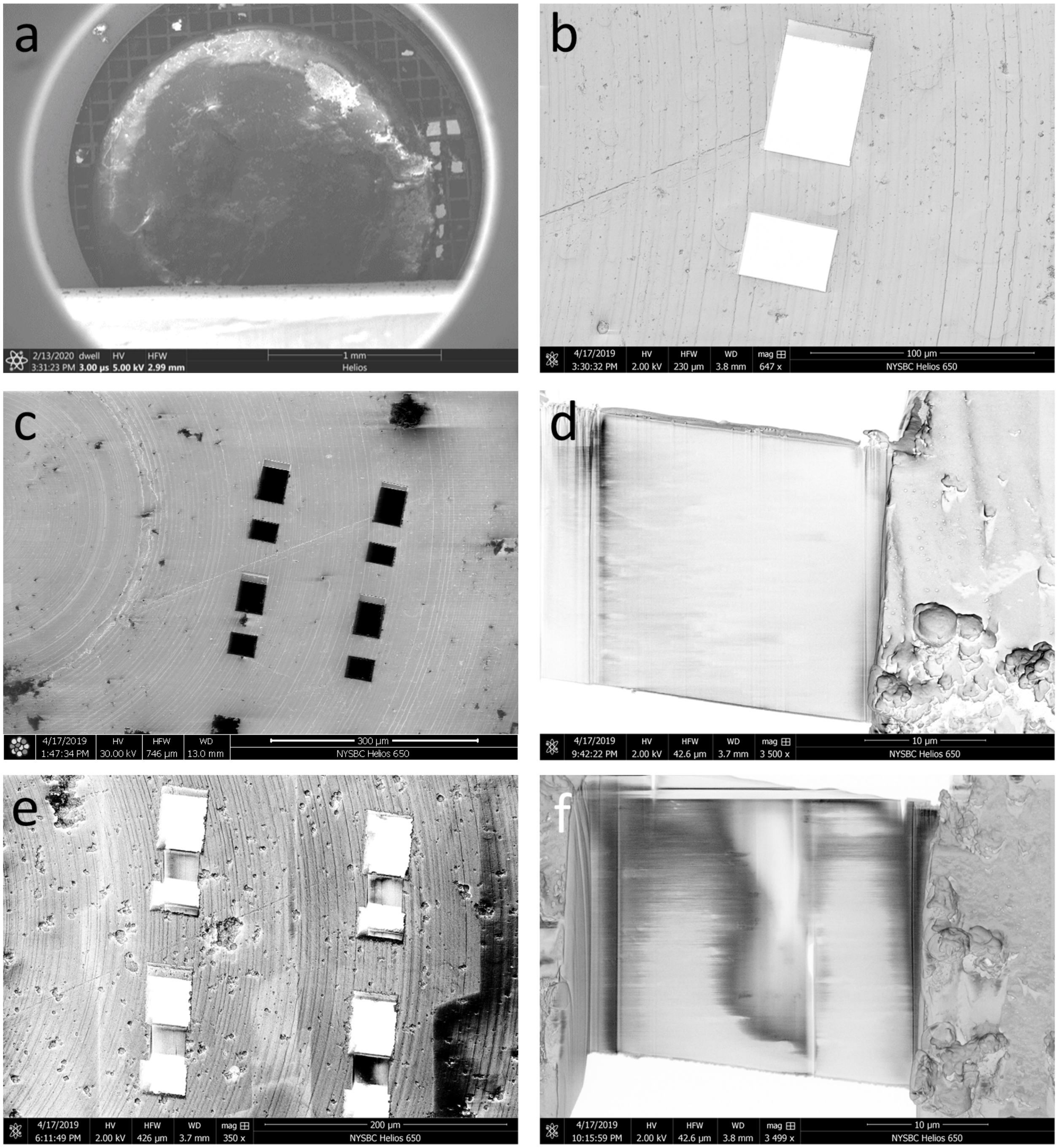

Supplementary Figure 4 | FIB/SEM images of the waffle milling workflow. **(a)** After creating a waffled sample, **(b,c)** two trenches per area of interest are milled as perpendicular to the plane of the grid as possible and tens of microns apart. **(d,e)** The grid is then tilted to the desired angle and milled first coarsely then finely while keeping the waffle platinum layer on the leading edge of milling. **(f)** The resulting lamellae are then polished. Notches may be milled for stress-relief between (c) and (d), as shown in Figure 2. n > 100 independent experiments with similar results.

Supplementary Figure 5: Importance of sufficiently smooth waffles and a sufficient amount of platinum GIS pre-coating

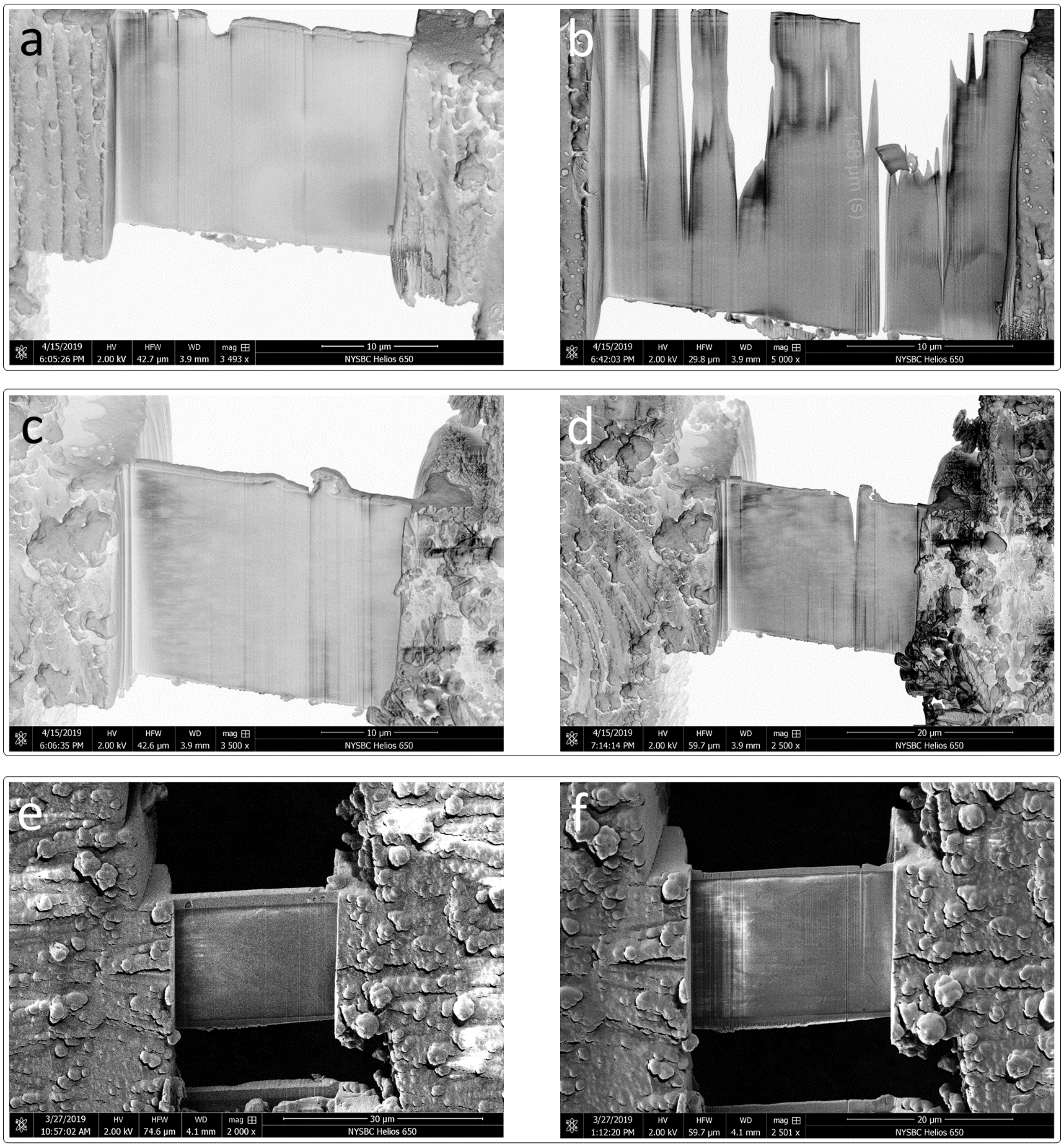

Supplementary Figure 5 | SEM images from three separate milling areas showing the importance of sufficiently smooth waffles and applying a sufficient amount of platinum GIS pre-coating. **(a)** shows a lamella milled to several hundred nanometers thickness with an insufficient amount of insufficiently smooth platinum GIS pre-coating causing further milling to curtain and deteriorate the lamella **(b)**. **(c)** shows a lamella milled to several hundred nanometers thickness with sufficiently smooth platinum GIS on the left side of the lamella, but not on the right. Finer milling results in reduced curtaining on the left side compared to the right **(d)**. **(e)** shows a lamella milled to several hundred nanometers thick with a sufficient amount of sufficiently smooth platinum GIS. Finer milling results in minimal curtaining **(f)**. n = 1 independent experiment for each panel.

Supplementary Figure 6: Very wide yeast waffle lamella

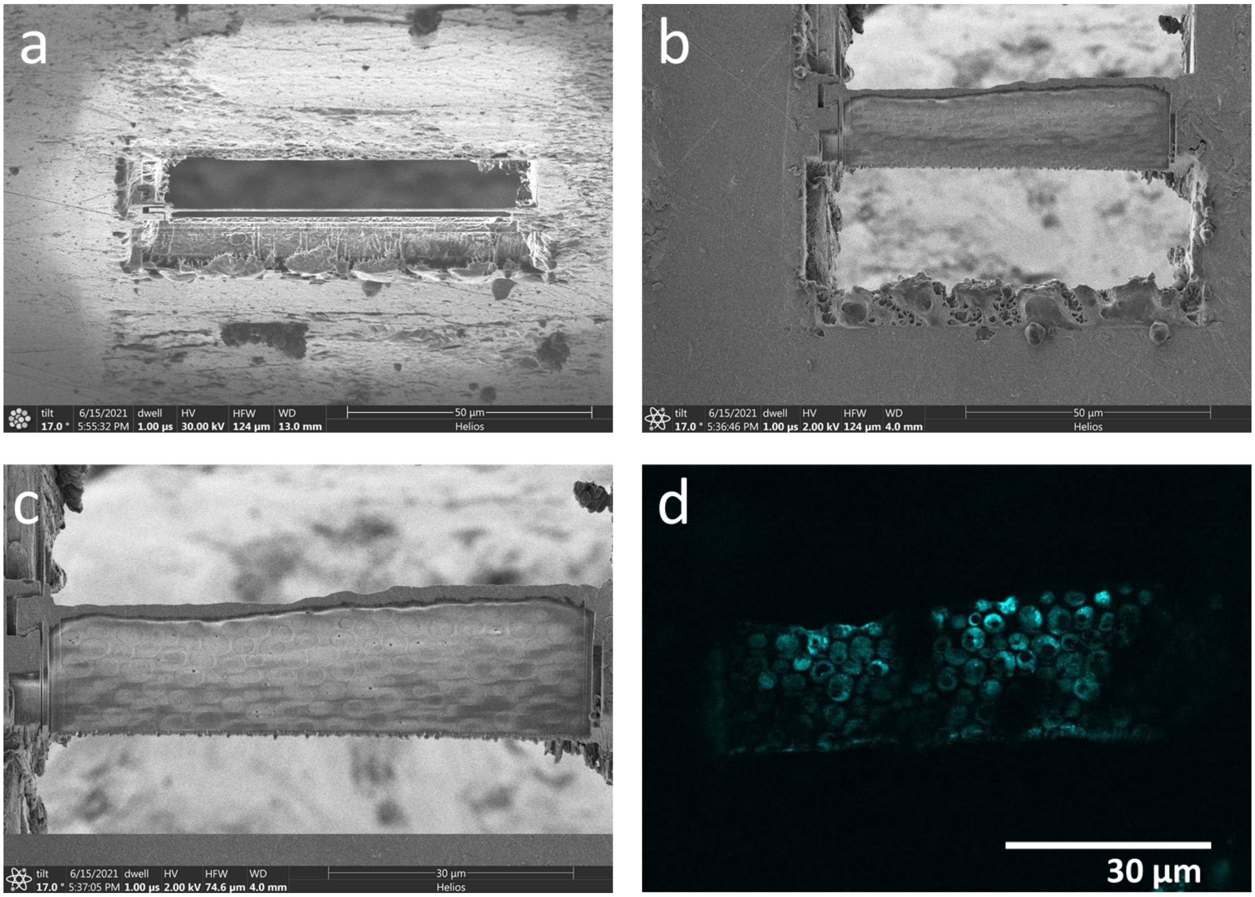

Supplementary Figure 6 | FIB/SEM images of waffle milling of very wide waffle lamella of yeast *S. cerevisiae* with a notch milled into the left-hand side. This lamella is about 70 x 25 µm. **(a)** FIB image of the finished waffle lamella. **(b)** SEM image of finished lamella. **(c)** Zoomed-in SEM image of the lamella in (b) showing dozens of imageable yeast cell cross-sections. **(d)** Zeiss Airyscan 2 cryo-FLM image (Zeiss, Oberkochen, Germany) of the lamella after cryo-ET collection showing GFP-tags. n = 1 experiments (ie. only one lamella with this width was milled).

Supplementary Figure 7: Gap milling development for waffled lamellae

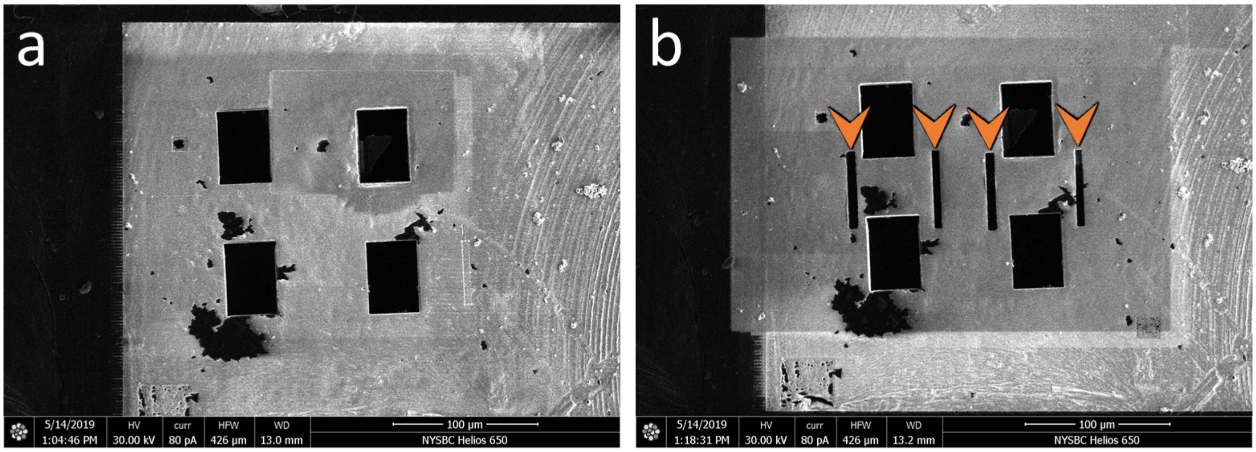

Supplementary Figure 7 | Initial stress-relief gap milling development for waffled lamellae. **(a)** Two trench-milled slabs. **(b)** Gaps (orange arrows) about 6 µm in width on either side of the slabs intended for lamellae stress-relief. Milling gaps in the sides of the lamellae after coarse milling was also explored (not pictured here). Neither method proved to relieve stress enough to avoid damage to waffled lamellae. n > 5 independent experiments with gap milling and waffle lamellae were performed.

Supplementary Figure 8: Possible mechanism for notch milling success

a) After milling

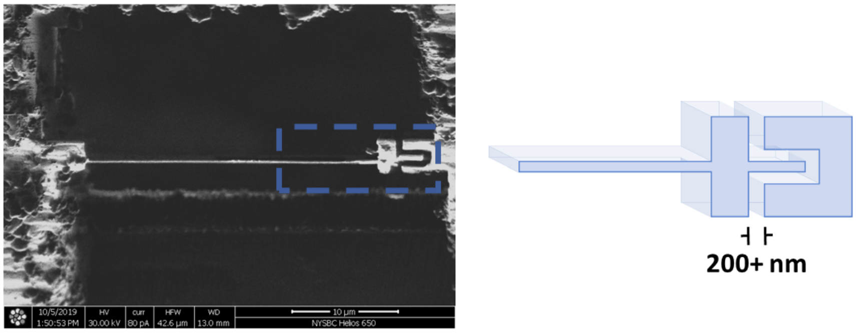

b) During applied forces on grid

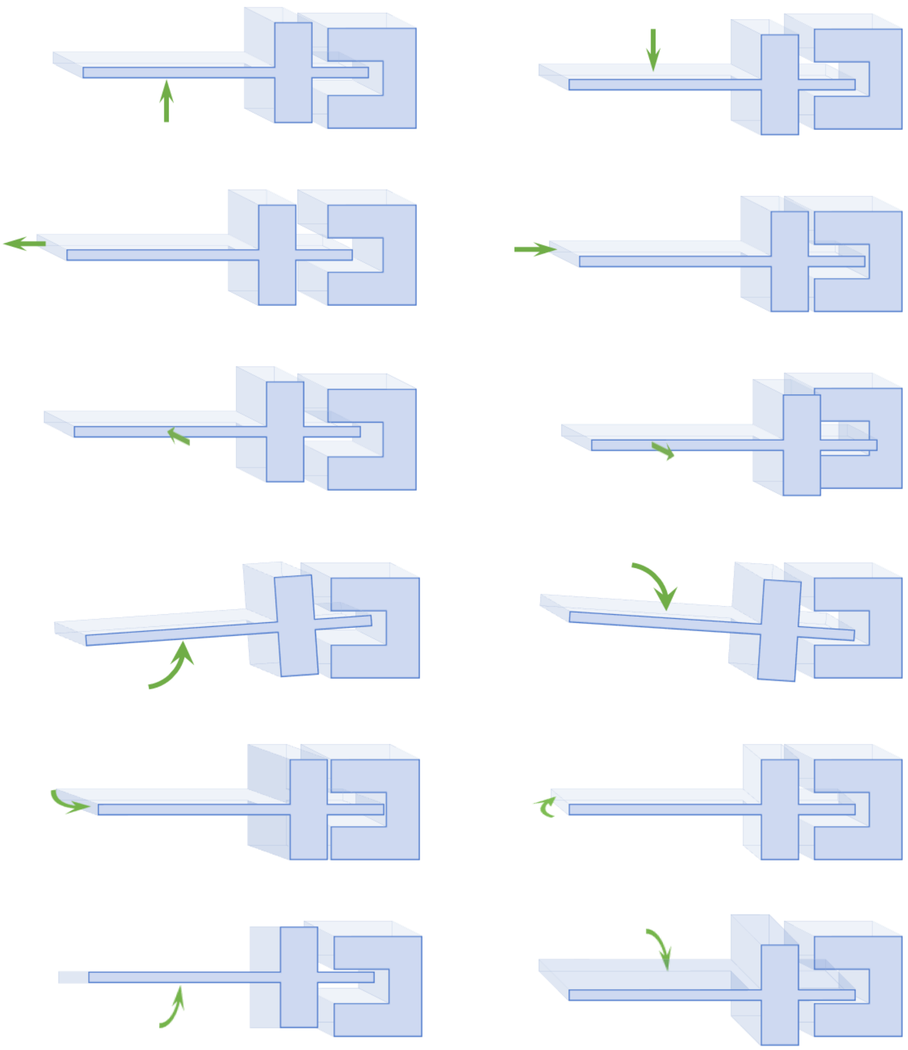

Supplementary Figure 8 | Illustrations of why notch milling waffled lamellae may allow for lamellae to withstand directional and angular forces applied to the grid. **(a)** Immediately after notch milling, the lamellae tab is separated from the notch. **(b)** Directional and angular forces applied during grid handling and from thermal fluctuations are dissipated by the notches instead of affecting the lamellae.

Supplementary Figure 9: Notch resting

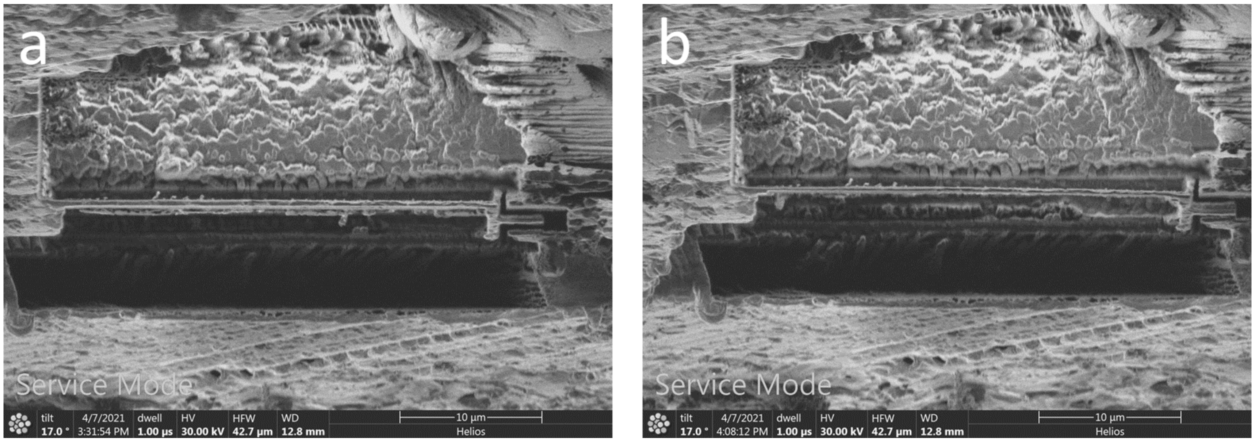

Supplementary Figure 9 | FIB images a milled notch coming to rest. **(a)** Image of a waffle lamella part way through milling and after the notch has already been milled. **(b)** Image of the same lamella after about 30 minutes had passed. No milling operations were performed on the lamella between (a) and (b). 1 < n < 5 independent experiments have each seen a notch come to rest.

Supplementary Figure 10: Arrowhead notch

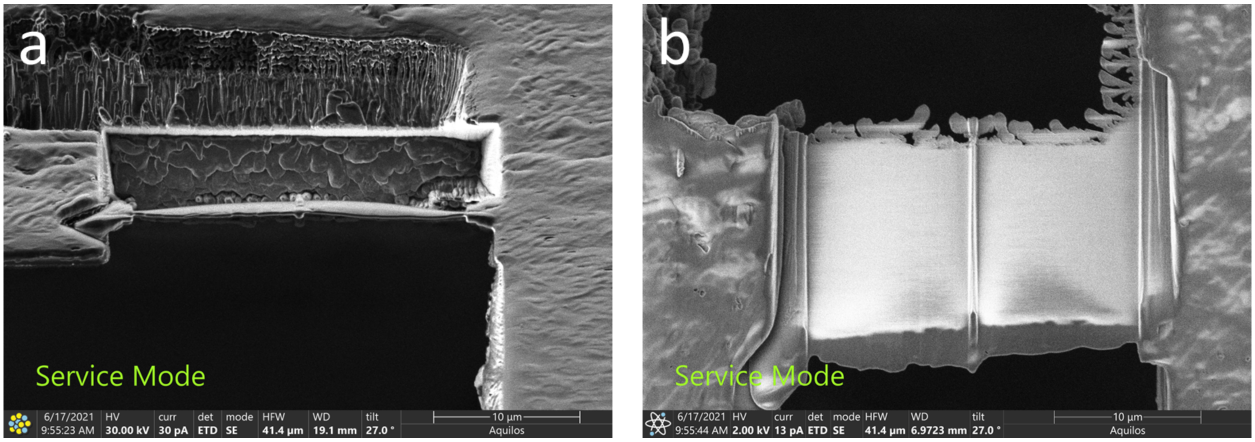

Supplementary Figure 10 | FIB image **(a)** and SEM image **(b)** of an alternative arrowhead notch (on the left side of the lamella). n = 1 experiment.
