# Supplementary material for: Waffle Method: A general and flexible approach for improving throughput in FIB-milling
Source: Nat Commun. 2022 Apr 6;13:1857. doi: 10.1038/s41467-022-29501-3 (PMC8987090; doi:10.1038/s41467-022-29501-3)
Supplement: Supplementary file 3 — Description of Additional Supplementary Files [file 41467_2022_29501_MOESM3_ESM.pdf]

**Description of Additional Supplementary Files**

File Name: Supplementary Movie 1
Description: **General Waffle Method workflow.** Animation showing the general Waffle Method workflow.

File Name: Supplementary Movie 2
Description: **Kotaro Kelley making a waffle grid.** Movie of Kotaro Kelley making a waffle grid. Here he performs most of Step 1 in Figure 1: (0:11) He places a planchette hat previously coated with 1-hexadecene into an HPF tip that was previously cleaned with ethanol, (0:27) several microliters of sample are applied to the planchette hat, (0:47) excess sample is wicked away after the top planchette hat is positioned, (1:09) the sample is high-pressure frozen, and (1:34) the waffled assembly is removed from the HPF tip in LN2.

File Name: Supplementary Movie 3
Description: **Microsporidian spore tomograms**. Tomogram slice-through movies of the microsporidian spores shown in Figure 3.

File Name: Supplementary Movie 4
Description: **Waffle milling time-lapse movie.** Time-lapse movie of coarse-to-fine waffle milling using AutoTEM on the Aquilos 2.

File Name: Supplementary Movie 5
Description: **Yeast tomograms.** Tomogram slice-through movies of the yeast S. cerevisiae cells. The left tomogram is 250 nm thick and the right tomogram is 500 nm thick. Partway through the movie, several ribosomes (orange), potential mitochondria (yellow), and the nuclear envelope (light blue) are highlighted.

File Name: Supplementary Movie 6
Description: **E. coli tomograms.** Tomogram slice-through movies of the E. coli BL21 (DE3) cells + Leviviridae PP7-PP7 virus capsid proteins. Partway through the movie, several virus capsids (red-orange), ribosomes (orange), and neighboring cell-cell membranes (green) are highlighted.

File Name: Supplementary Movie 7
Description: **HEK 293S cell tomogram.** Tomogram slice-through movie of the HEK cells. Partway through the movie, several transmembrane proteins (blue), ribosomes (orange), and actin networks (purple) are highlighted.

File Name: Supplementary Movie 8
Description: **Low-magnification E. coli tomogram.** Low magnification tomogram slice-through movies of the E. coli cells showing top-ends and sides of cell outer membranes.

File Name: Supplementary Movie 9
Description: **Possible mechanism for notch milling success.** Animation of the illustration in Supplementary Figure 8 of why notch milling waffled lamellae may allow for lamellae to withstand directional and angular forces applied to the grid.

File Name: Supplementary Movie 10
Description: **Single particle ThG waffle tomograms.** Tomogram slice-through movies of the ThG single particle lamella. Several orientational views of the protein are highlighted in red partway through the movie.
