# Supplementary material for: Waffle Method: A general and flexible approach for improving throughput in FIB-milling
Source: Nat Commun. 2022 Apr 6;13:1857. doi: 10.1038/s41467-022-29501-3 (PMC8987090; doi:10.1038/s41467-022-29501-3)
Supplement: Supplementary file 4 — Supplementary Movie 1 [file 41467_2022_29501_MOESM4_ESM.pptx]

## Slide 1
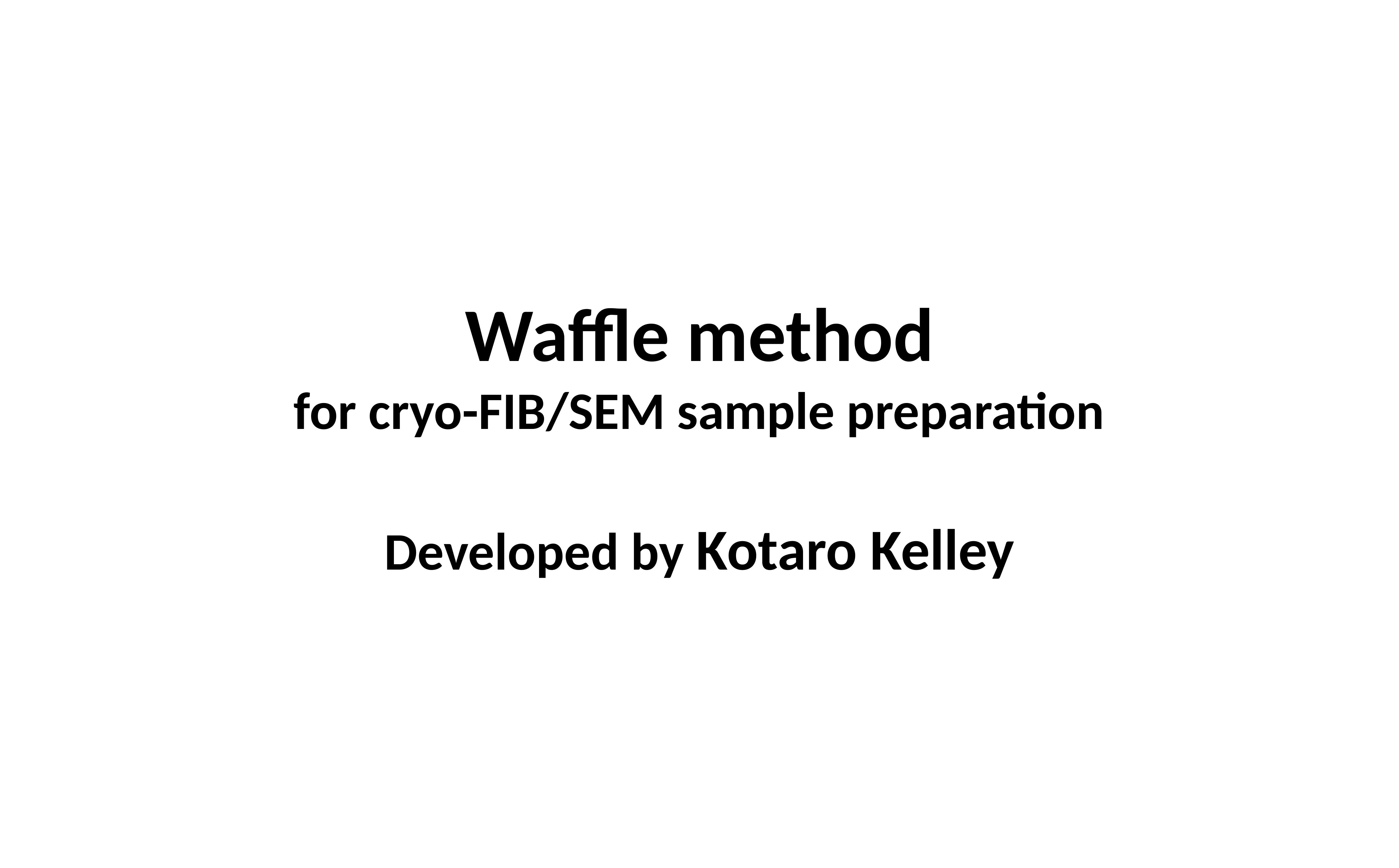

Waffle method
for cryo-FIB/SEM sample preparation
Developed by Kotaro Kelley

## Slide 2
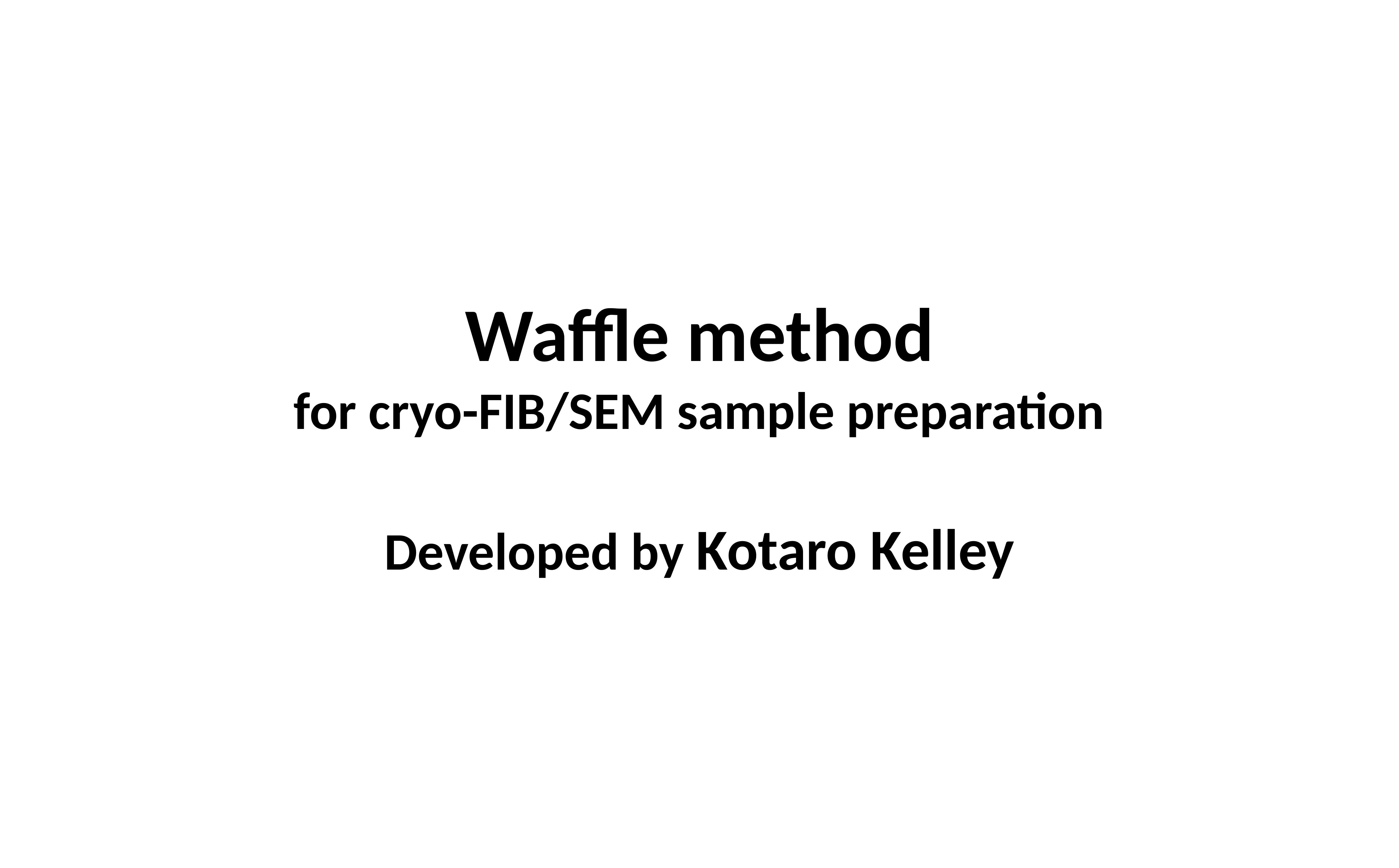

Waffle method
for cryo-FIB/SEM sample preparation
Developed by Kotaro Kelley

## Slide 3
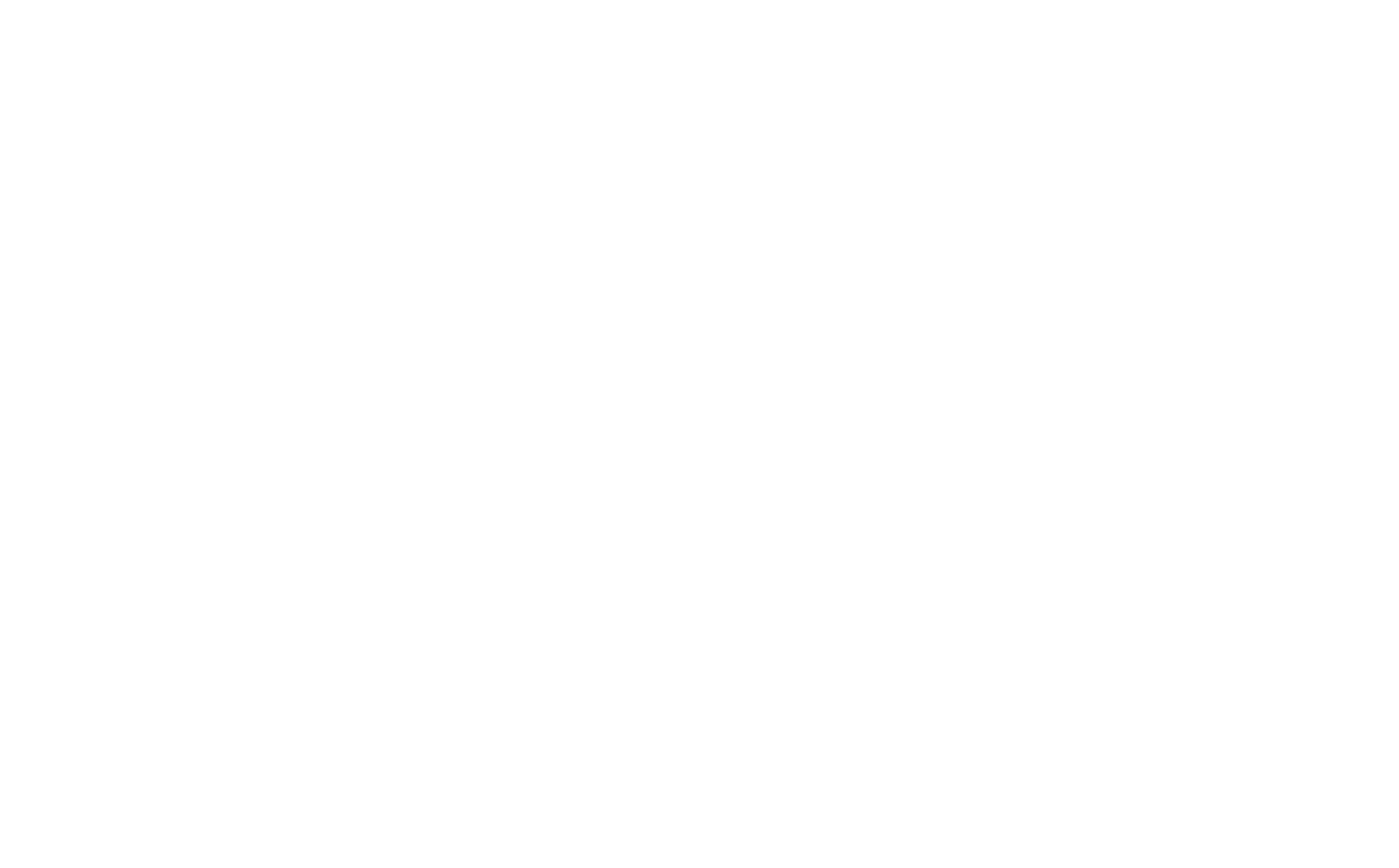

## Slide 4
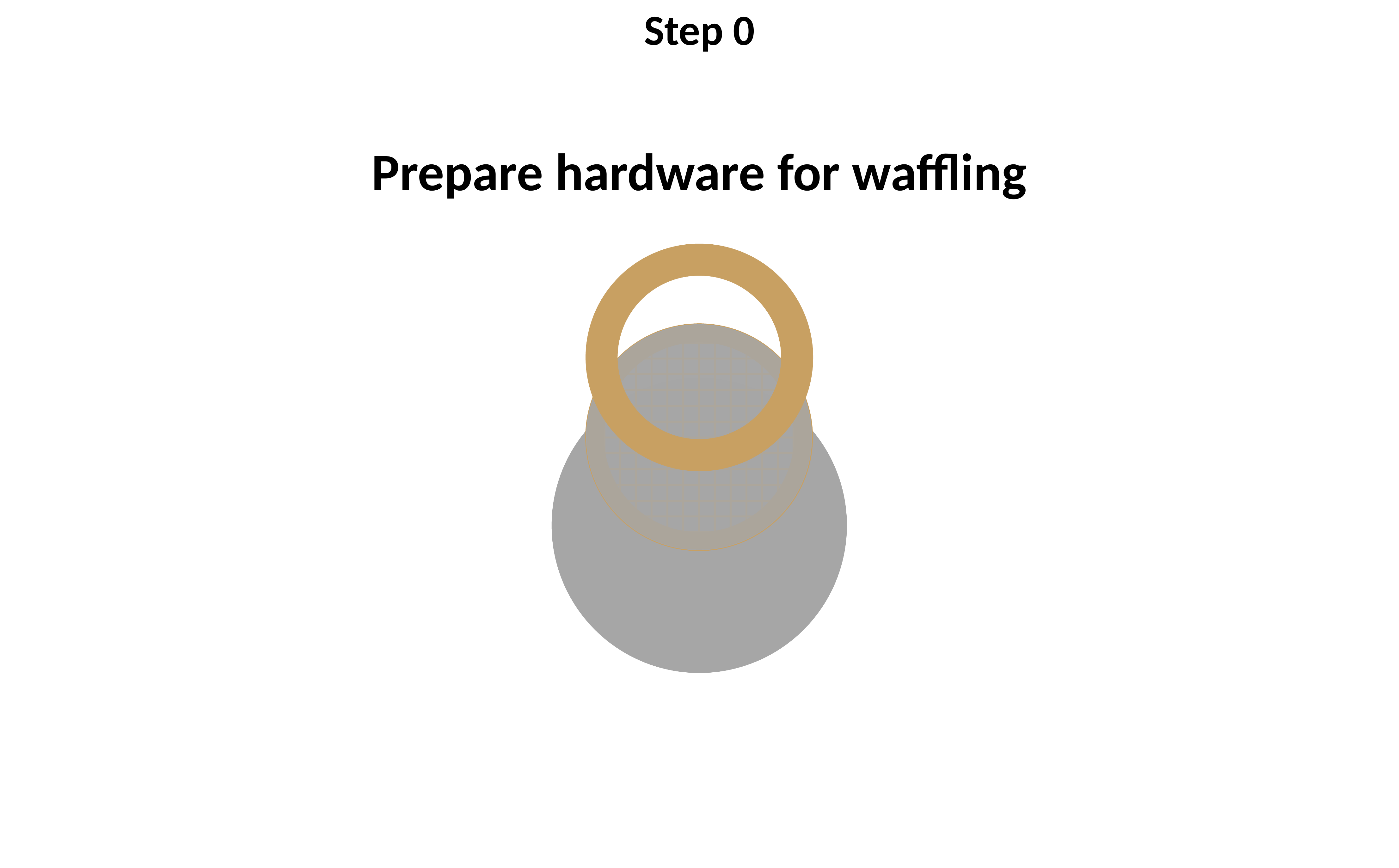

Step 0
Prepare hardware for waffling

## Slide 5
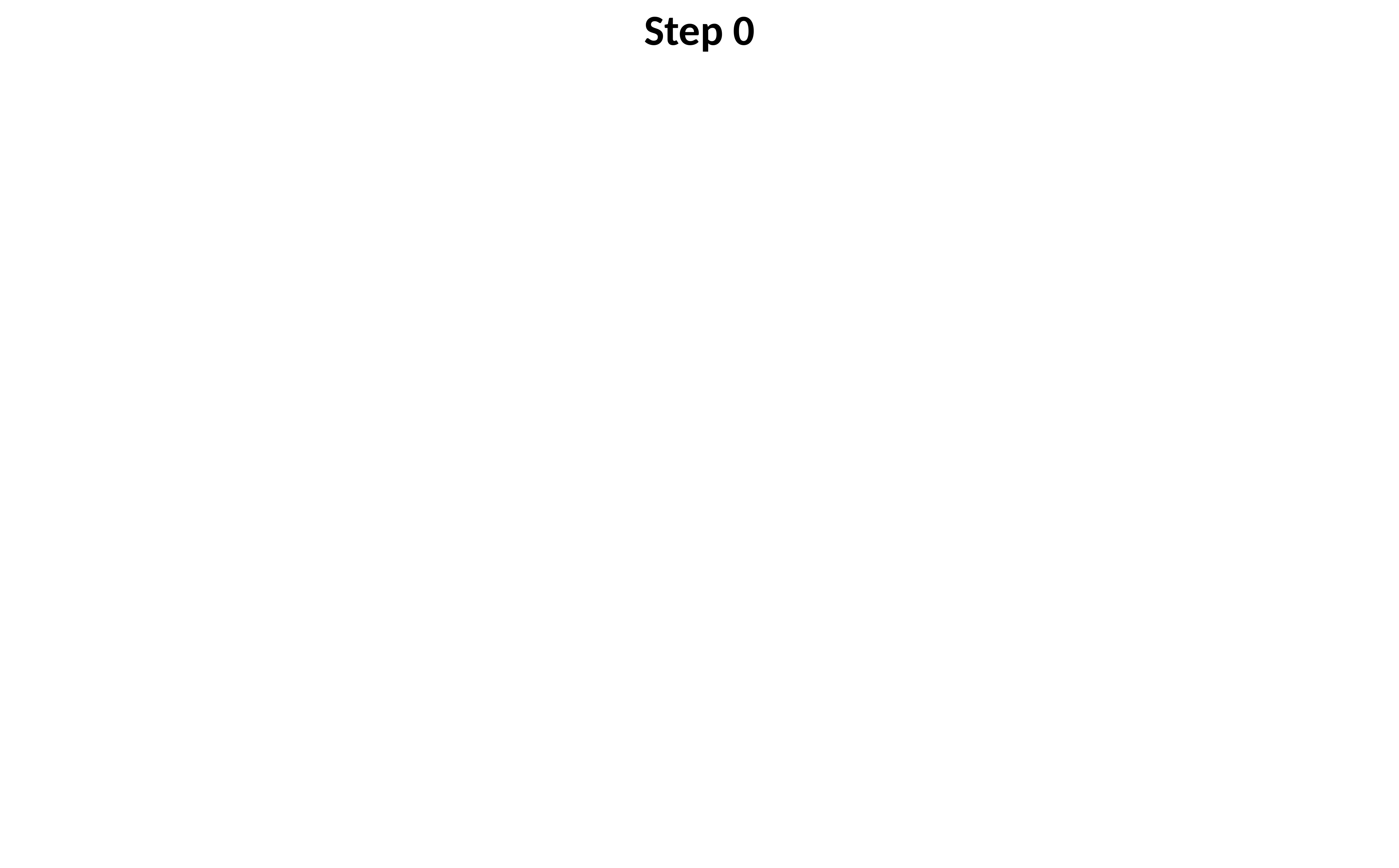

Step 0

## Slide 6
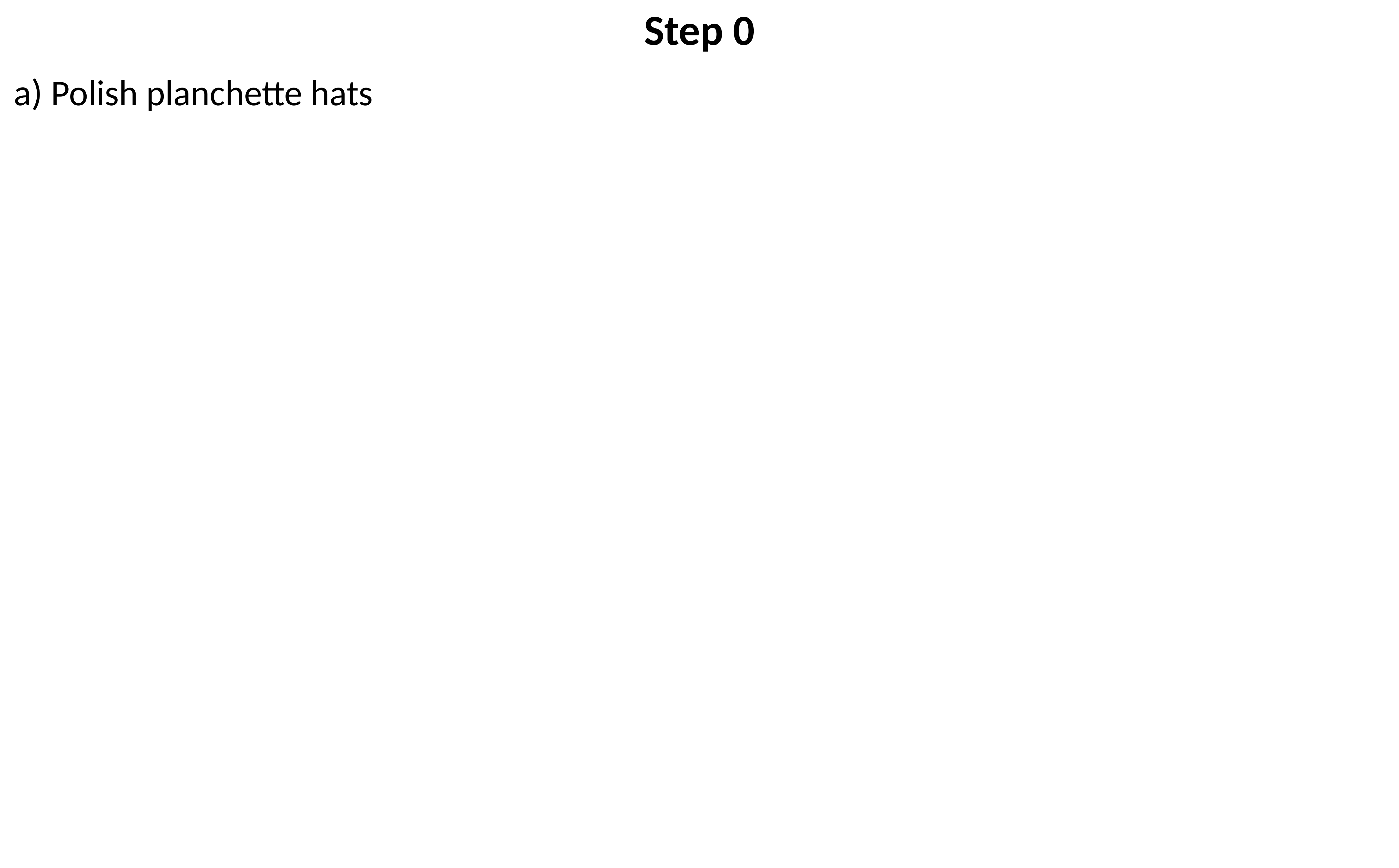

Step 0
a) Polish planchette hats

## Slide 7
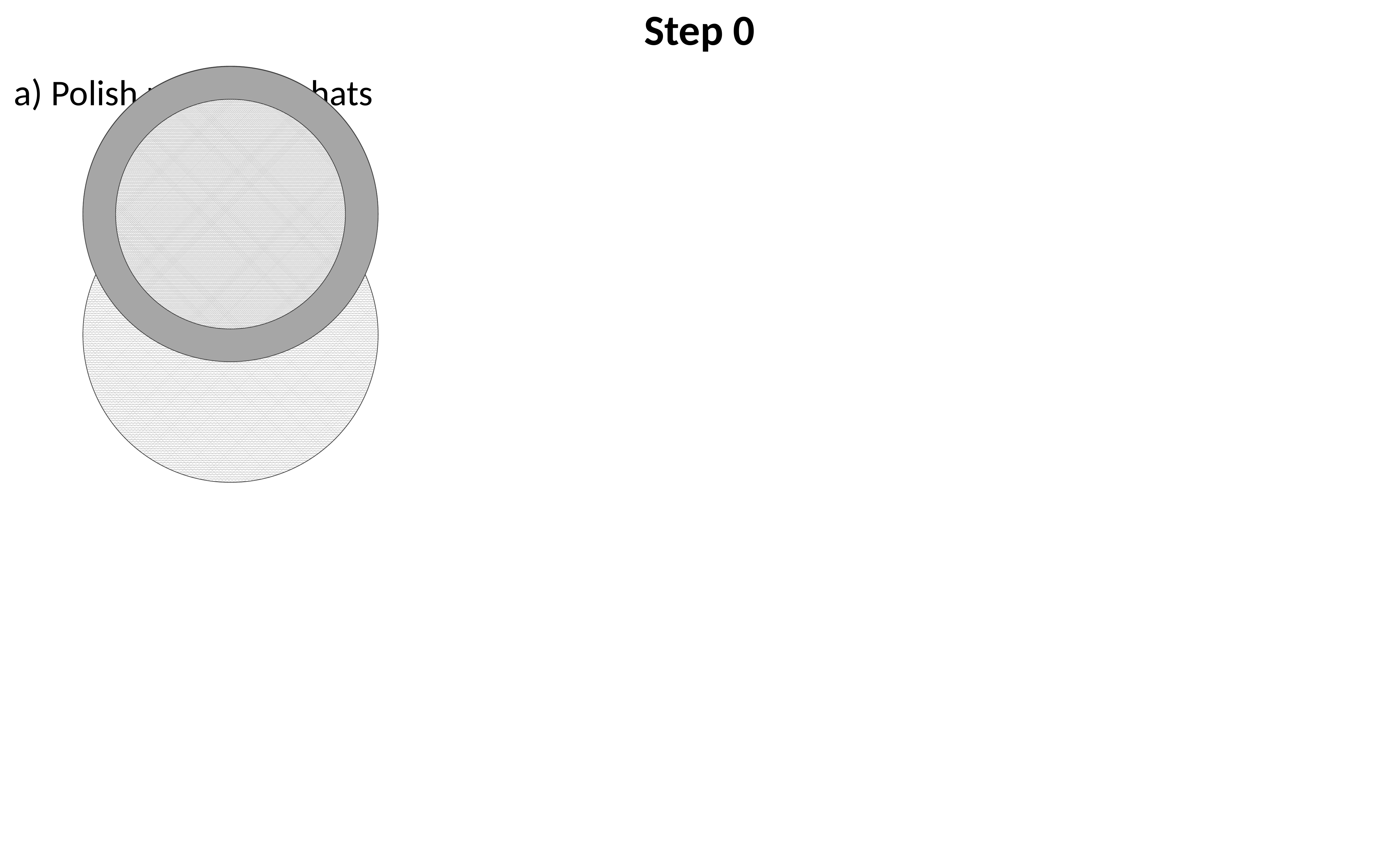

Step 0
a) Polish planchette hats

## Slide 8
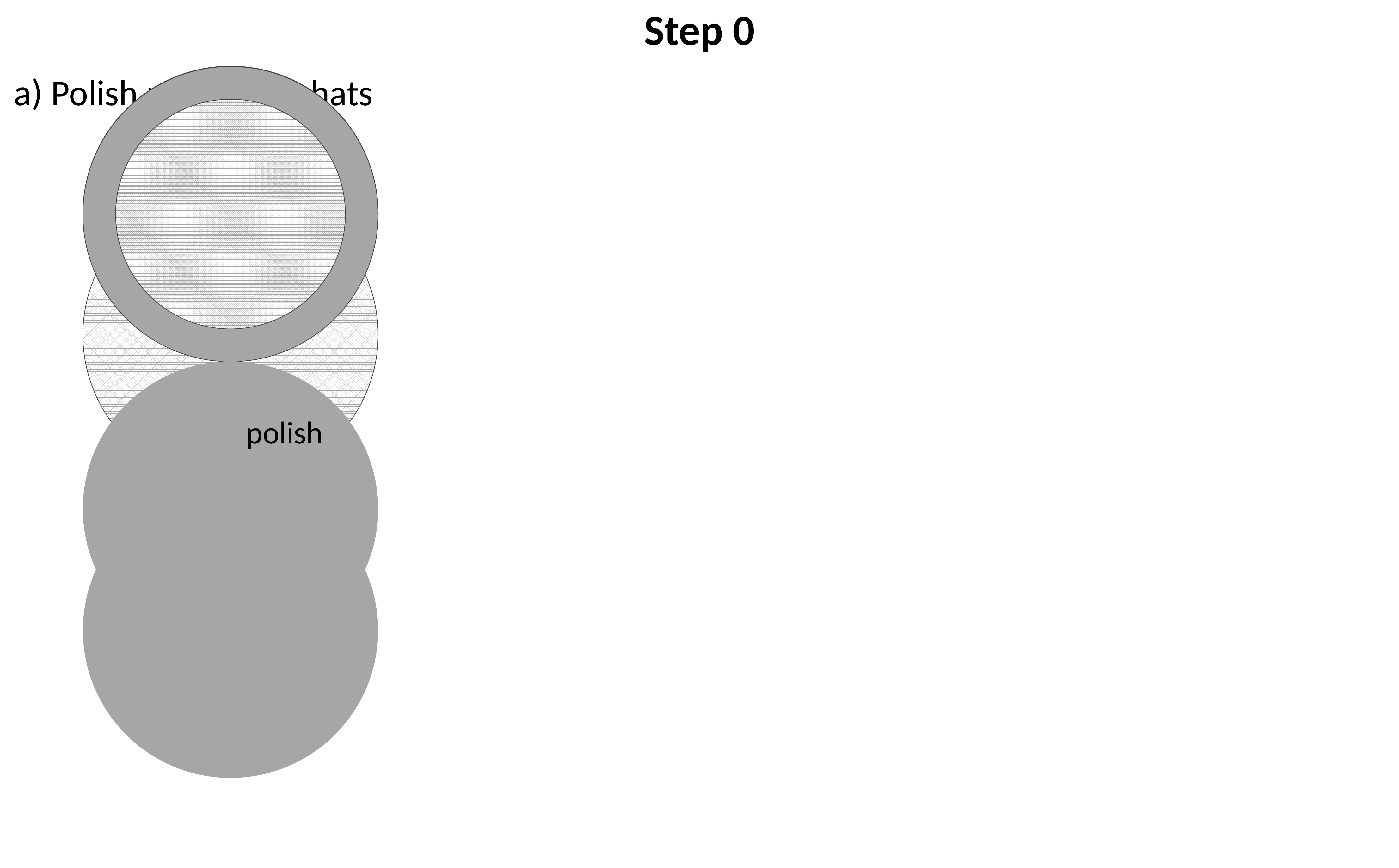

Step 0
a) Polish planchette hats
polish

## Slide 9
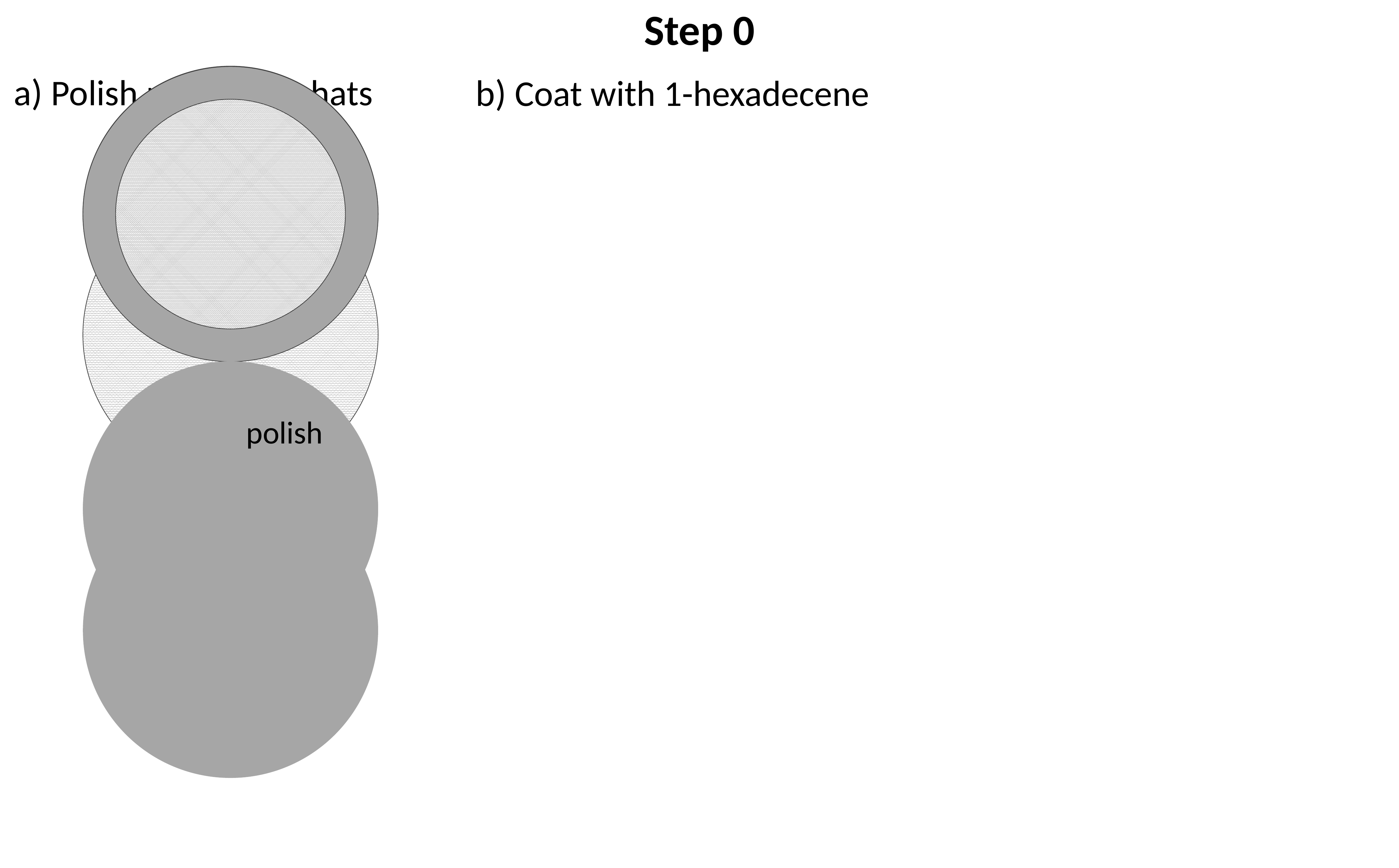

Step 0
a) Polish planchette hats
b) Coat with 1-hexadecene
polish

## Slide 10
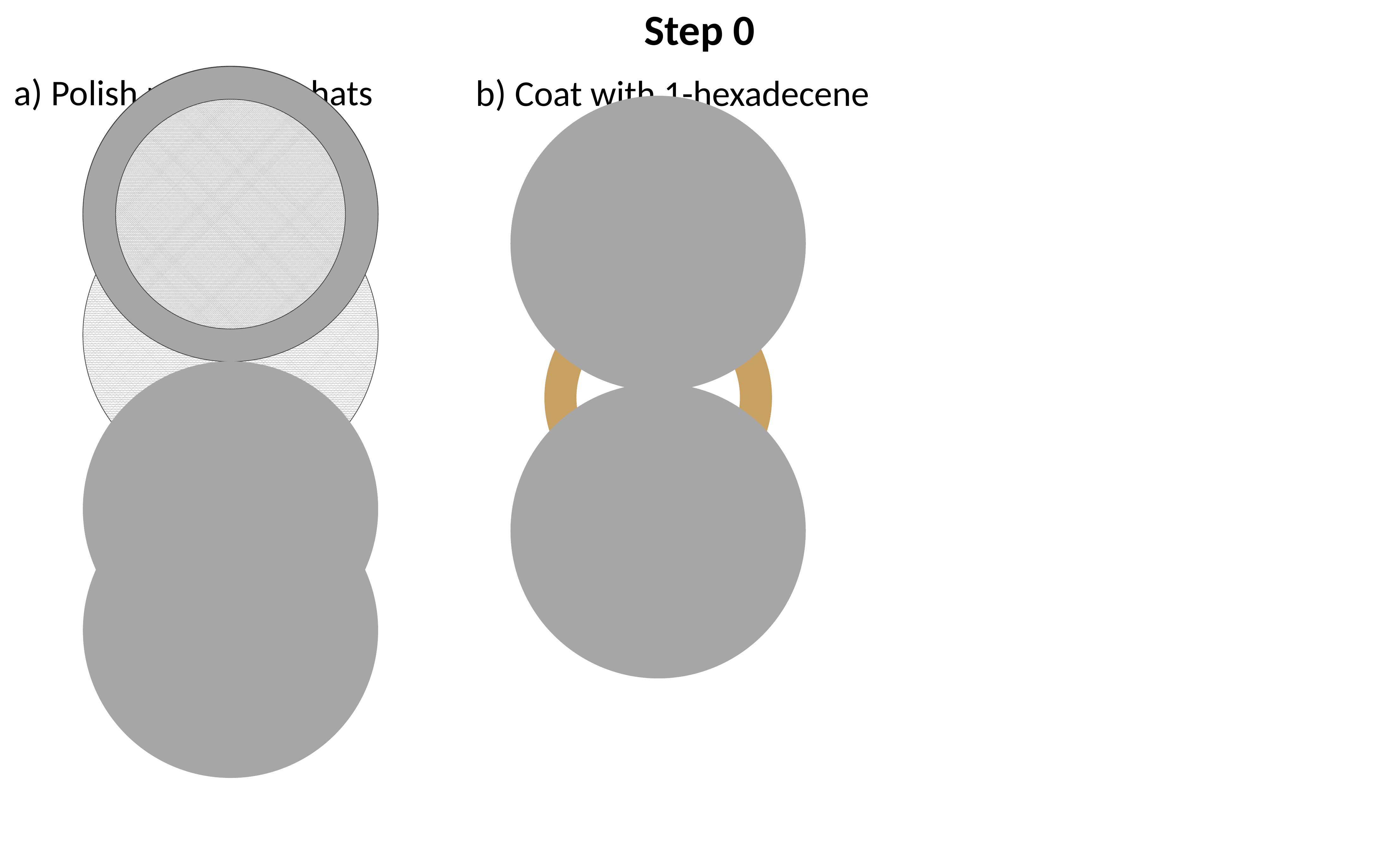

Step 0
a) Polish planchette hats
b) Coat with 1-hexadecene

## Slide 11
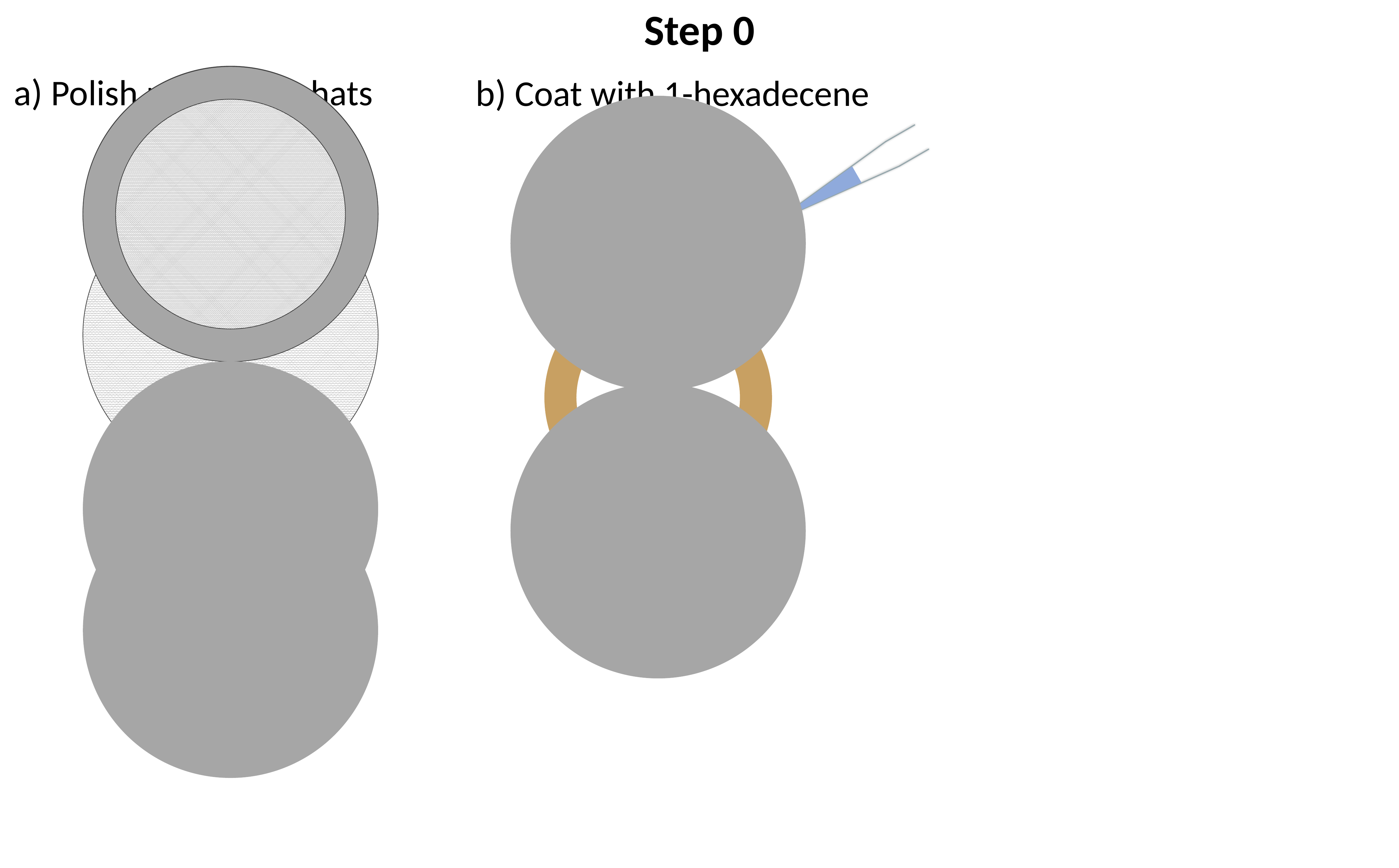

Step 0
a) Polish planchette hats
b) Coat with 1-hexadecene

## Slide 12
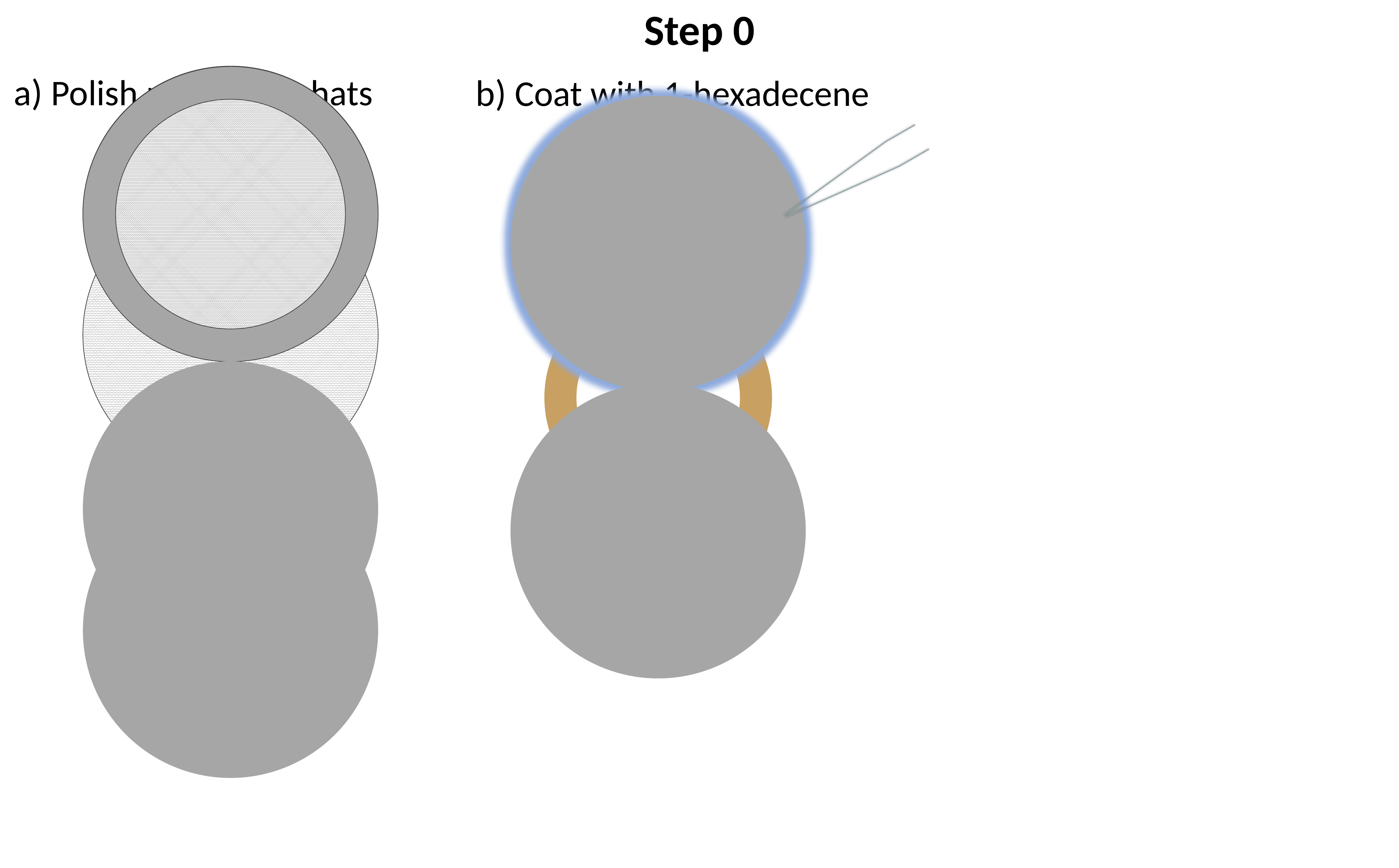

Step 0
a) Polish planchette hats
b) Coat with 1-hexadecene

## Slide 13
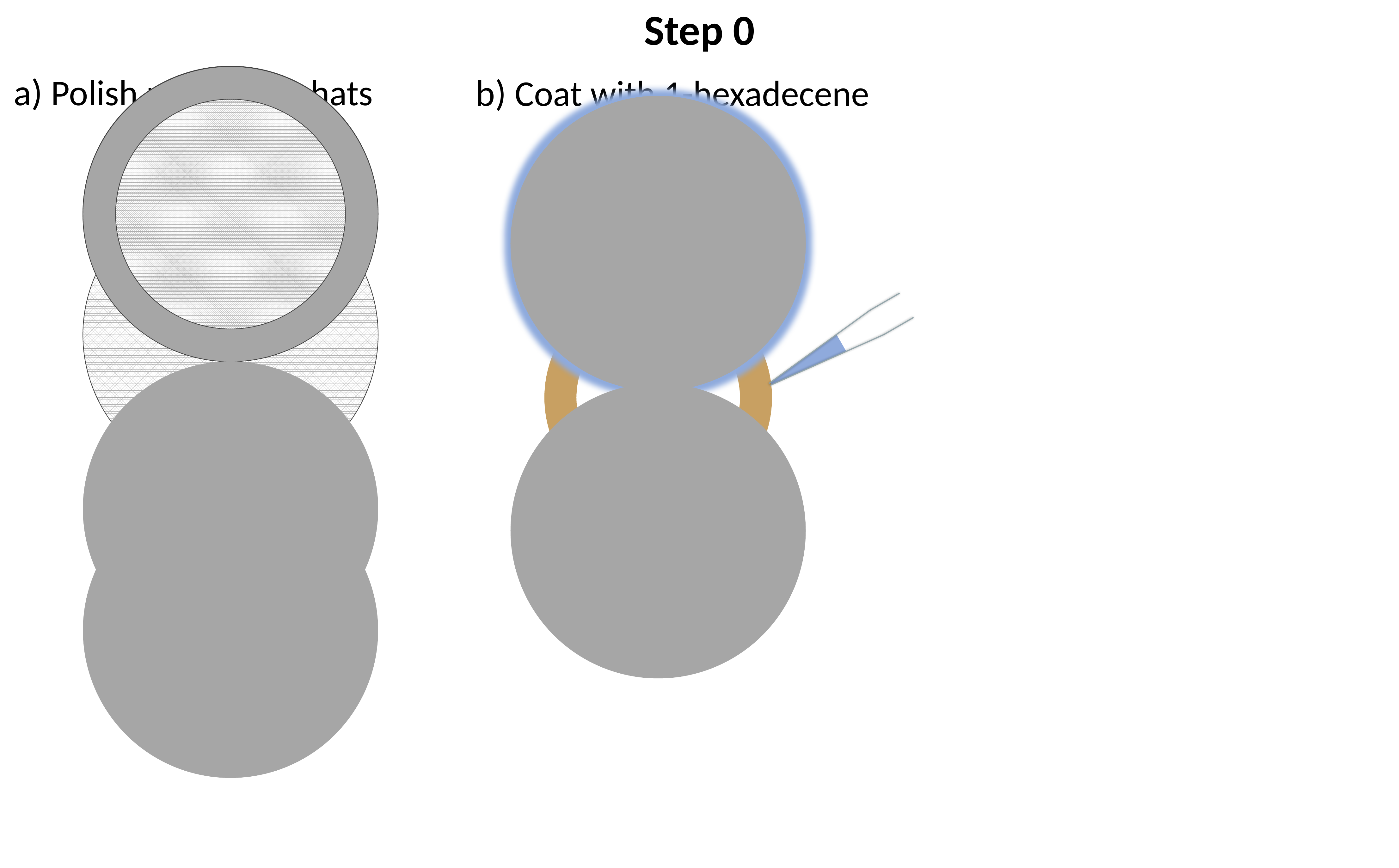

Step 0
a) Polish planchette hats
b) Coat with 1-hexadecene

## Slide 14
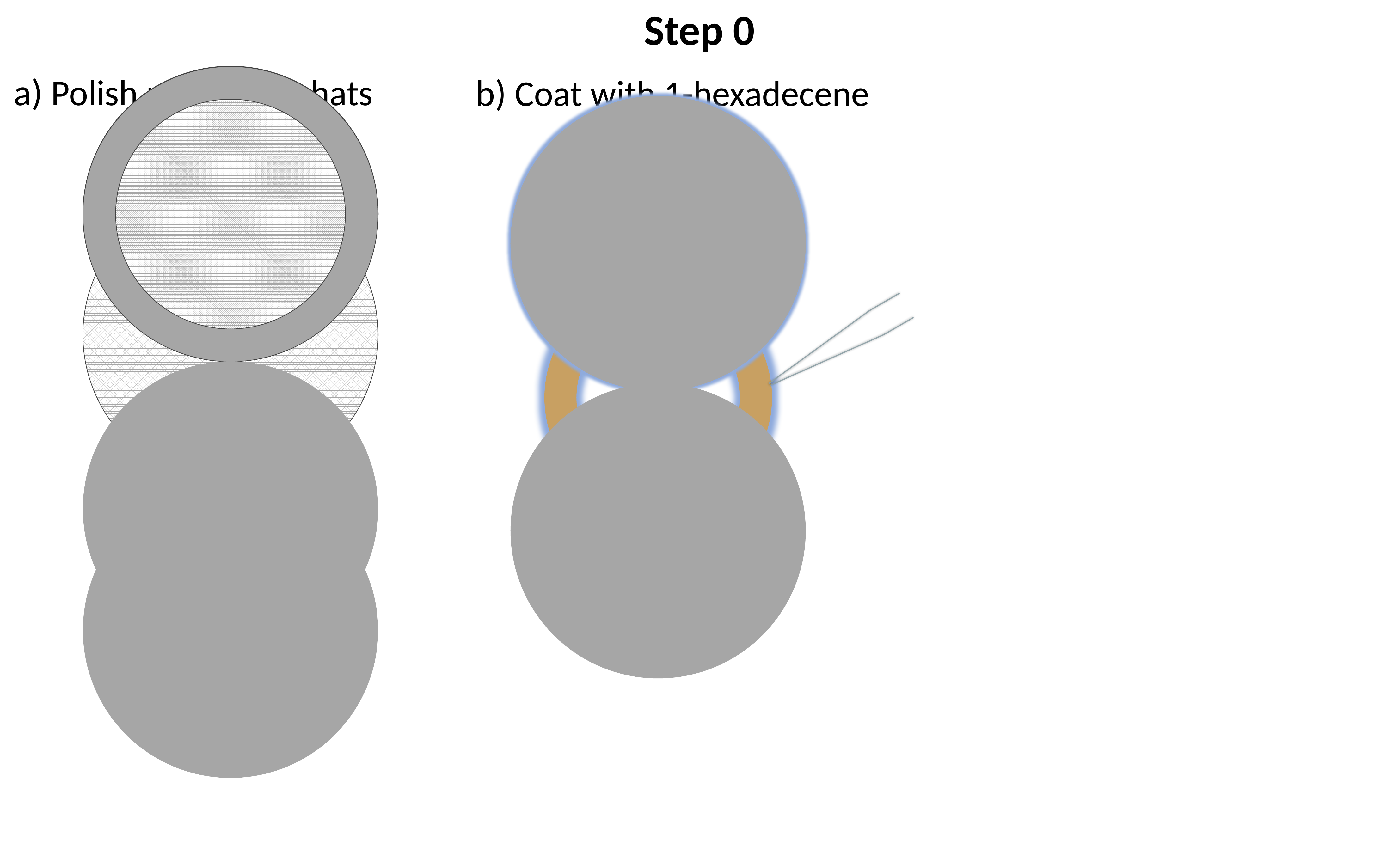

Step 0
a) Polish planchette hats
b) Coat with 1-hexadecene

## Slide 15
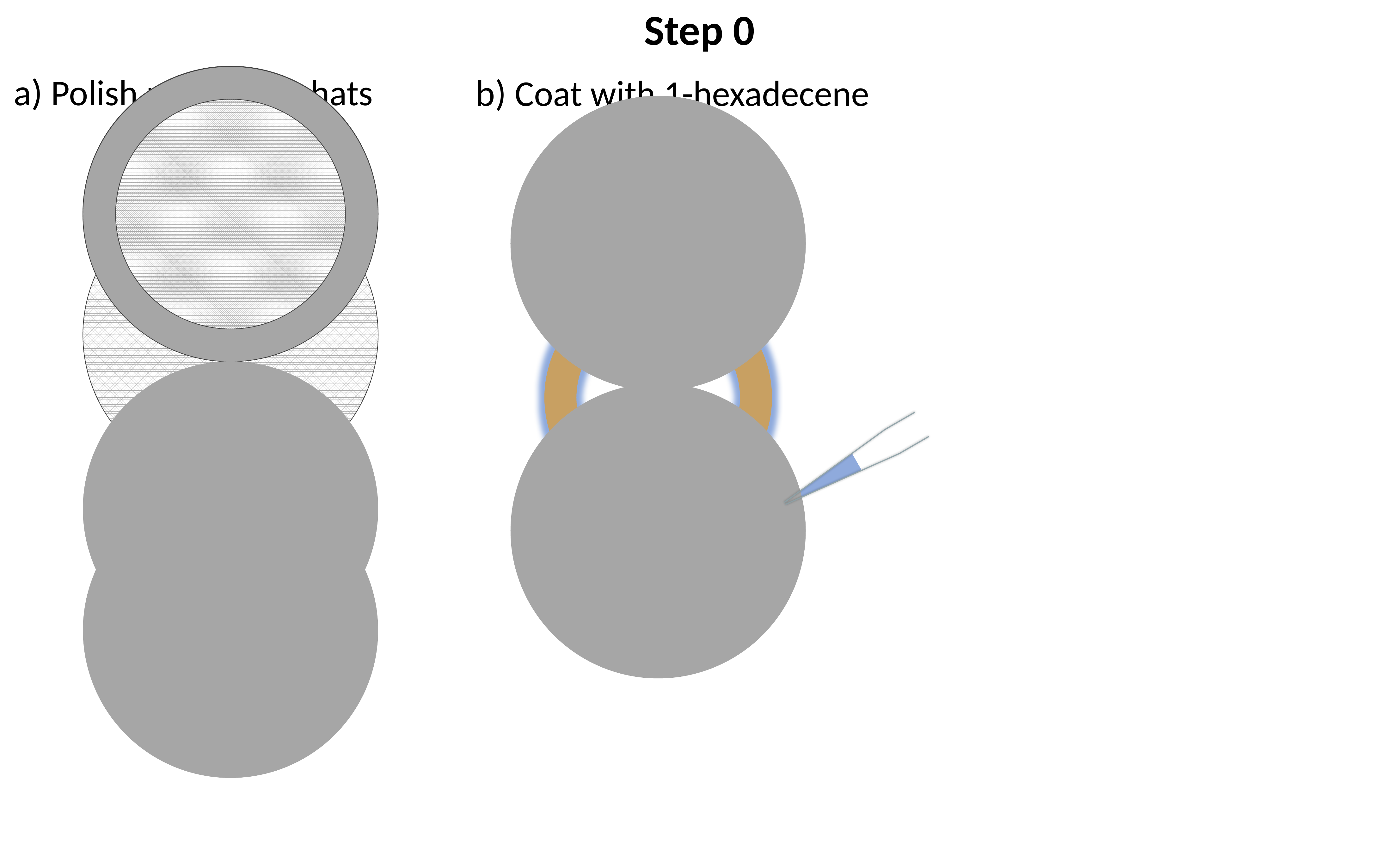

Step 0
a) Polish planchette hats
b) Coat with 1-hexadecene

## Slide 16
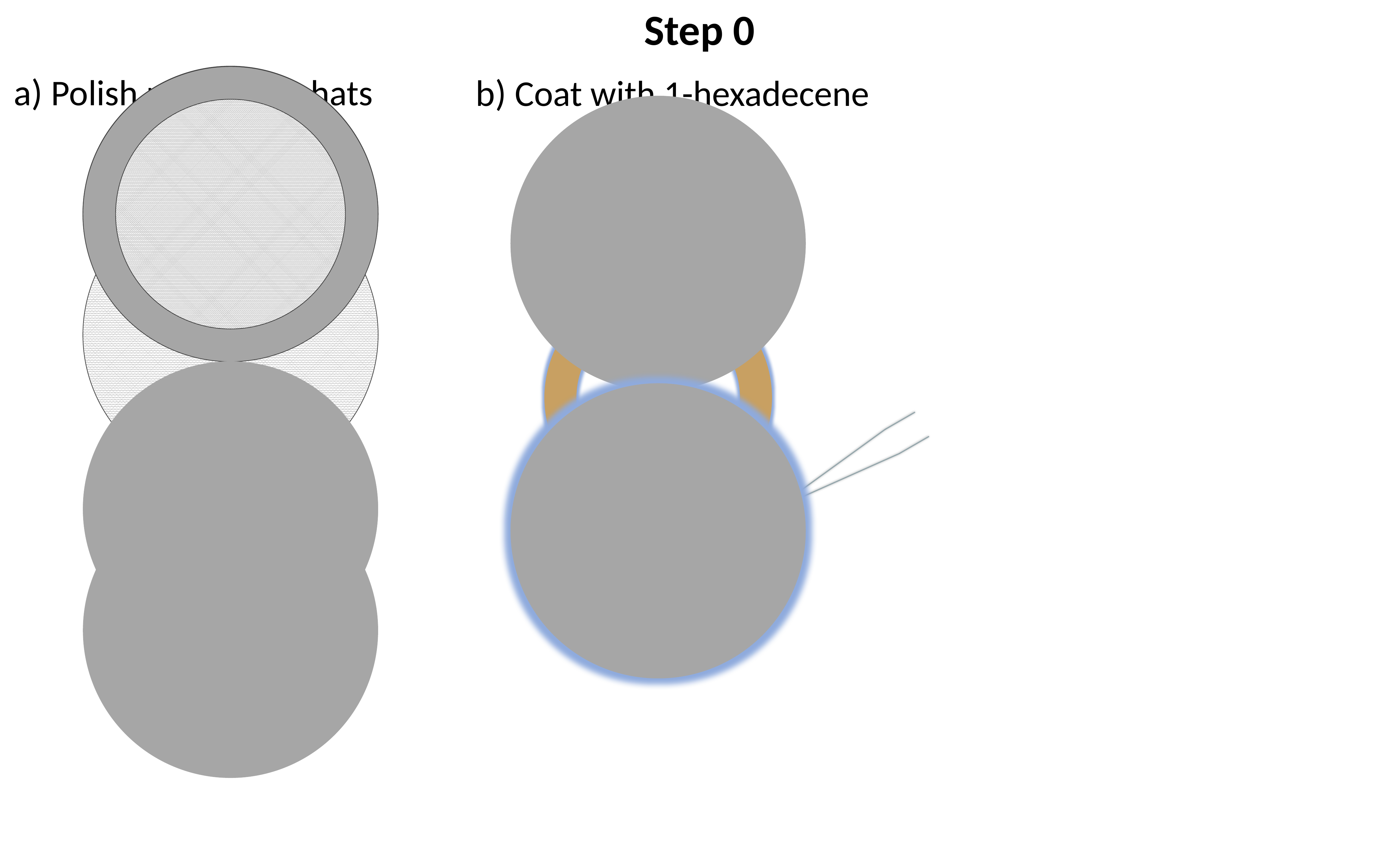

Step 0
a) Polish planchette hats
b) Coat with 1-hexadecene

## Slide 17
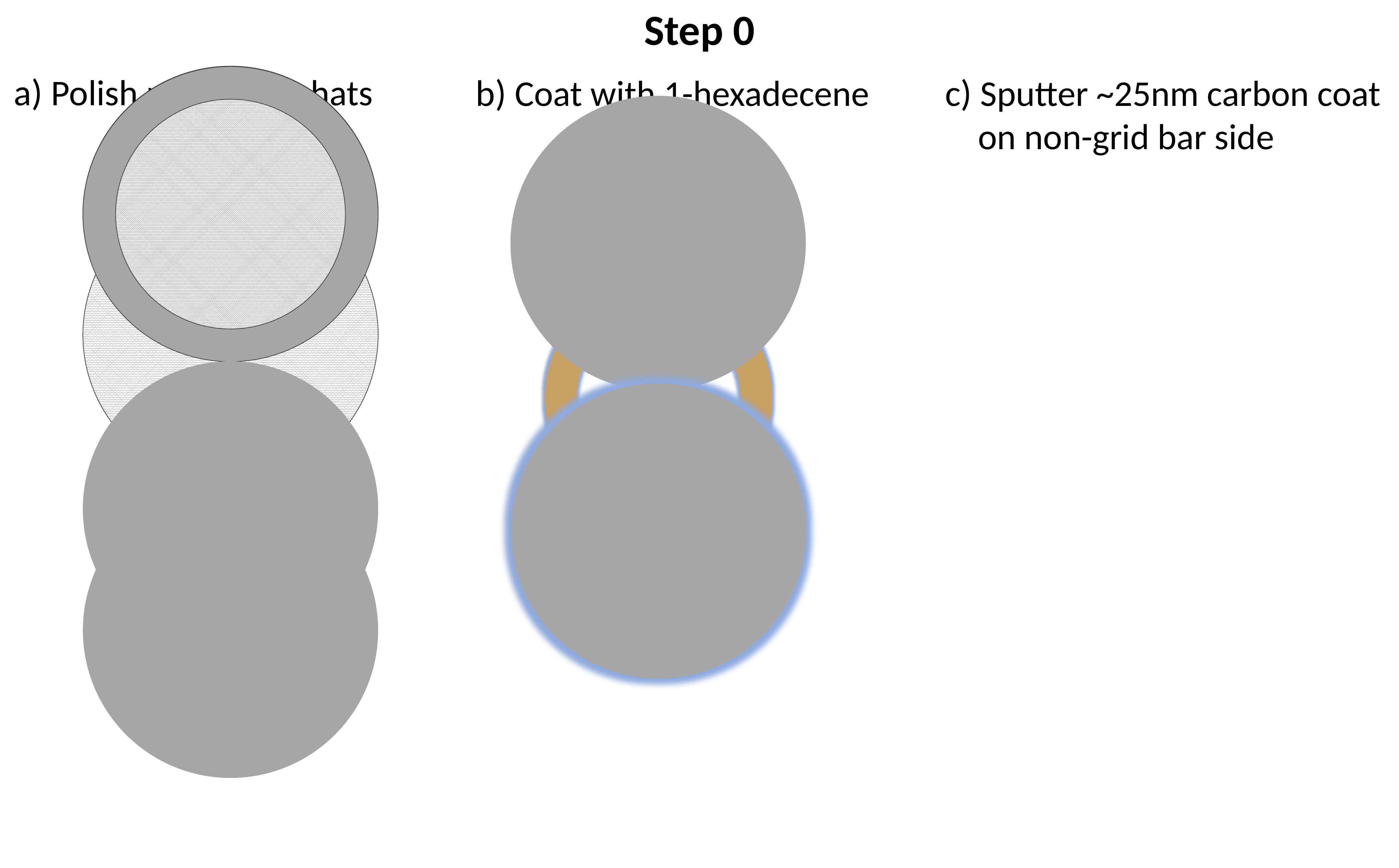

Step 0
a) Polish planchette hats
b) Coat with 1-hexadecene
c) Sputter ~25nm carbon coat
 on non-grid bar side

## Slide 18
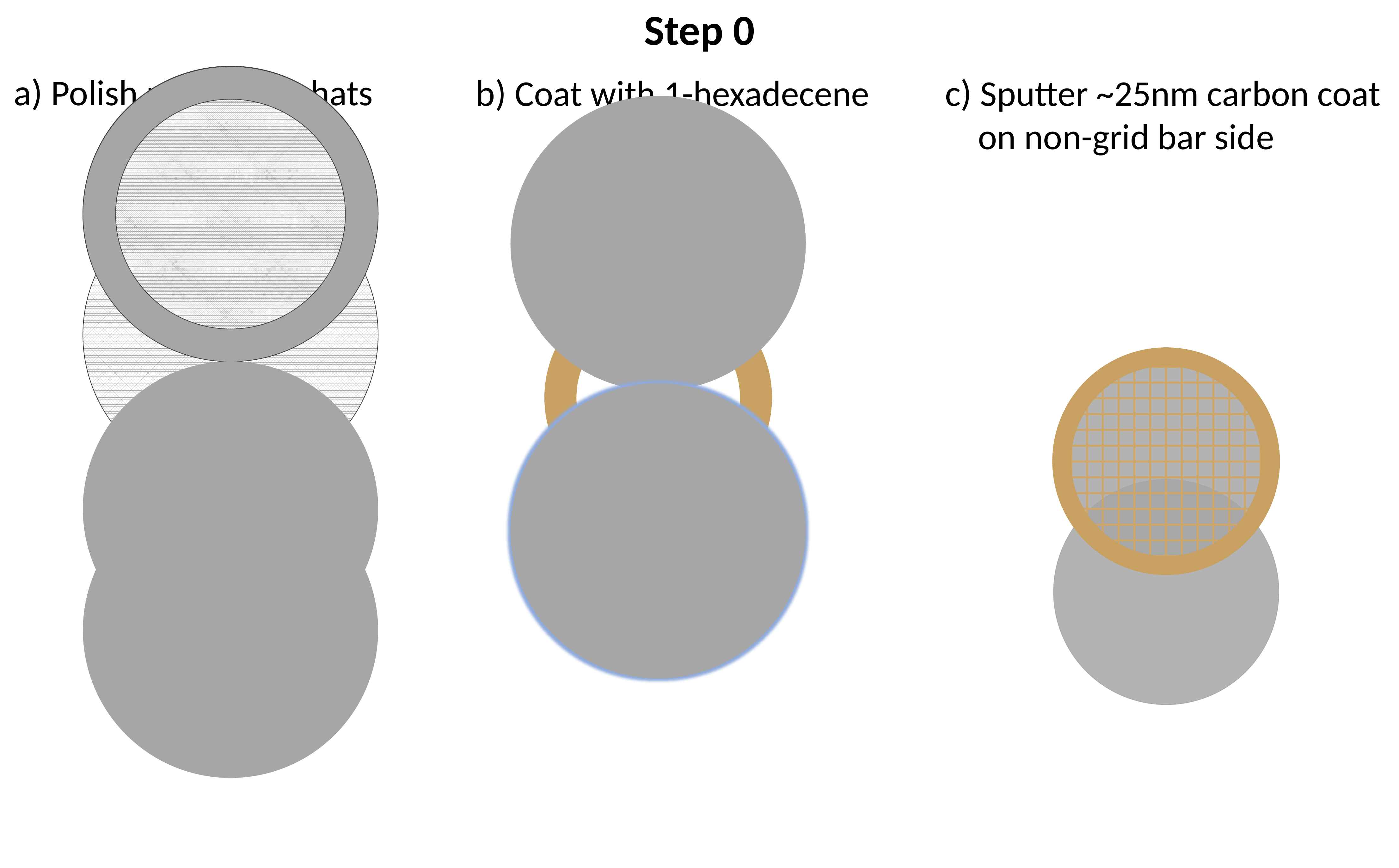

Step 0
a) Polish planchette hats
b) Coat with 1-hexadecene
c) Sputter ~25nm carbon coat
 on non-grid bar side

## Slide 19
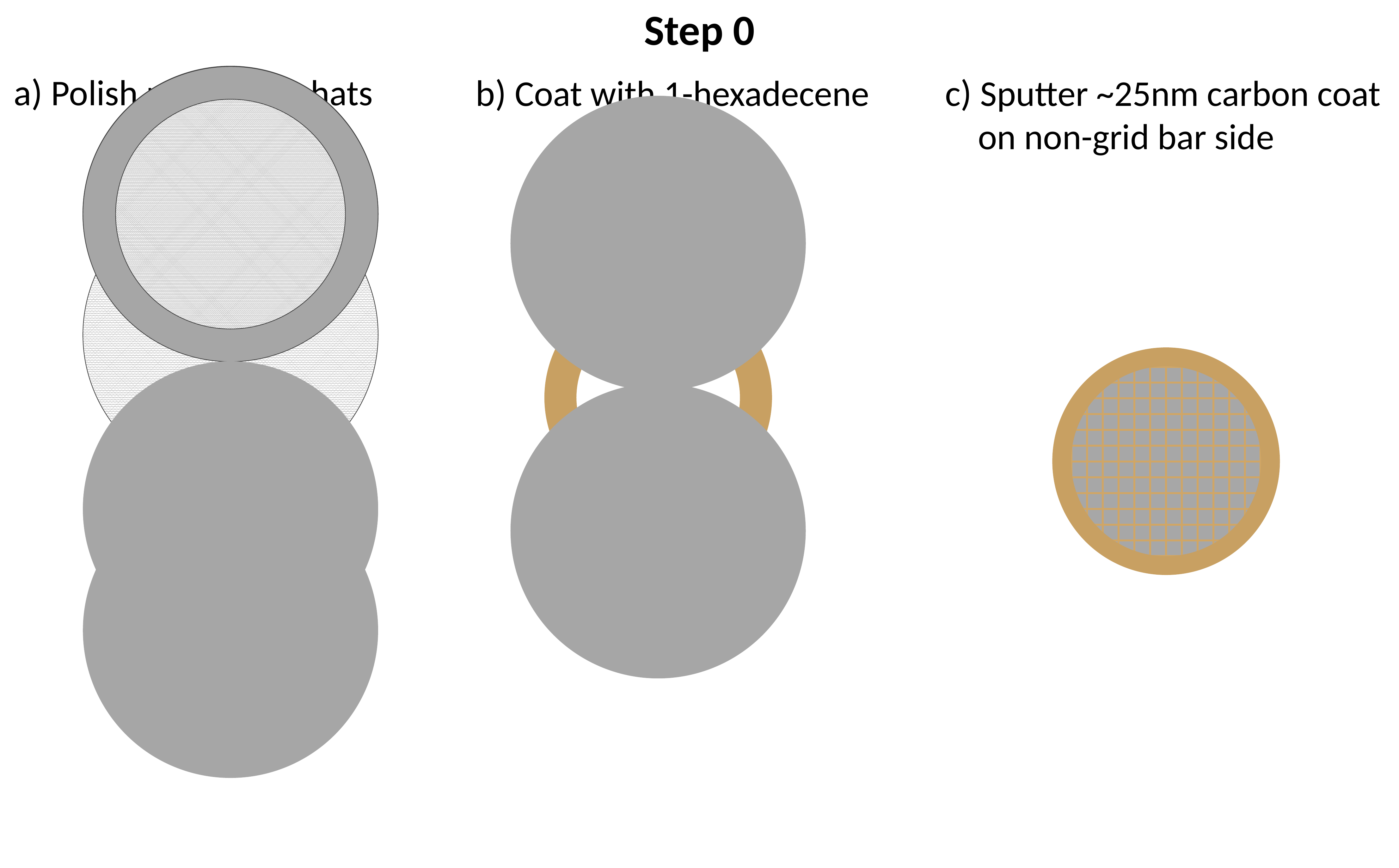

Step 0
a) Polish planchette hats
b) Coat with 1-hexadecene
c) Sputter ~25nm carbon coat
 on non-grid bar side

## Slide 20
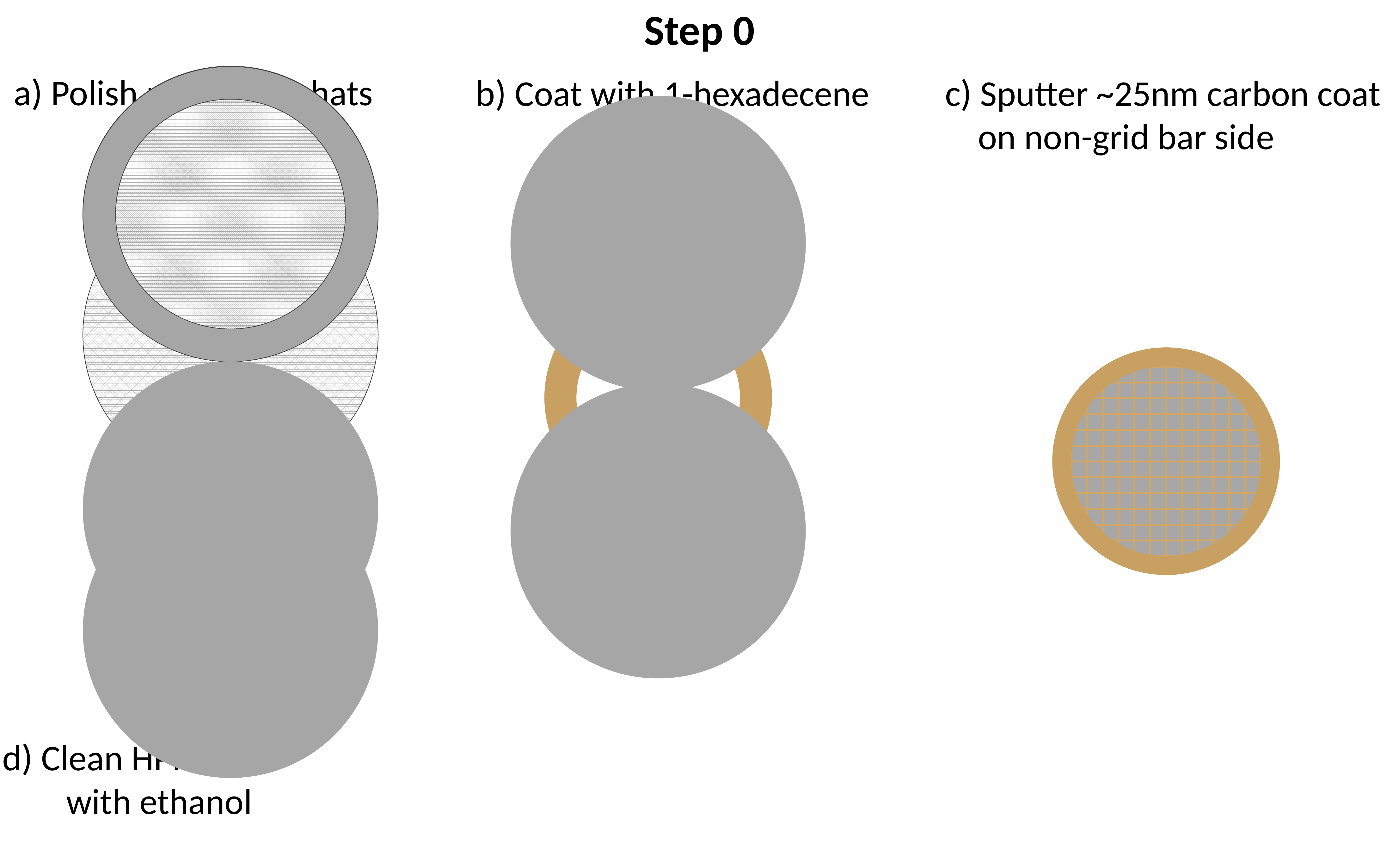

Step 0
a) Polish planchette hats
b) Coat with 1-hexadecene
c) Sputter ~25nm carbon coat
 on non-grid bar side
d) Clean HPF tip
	 with ethanol

## Slide 21
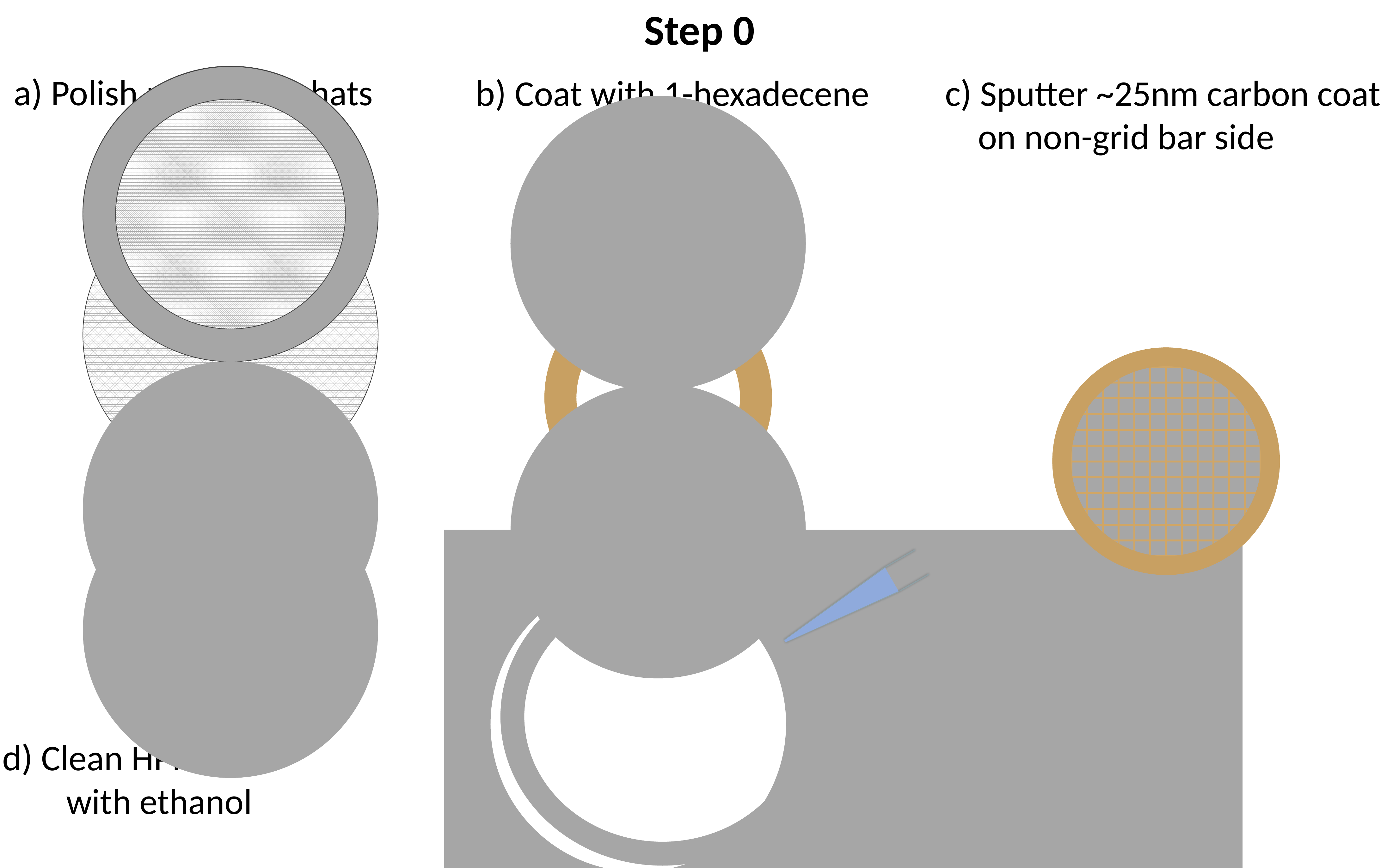

Step 0
a) Polish planchette hats
b) Coat with 1-hexadecene
c) Sputter ~25nm carbon coat
 on non-grid bar side
d) Clean HPF tip
	 with ethanol

## Slide 22
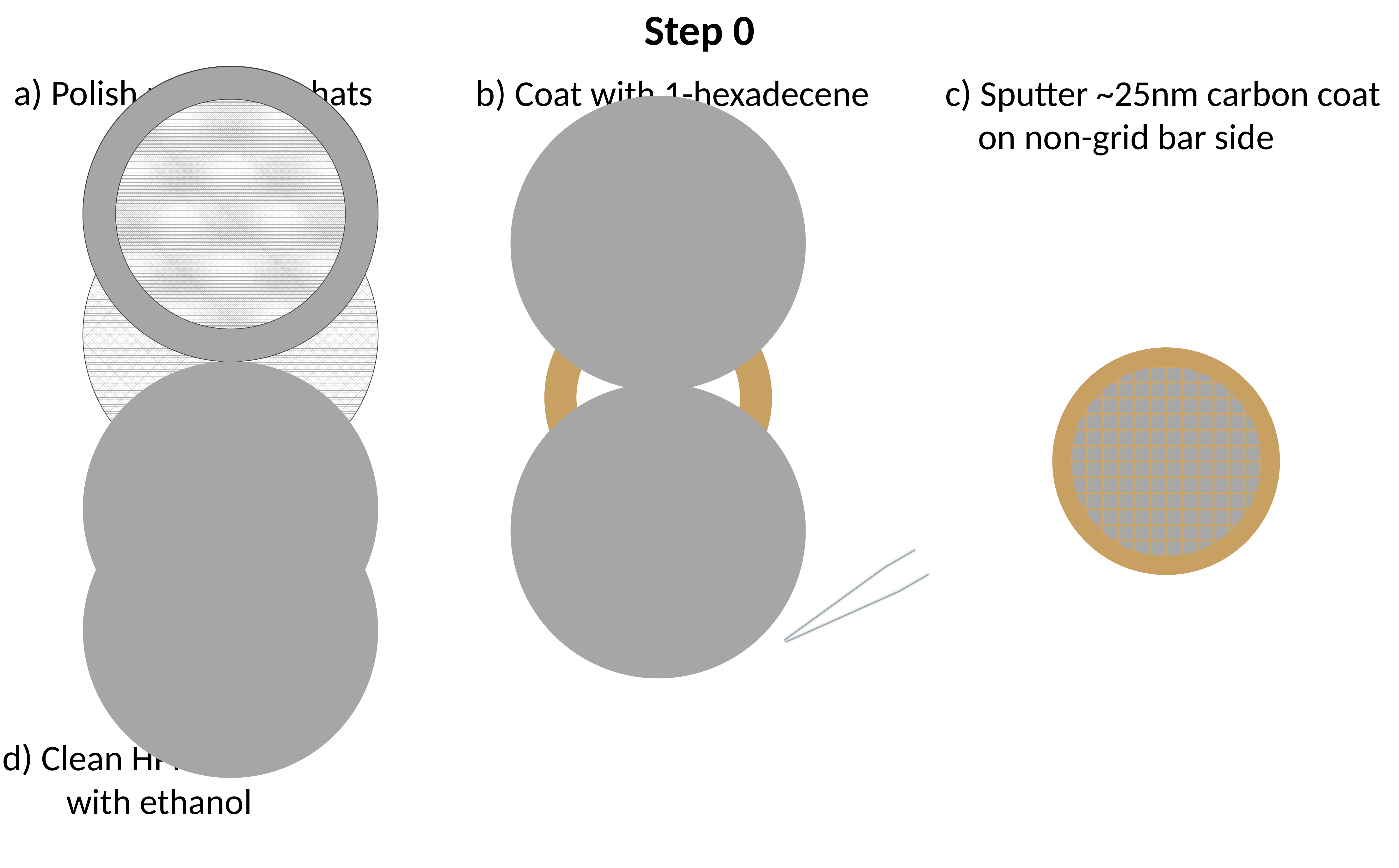

Step 0
a) Polish planchette hats
b) Coat with 1-hexadecene
c) Sputter ~25nm carbon coat
 on non-grid bar side
d) Clean HPF tip
	 with ethanol

## Slide 23
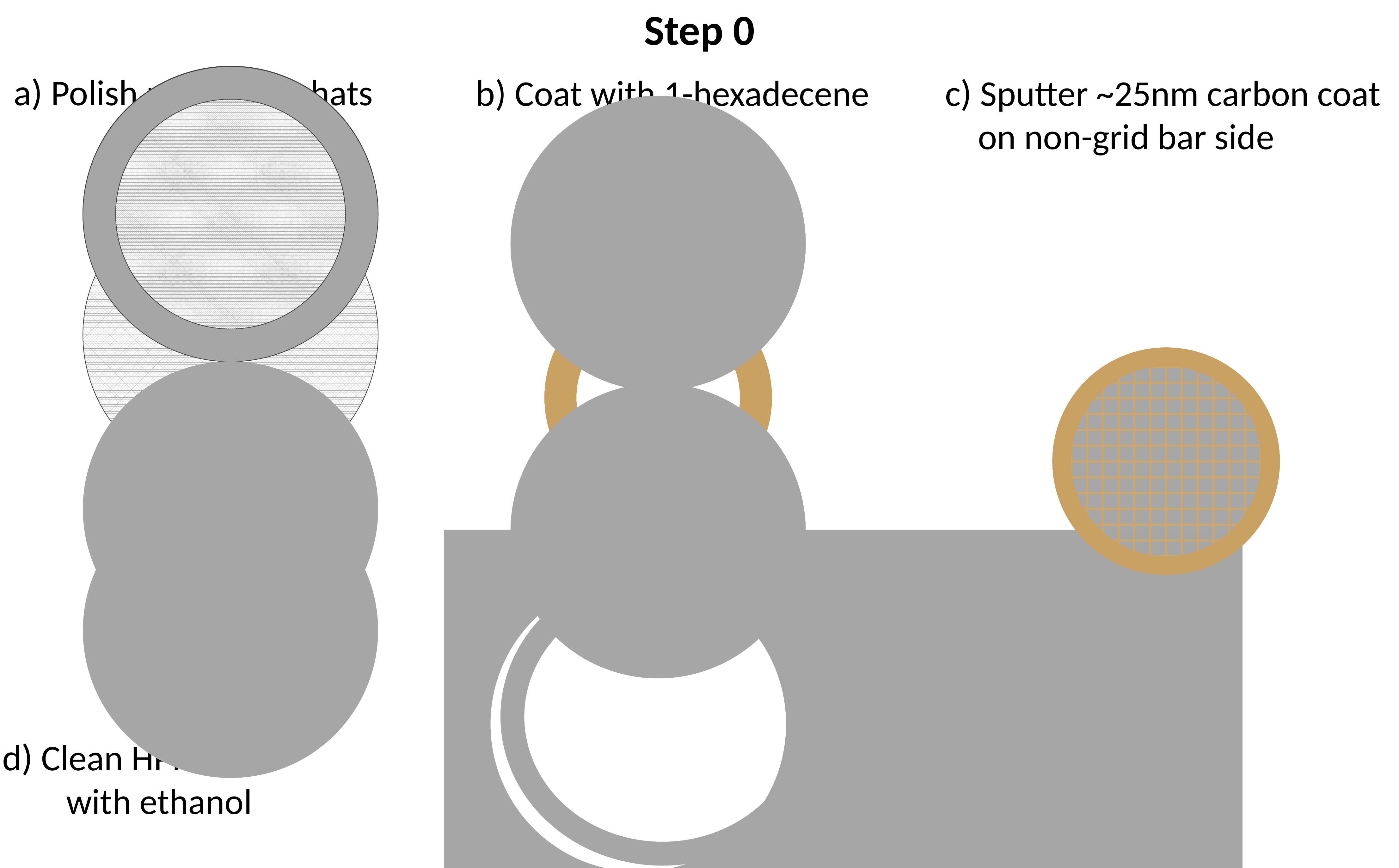

Step 0
a) Polish planchette hats
b) Coat with 1-hexadecene
c) Sputter ~25nm carbon coat
 on non-grid bar side
d) Clean HPF tip
	 with ethanol

## Slide 24
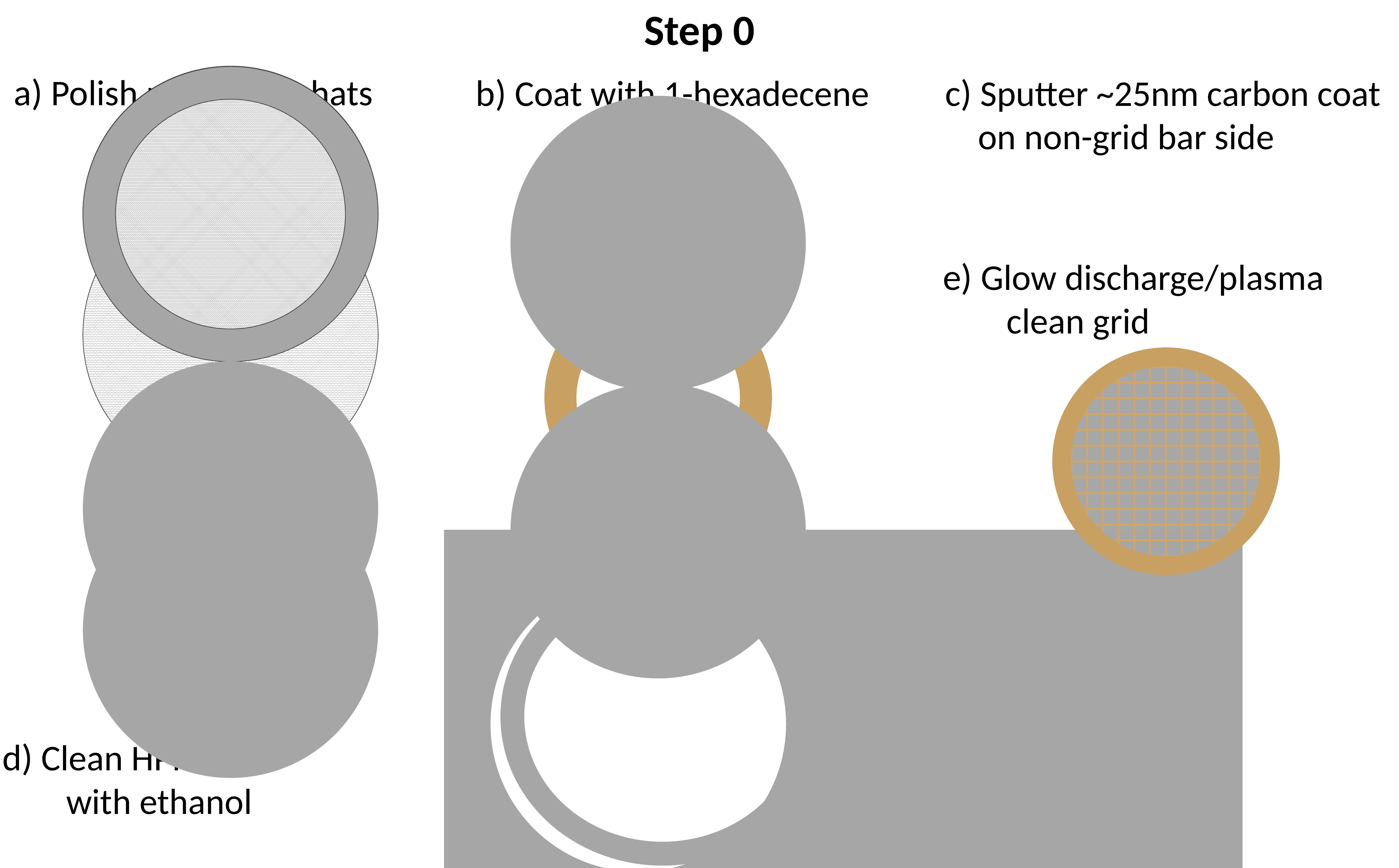

Step 0
a) Polish planchette hats
b) Coat with 1-hexadecene
c) Sputter ~25nm carbon coat
 on non-grid bar side
e) Glow discharge/plasma
	 clean grid
d) Clean HPF tip
	 with ethanol

## Slide 25
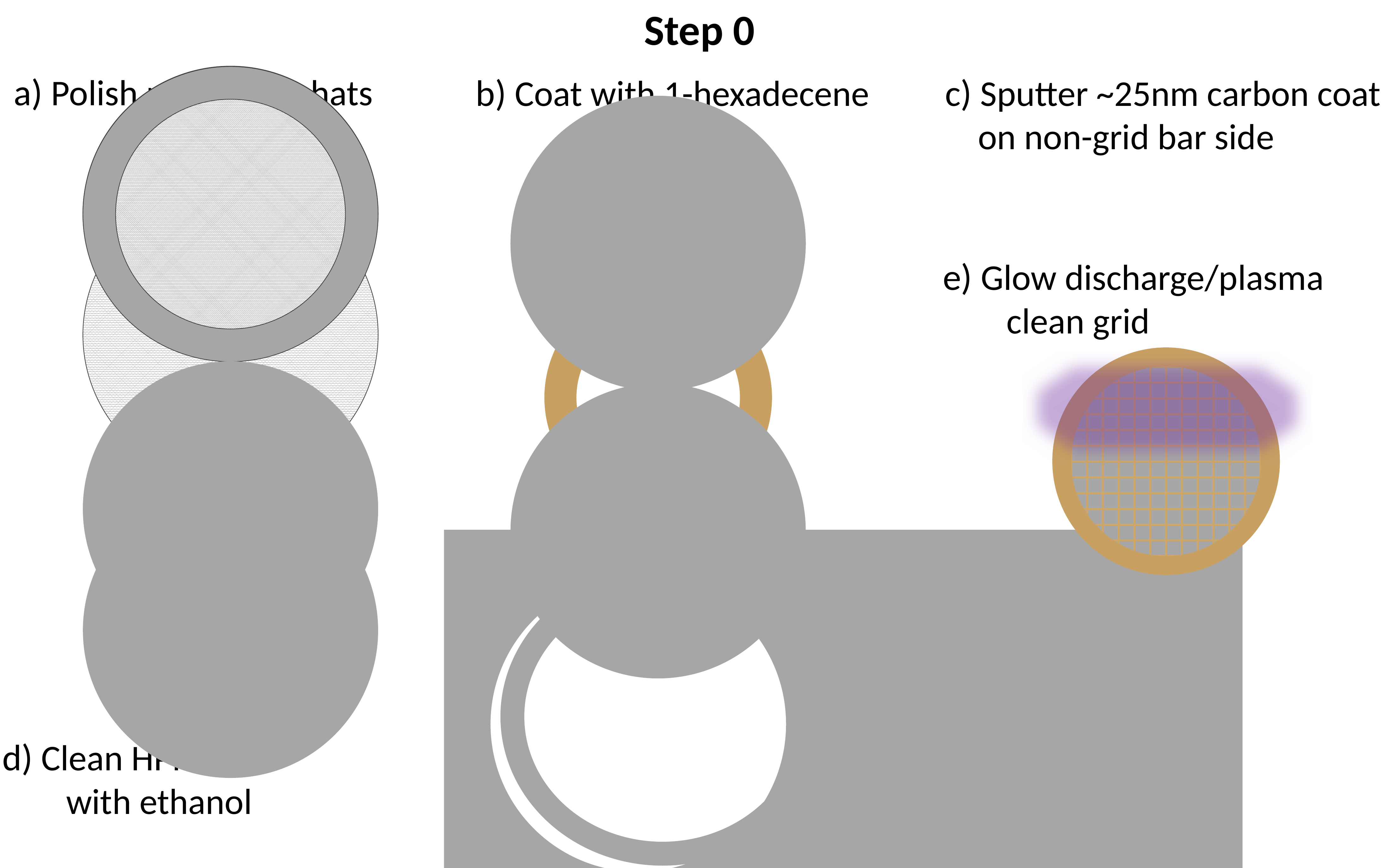

Step 0
a) Polish planchette hats
b) Coat with 1-hexadecene
c) Sputter ~25nm carbon coat
 on non-grid bar side
e) Glow discharge/plasma
	 clean grid
d) Clean HPF tip
	 with ethanol

## Slide 26
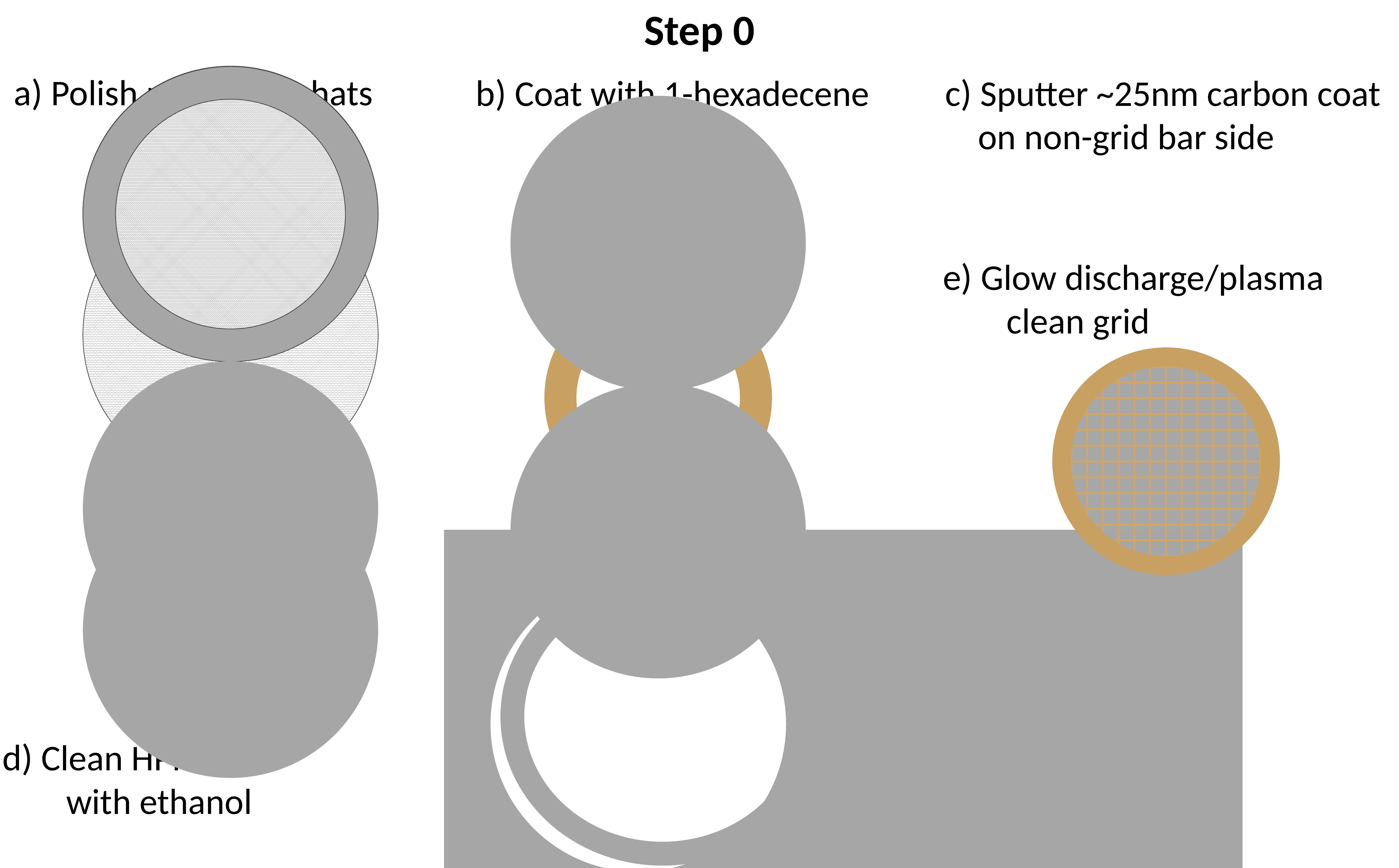

Step 0
a) Polish planchette hats
b) Coat with 1-hexadecene
c) Sputter ~25nm carbon coat
 on non-grid bar side
e) Glow discharge/plasma
	 clean grid
d) Clean HPF tip
	 with ethanol

## Slide 27
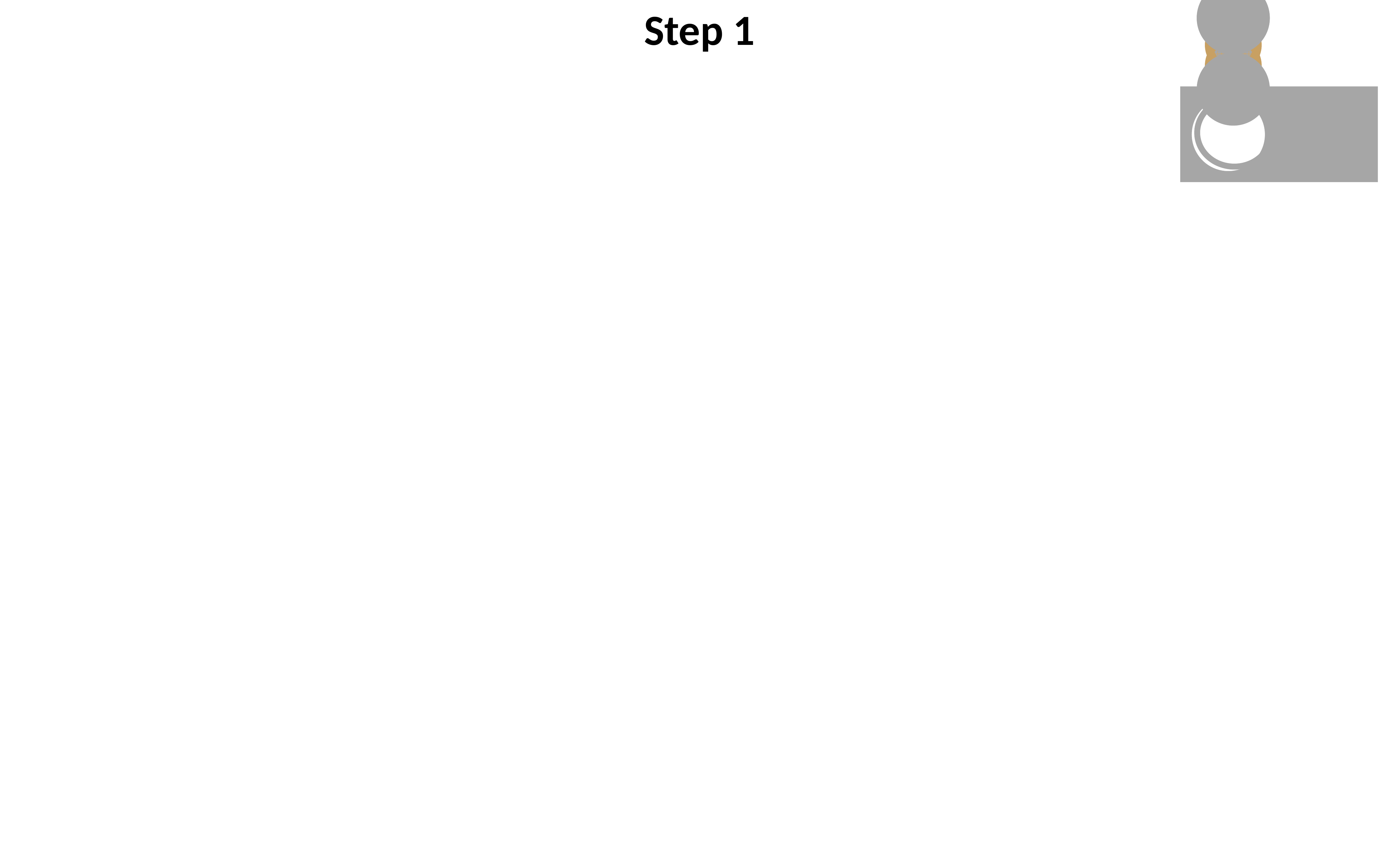

Step 1

## Slide 28
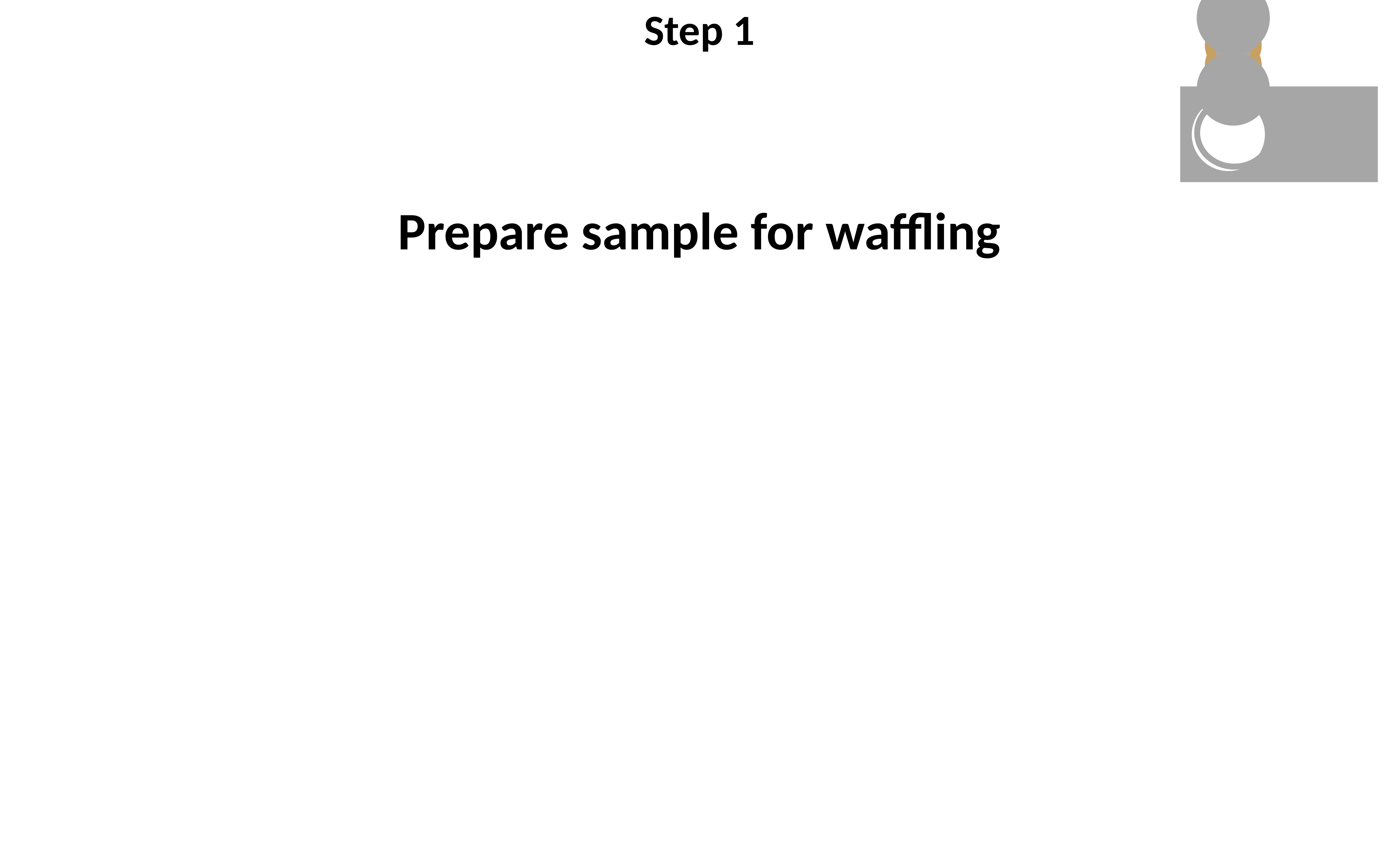

Step 1
Prepare sample for waffling

## Slide 29
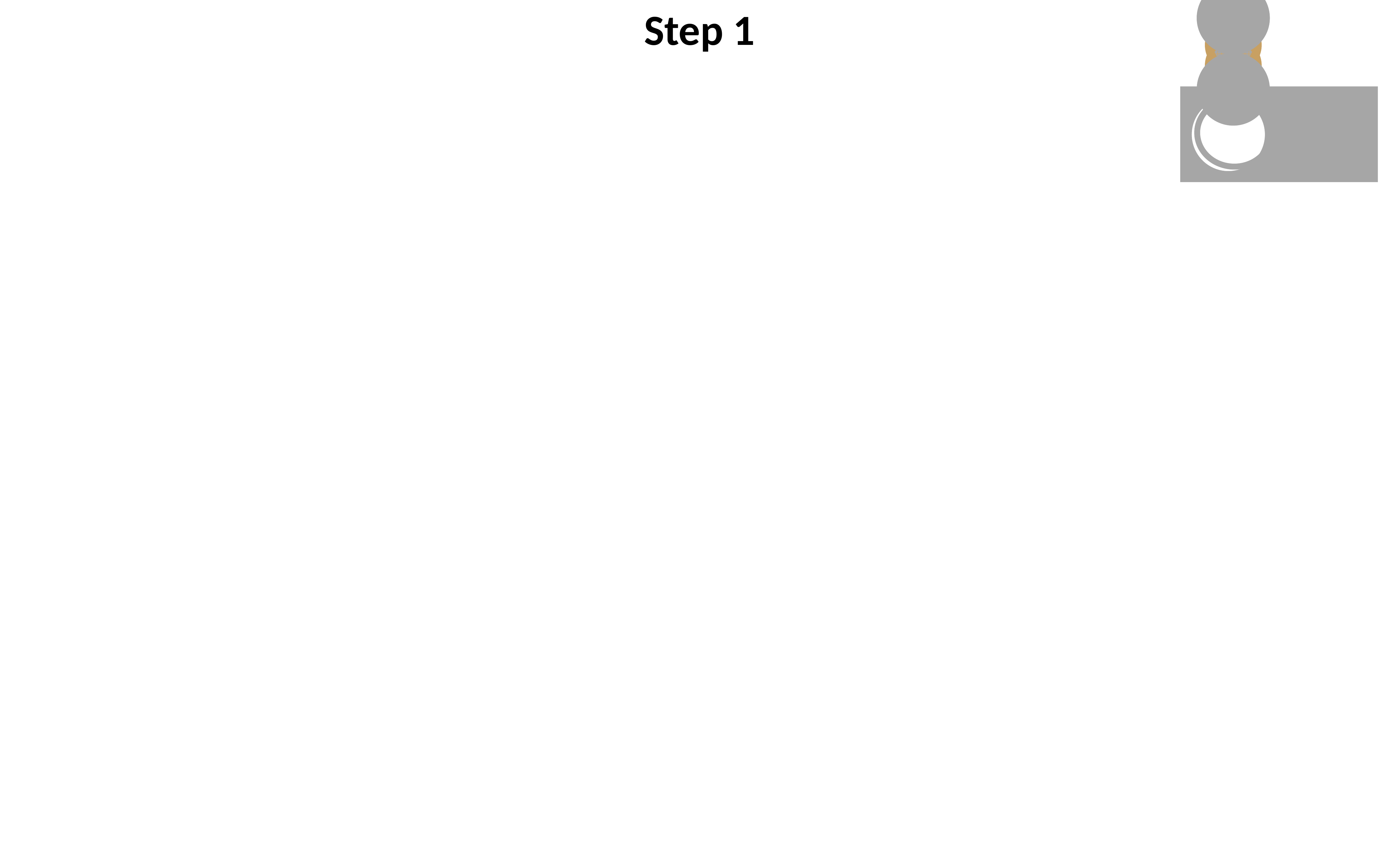

Step 1

## Slide 30
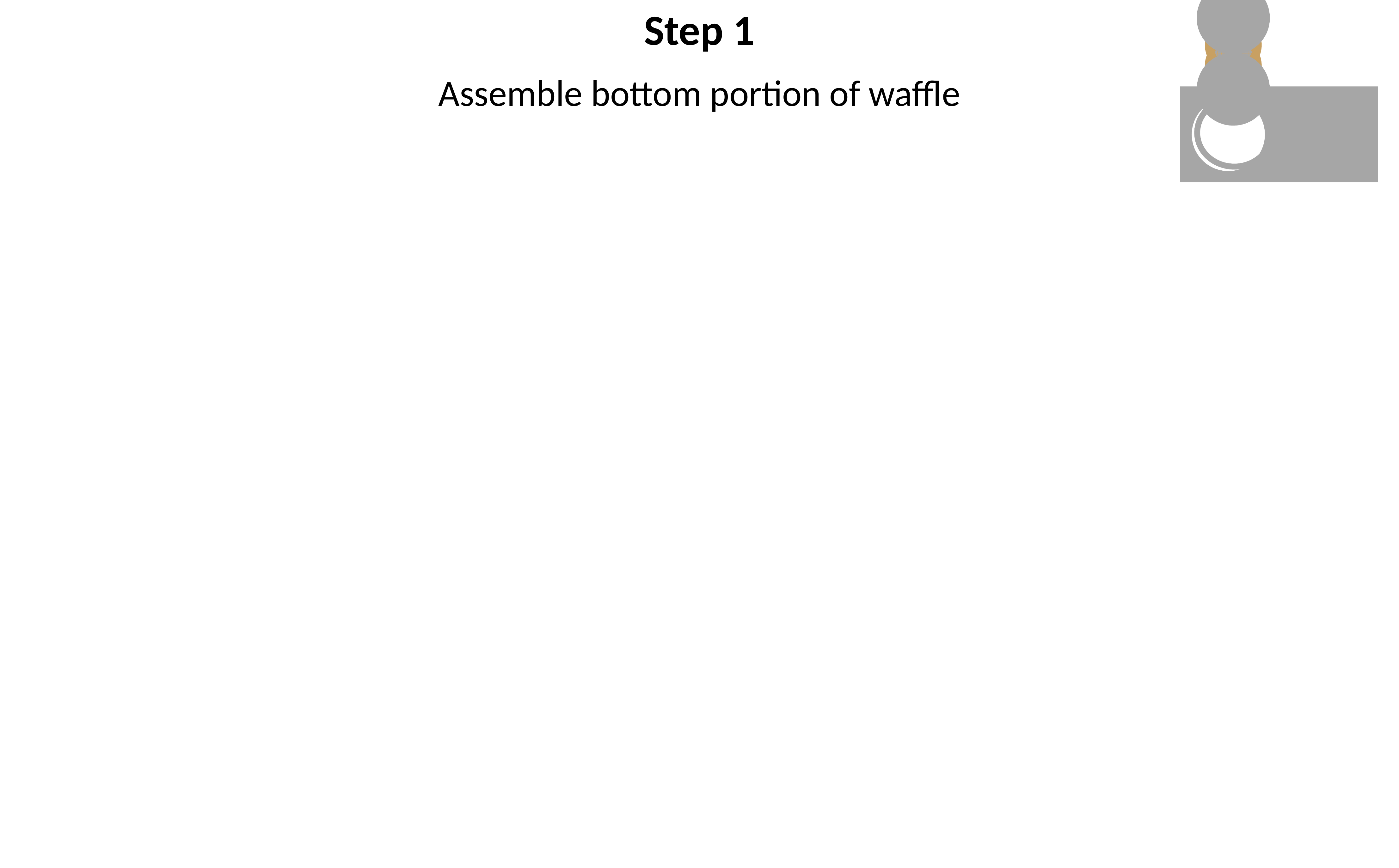

Step 1
Assemble bottom portion of waffle

## Slide 31
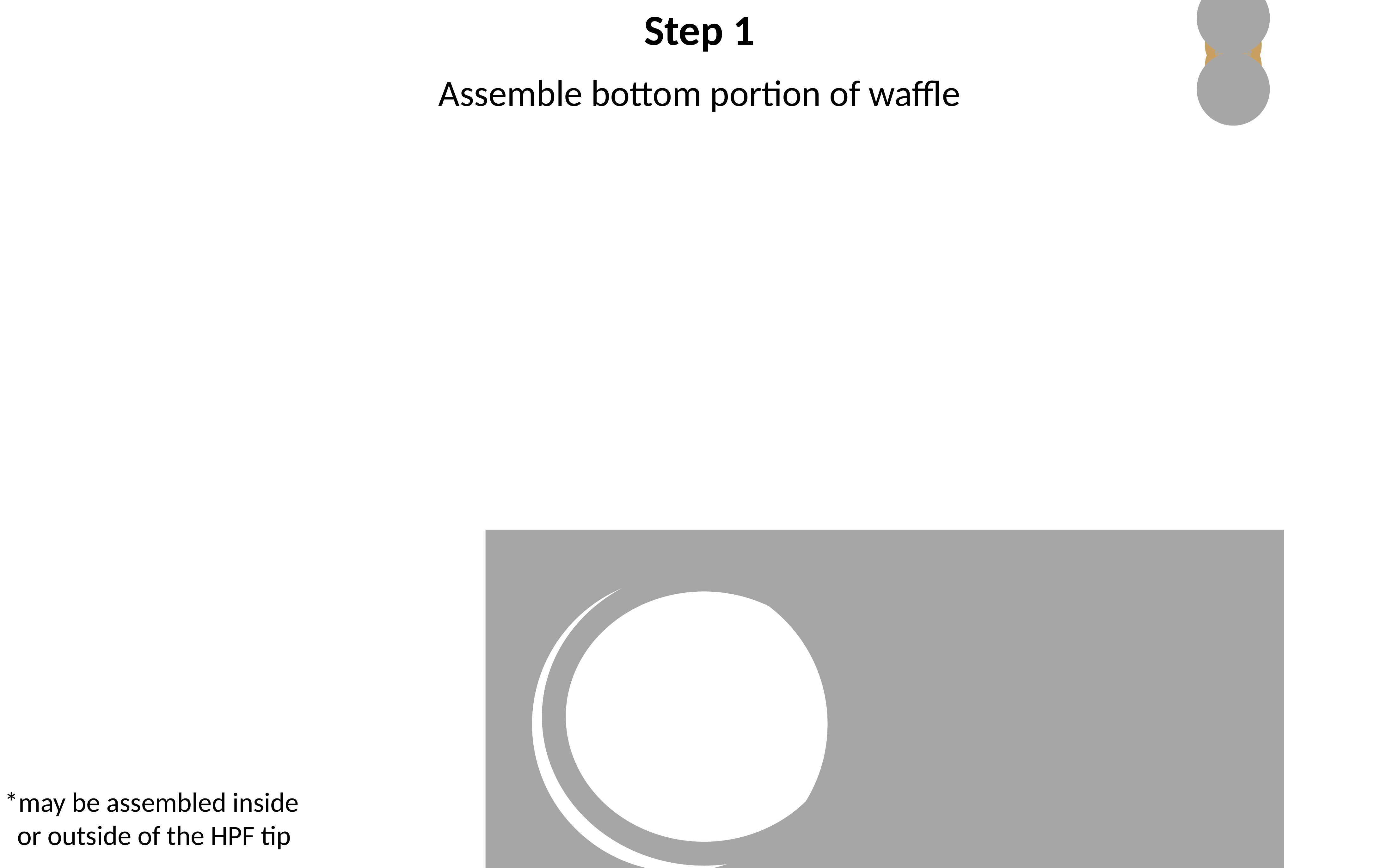

Step 1
Assemble bottom portion of waffle
*may be assembled inside
 or outside of the HPF tip

## Slide 32
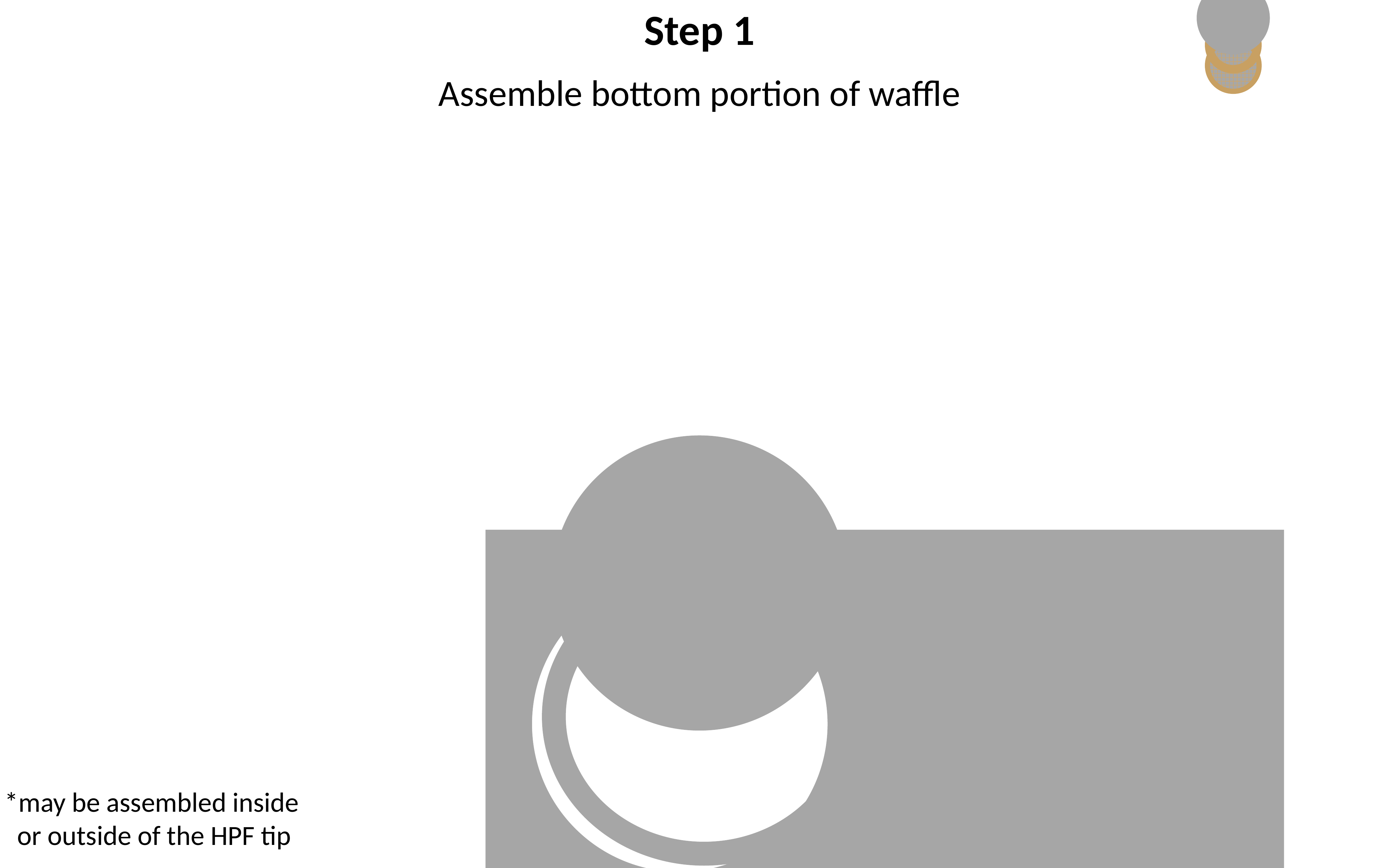

Step 1
Assemble bottom portion of waffle
*may be assembled inside
 or outside of the HPF tip

## Slide 33
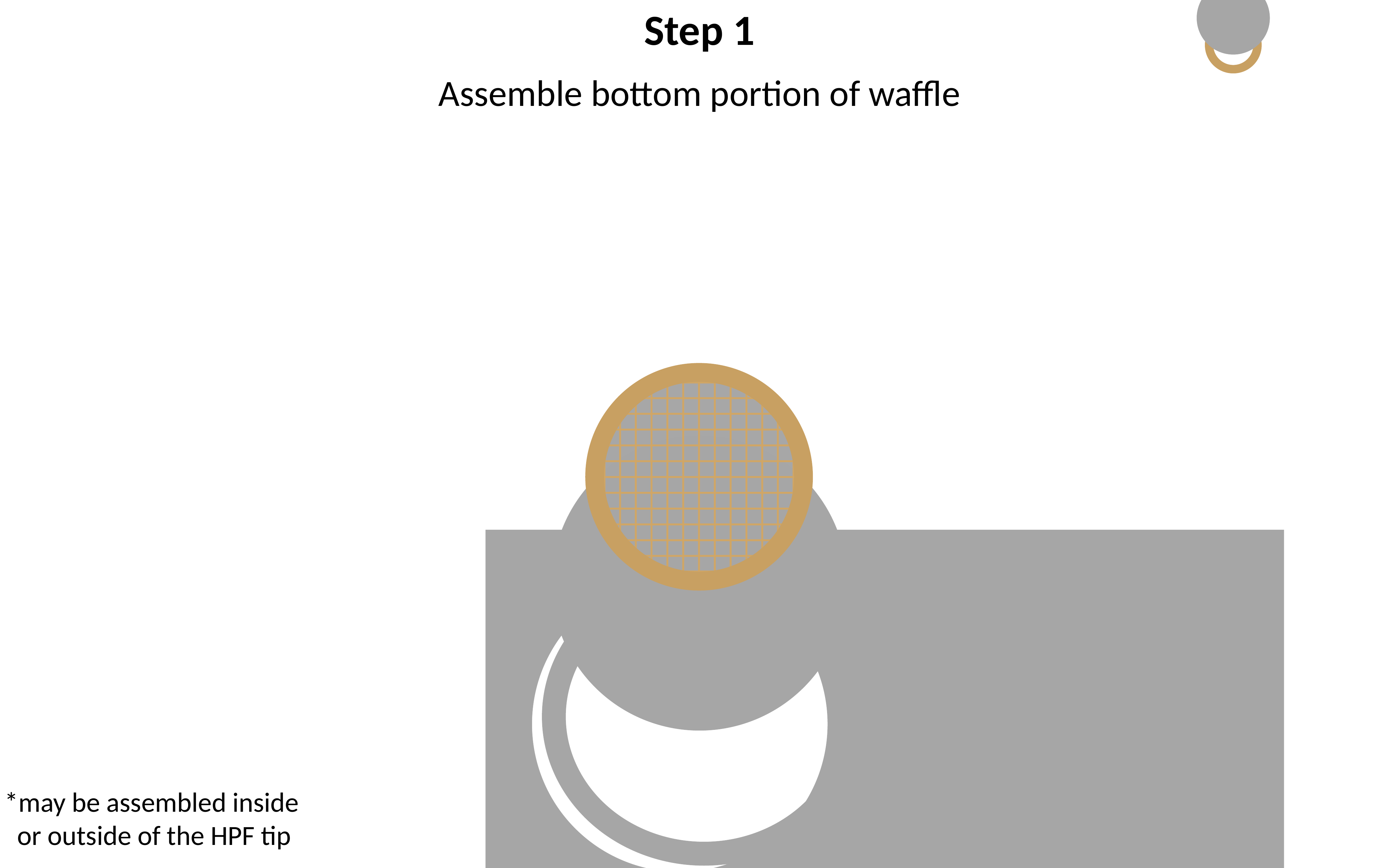

Step 1
Assemble bottom portion of waffle
*may be assembled inside
 or outside of the HPF tip

## Slide 34
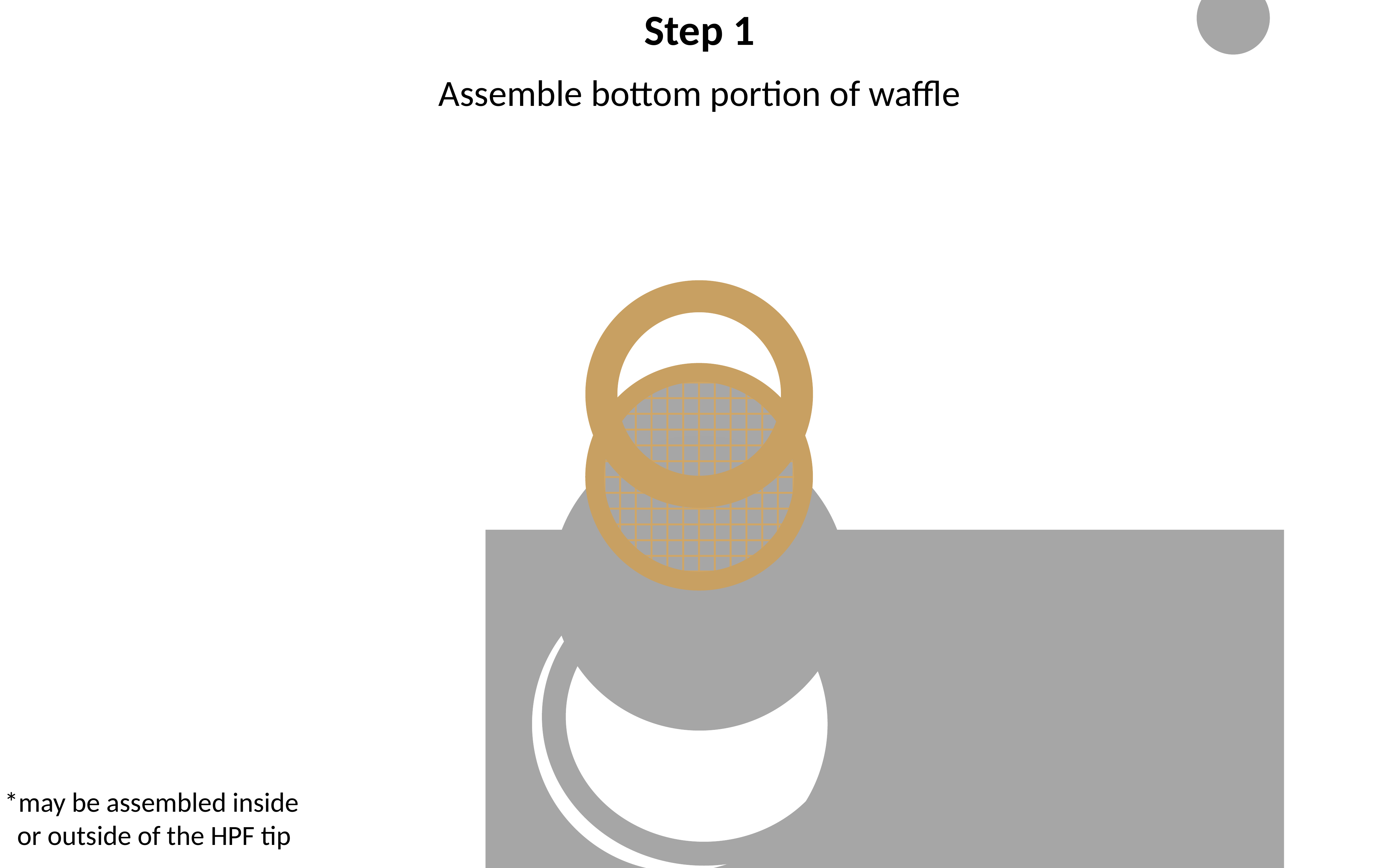

Step 1
Assemble bottom portion of waffle
*may be assembled inside
 or outside of the HPF tip

## Slide 35
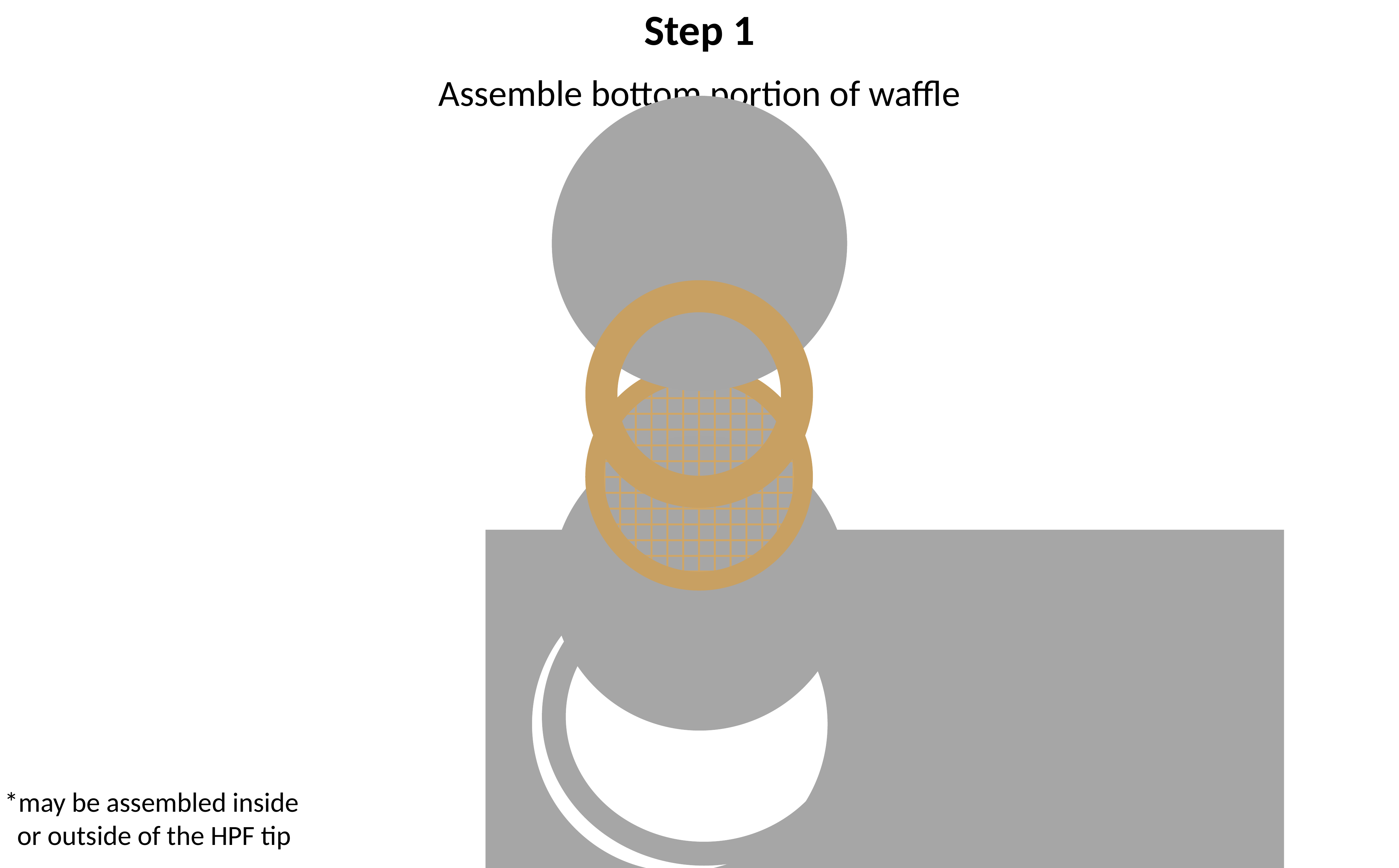

Step 1
Assemble bottom portion of waffle
*may be assembled inside
 or outside of the HPF tip

## Slide 36
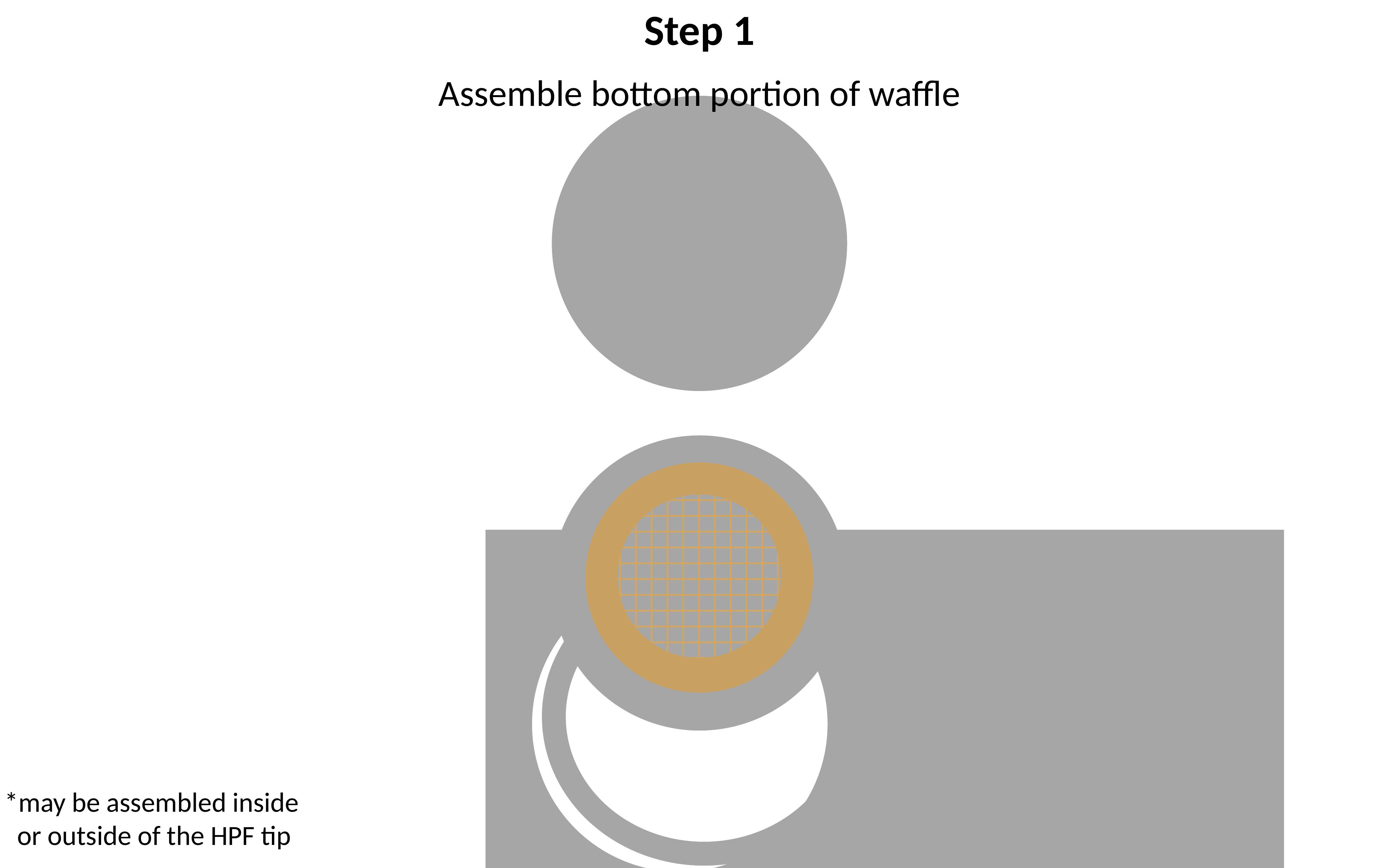

Step 1
Assemble bottom portion of waffle
*may be assembled inside
 or outside of the HPF tip

## Slide 37
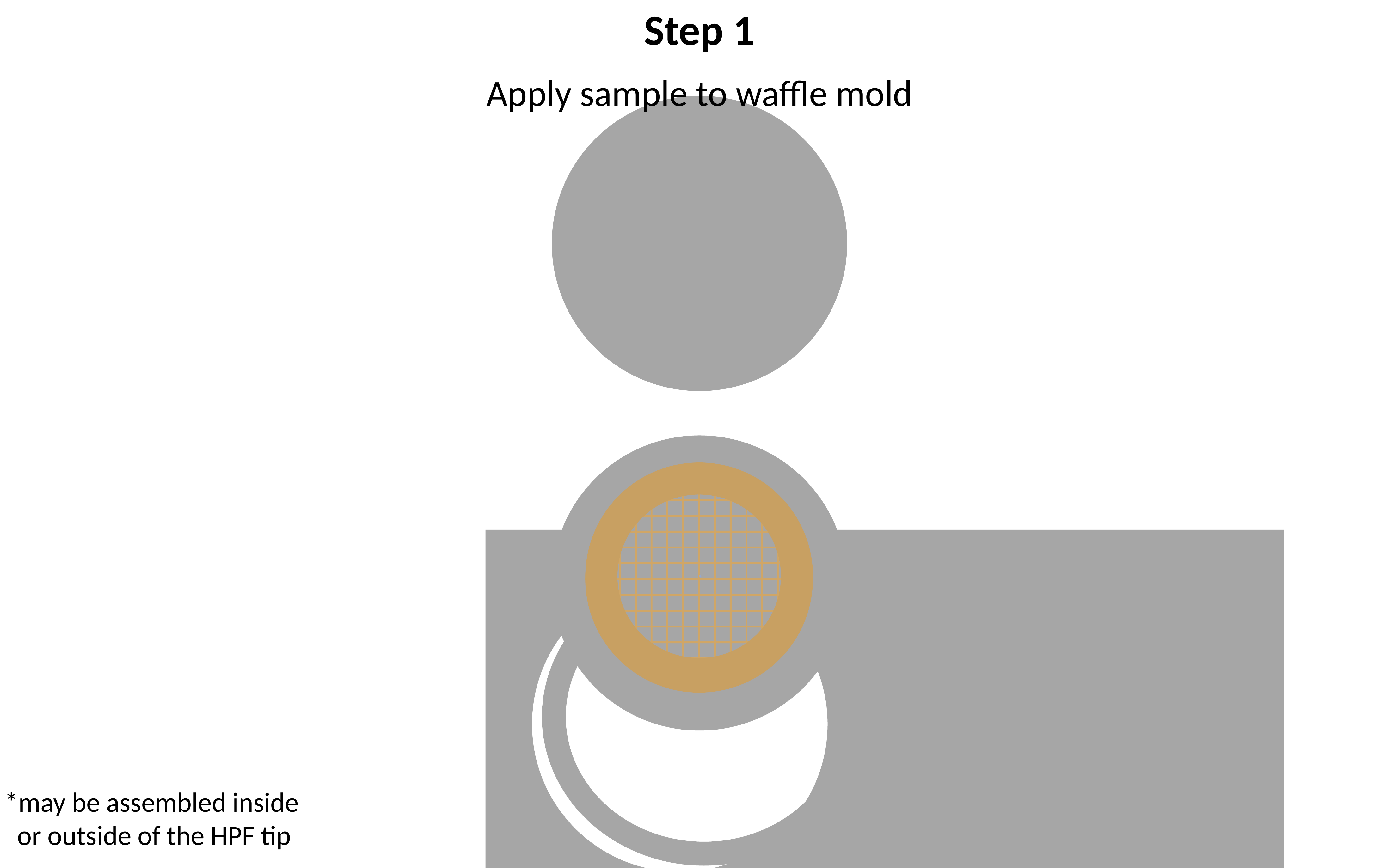

Step 1
Apply sample to waffle mold
*may be assembled inside
 or outside of the HPF tip

## Slide 38
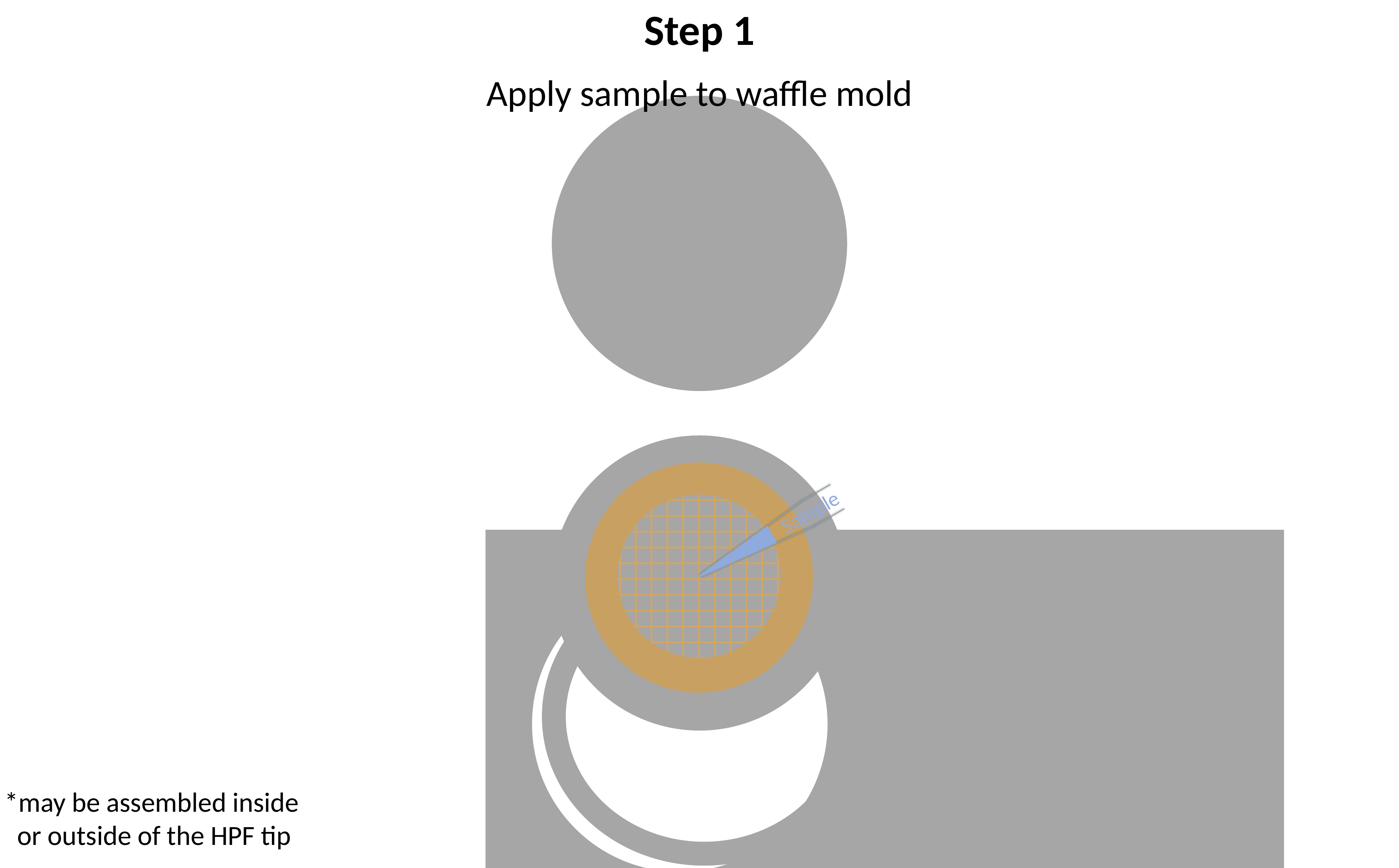

Step 1
Apply sample to waffle mold
Sample
*may be assembled inside
 or outside of the HPF tip

## Slide 39
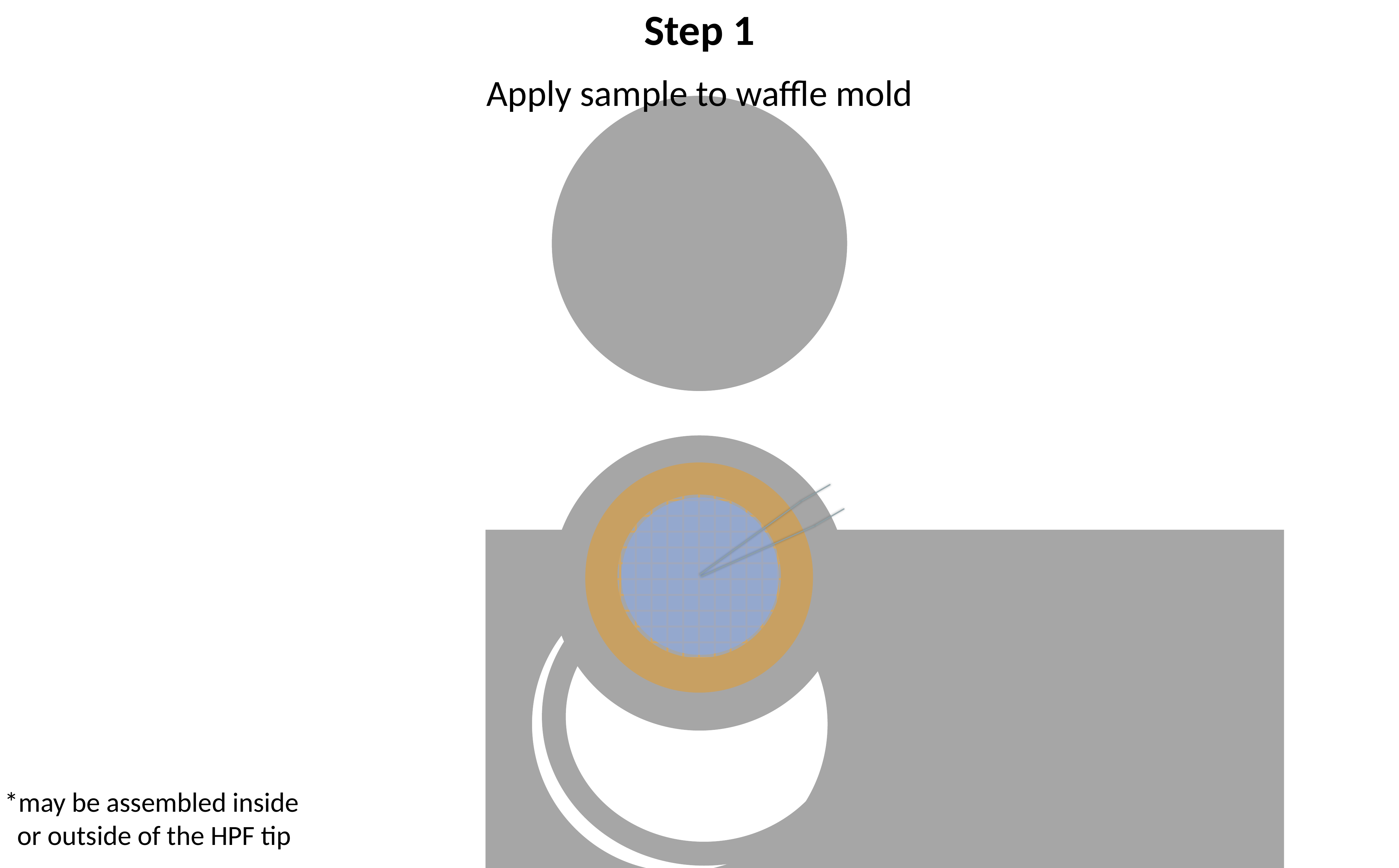

Step 1
Apply sample to waffle mold
*may be assembled inside
 or outside of the HPF tip

## Slide 40
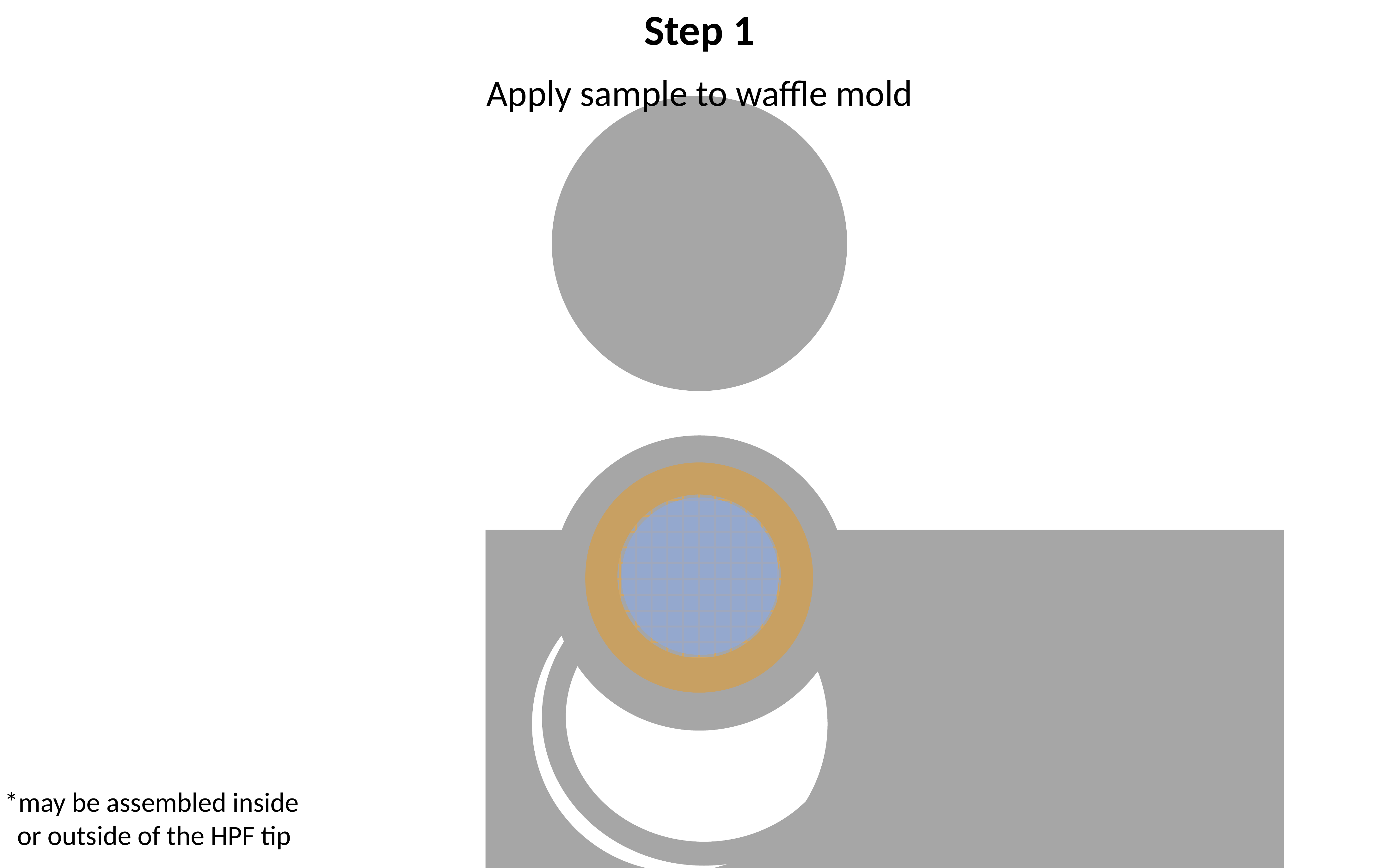

Step 1
Apply sample to waffle mold
*may be assembled inside
 or outside of the HPF tip

## Slide 41
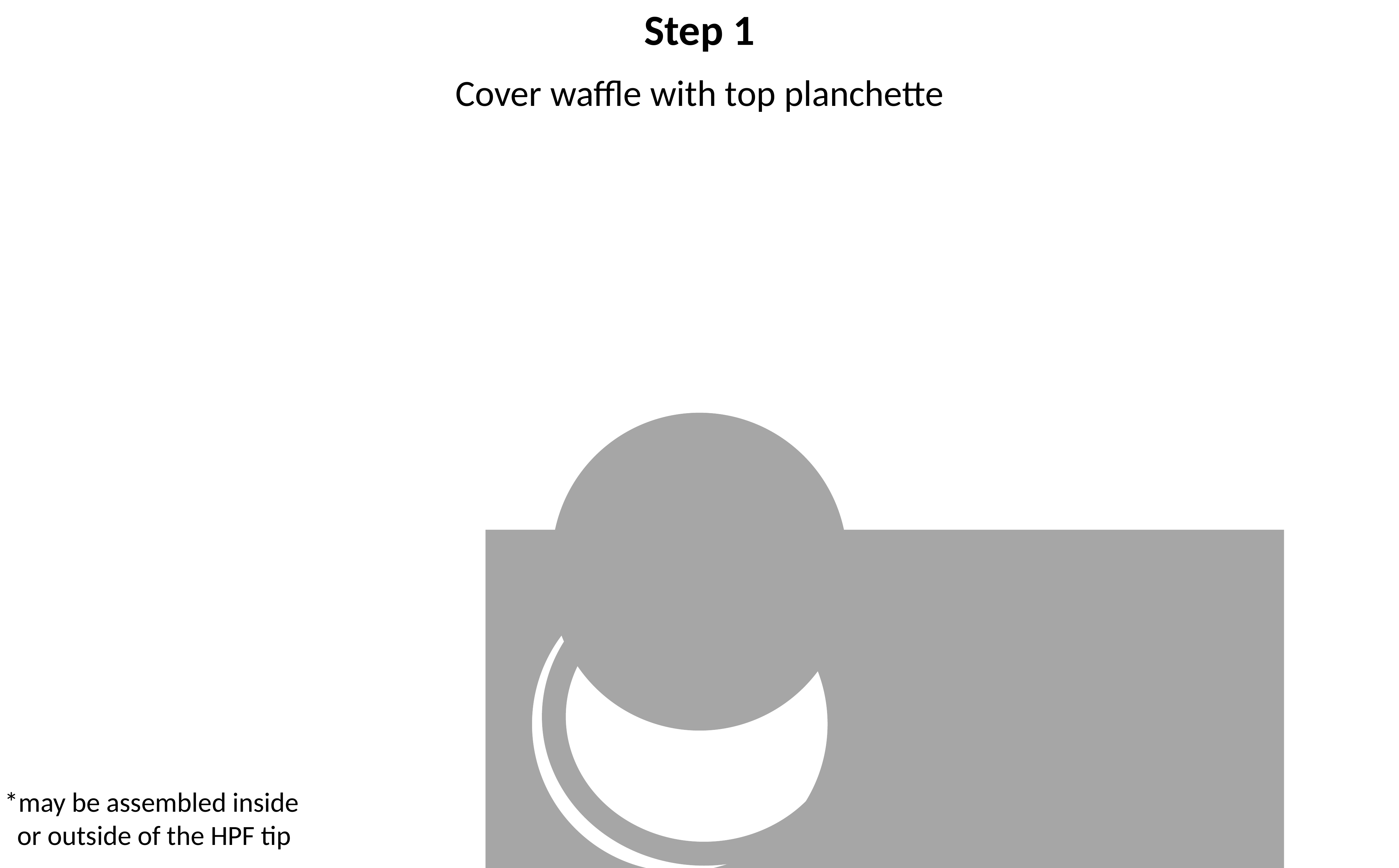

Step 1
Cover waffle with top planchette
*may be assembled inside
 or outside of the HPF tip

## Slide 42
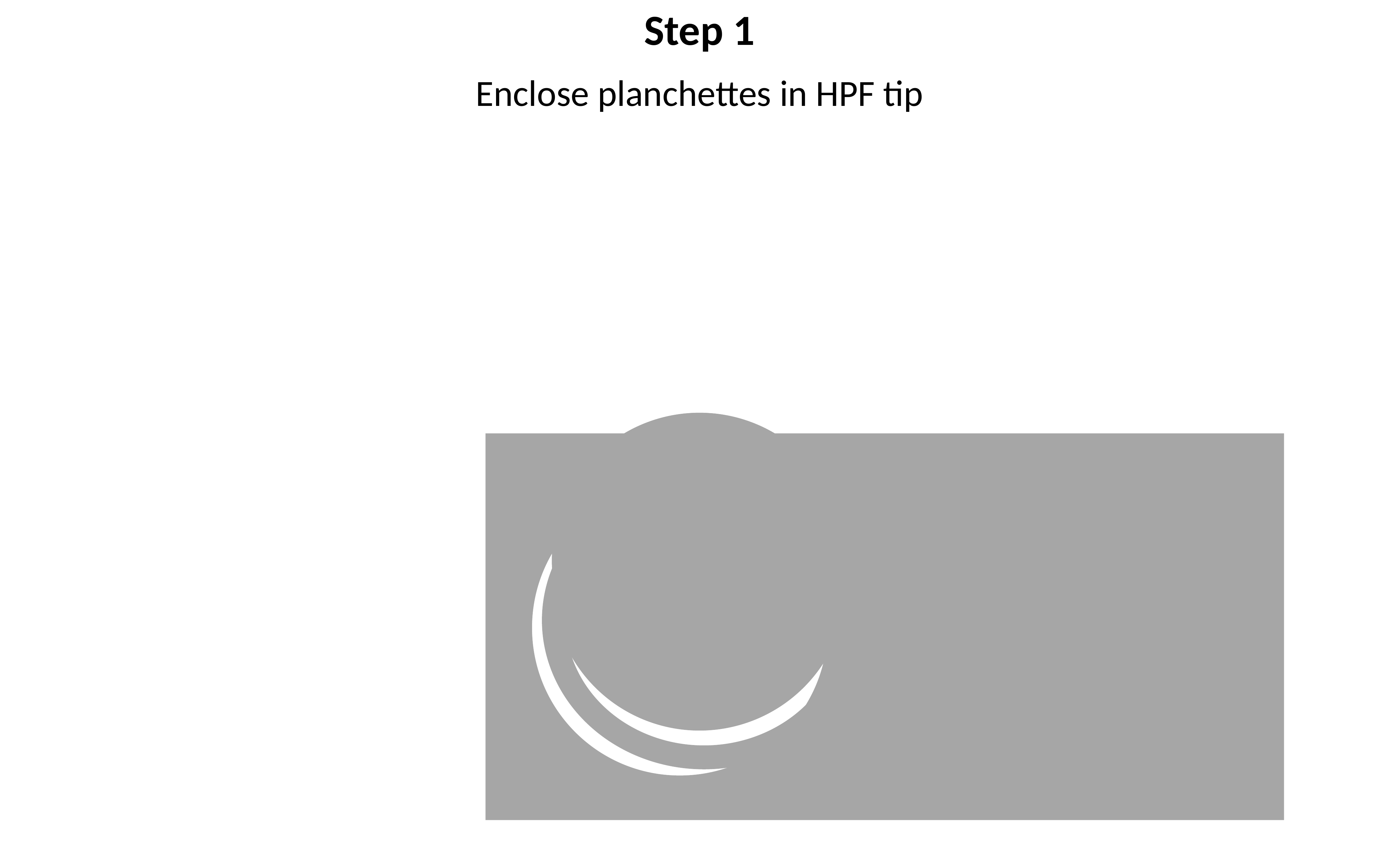

Step 1
Enclose planchettes in HPF tip

## Slide 43
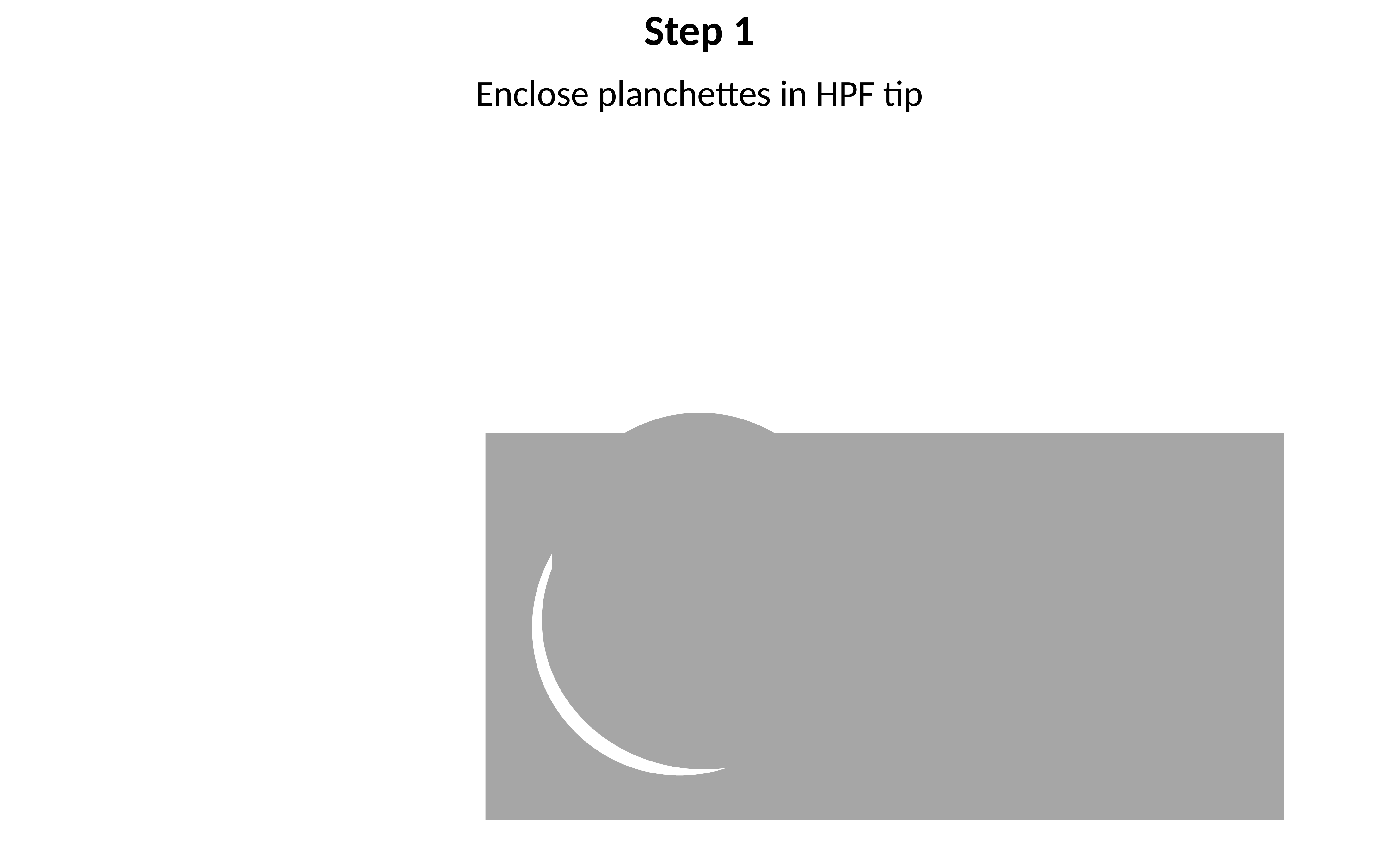

Step 1
Enclose planchettes in HPF tip

## Slide 44
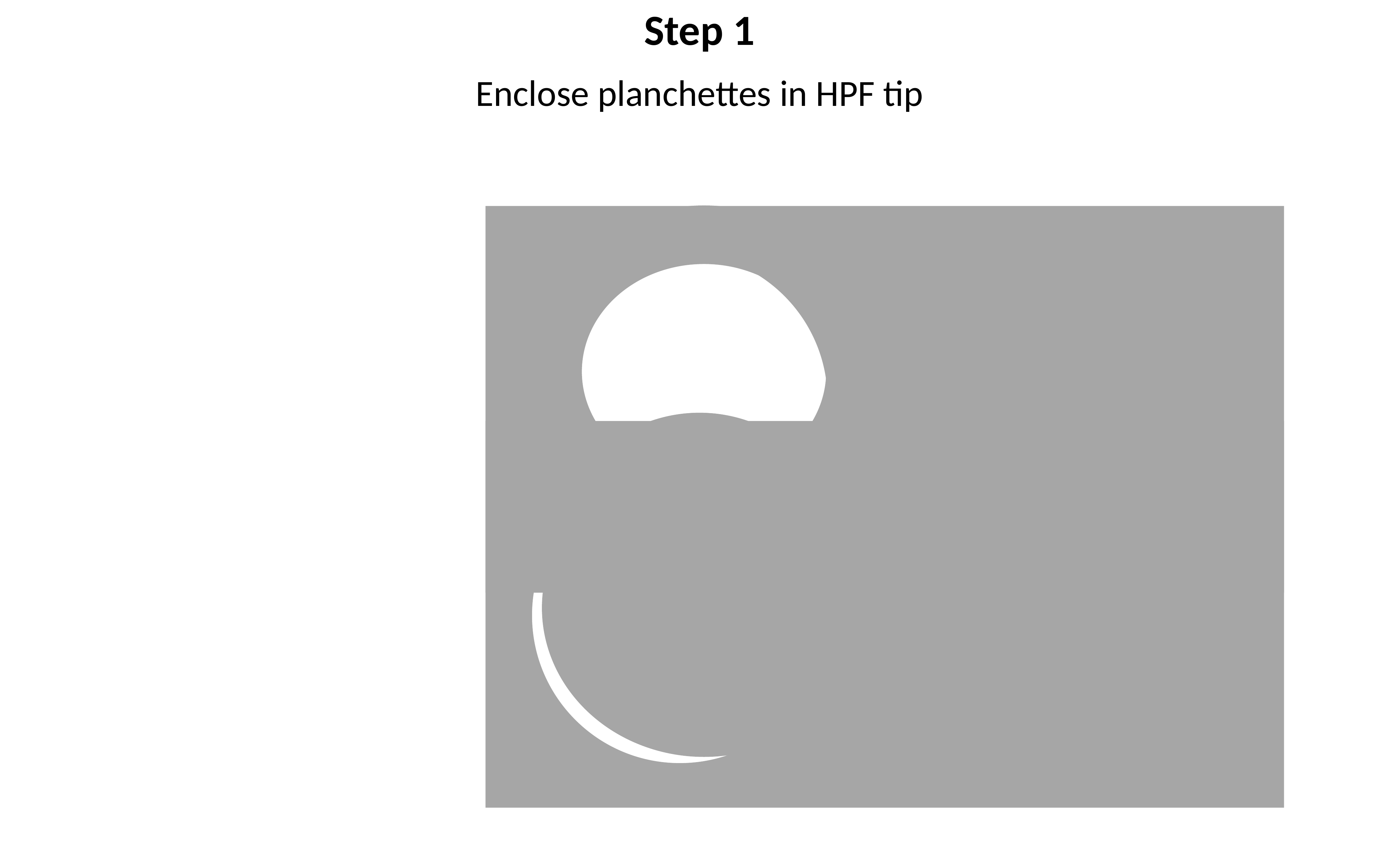

Step 1
Enclose planchettes in HPF tip

## Slide 45
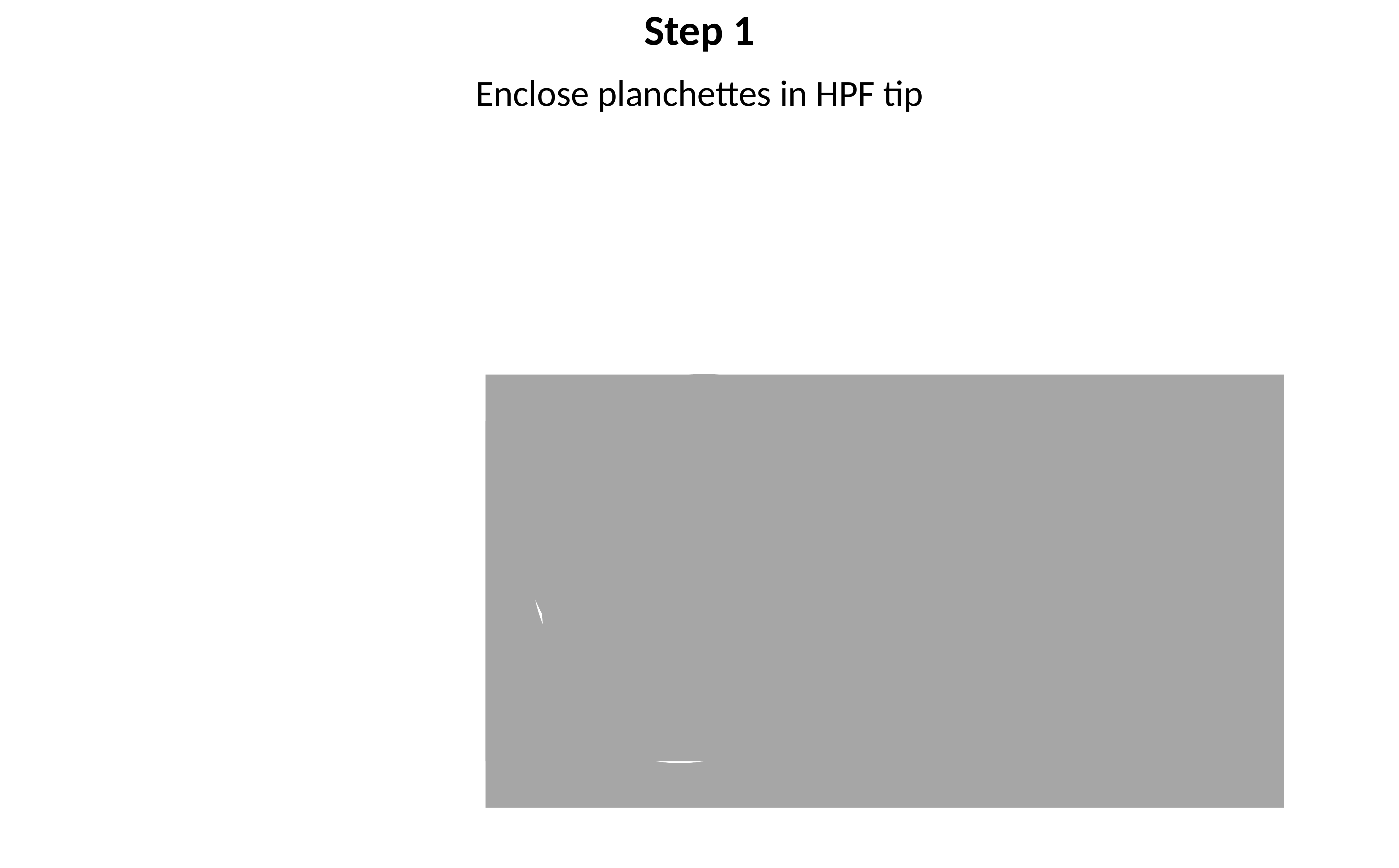

Step 1
Enclose planchettes in HPF tip

## Slide 46
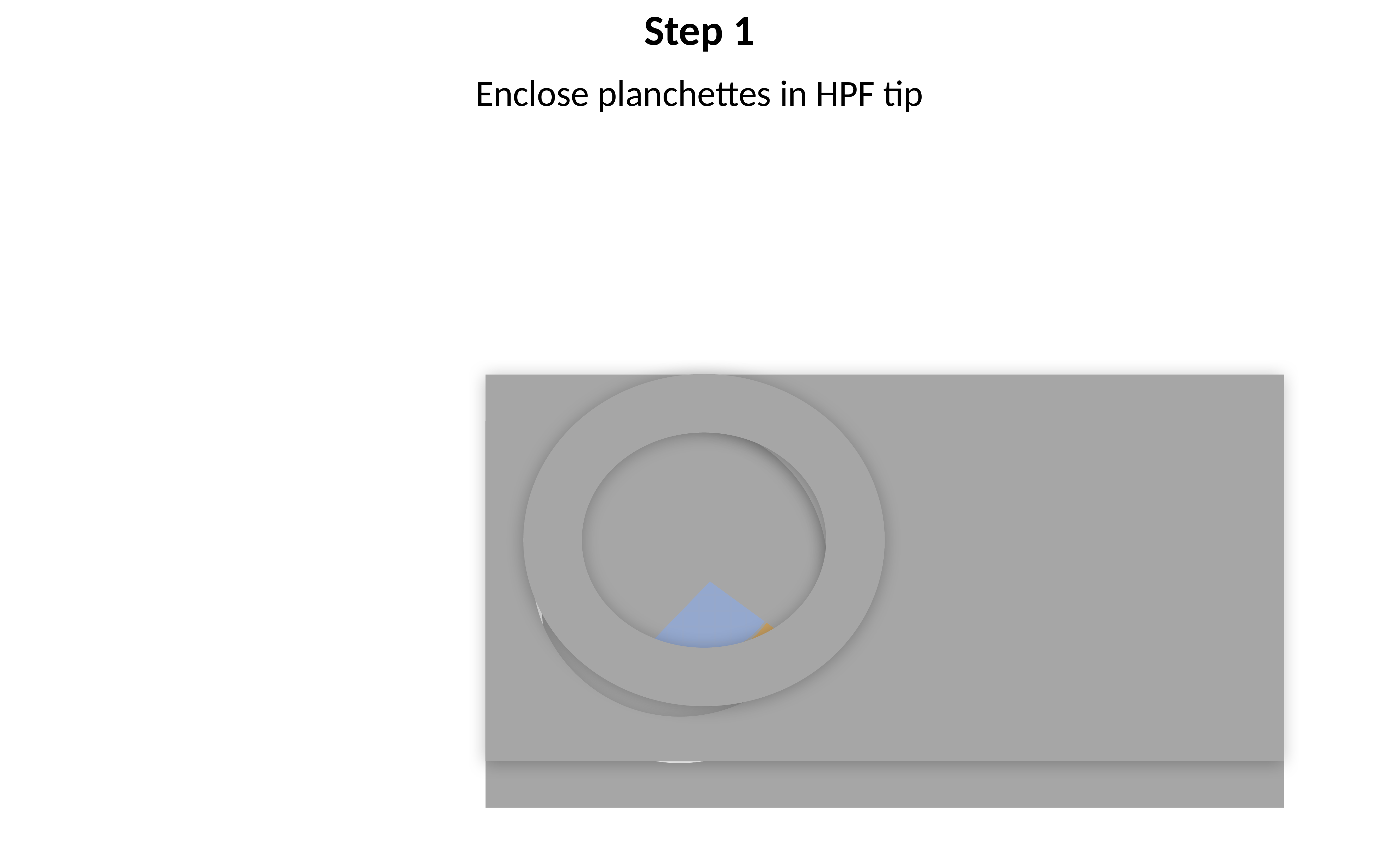

Step 1
Enclose planchettes in HPF tip

## Slide 47
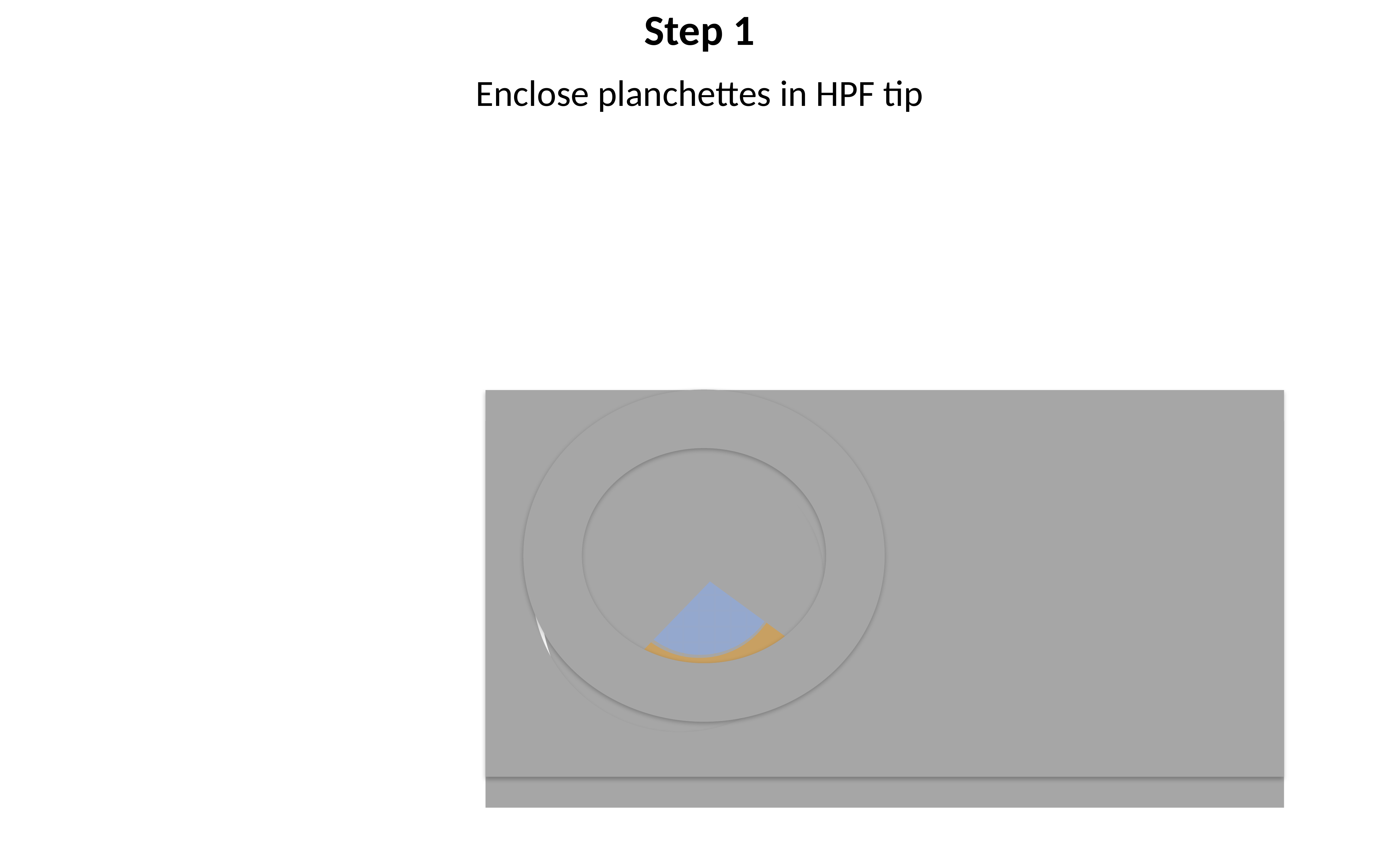

Step 1
Enclose planchettes in HPF tip

## Slide 48
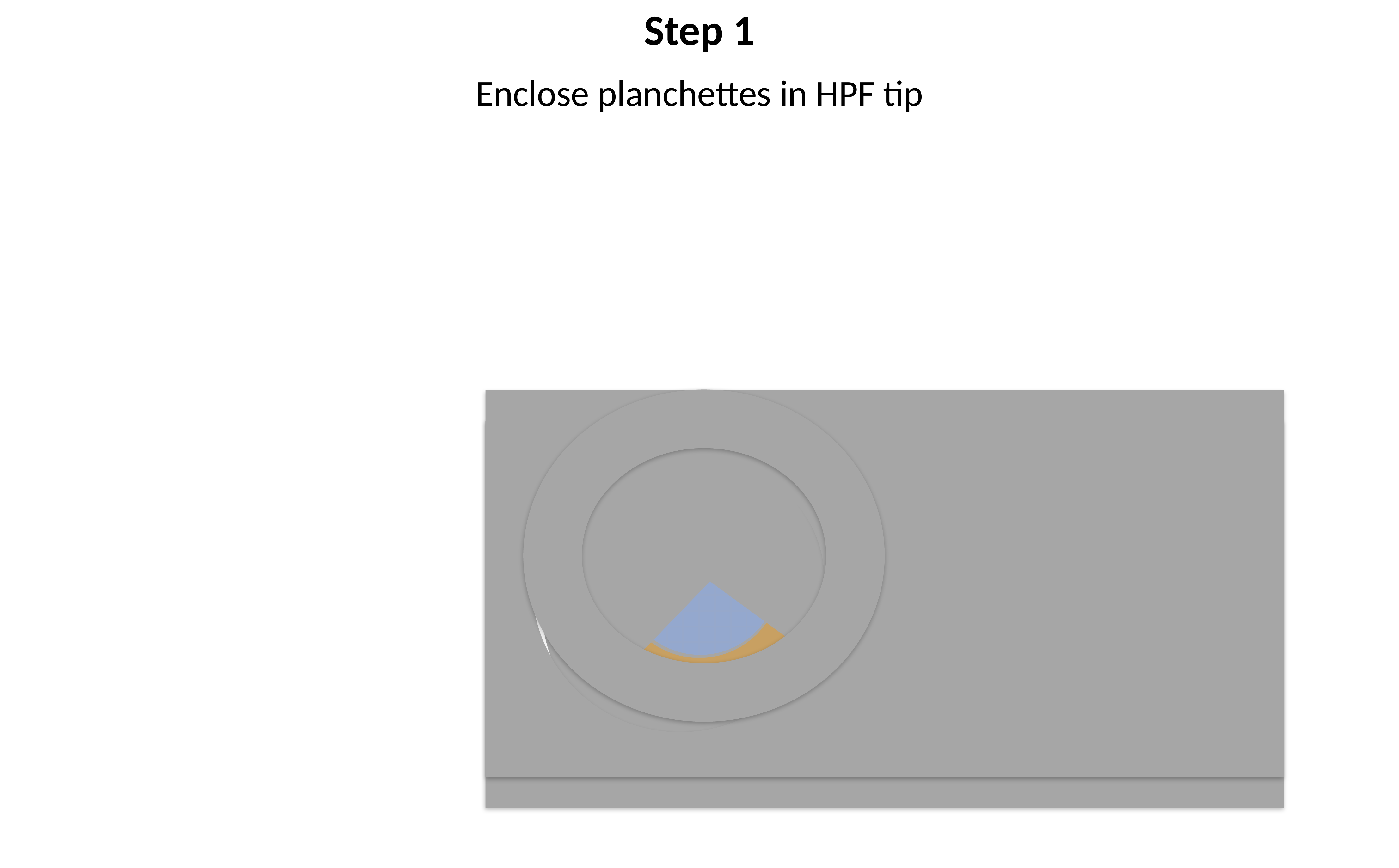

Step 1
Enclose planchettes in HPF tip

## Slide 49
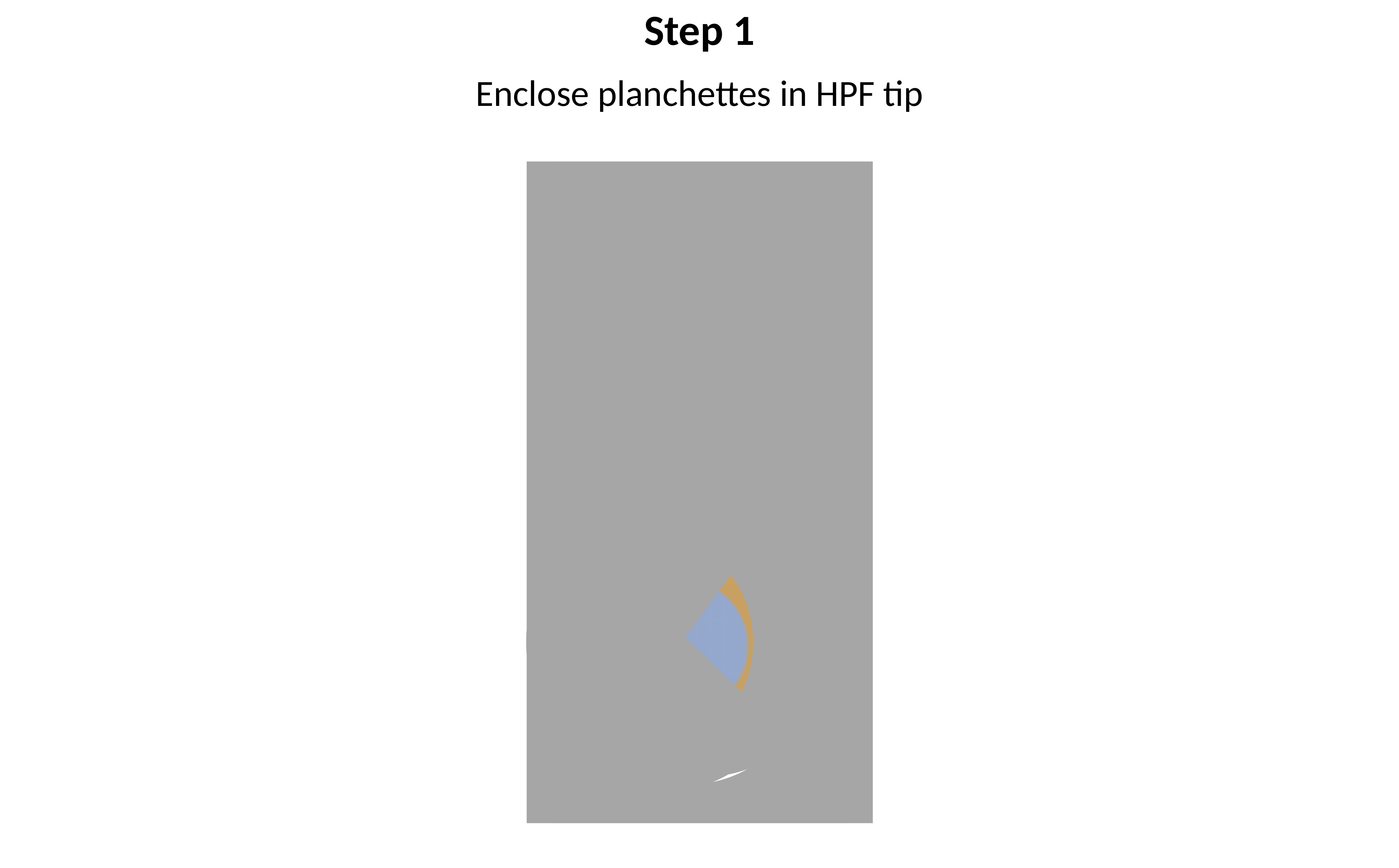

Step 1
Enclose planchettes in HPF tip

## Slide 50
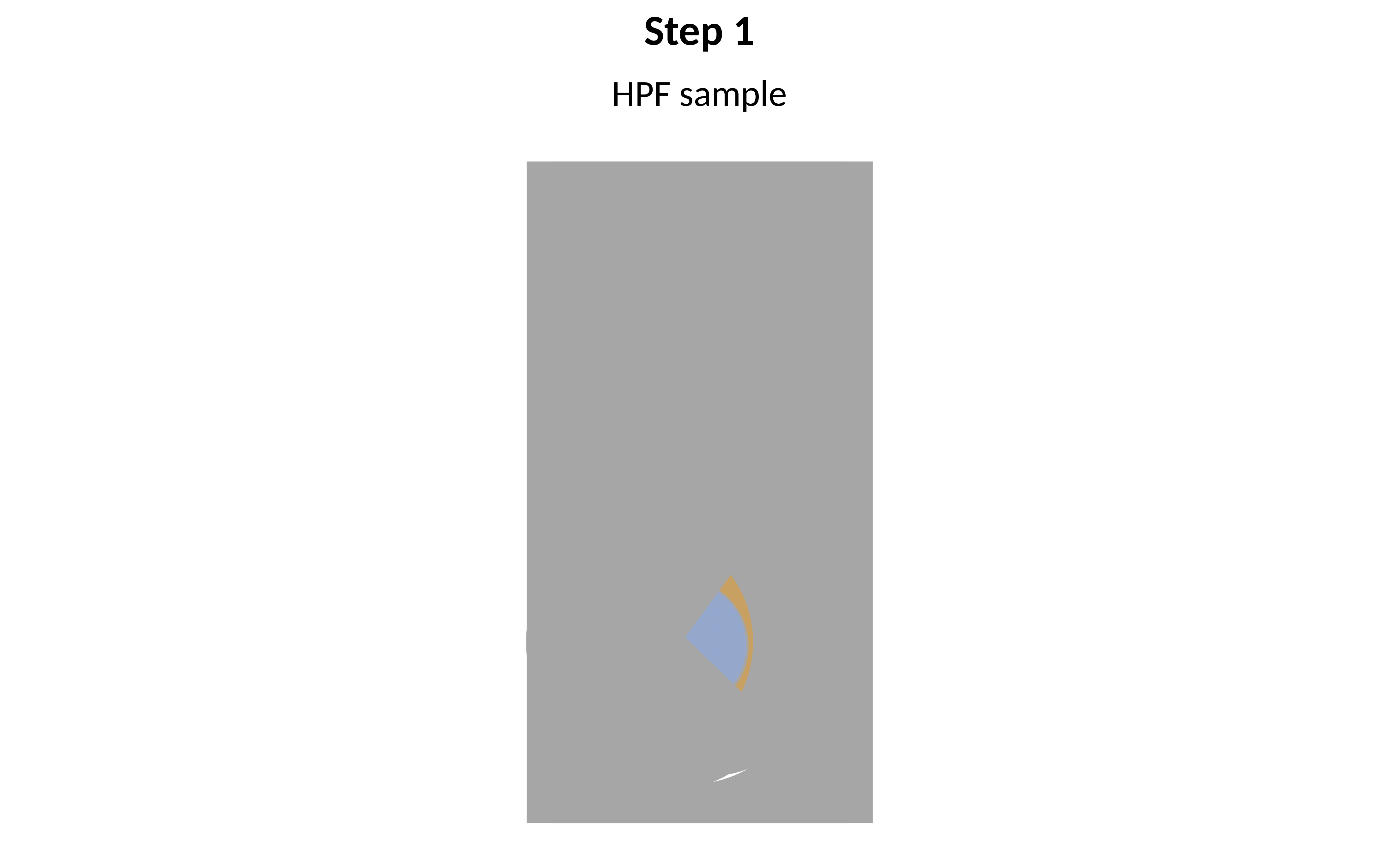

Step 1
HPF sample

## Slide 51
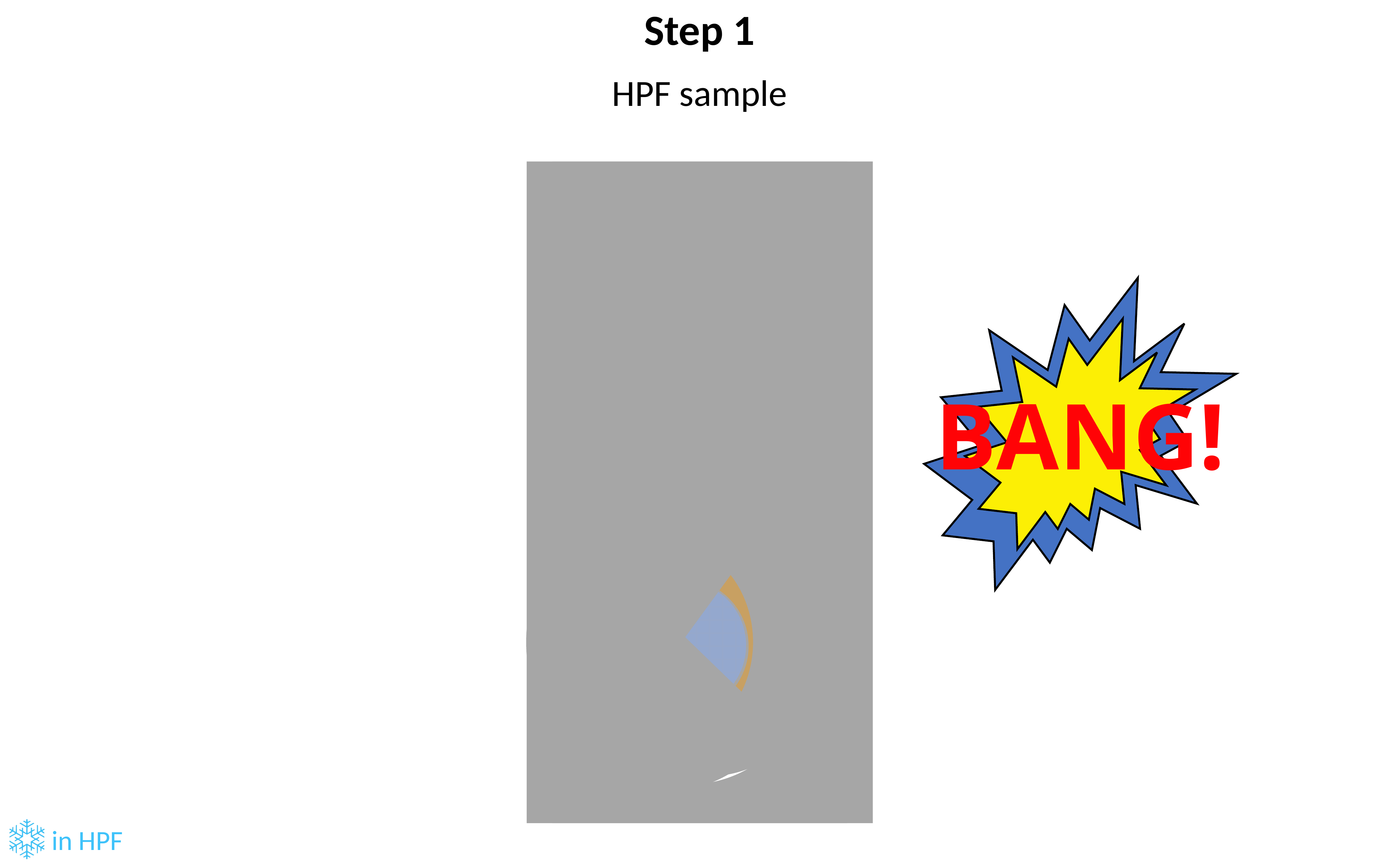

Step 1
HPF sample
BANG!
in HPF

## Slide 52
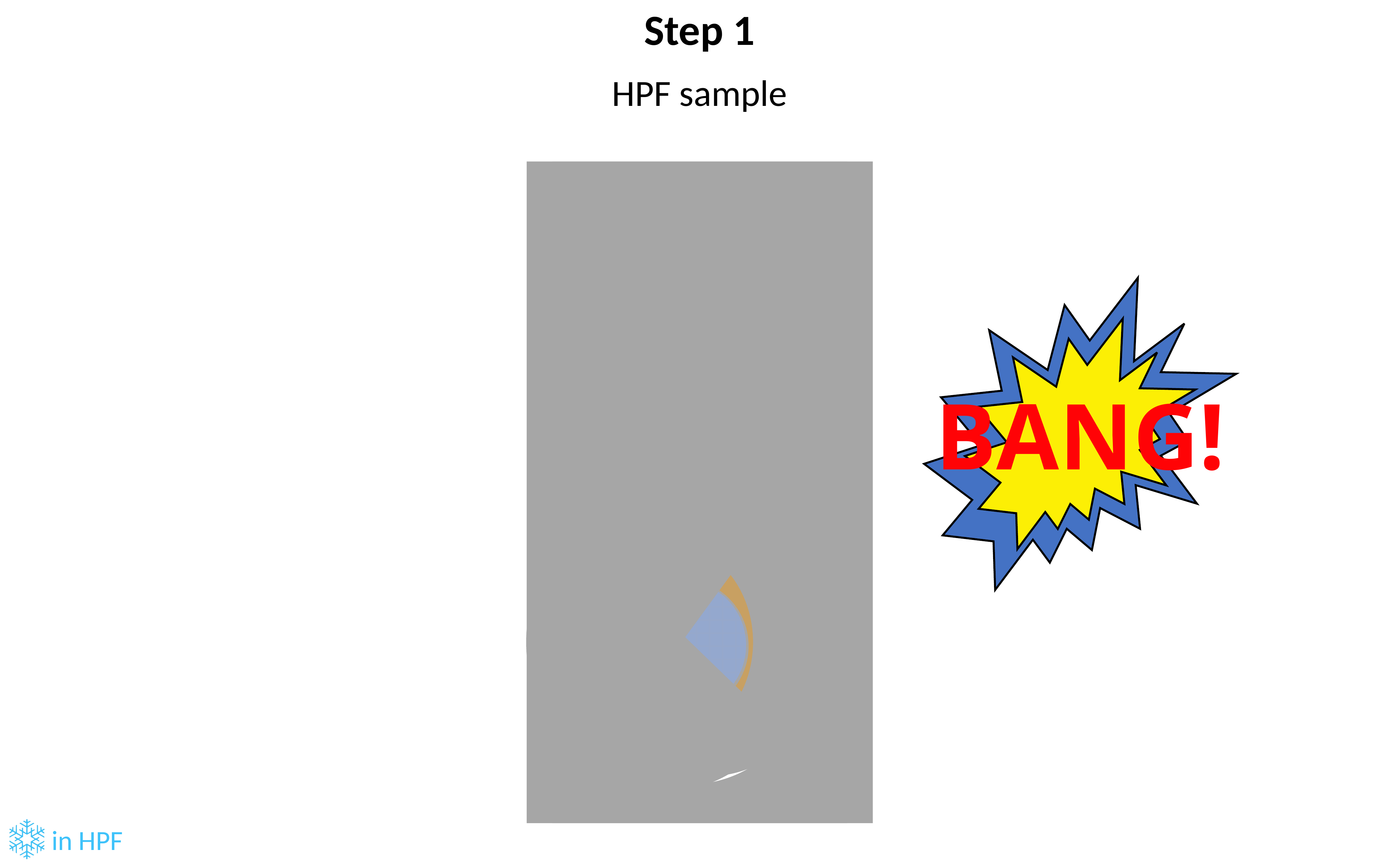

Step 1
HPF sample
BANG!
in HPF

## Slide 53
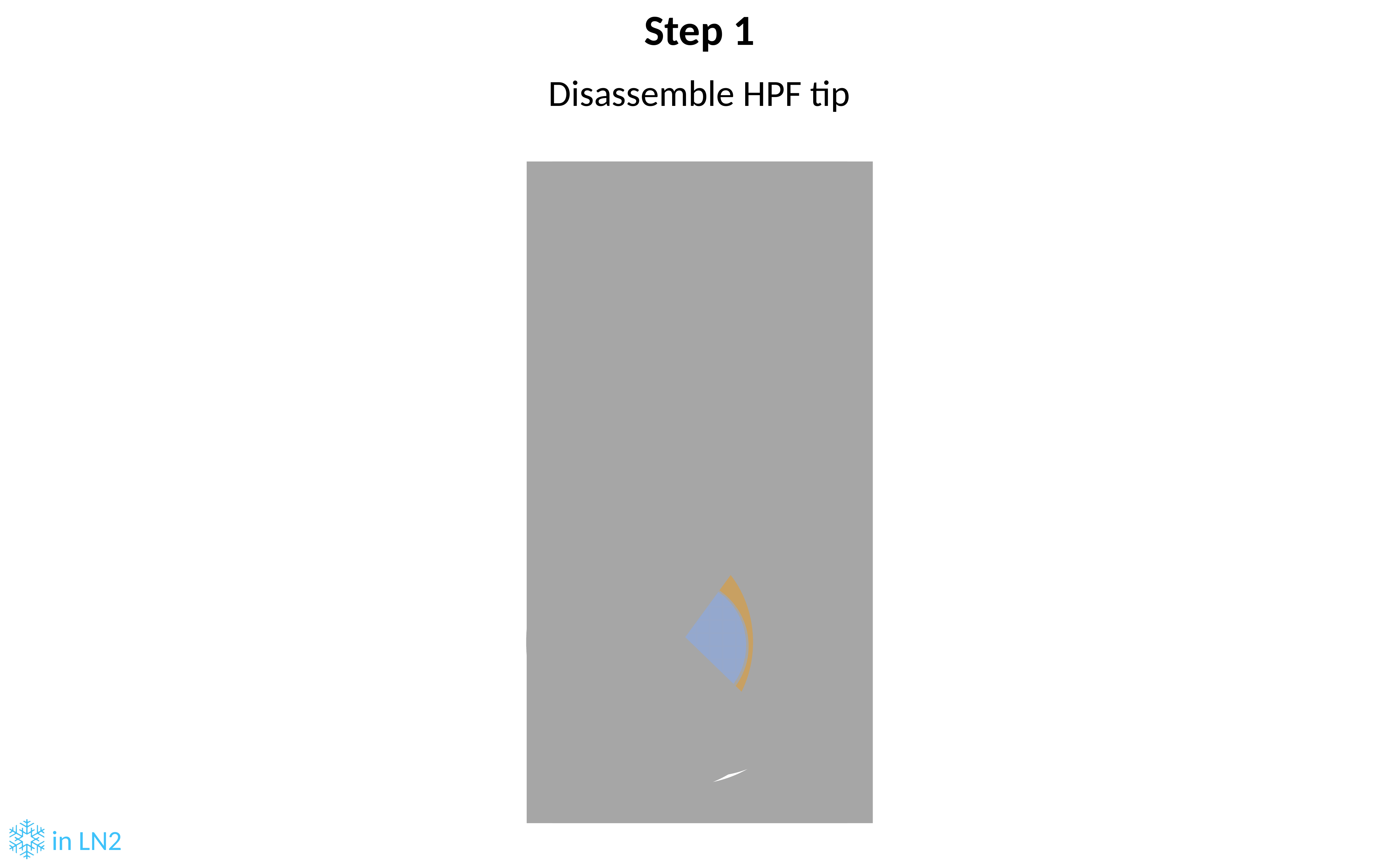

Step 1
Disassemble HPF tip
in LN2

## Slide 54
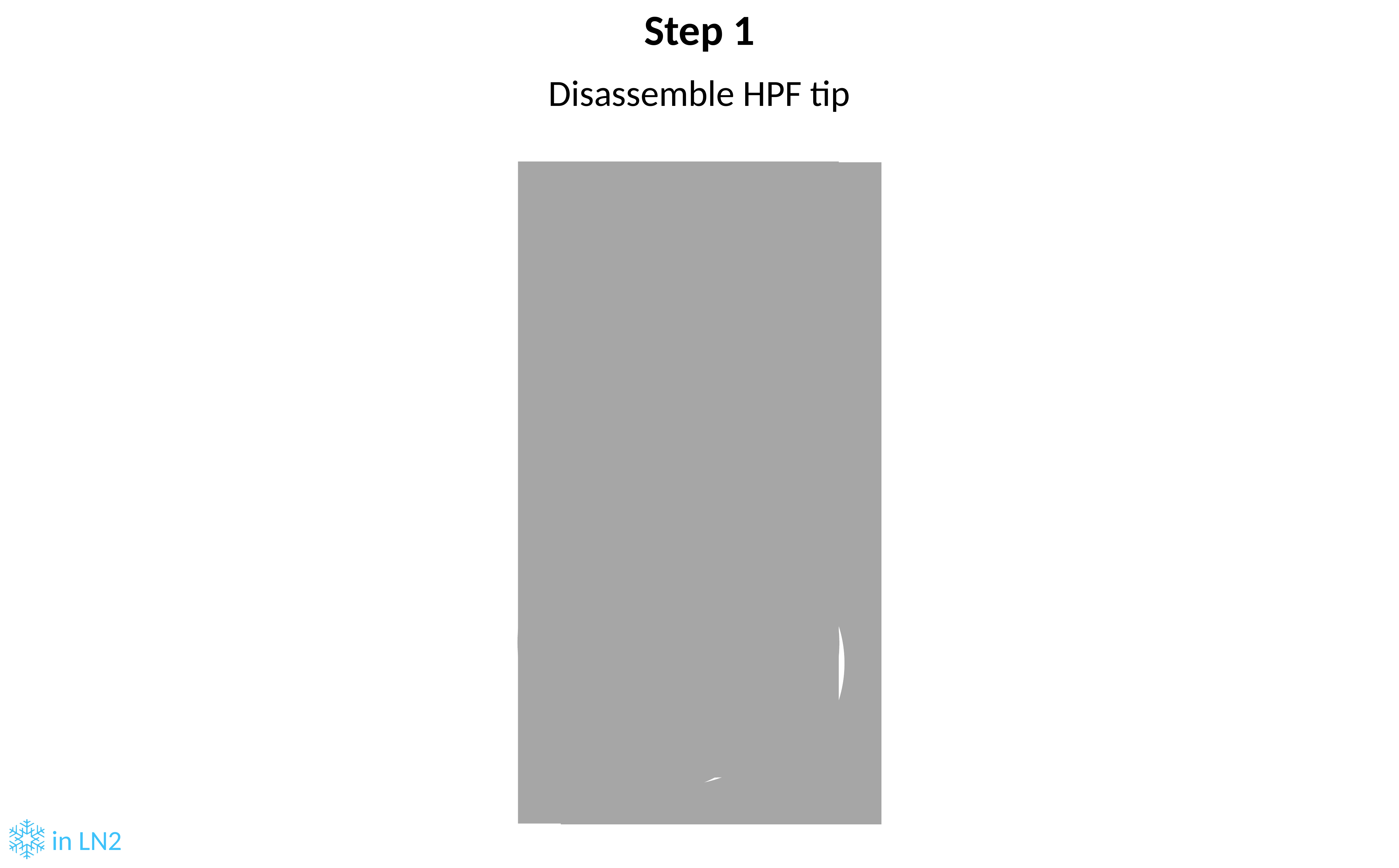

Step 1
Disassemble HPF tip
in LN2

## Slide 55
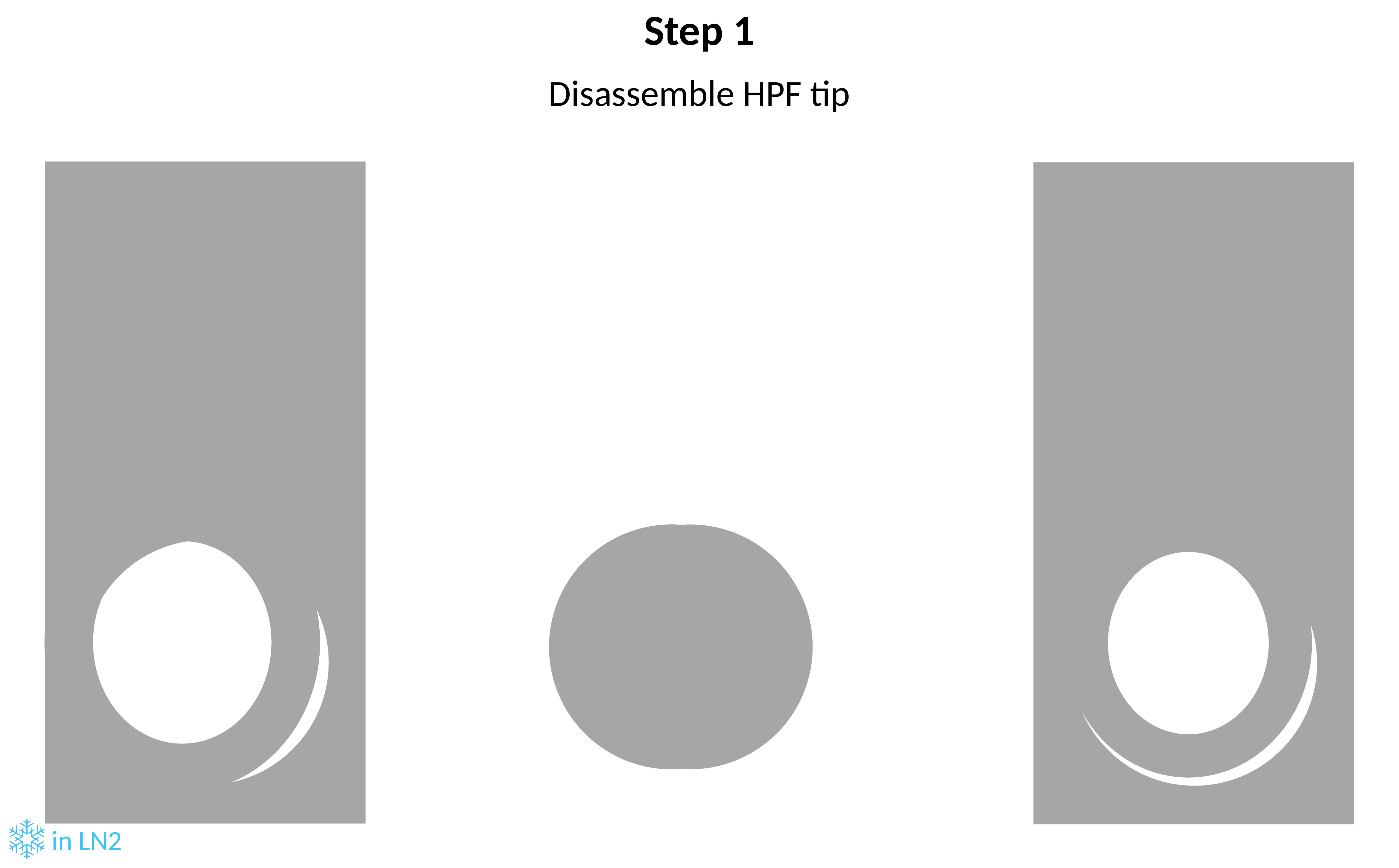

Step 1
Disassemble HPF tip
in LN2

## Slide 56
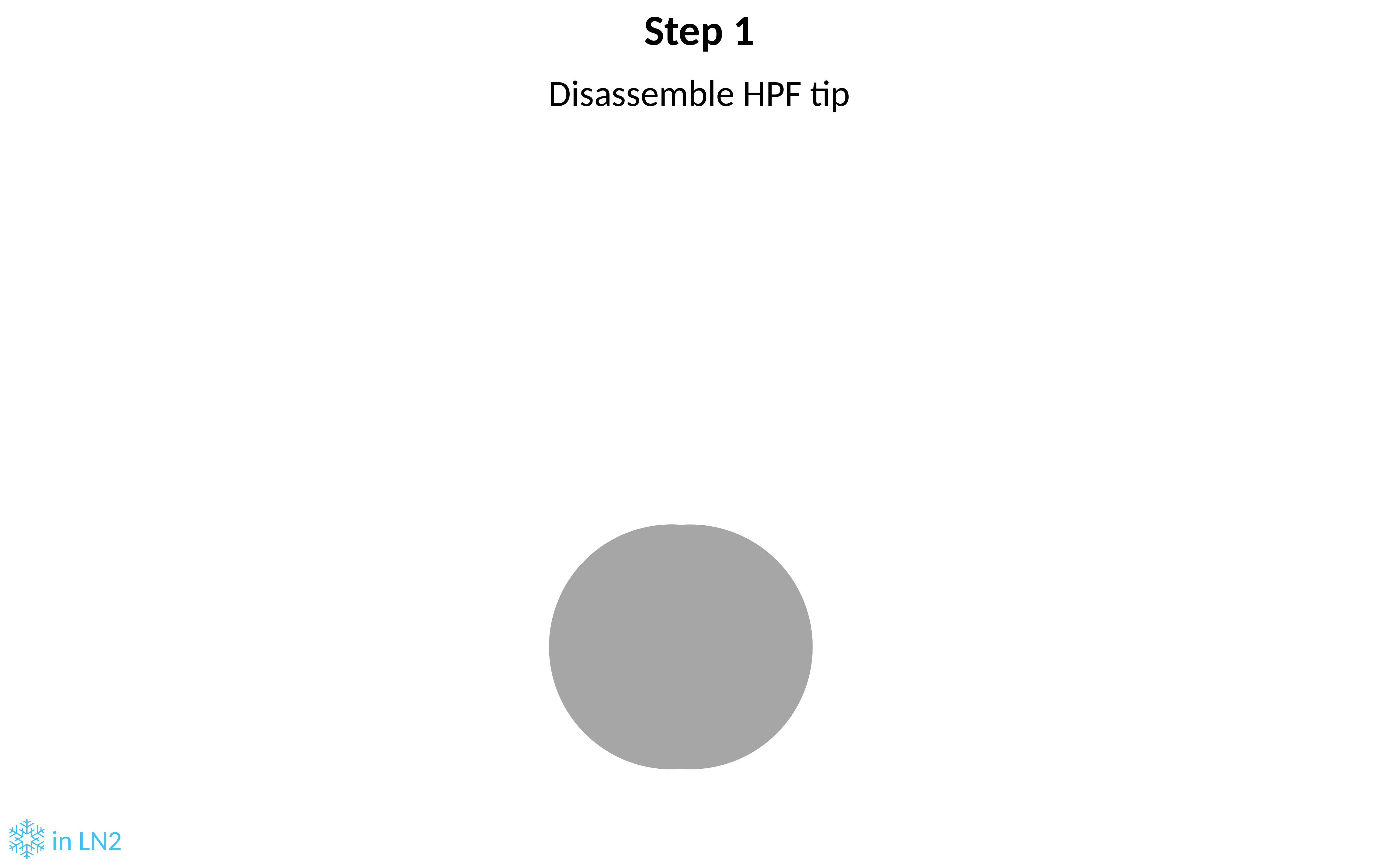

Step 1
Disassemble HPF tip
in LN2

## Slide 57
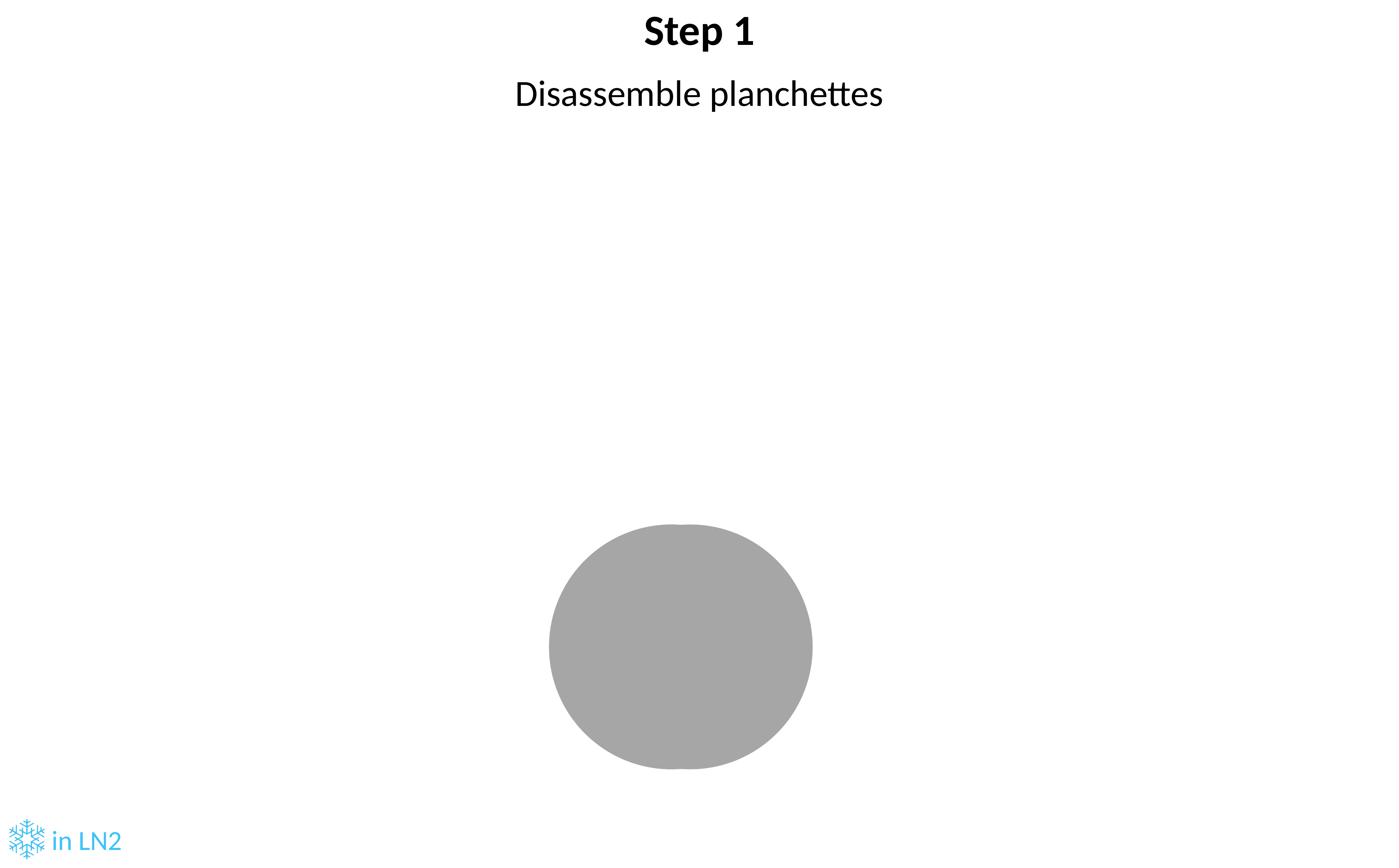

Step 1
Disassemble planchettes
in LN2

## Slide 58
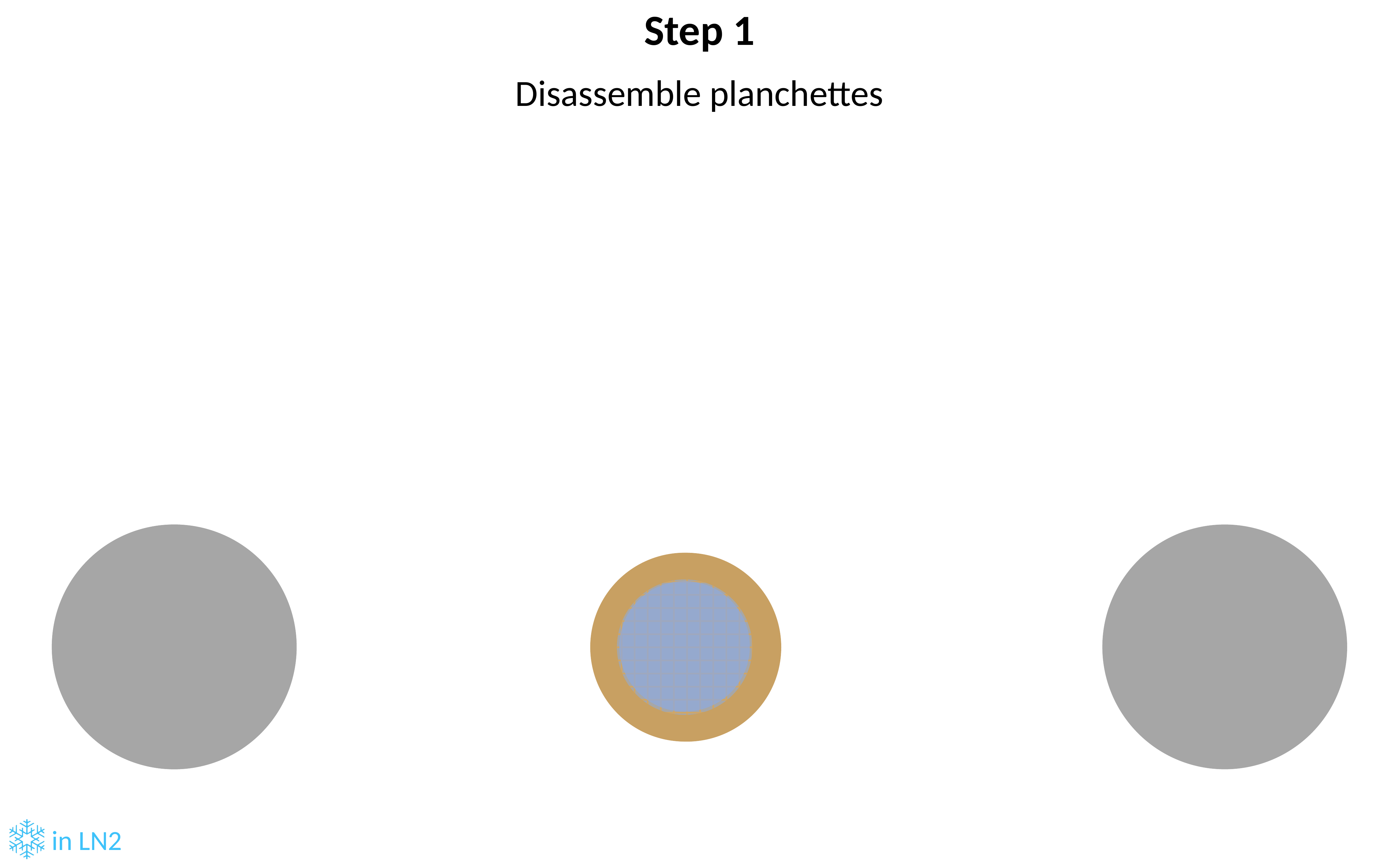

Step 1
Disassemble planchettes
in LN2

## Slide 59
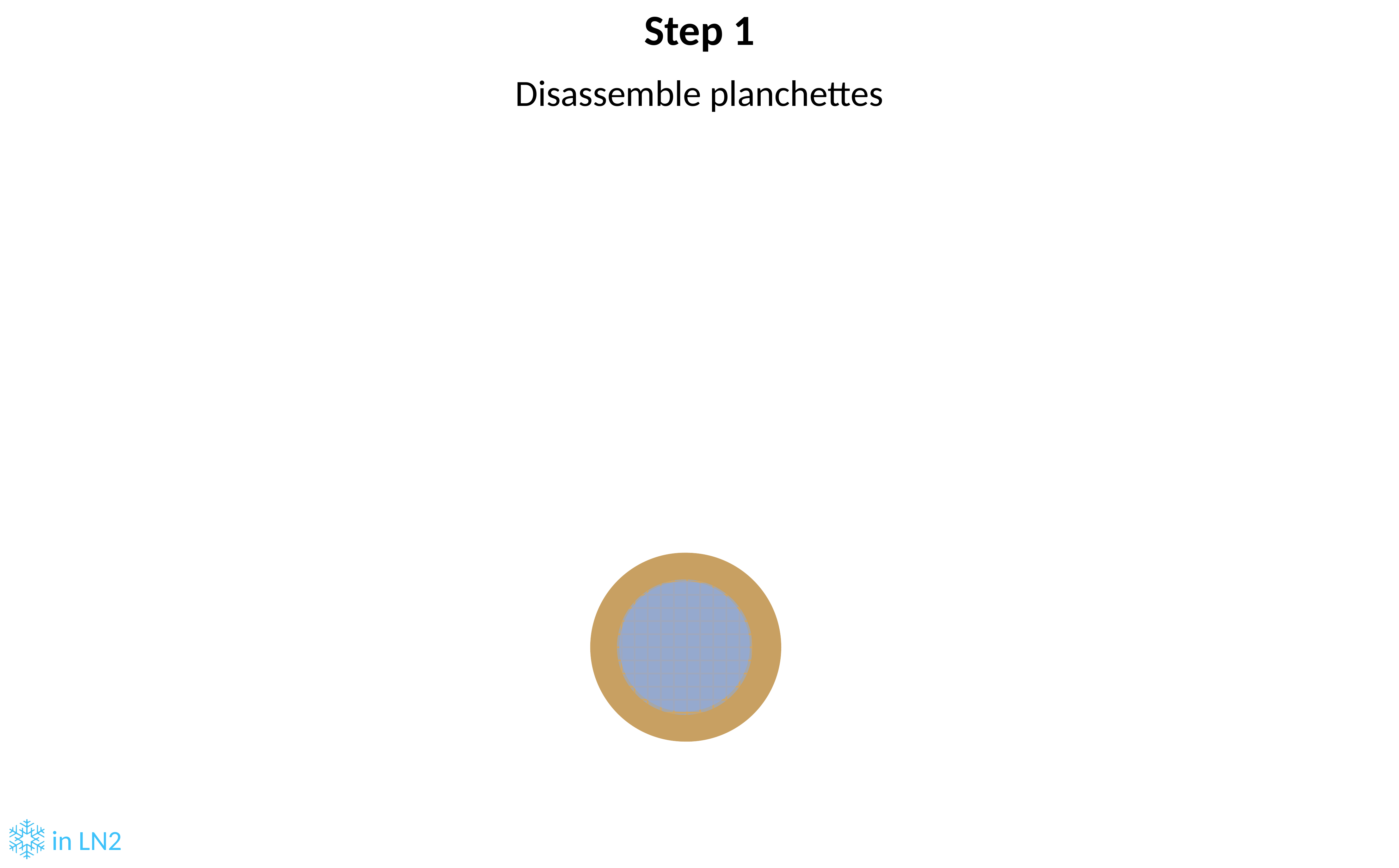

Step 1
Disassemble planchettes
in LN2

## Slide 60
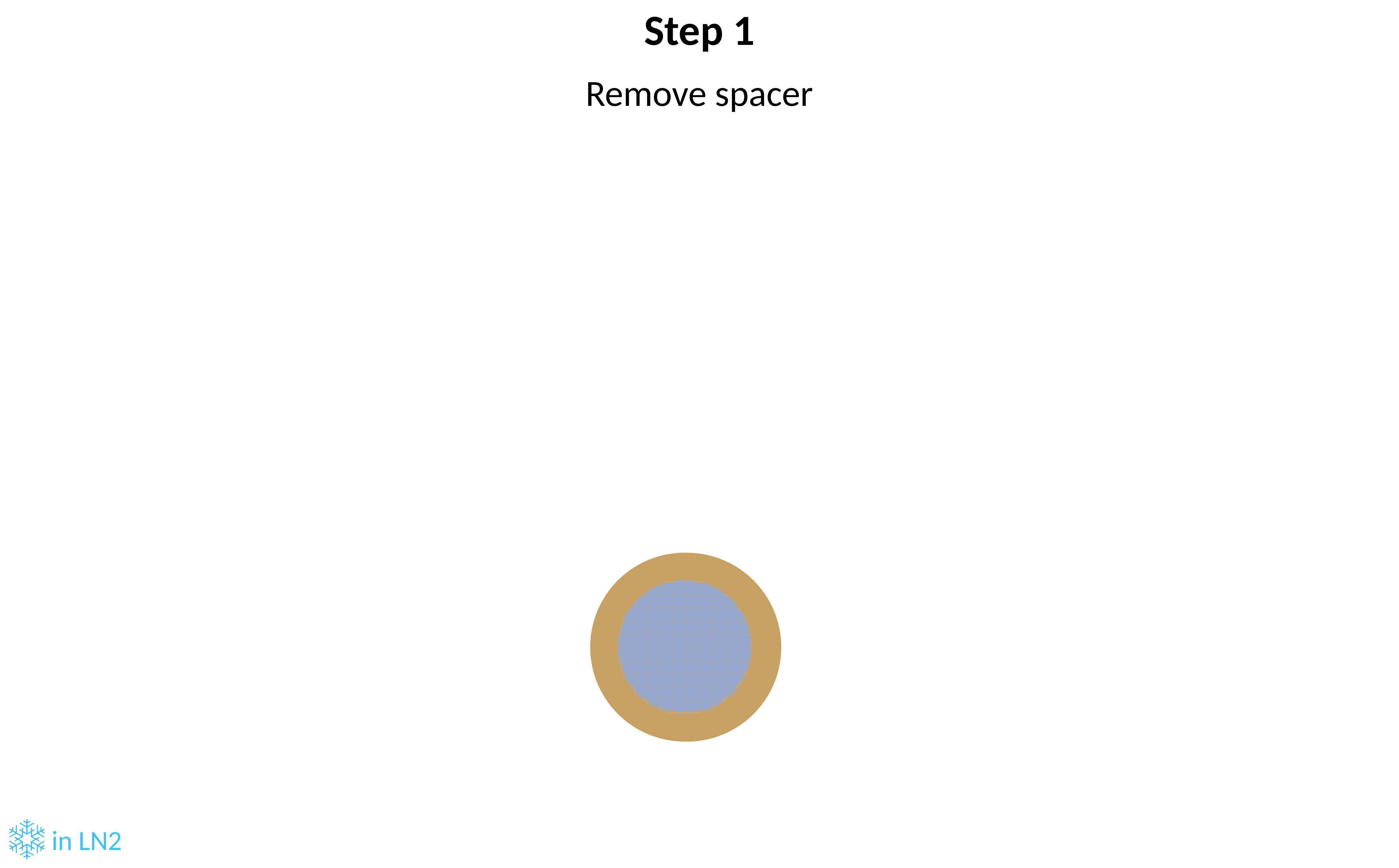

Step 1
Remove spacer
in LN2

## Slide 61
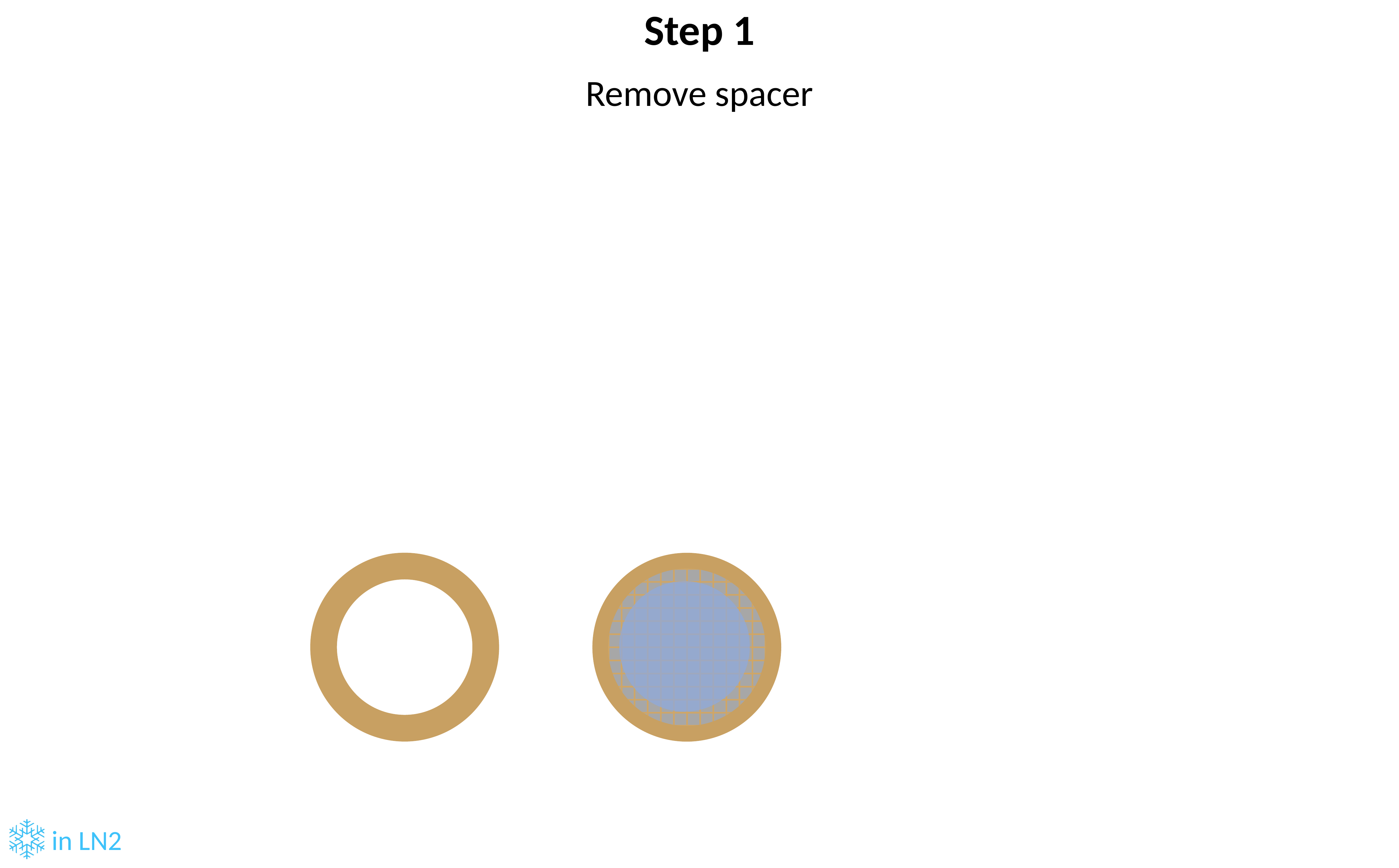

Step 1
Remove spacer
in LN2

## Slide 62
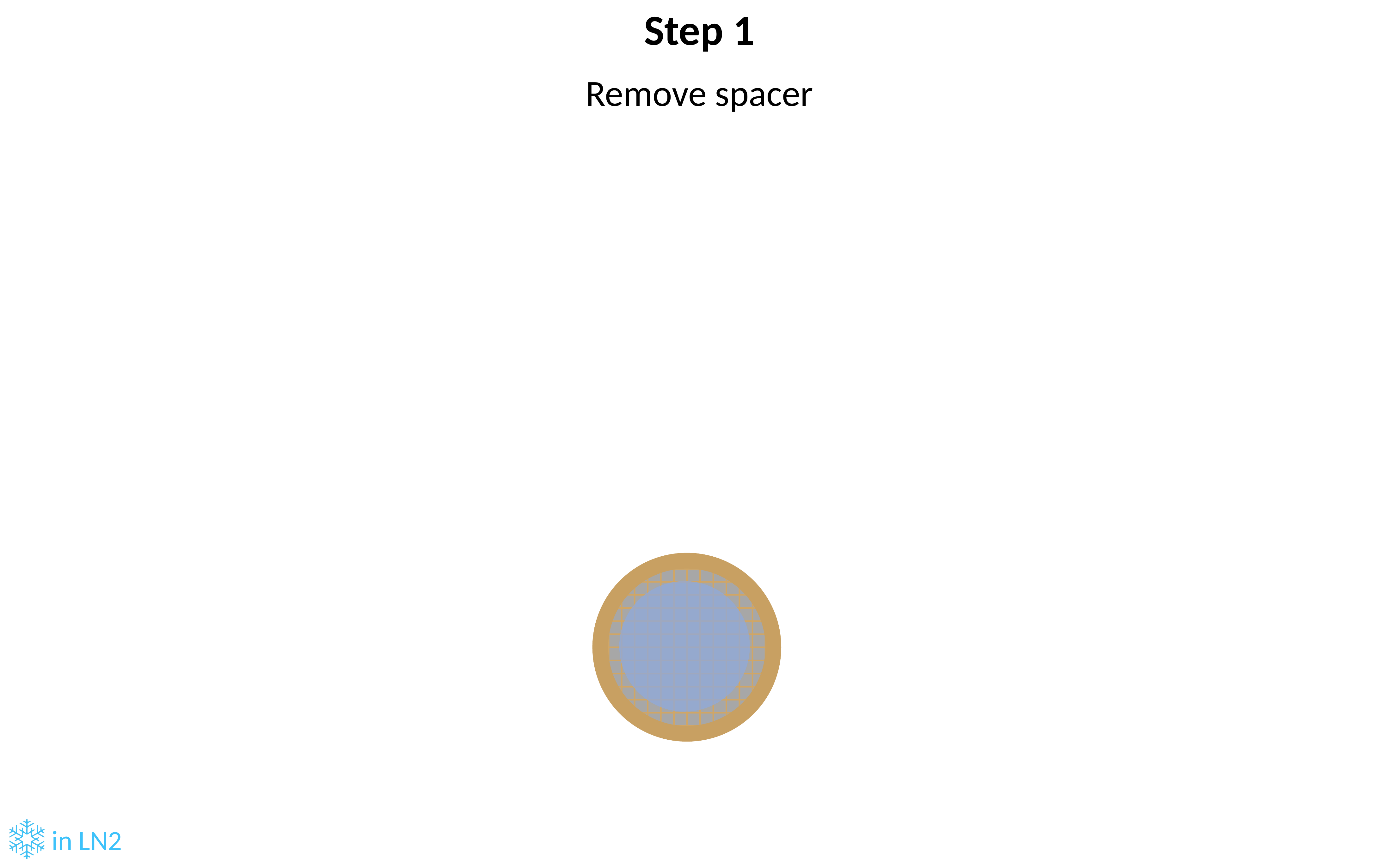

Step 1
Remove spacer
in LN2

## Slide 63
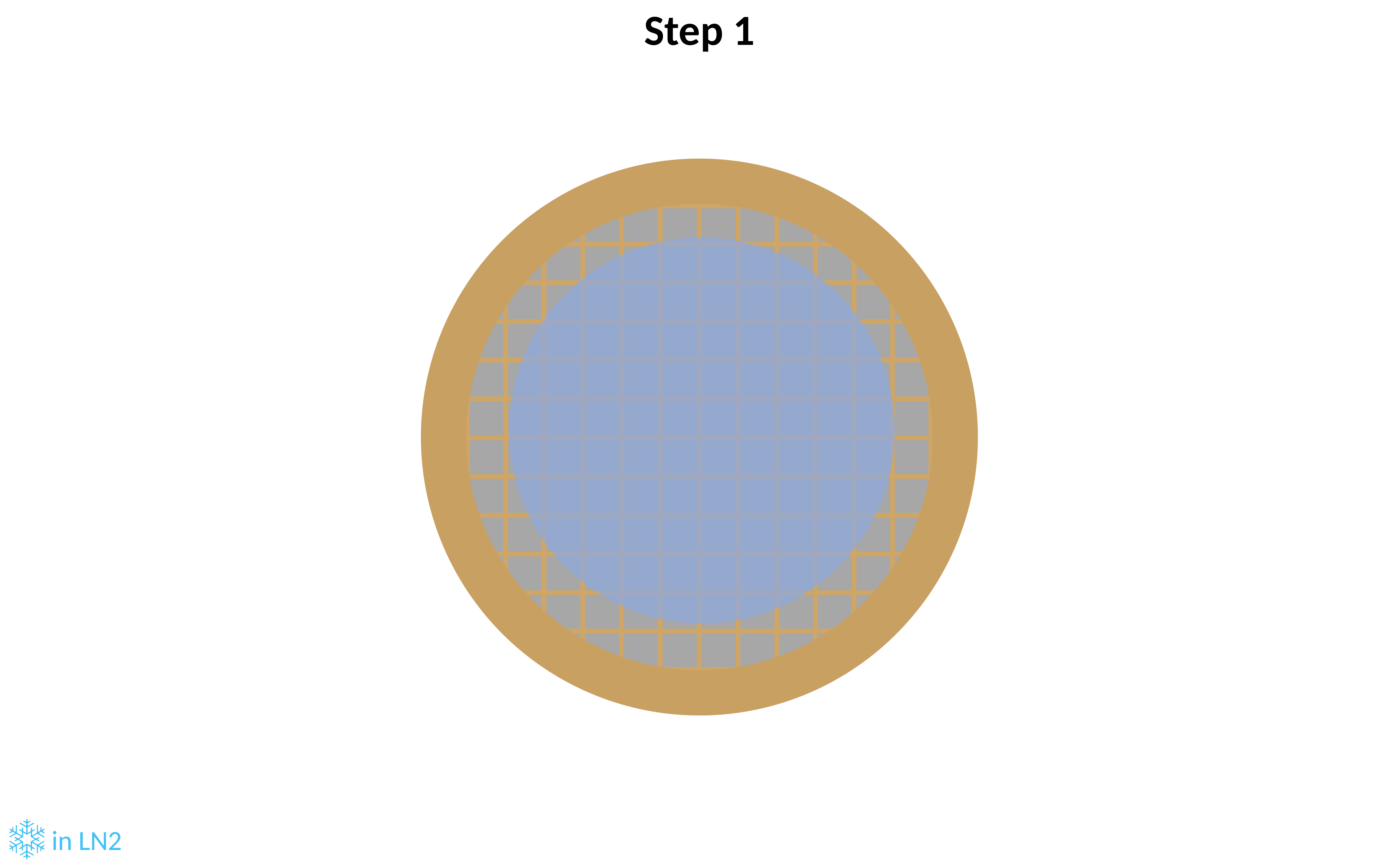

Step 1
in LN2

## Slide 64
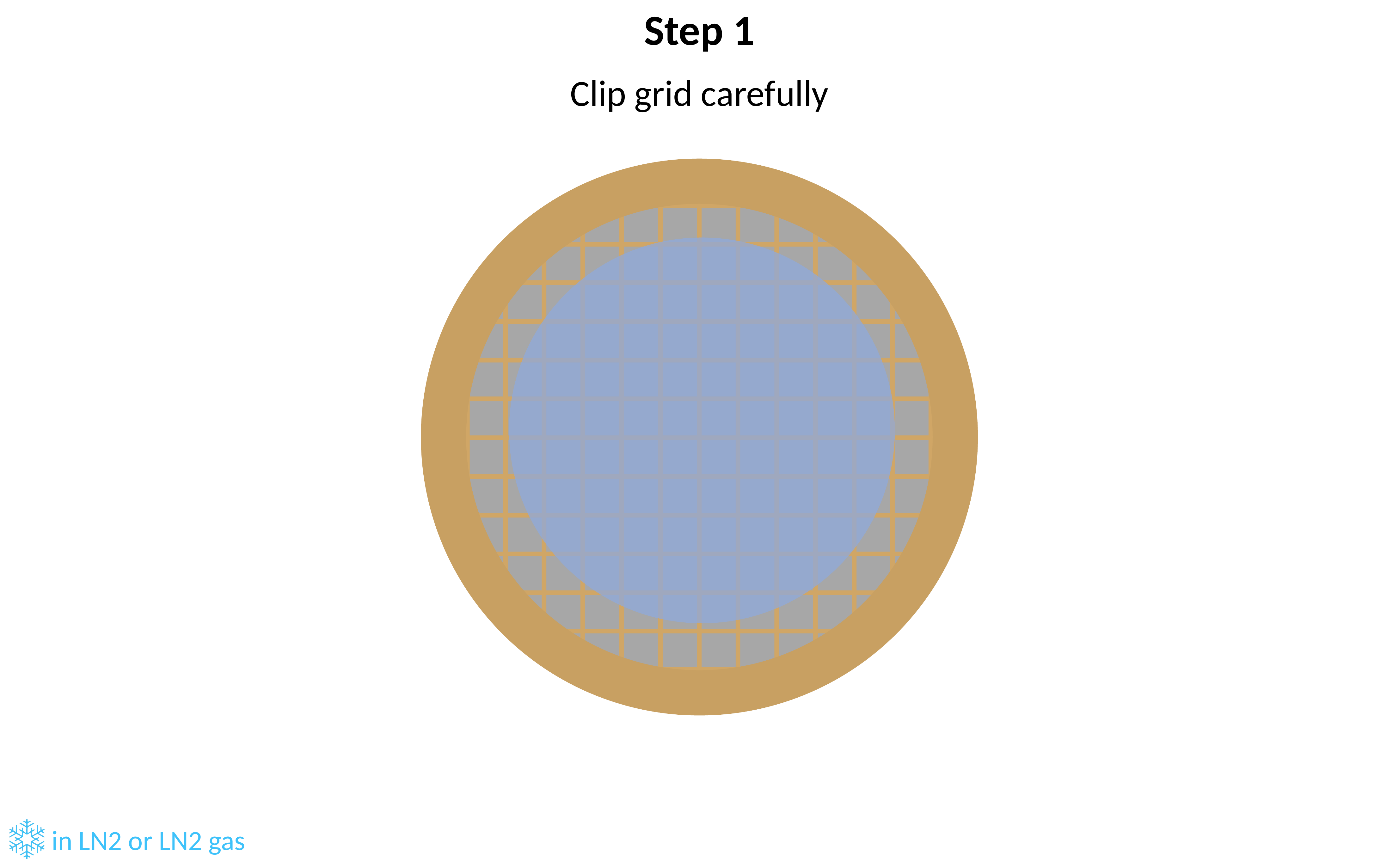

Step 1
Clip grid carefully
in LN2 or LN2 gas

## Slide 65
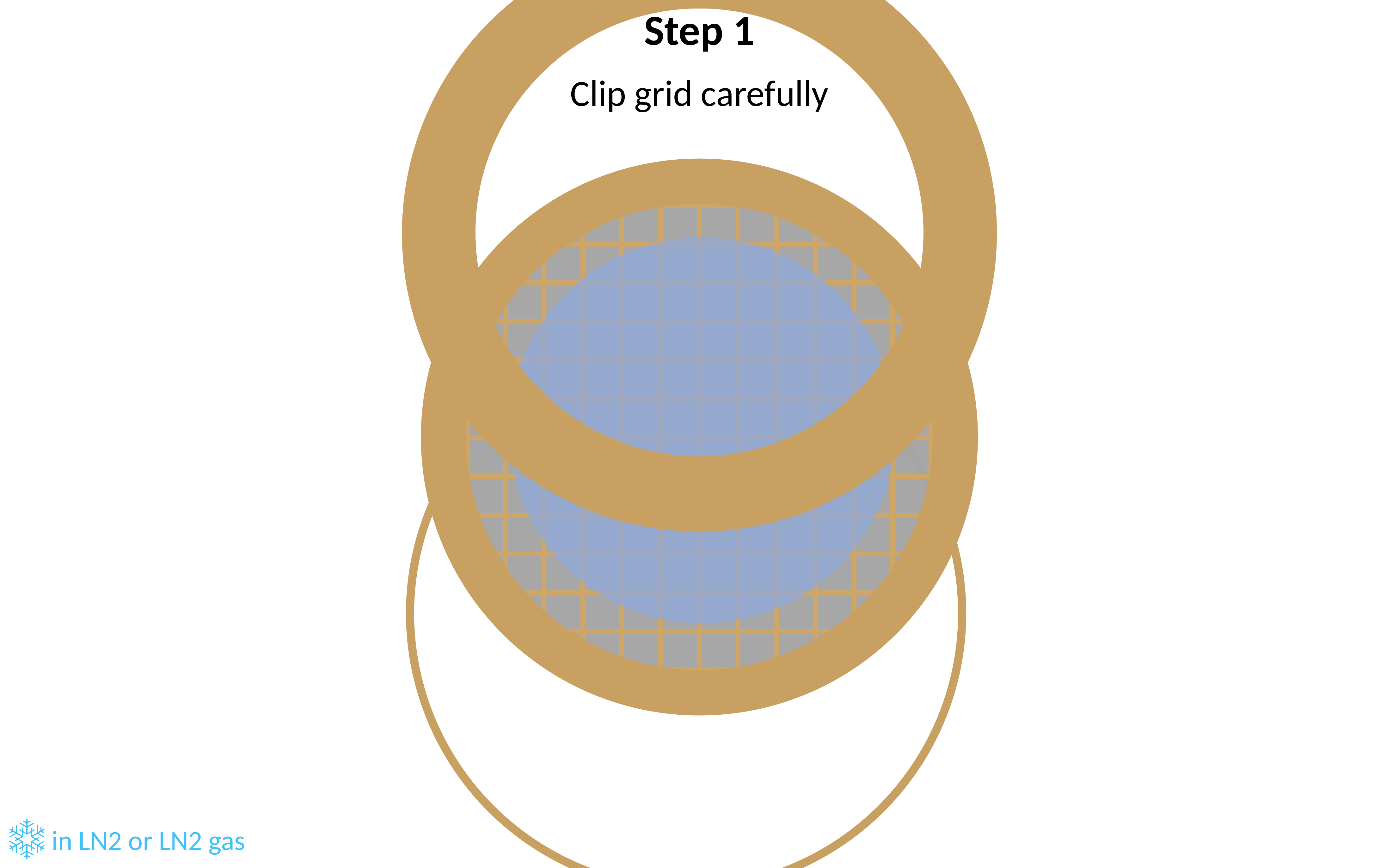

Step 1
Clip grid carefully
in LN2 or LN2 gas

## Slide 66
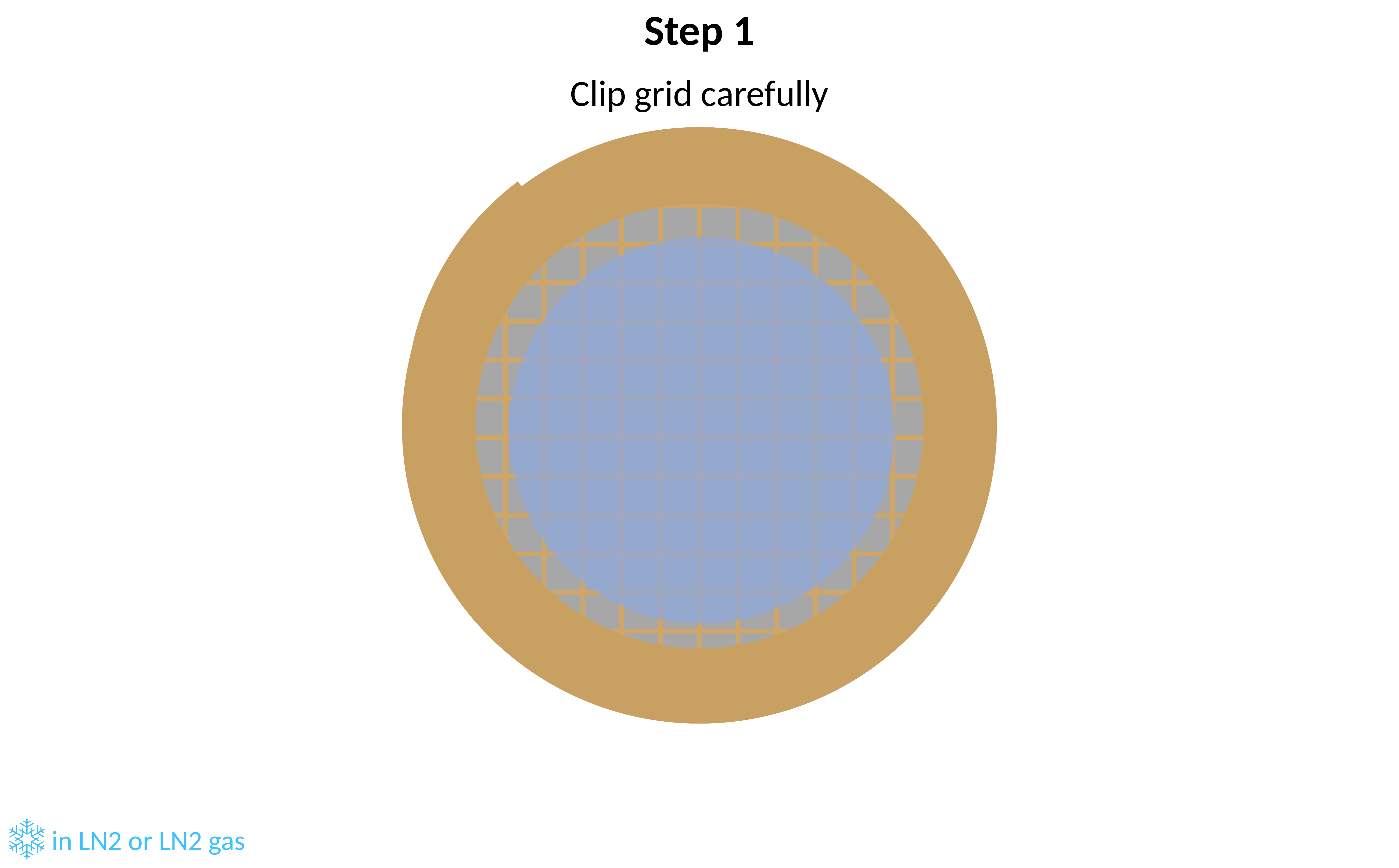

Step 1
Clip grid carefully
in LN2 or LN2 gas

## Slide 67
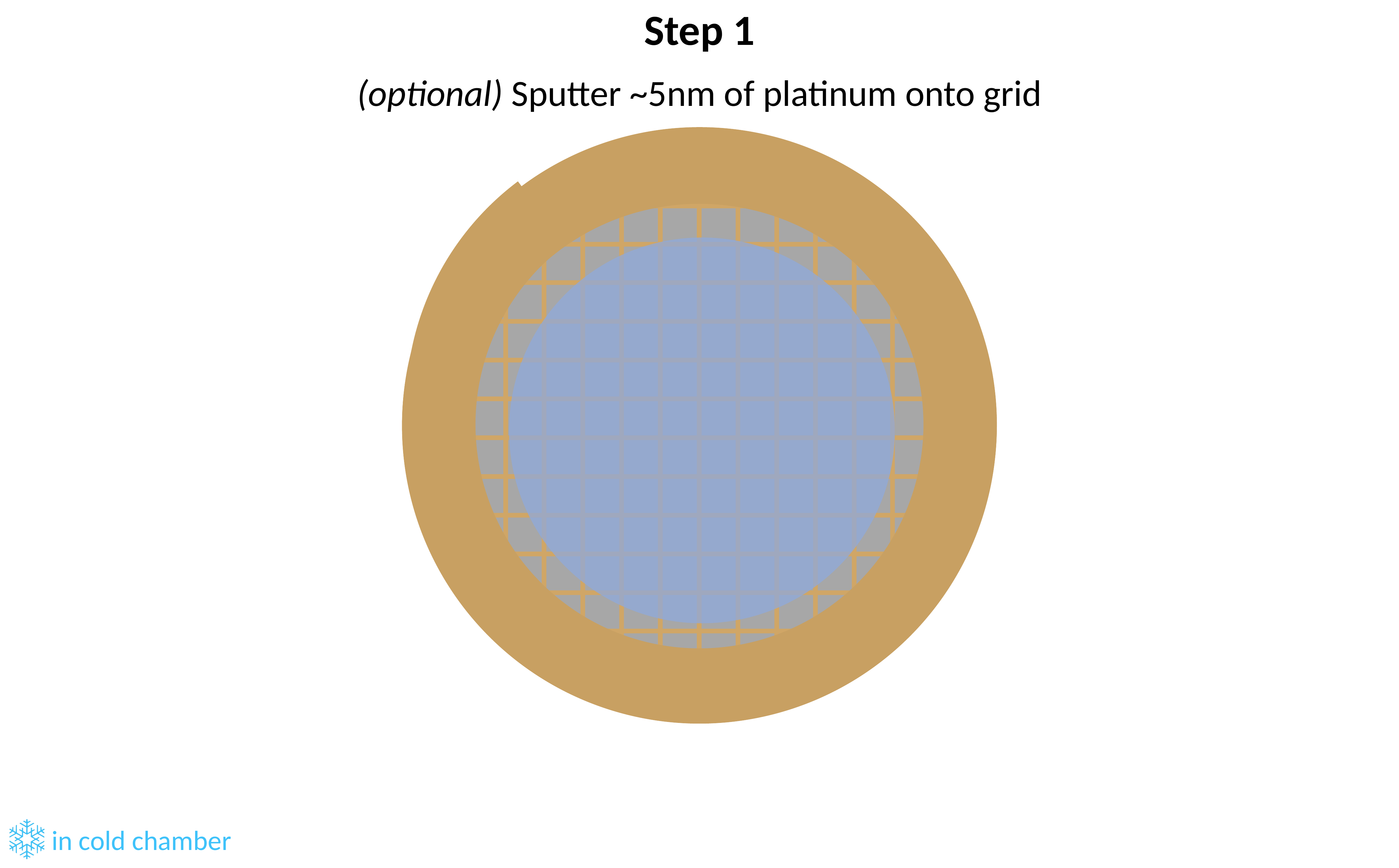

Step 1
(optional) Sputter ~5nm of platinum onto grid
in cold chamber

## Slide 68
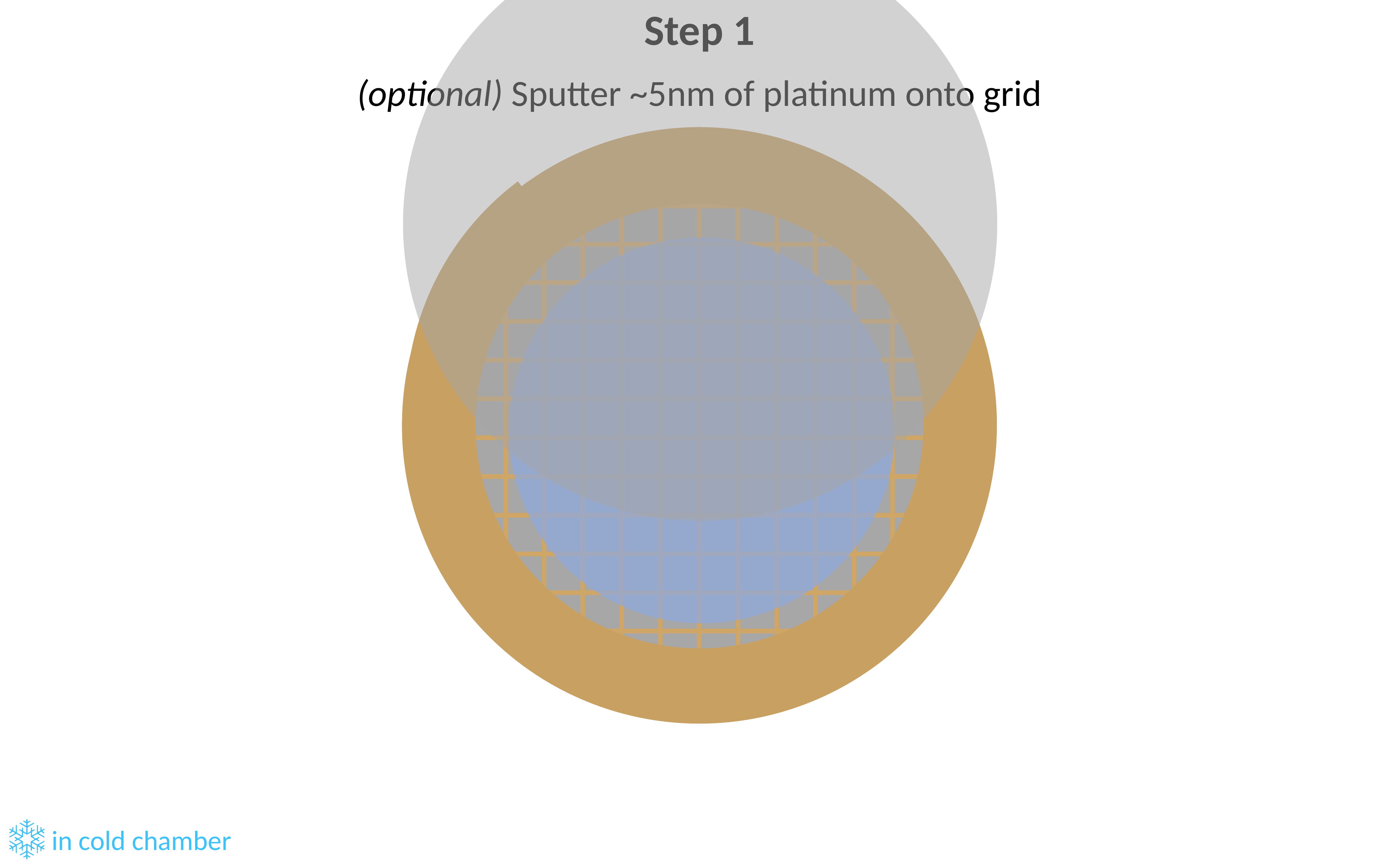

Step 1
(optional) Sputter ~5nm of platinum onto grid
in cold chamber

## Slide 69
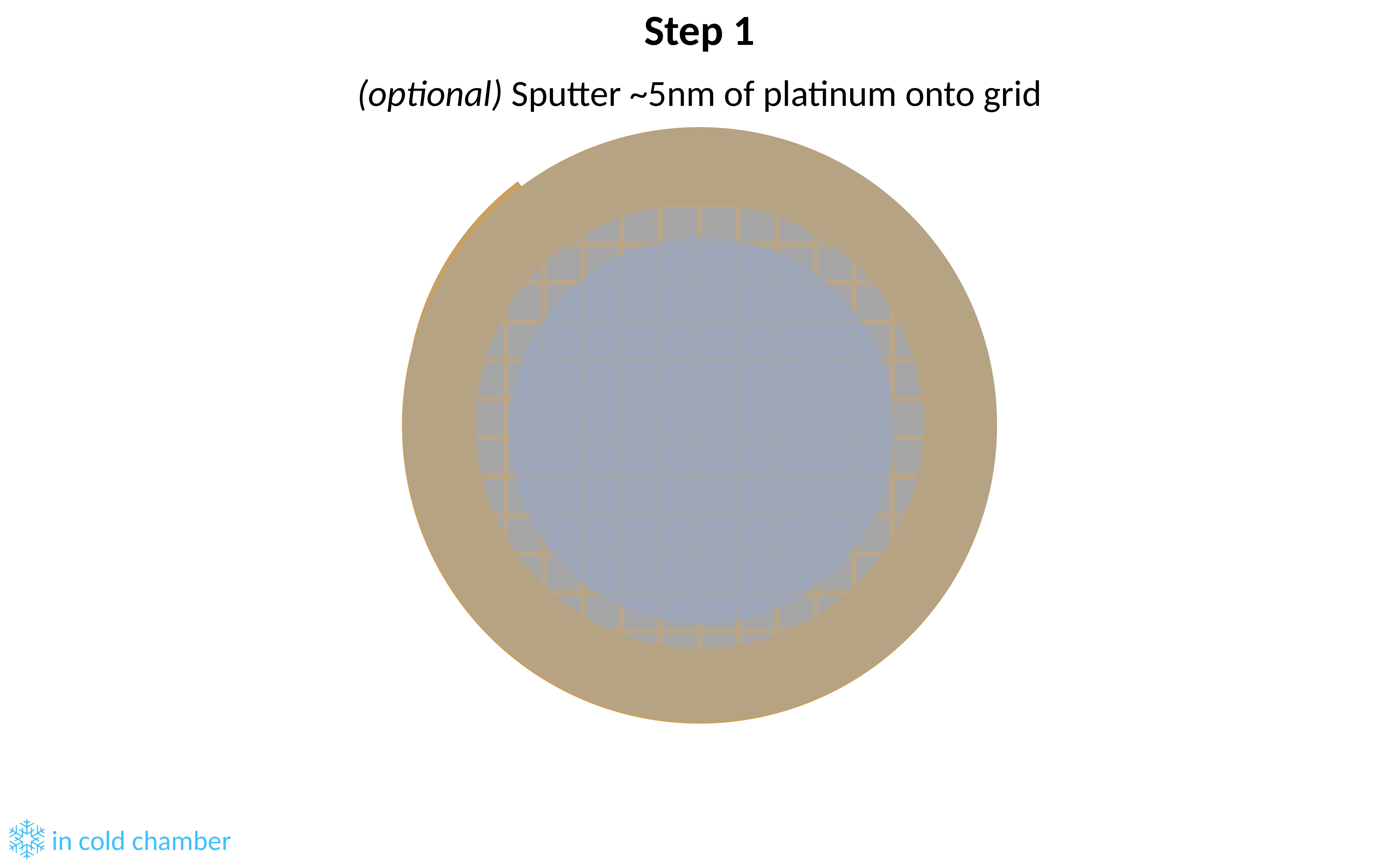

Step 1
(optional) Sputter ~5nm of platinum onto grid
in cold chamber

## Slide 70
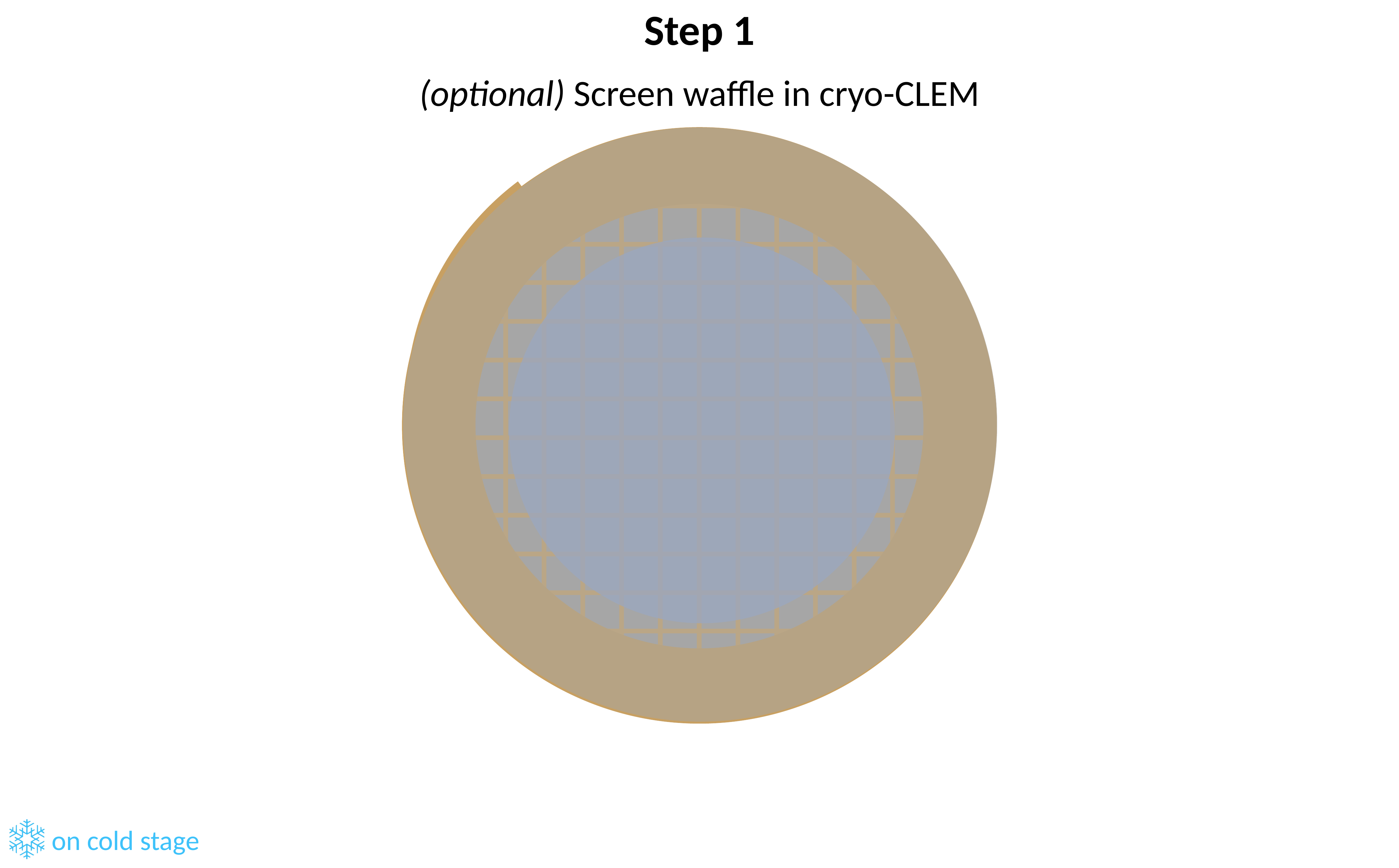

Step 1
(optional) Screen waffle in cryo-CLEM
on cold stage

## Slide 71
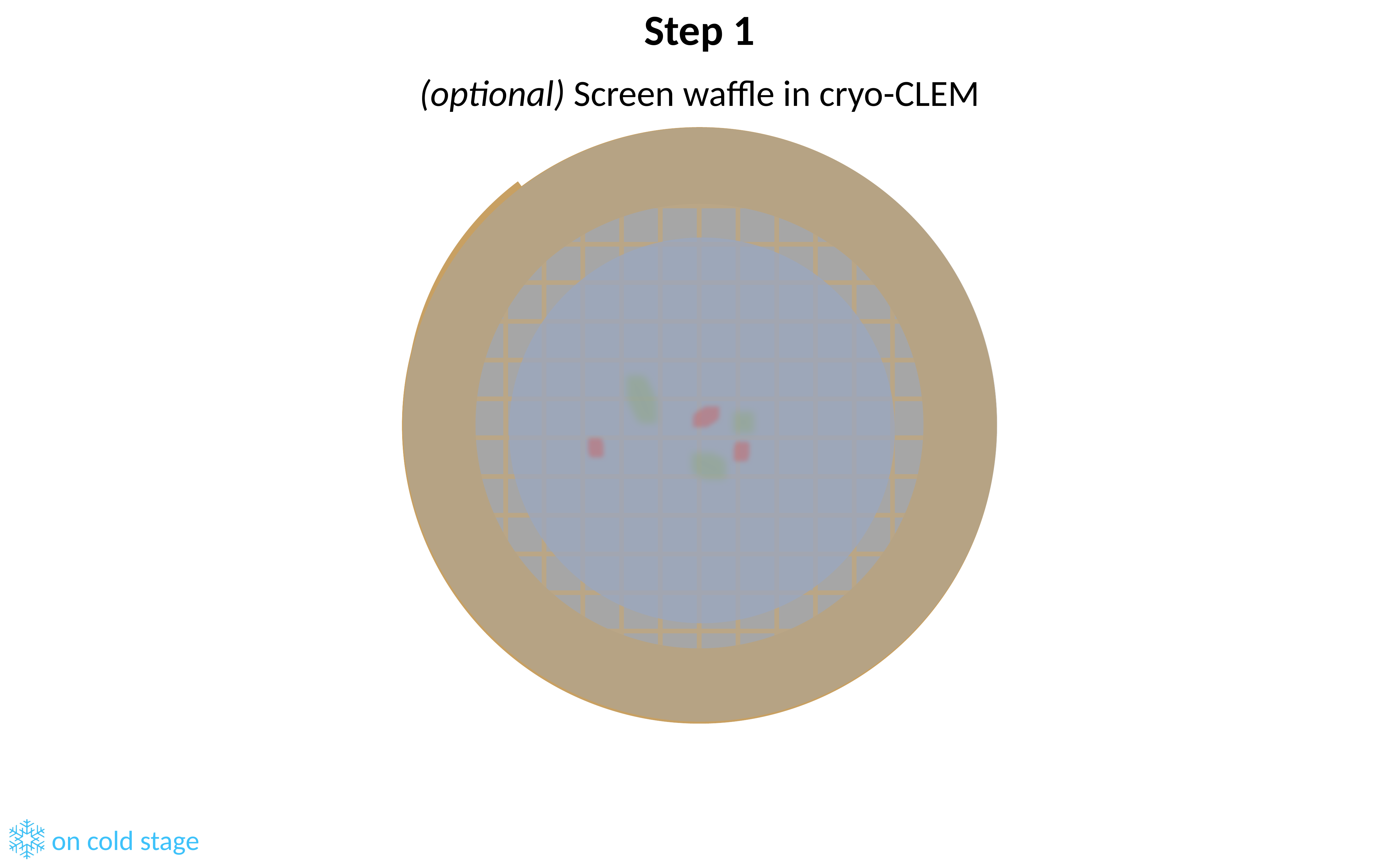

Step 1
(optional) Screen waffle in cryo-CLEM
on cold stage

## Slide 72
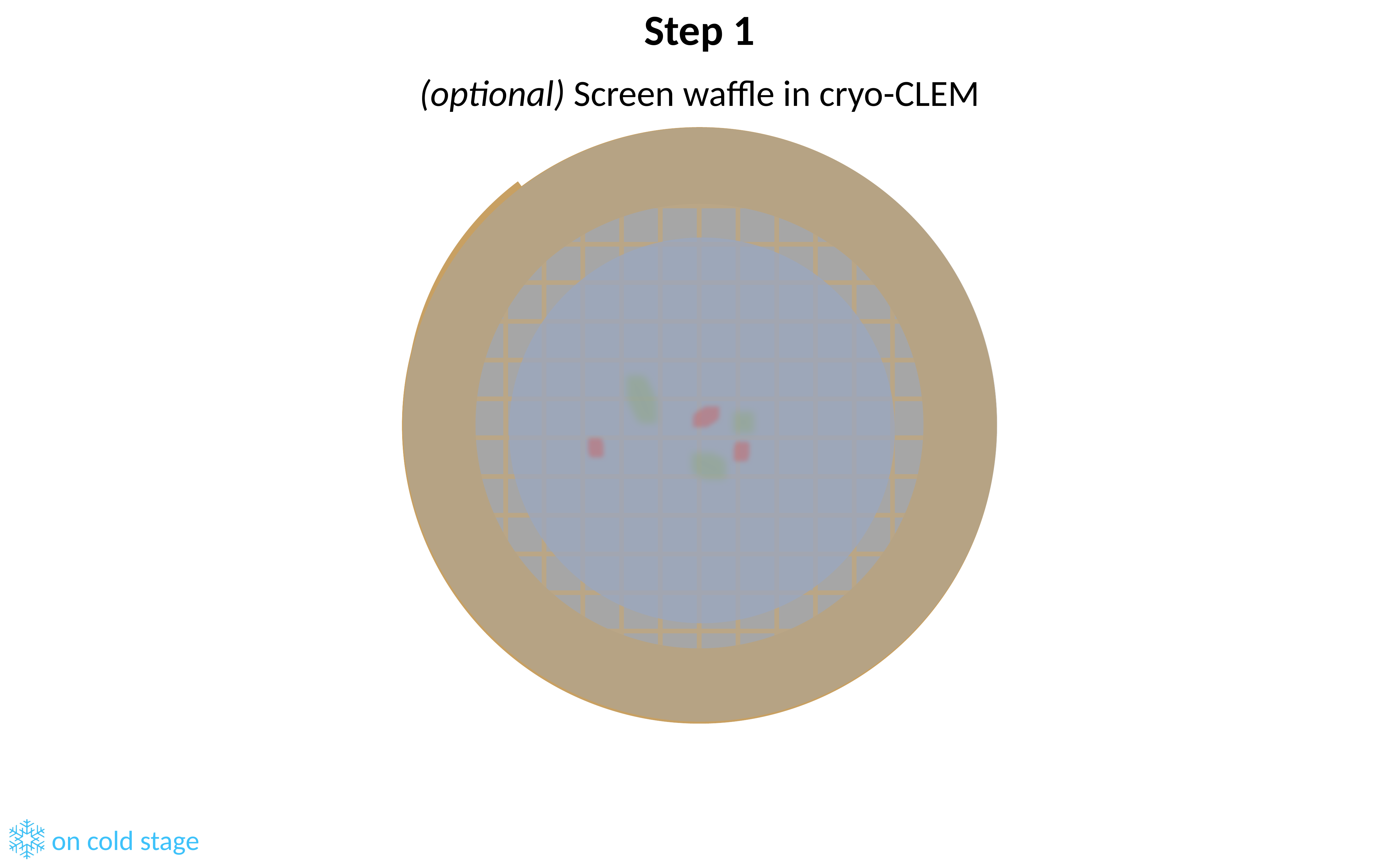

Step 1
(optional) Screen waffle in cryo-CLEM
on cold stage

## Slide 73
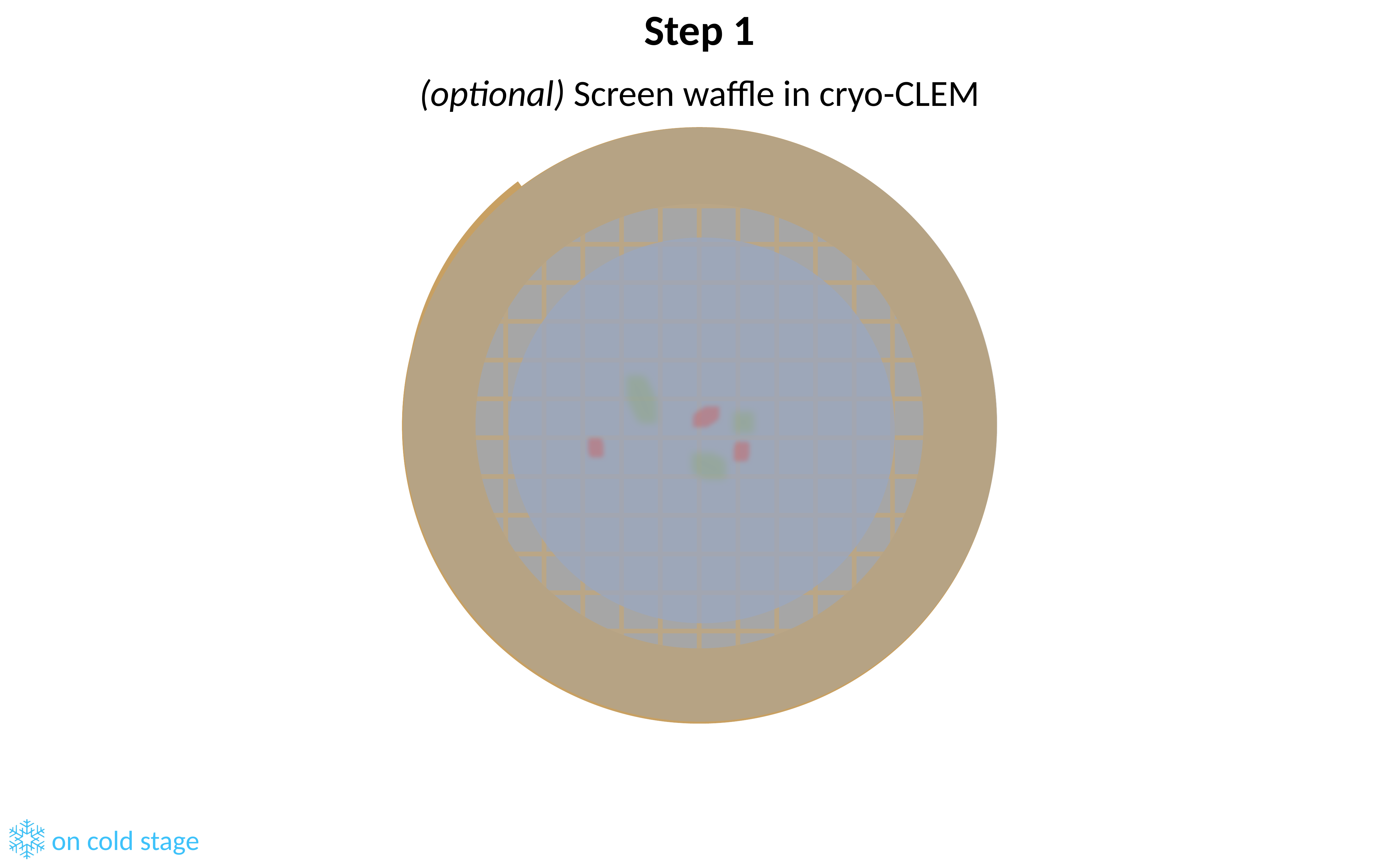

Step 1
(optional) Screen waffle in cryo-CLEM
on cold stage

## Slide 74
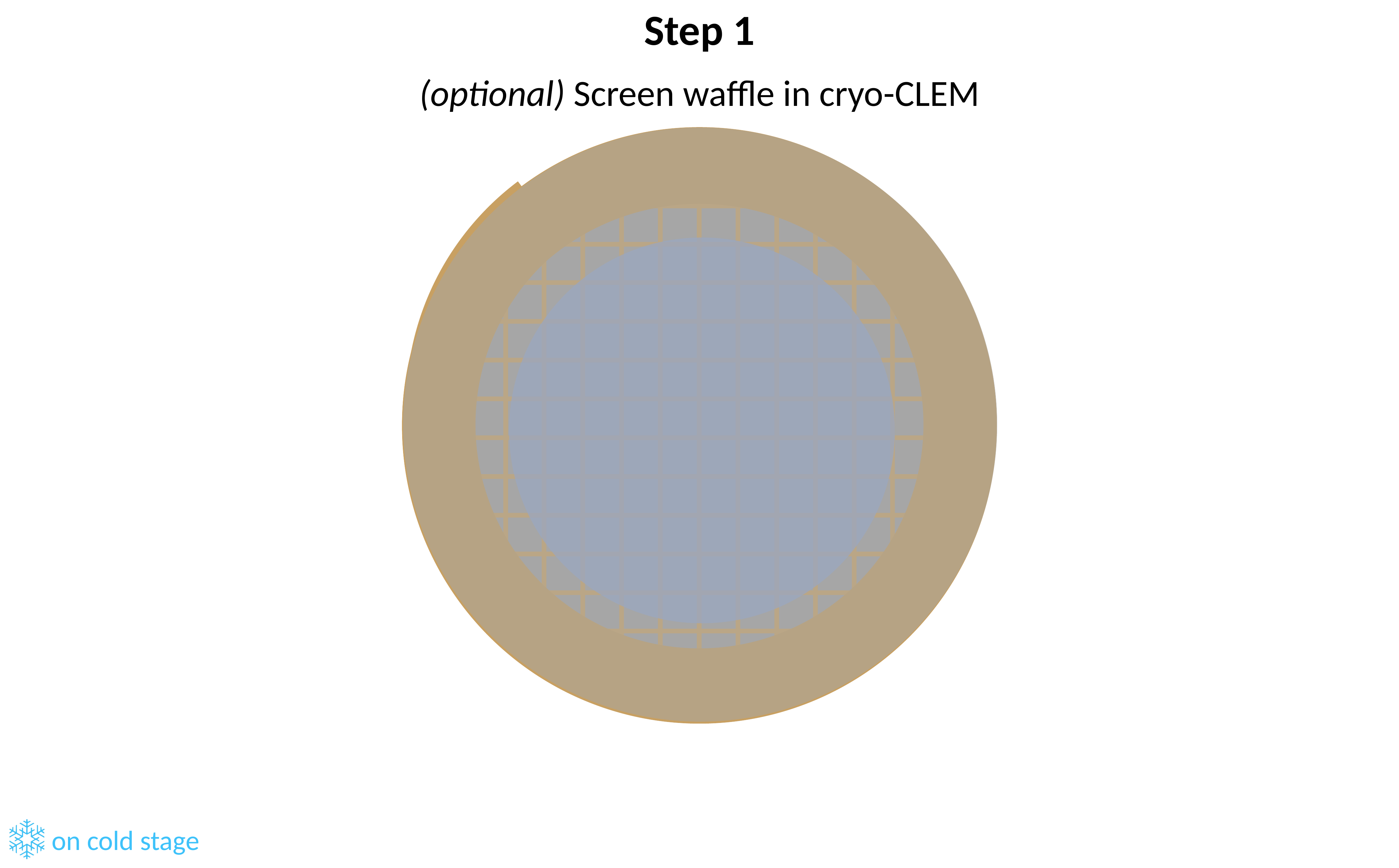

Step 1
(optional) Screen waffle in cryo-CLEM
on cold stage

## Slide 75
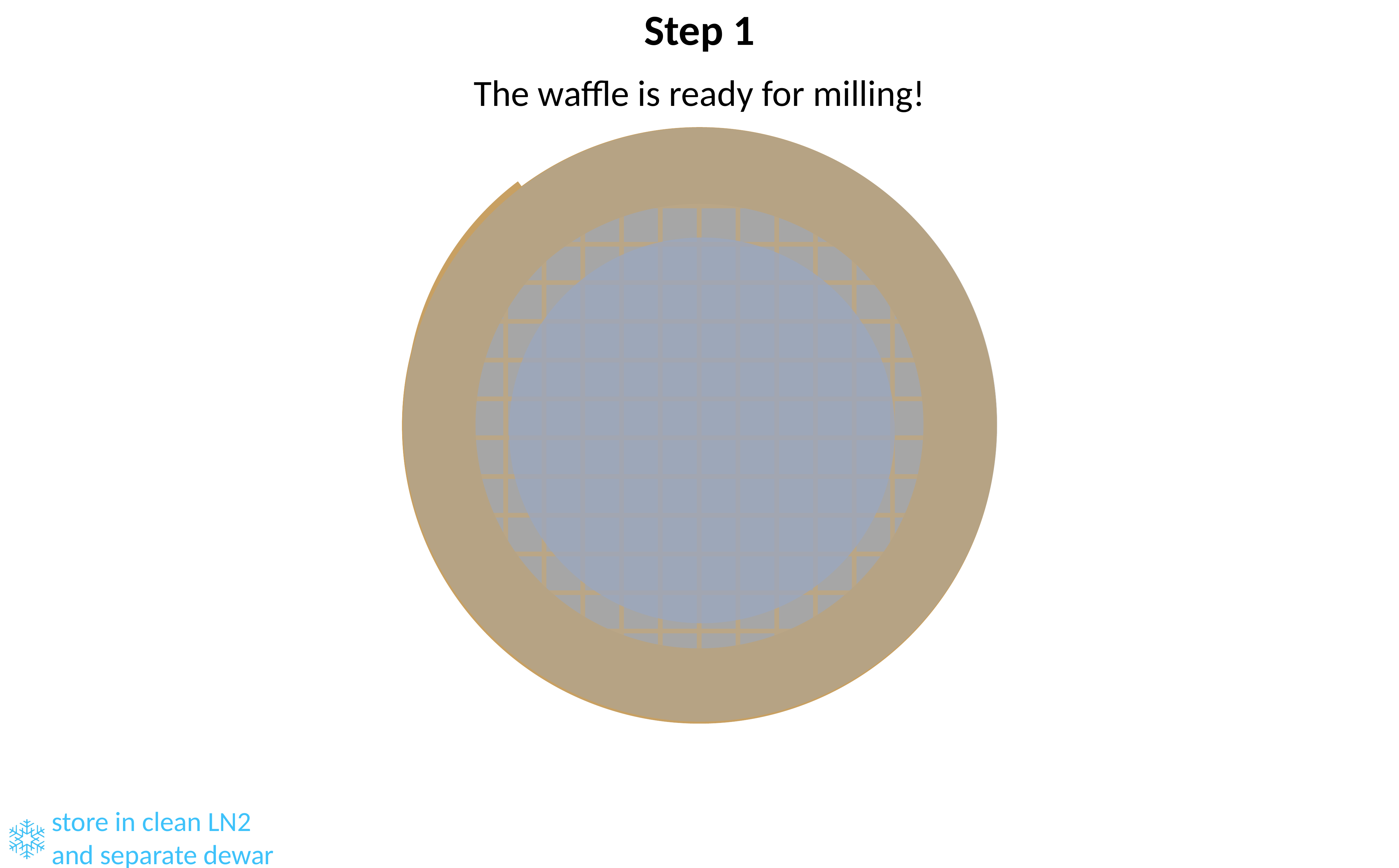

Step 1
The waffle is ready for milling!
store in clean LN2 and separate dewar

## Slide 76
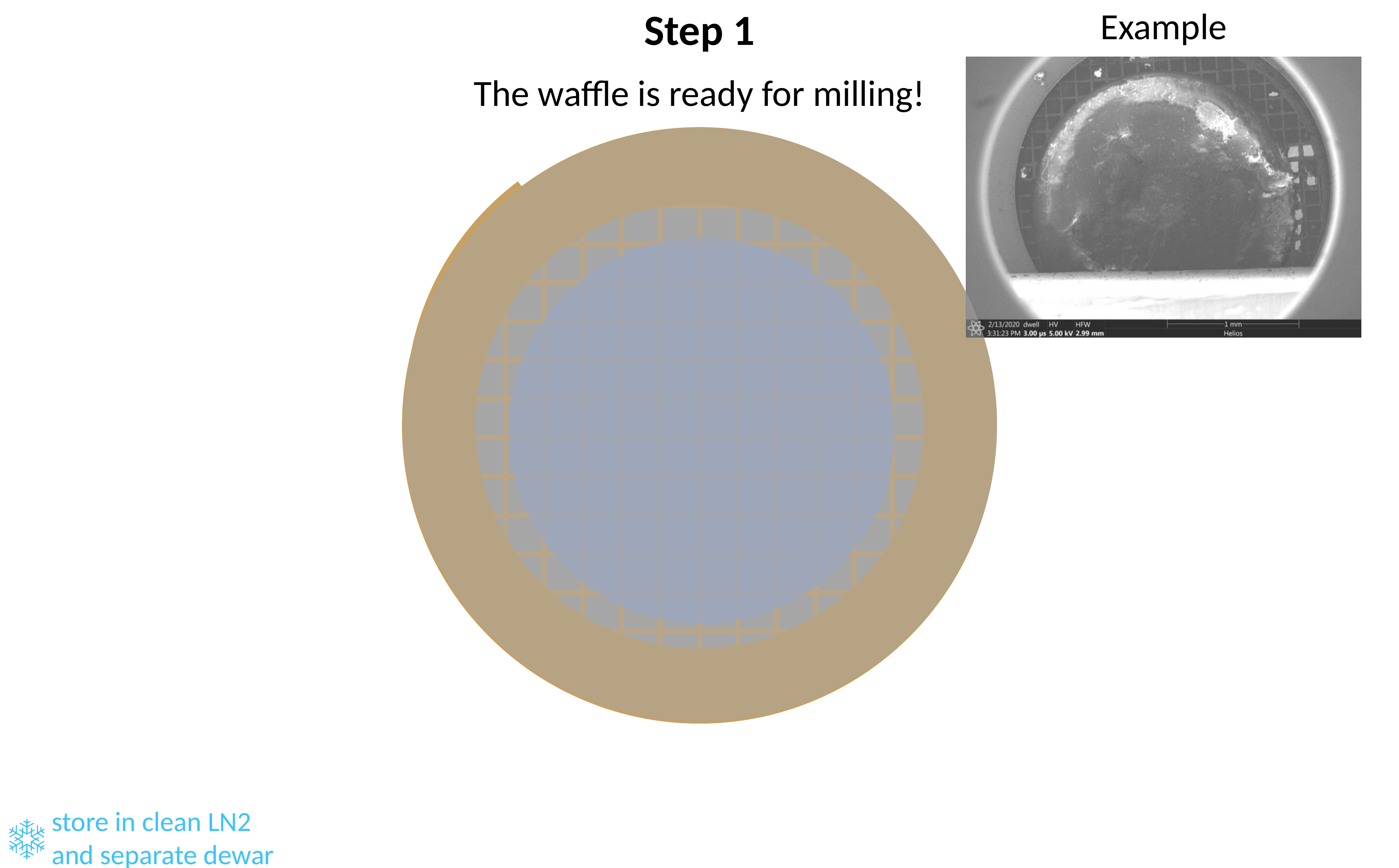

Example
Step 1
The waffle is ready for milling!
store in clean LN2 and separate dewar

## Slide 77
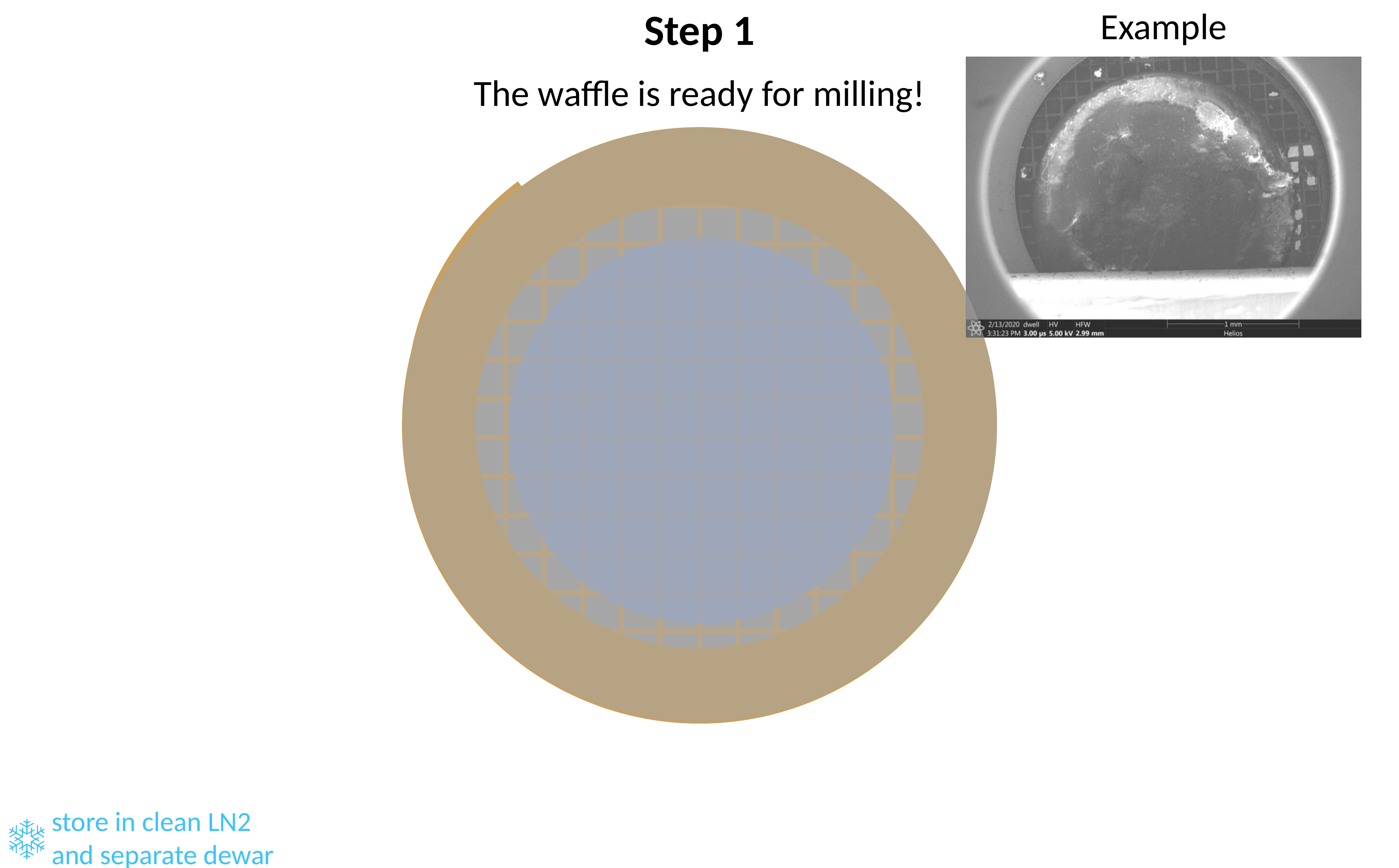

Example
Step 1
The waffle is ready for milling!
store in clean LN2 and separate dewar

## Slide 78
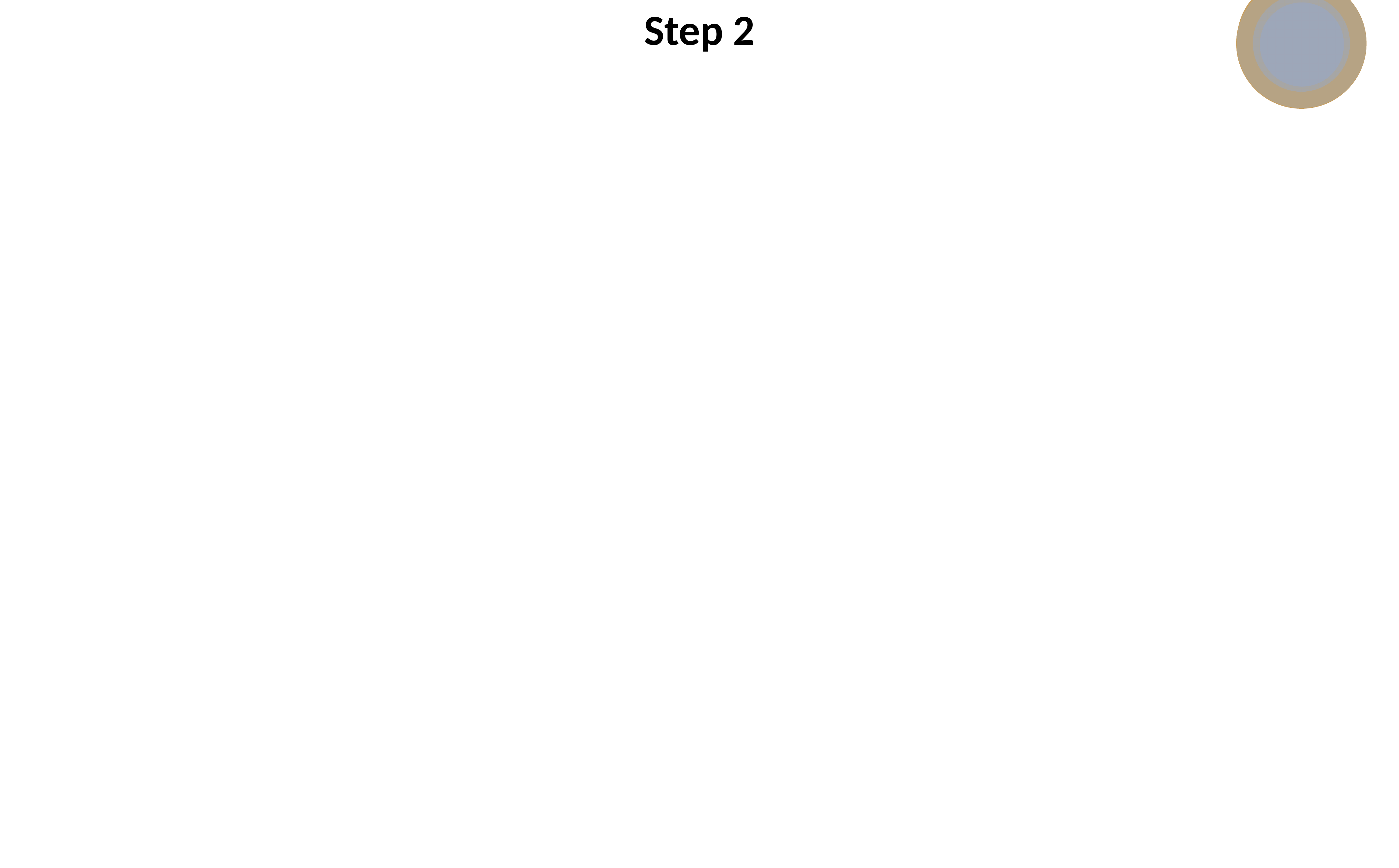

Step 2

## Slide 79
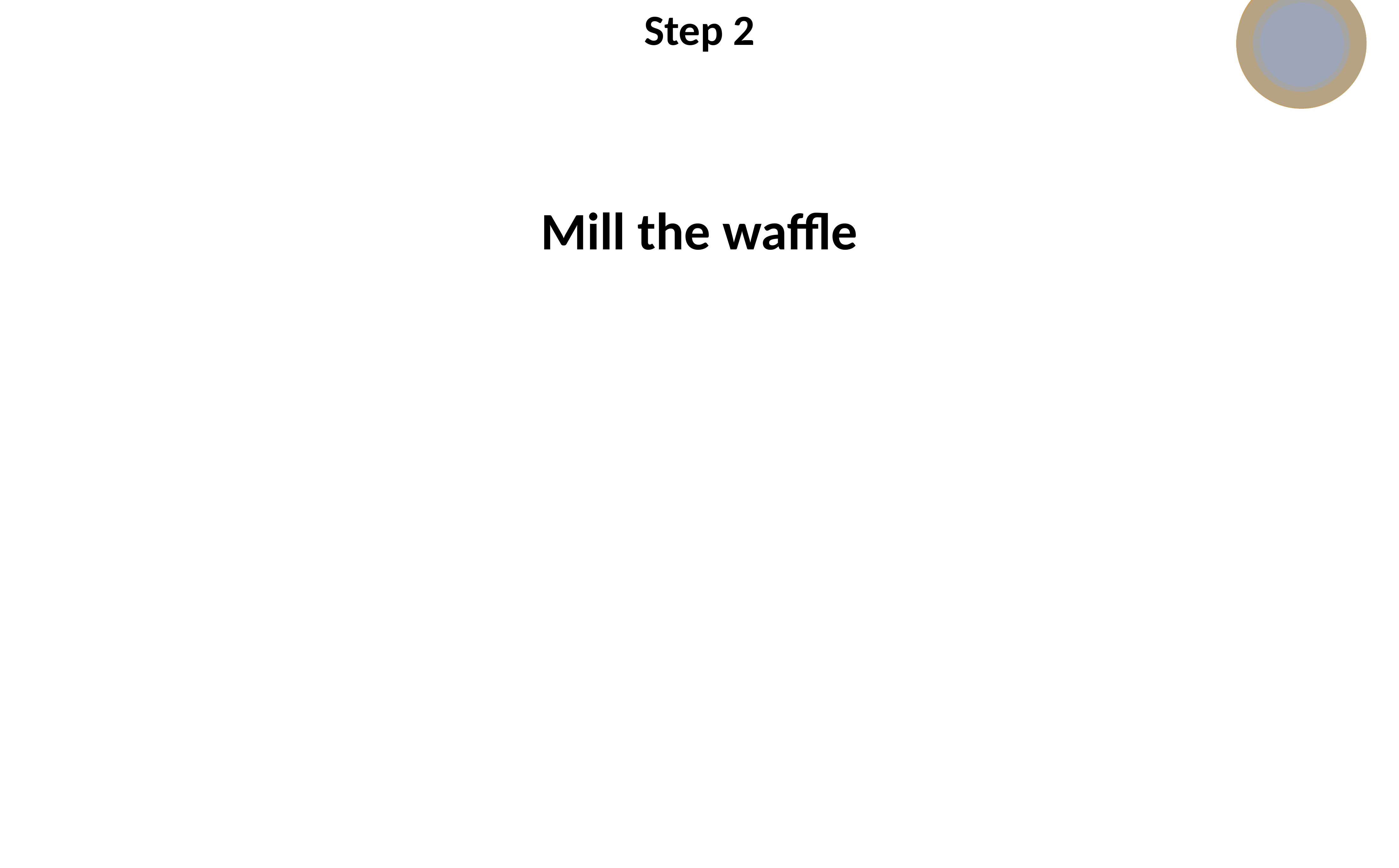

Step 2
Mill the waffle

## Slide 80
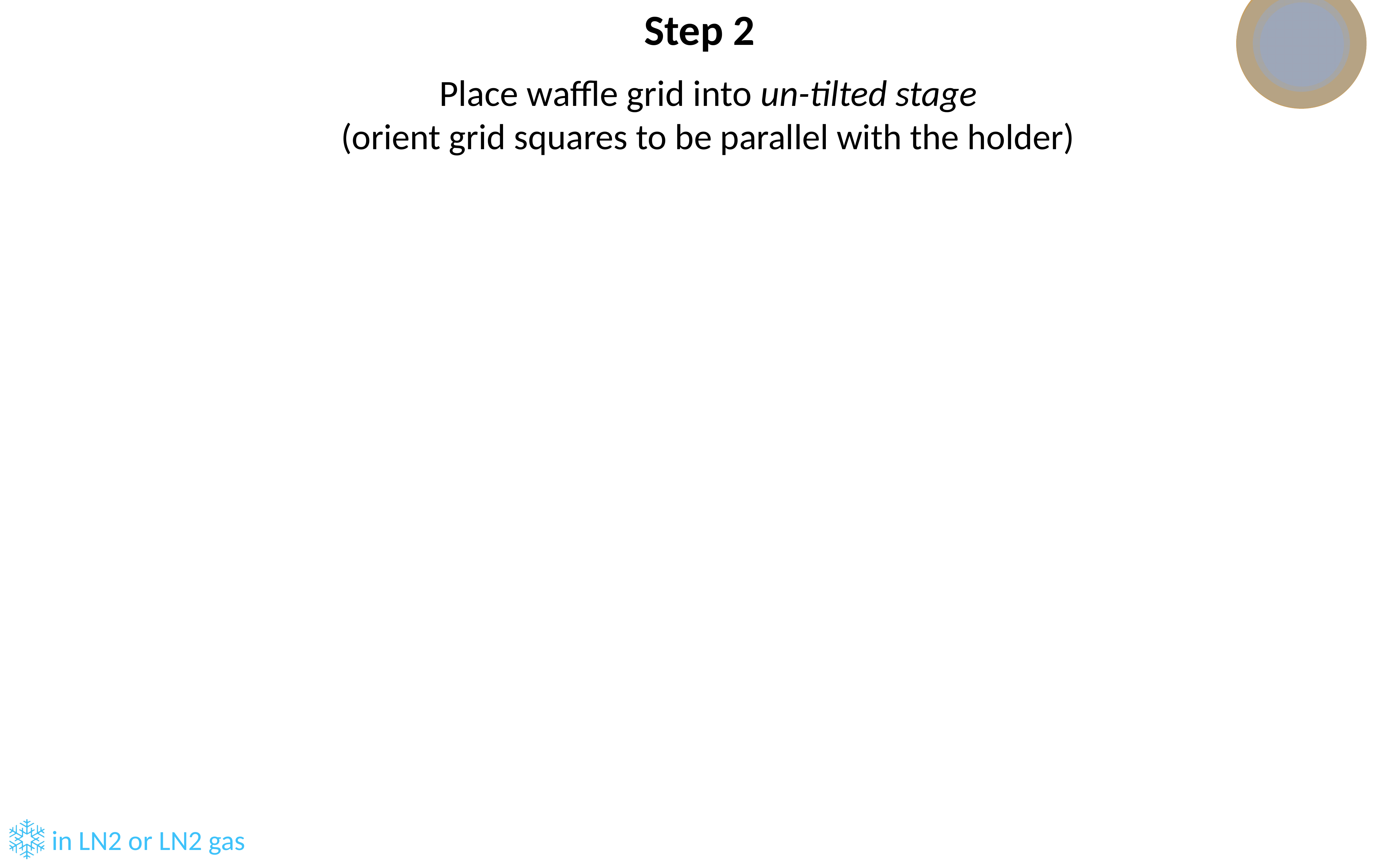

Step 2
Place waffle grid into un-tilted stage
(orient grid squares to be parallel with the holder)
in LN2 or LN2 gas

## Slide 81
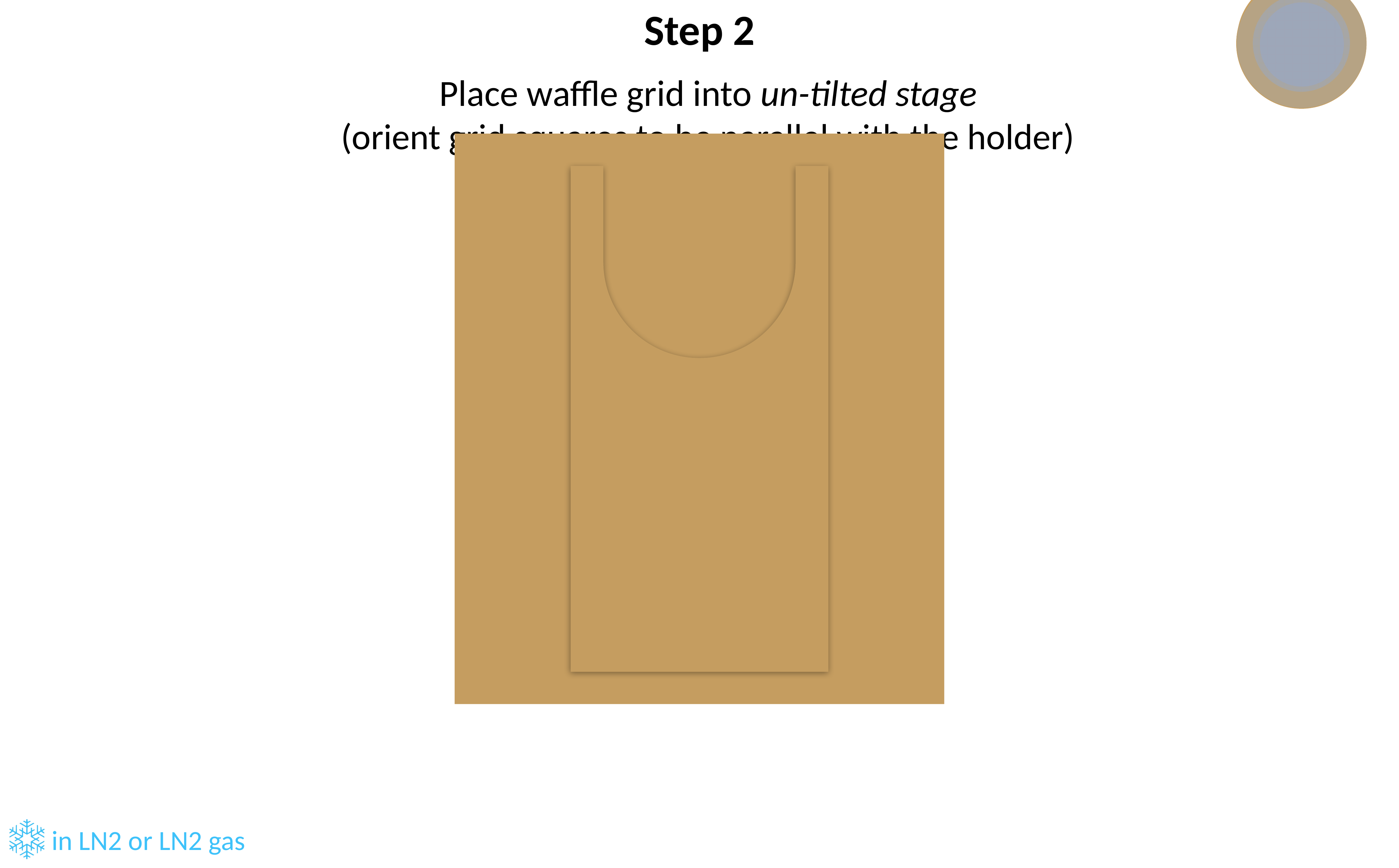

Step 2
Place waffle grid into un-tilted stage
(orient grid squares to be parallel with the holder)
in LN2 or LN2 gas

## Slide 82
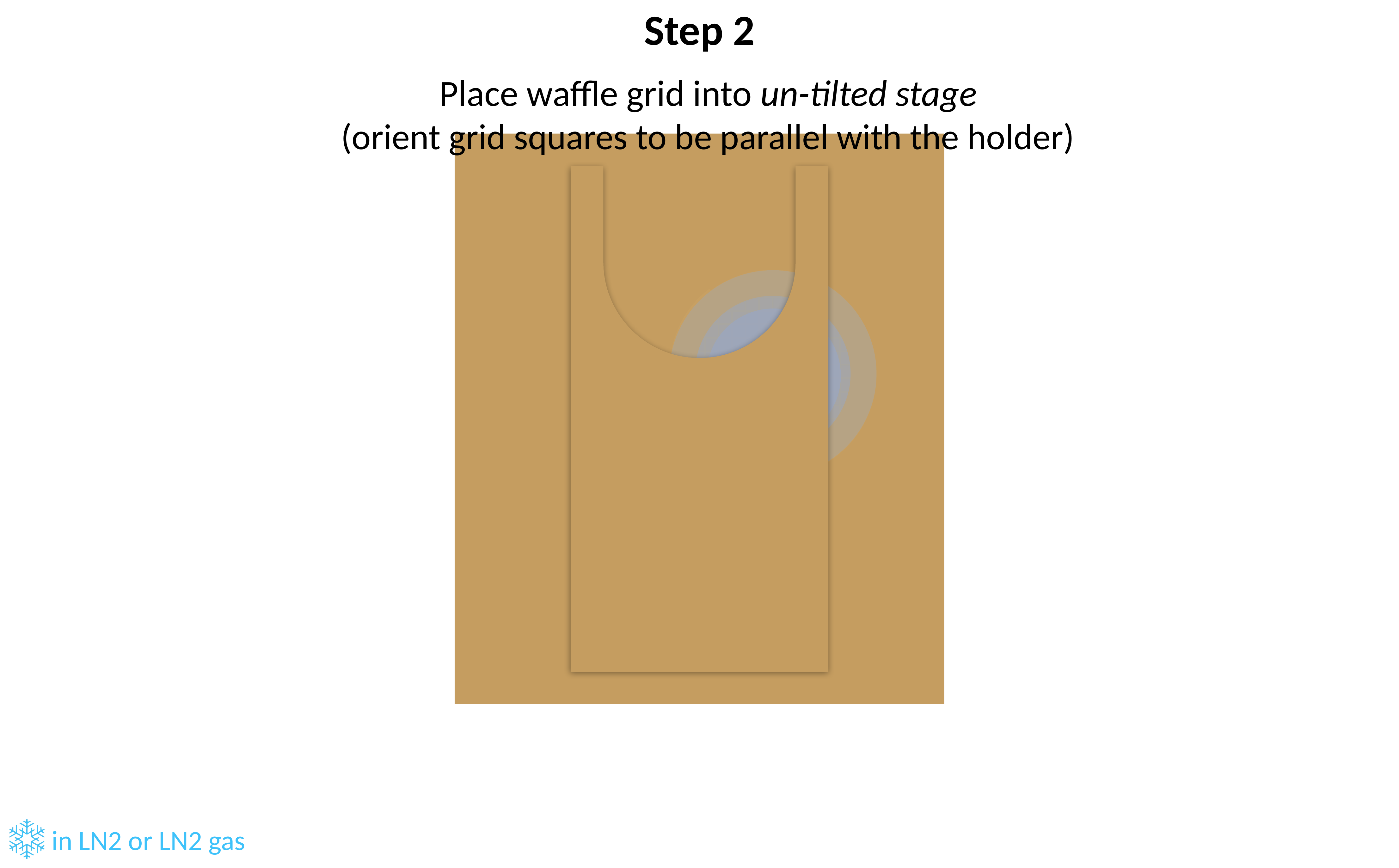

Step 2
Place waffle grid into un-tilted stage
(orient grid squares to be parallel with the holder)
in LN2 or LN2 gas

## Slide 83
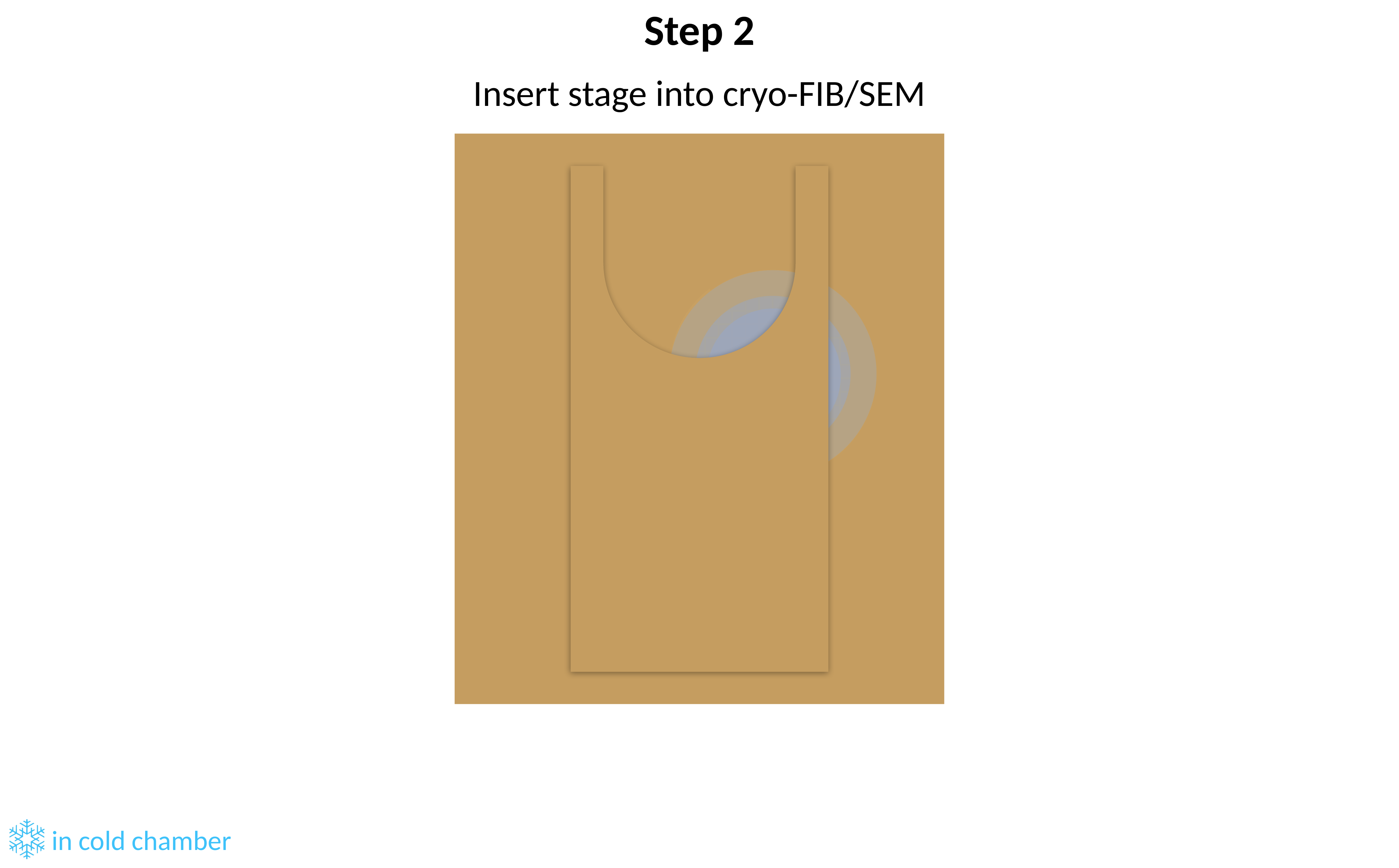

Step 2
Insert stage into cryo-FIB/SEM
in cold chamber

## Slide 84
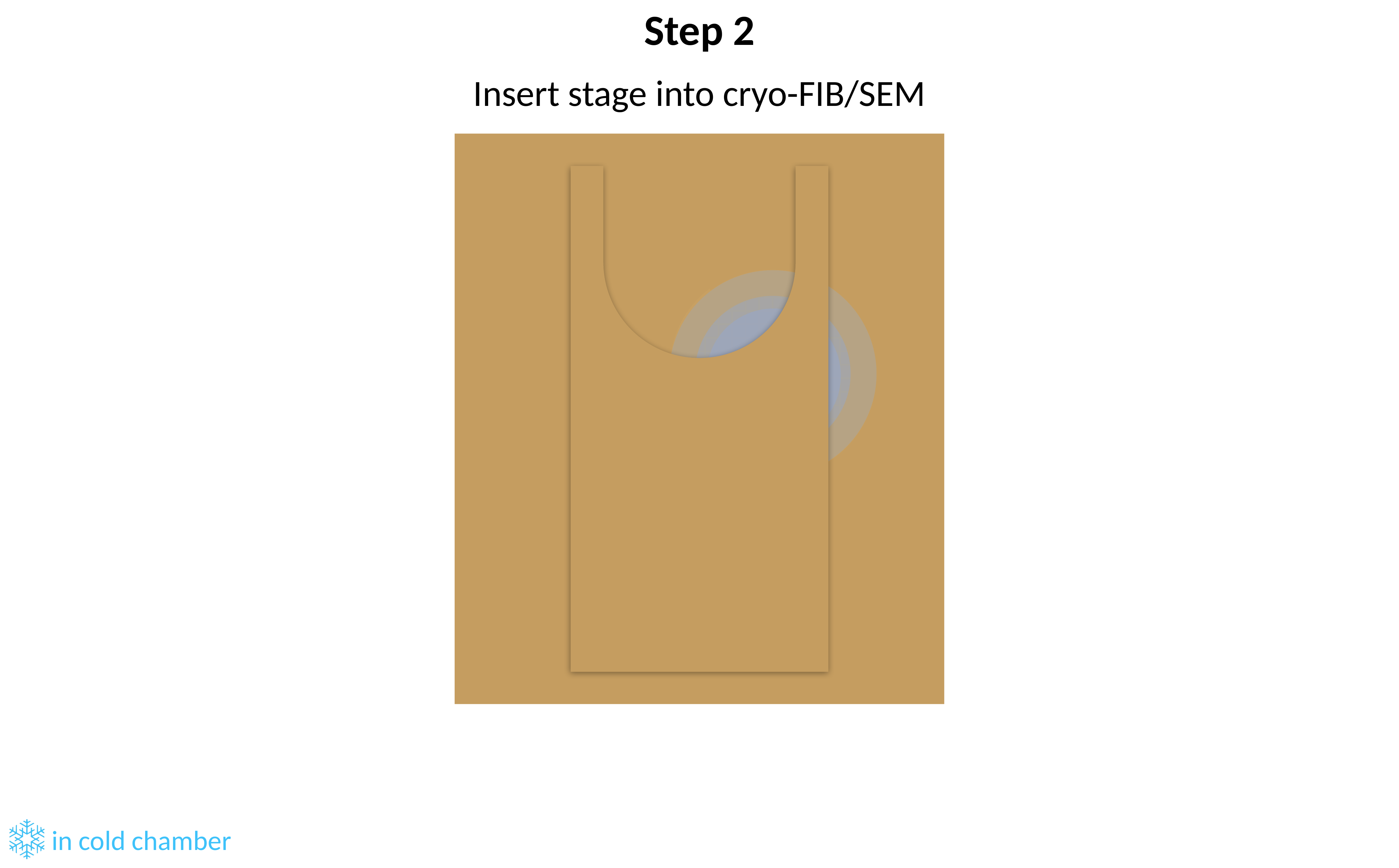

Step 2
Insert stage into cryo-FIB/SEM
in cold chamber

## Slide 85
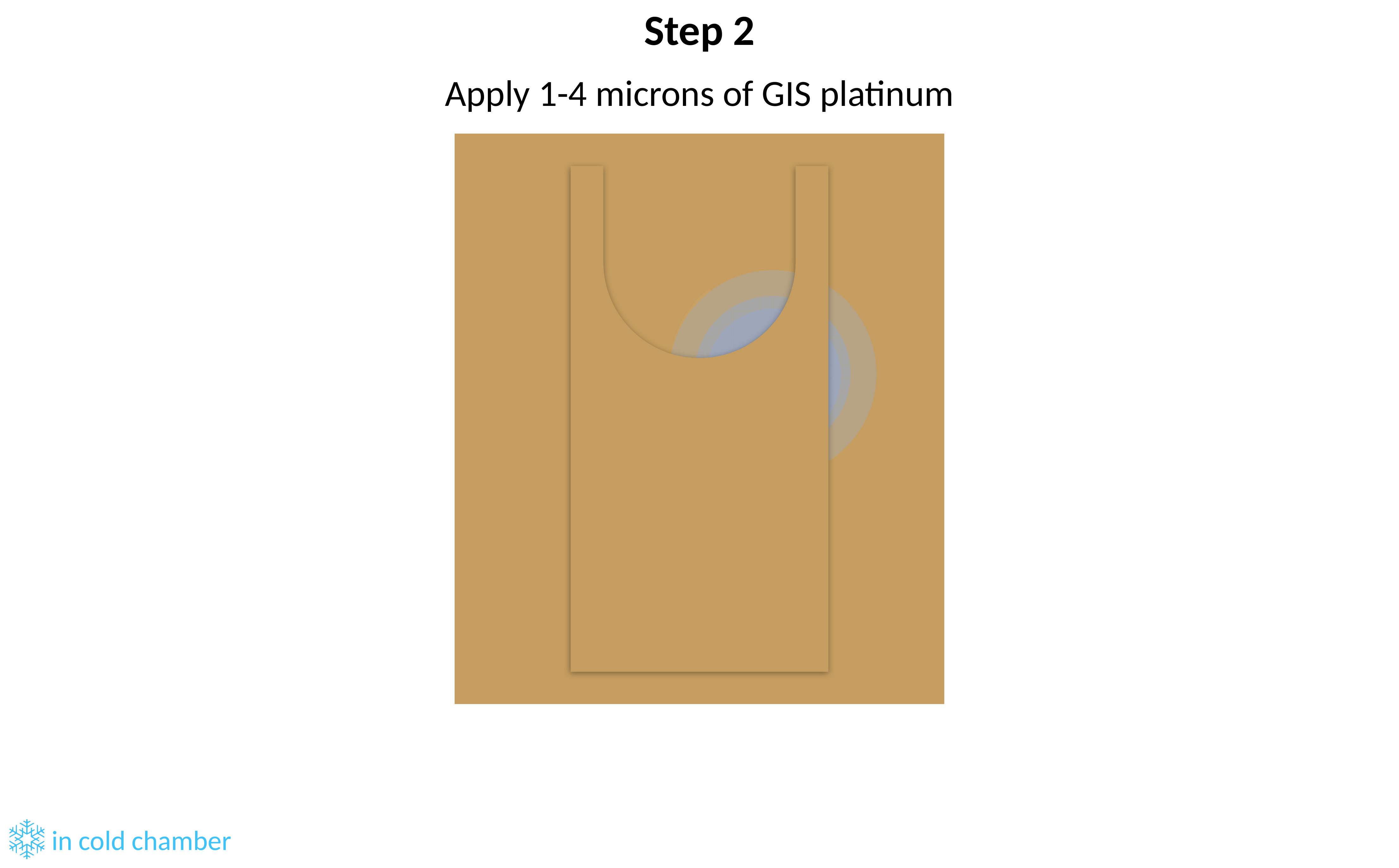

Step 2
Apply 1-4 microns of GIS platinum
in cold chamber

## Slide 86
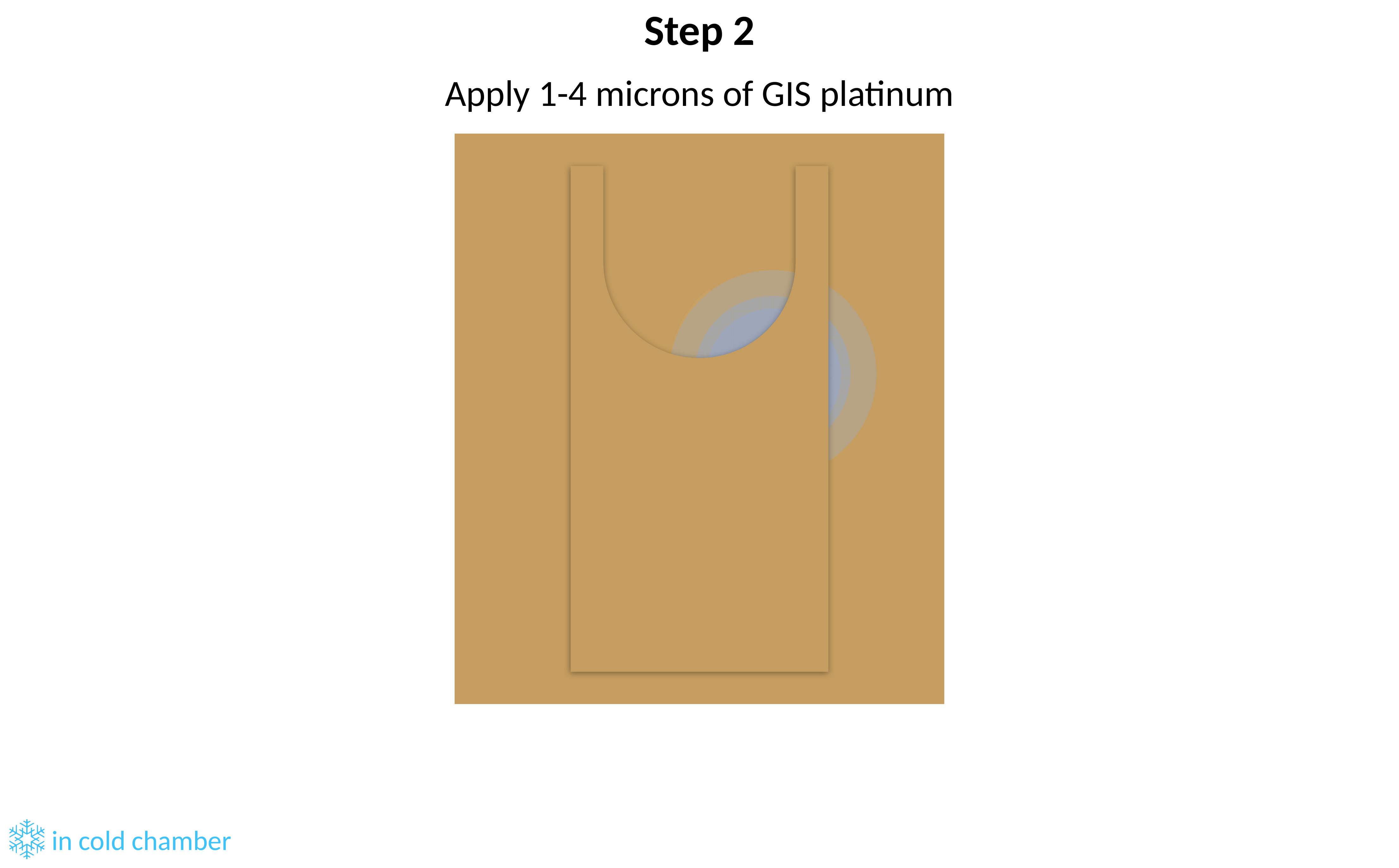

Step 2
Apply 1-4 microns of GIS platinum
in cold chamber

## Slide 87
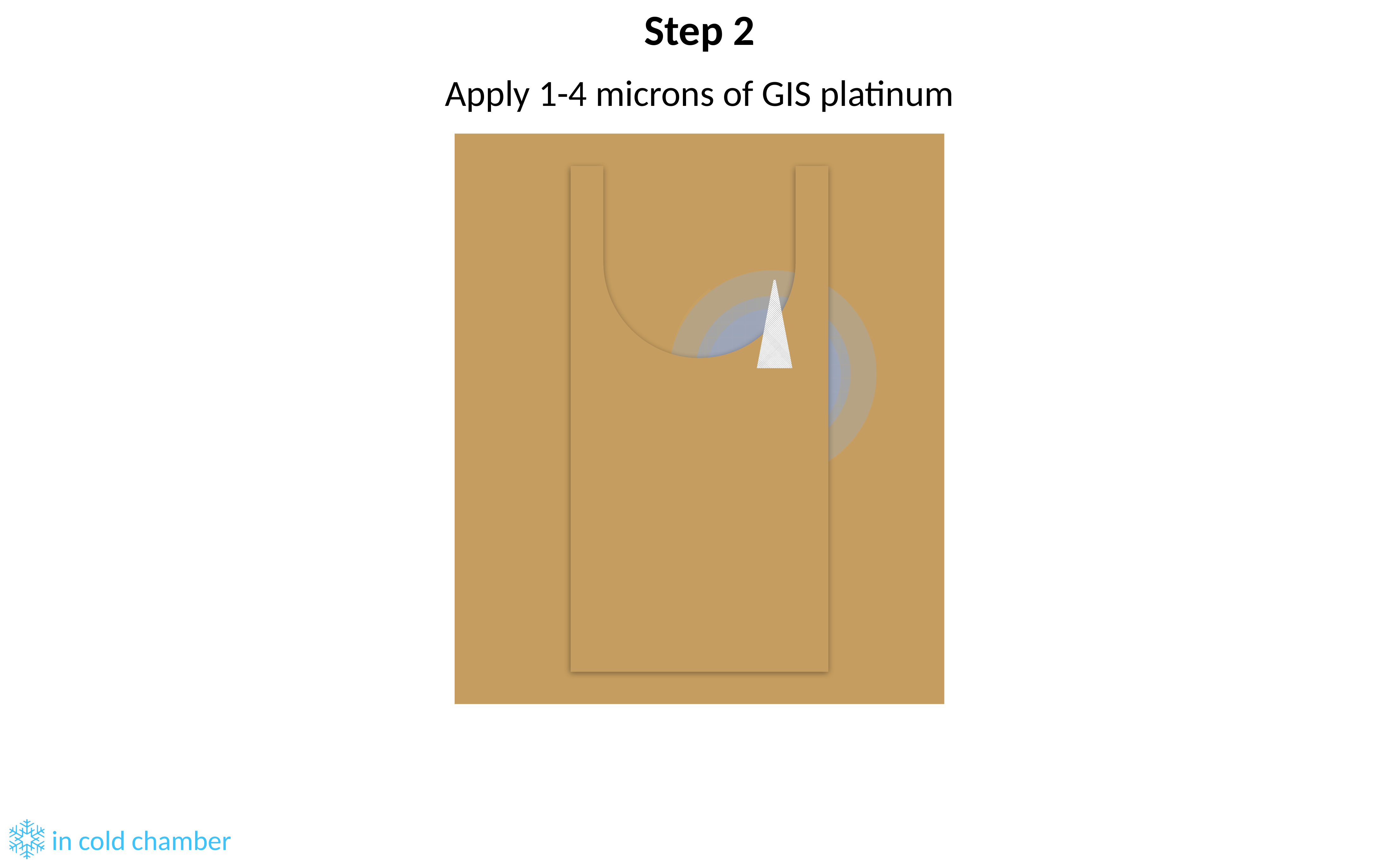

Step 2
Apply 1-4 microns of GIS platinum
in cold chamber

## Slide 88
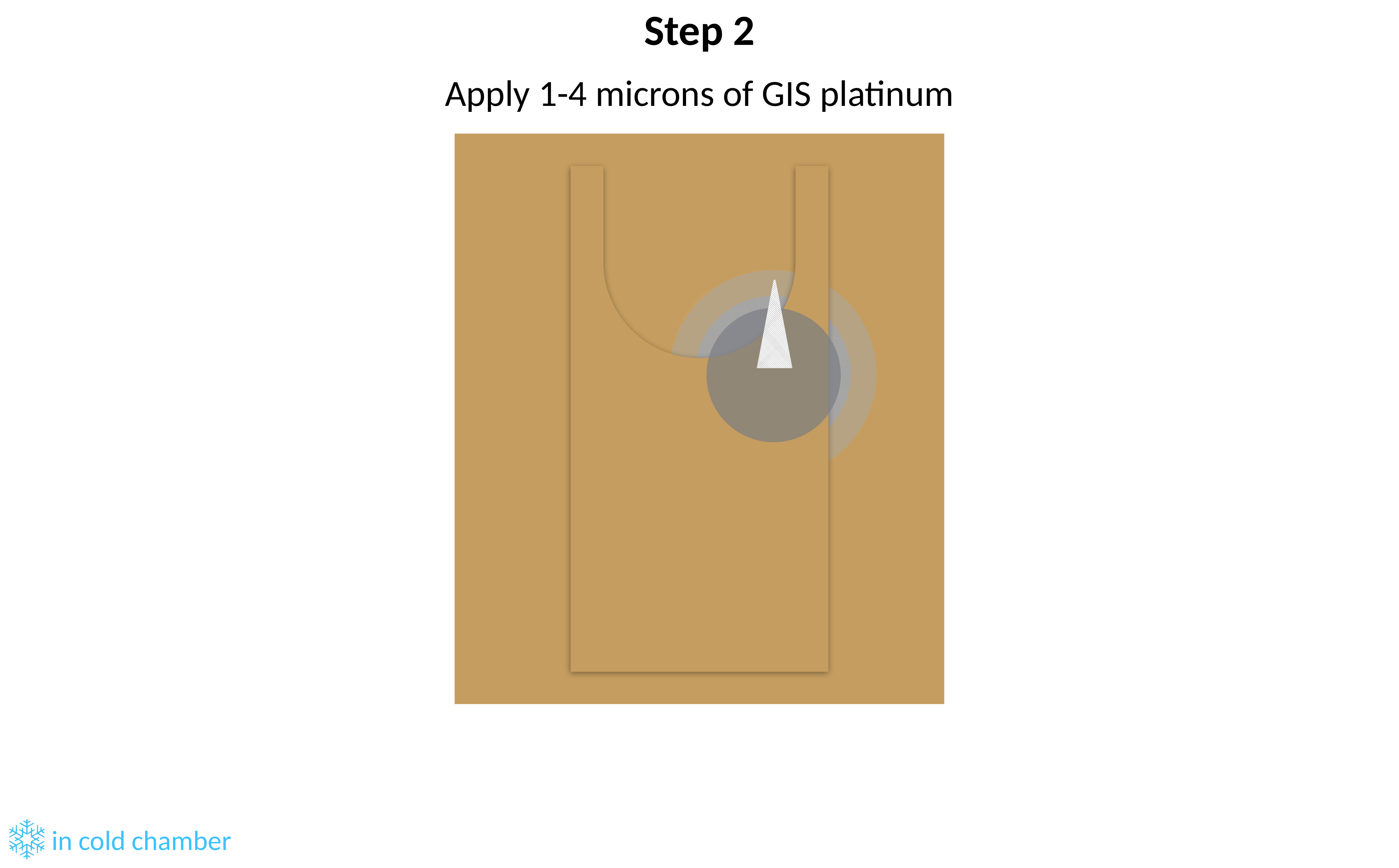

Step 2
Apply 1-4 microns of GIS platinum
in cold chamber

## Slide 89
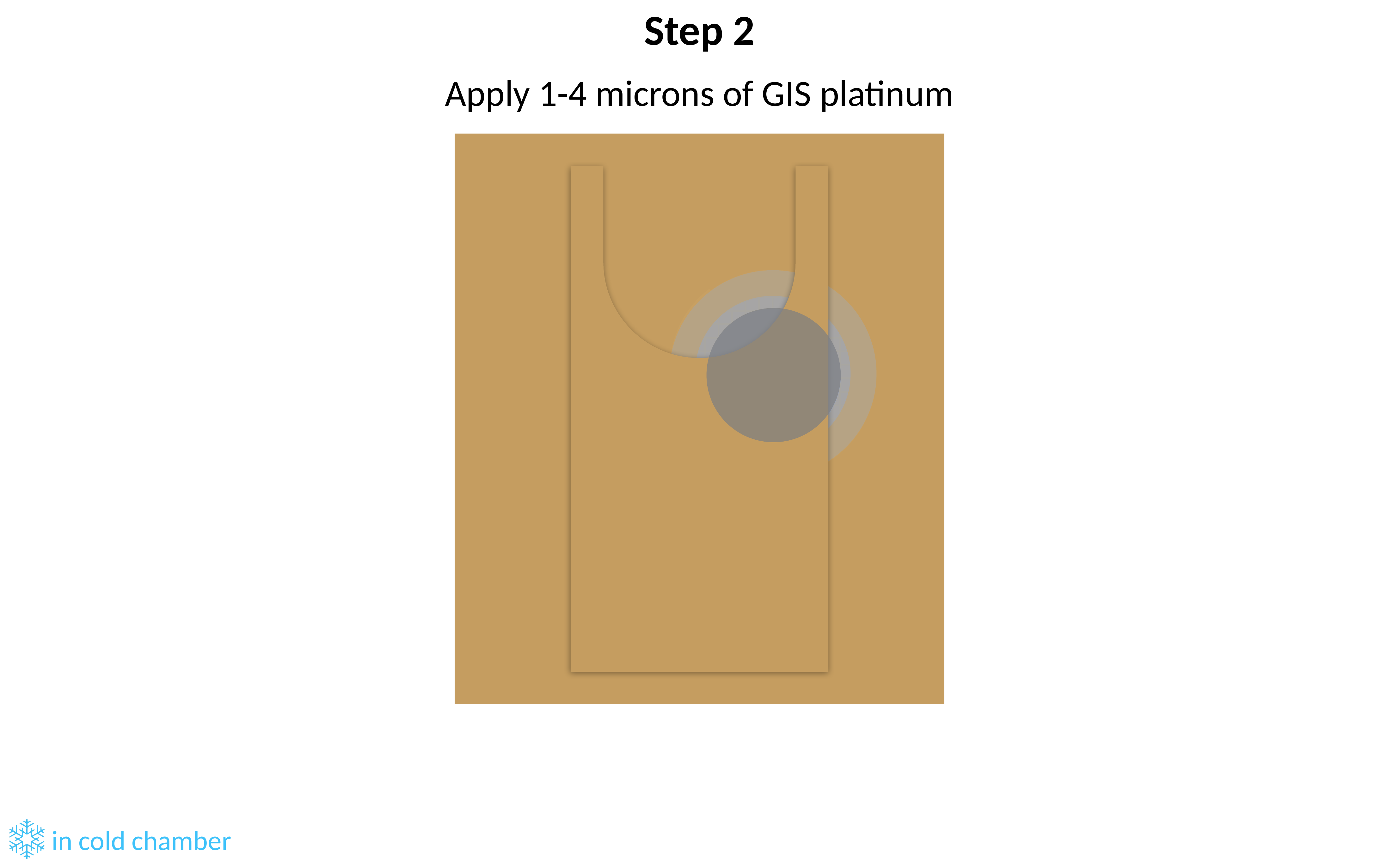

Step 2
Apply 1-4 microns of GIS platinum
in cold chamber

## Slide 90
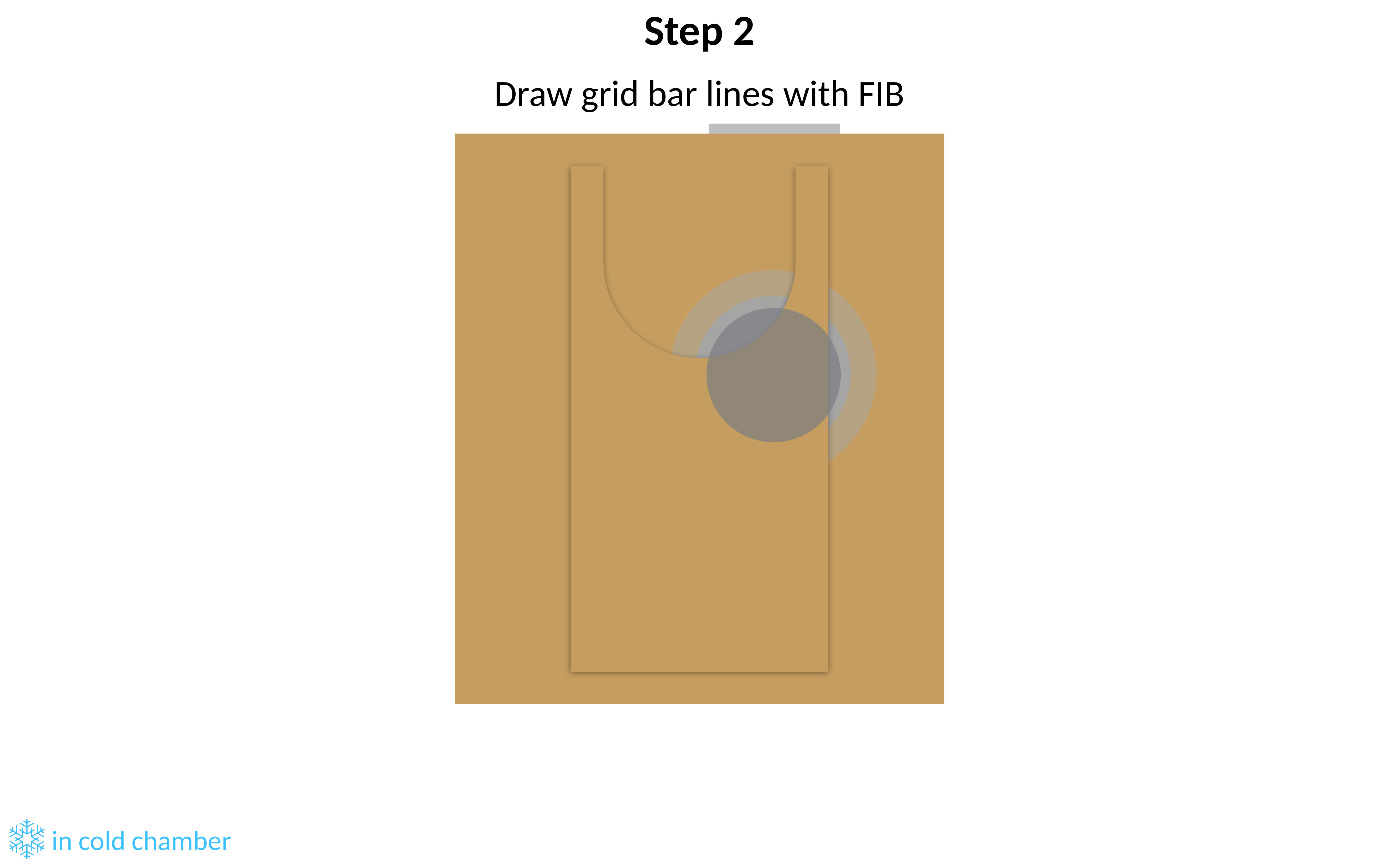

Step 2
Draw grid bar lines with FIB
in cold chamber

## Slide 91
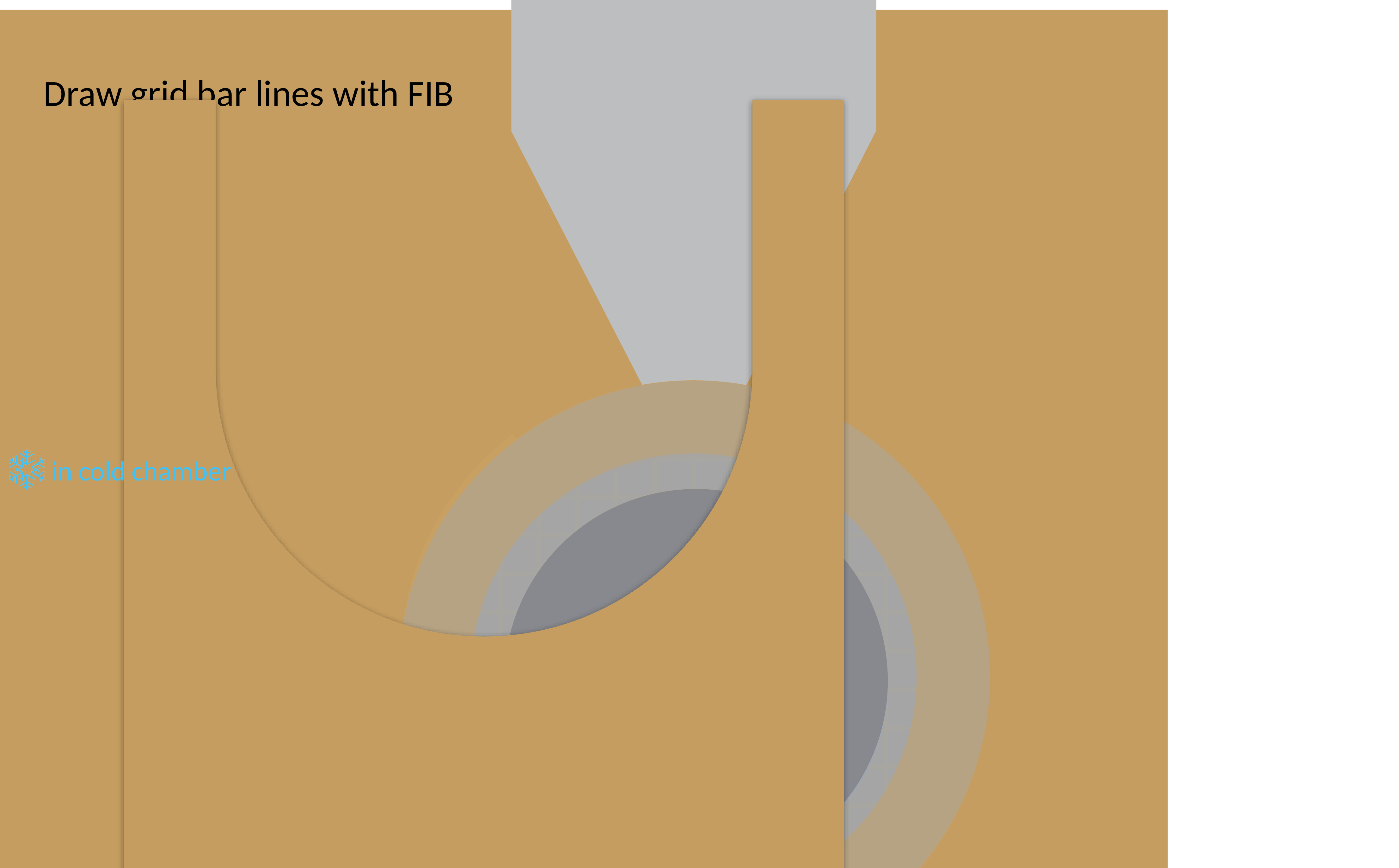

Step 2
Draw grid bar lines with FIB
in cold chamber

## Slide 92
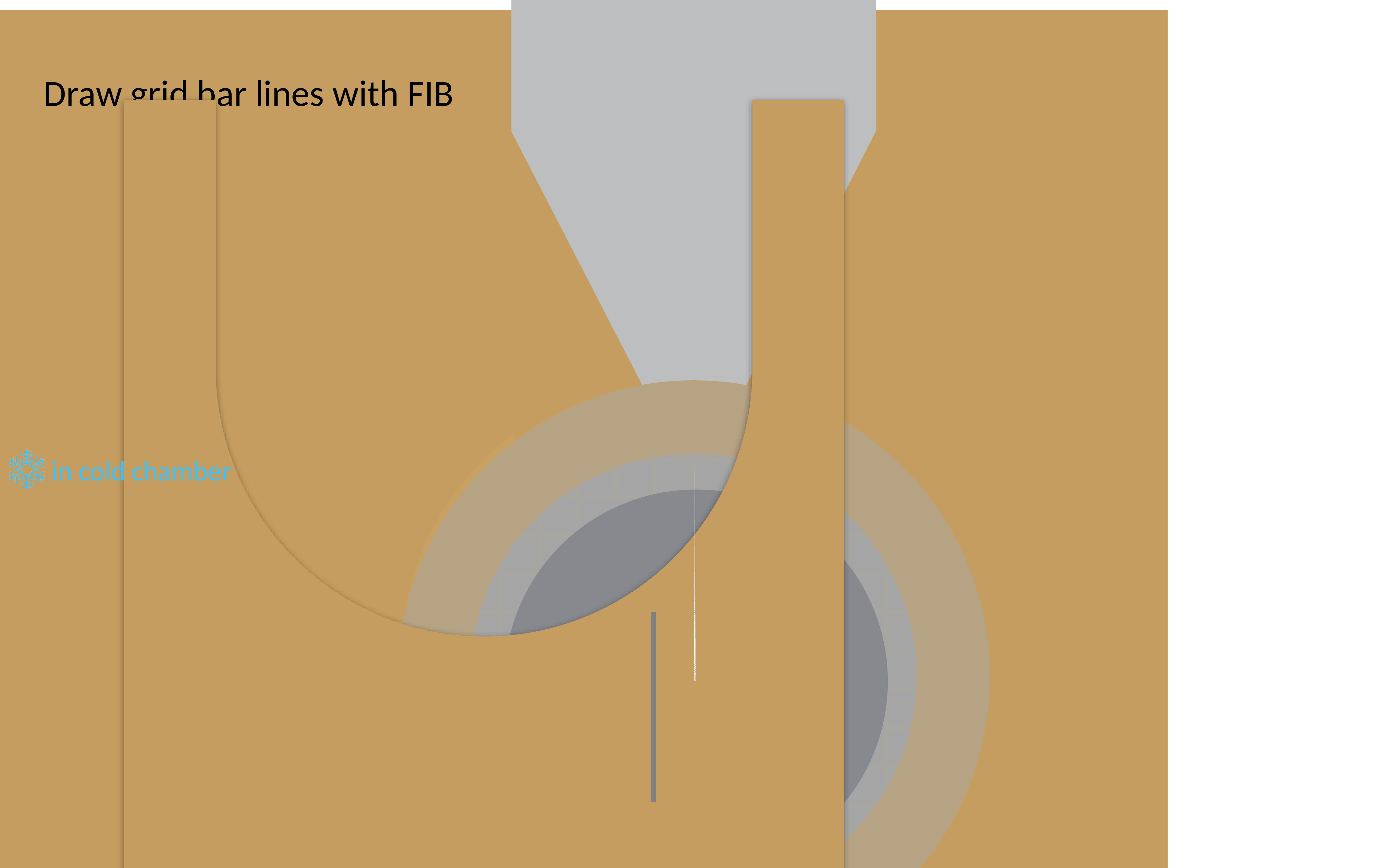

Step 2
Draw grid bar lines with FIB
in cold chamber

## Slide 93
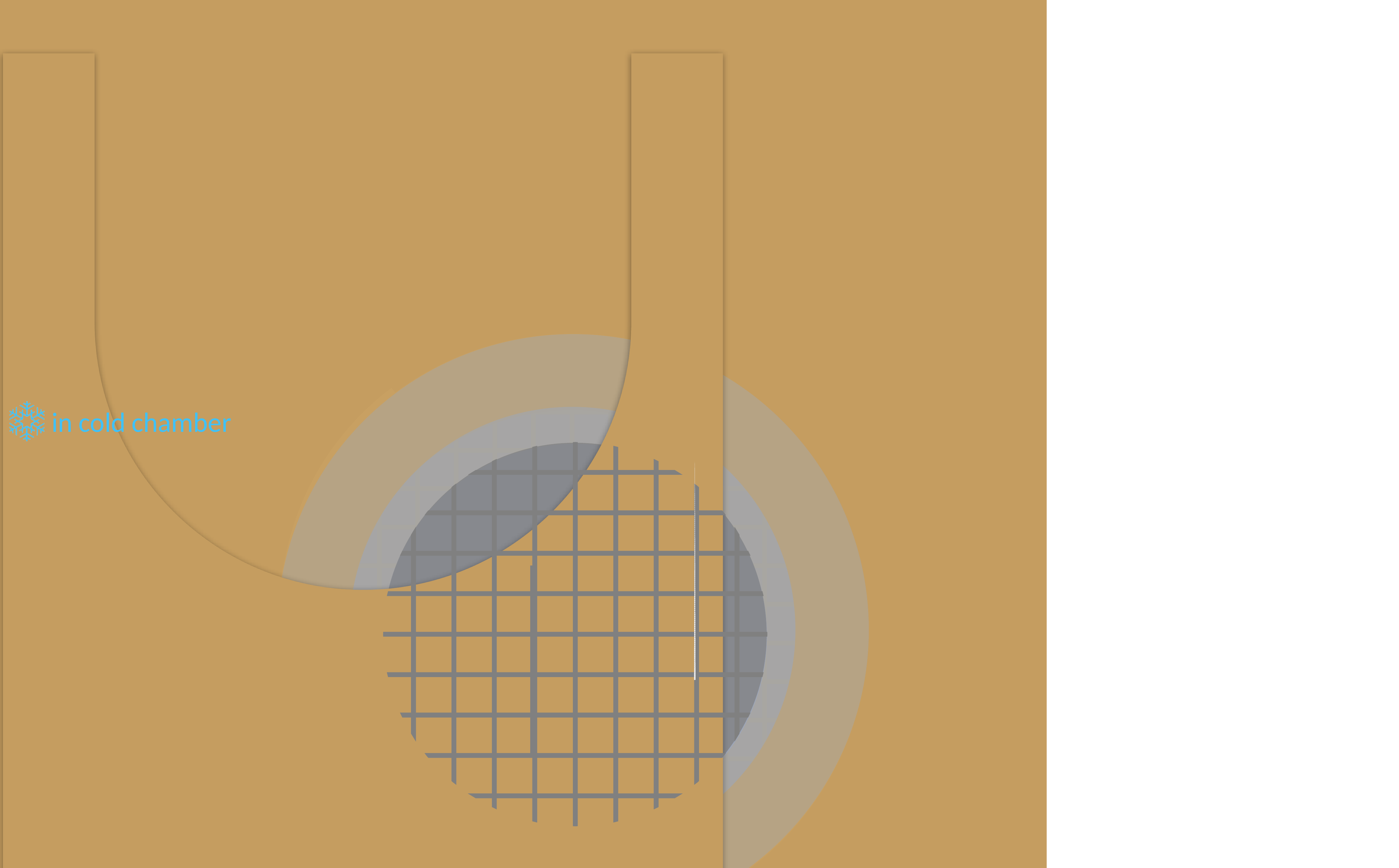

Step 2
Draw grid bar lines with FIB
in cold chamber

## Slide 94
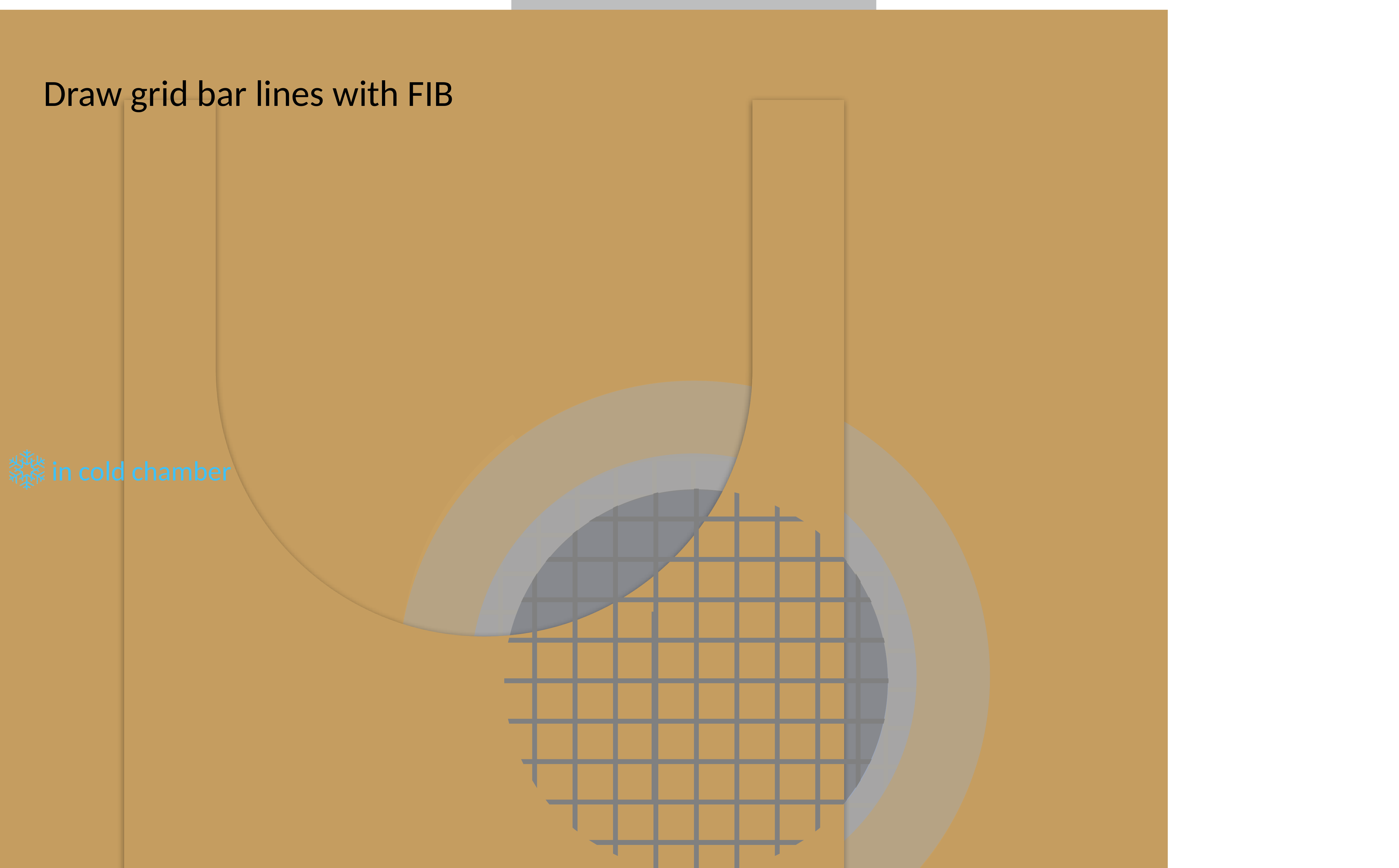

Step 2
Draw grid bar lines with FIB
in cold chamber

## Slide 95
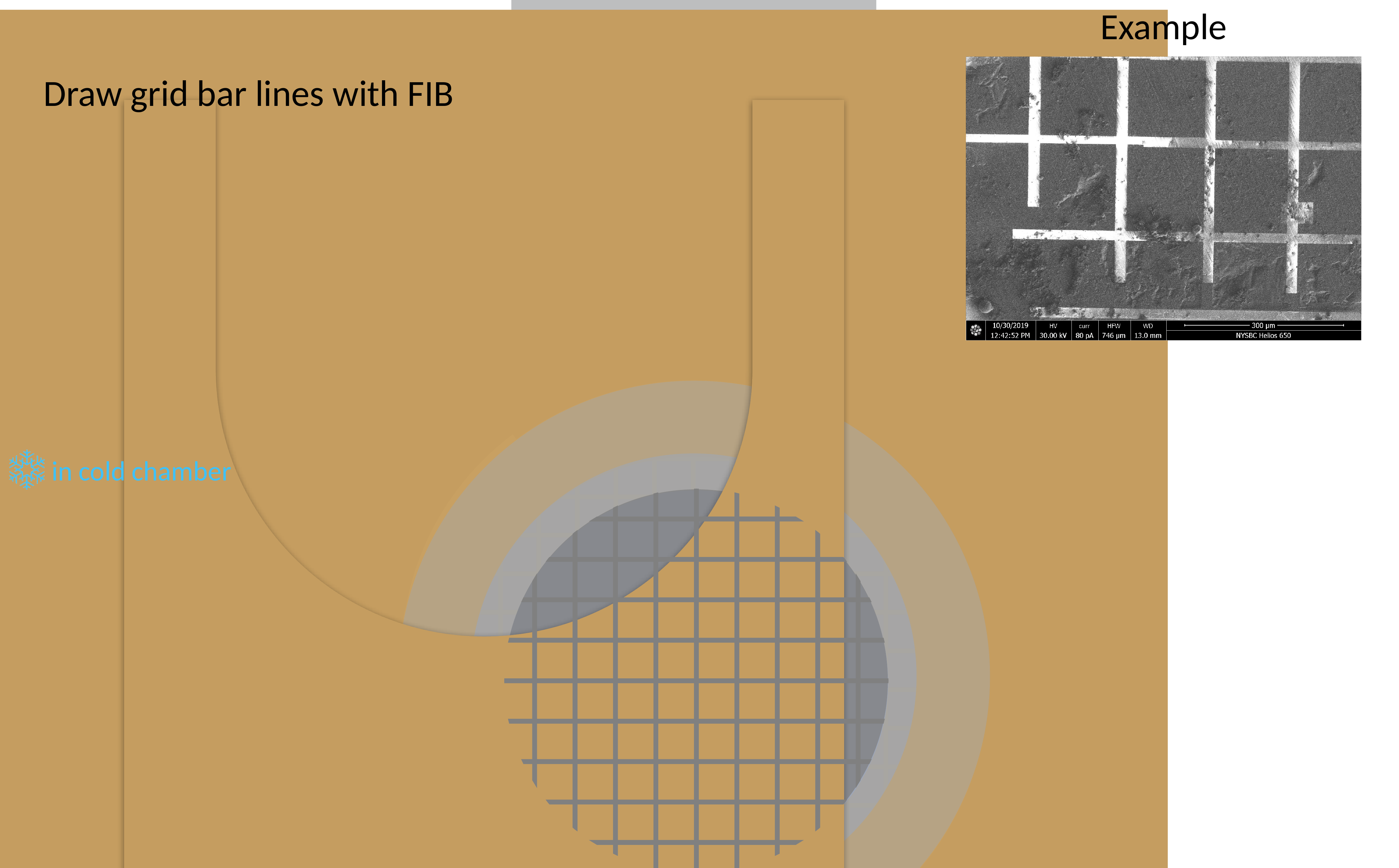

Example
Step 2
Draw grid bar lines with FIB
in cold chamber

## Slide 96
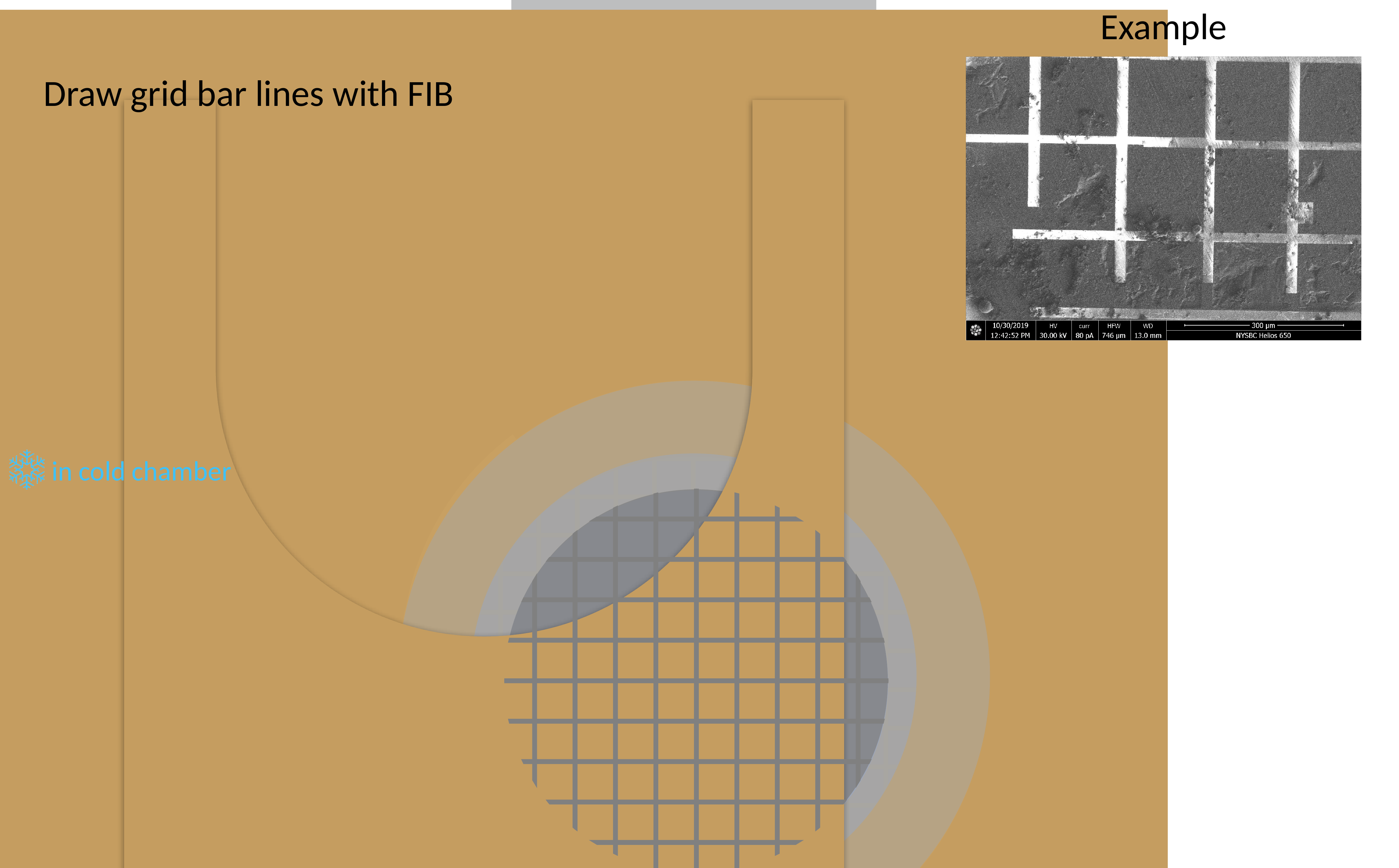

Example
Step 2
Draw grid bar lines with FIB
in cold chamber

## Slide 97
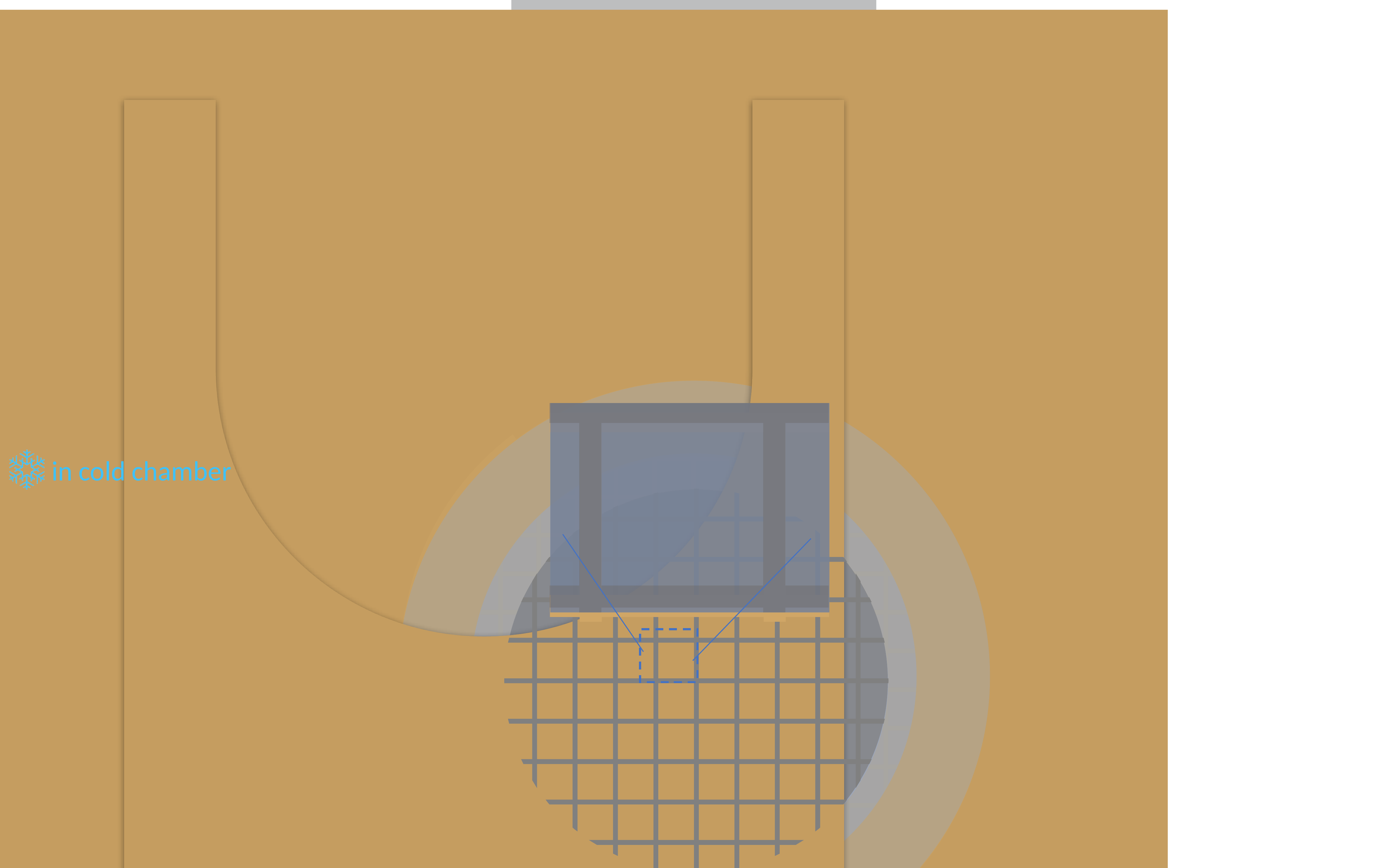

Step 2
FIB-mill trenches:
2 trenches per area of interest
(in flat holder)
in cold chamber

## Slide 98
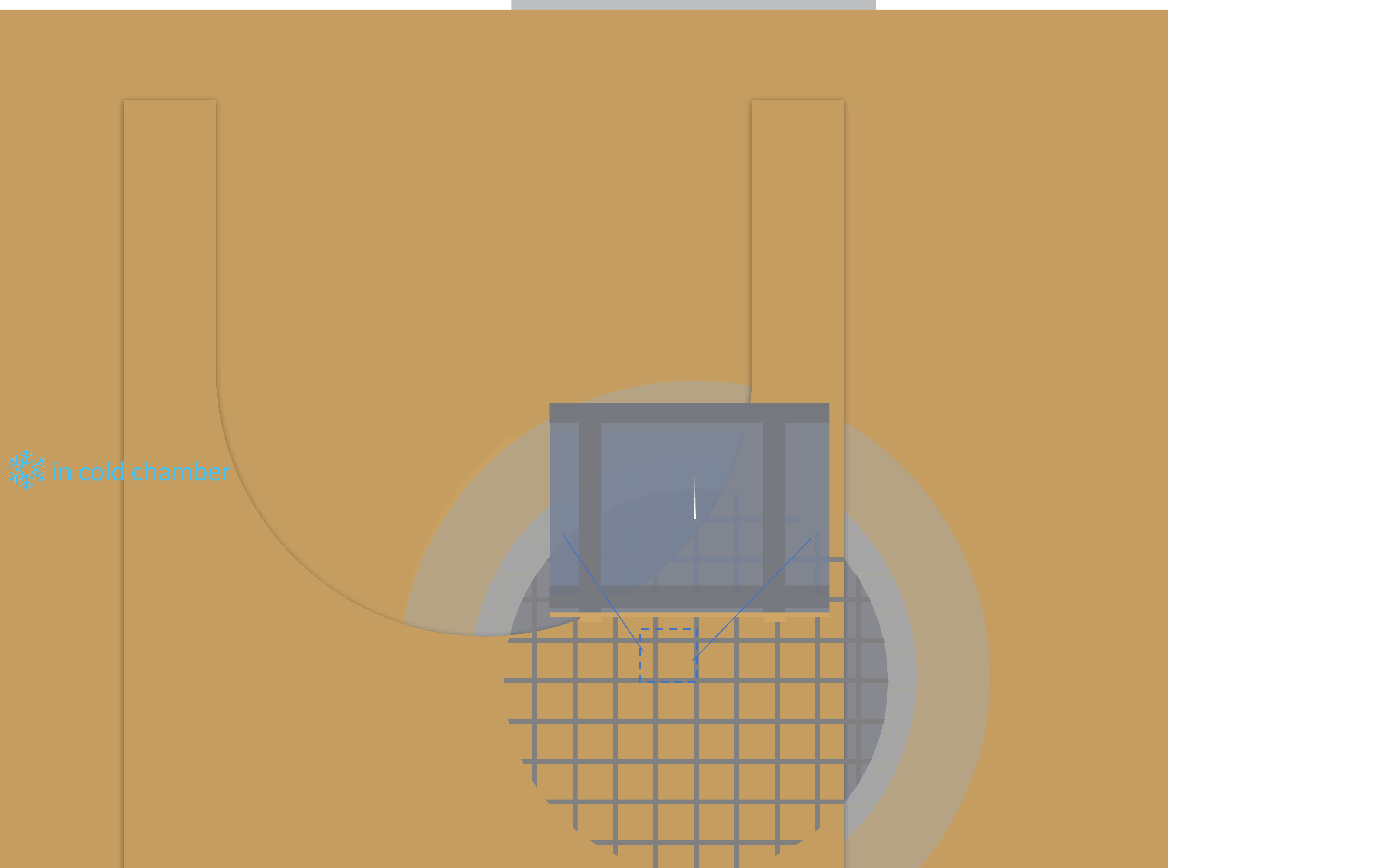

Step 2
FIB-mill trenches:
2 trenches per area of interest
(in flat holder)
in cold chamber

## Slide 99
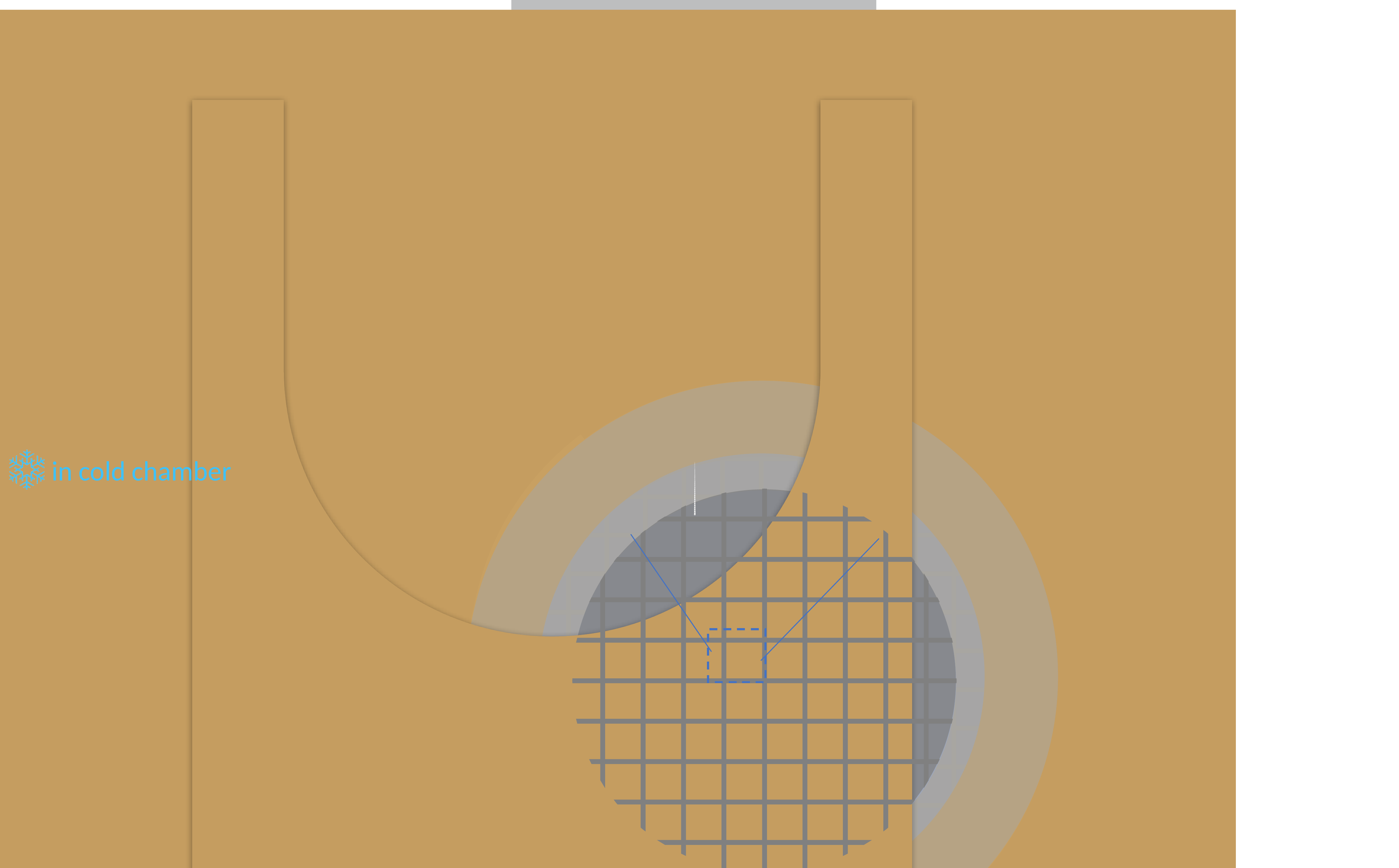

Step 2
FIB-mill trenches:
2 trenches per area of interest
(in flat holder)
in cold chamber

## Slide 100
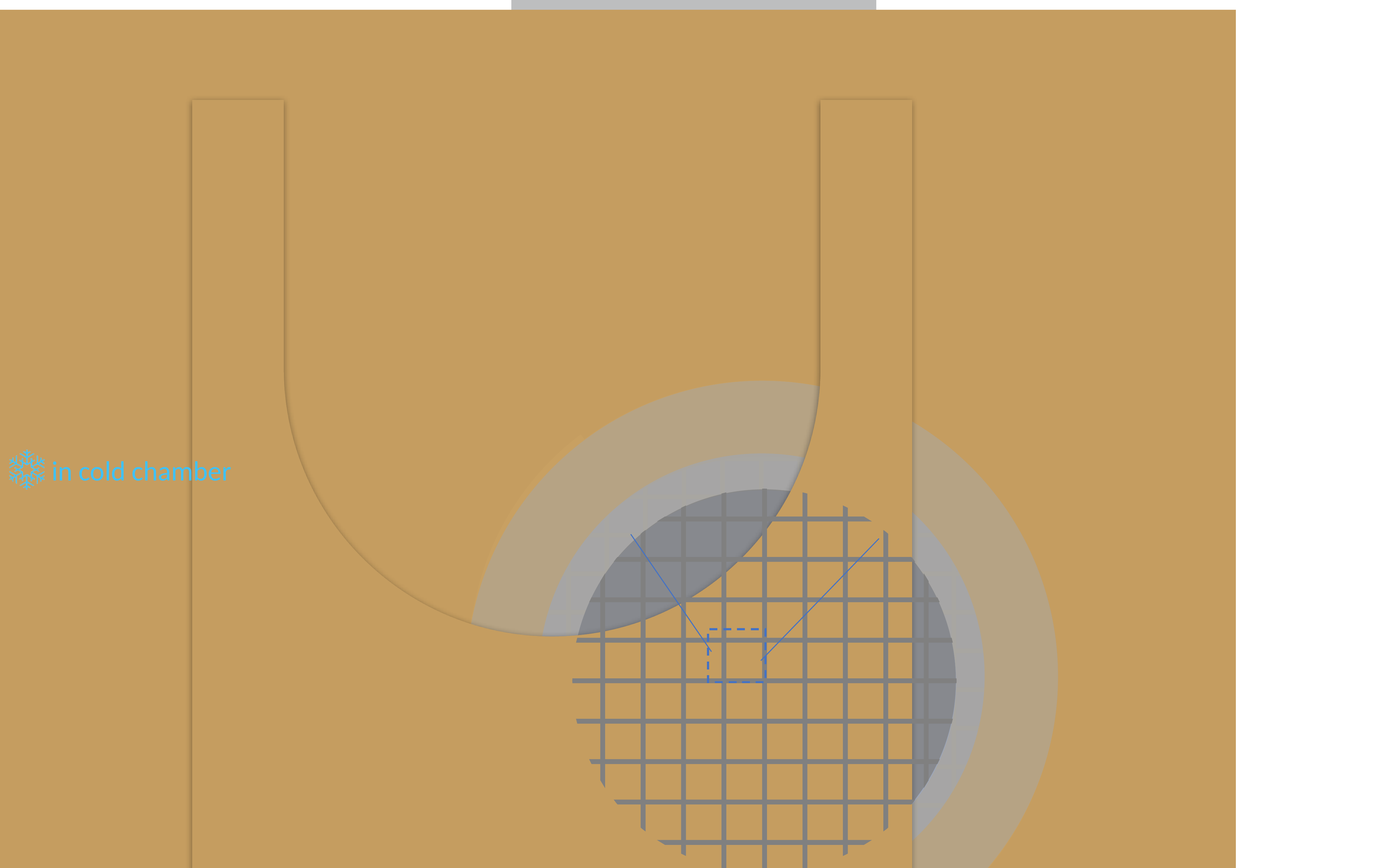

Step 2
FIB-mill trenches:
2 trenches per area of interest
(in flat holder)
in cold chamber

## Slide 101
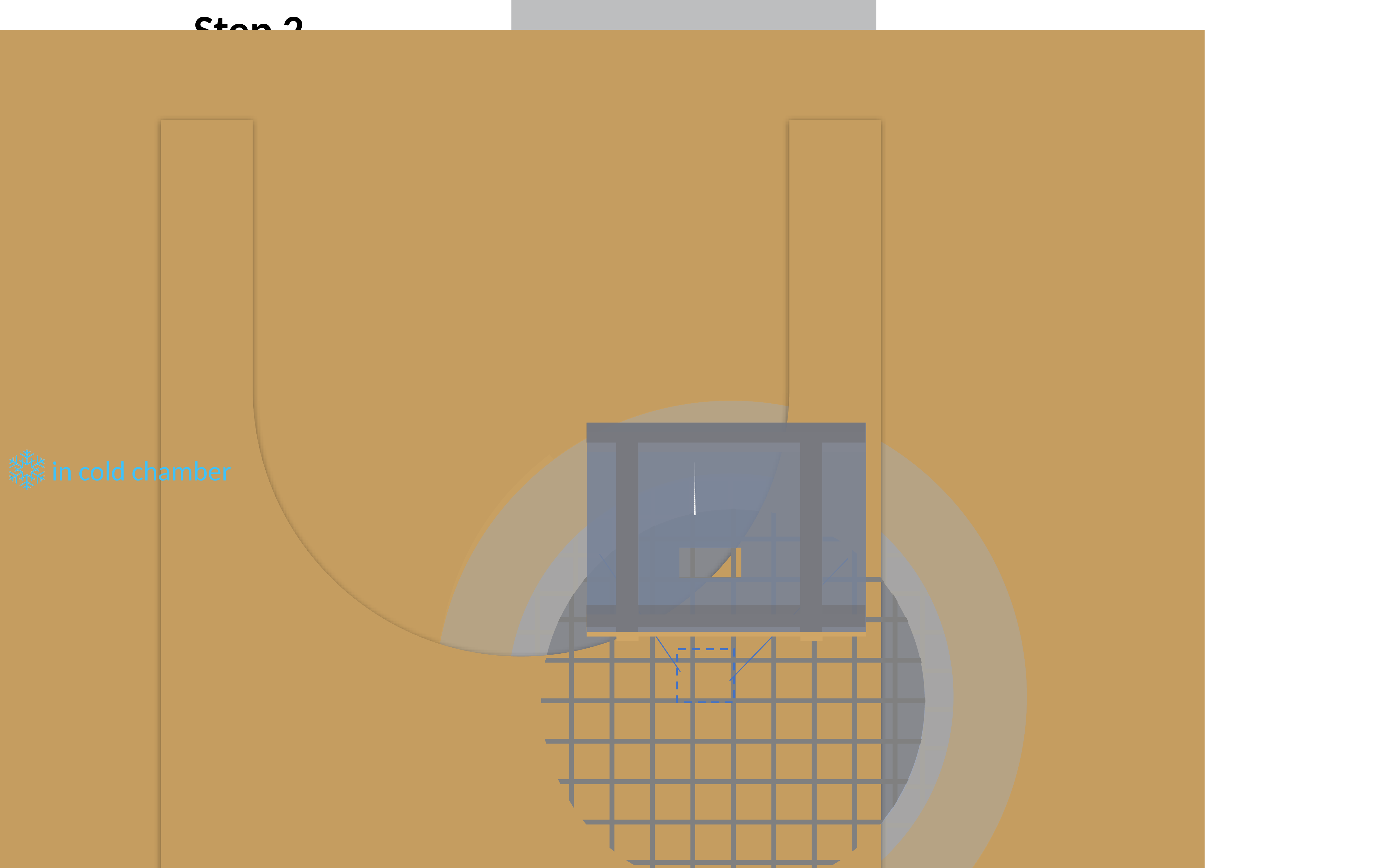

Step 2
FIB-mill trenches:
2 trenches per area of interest
(in flat holder)
in cold chamber

## Slide 102
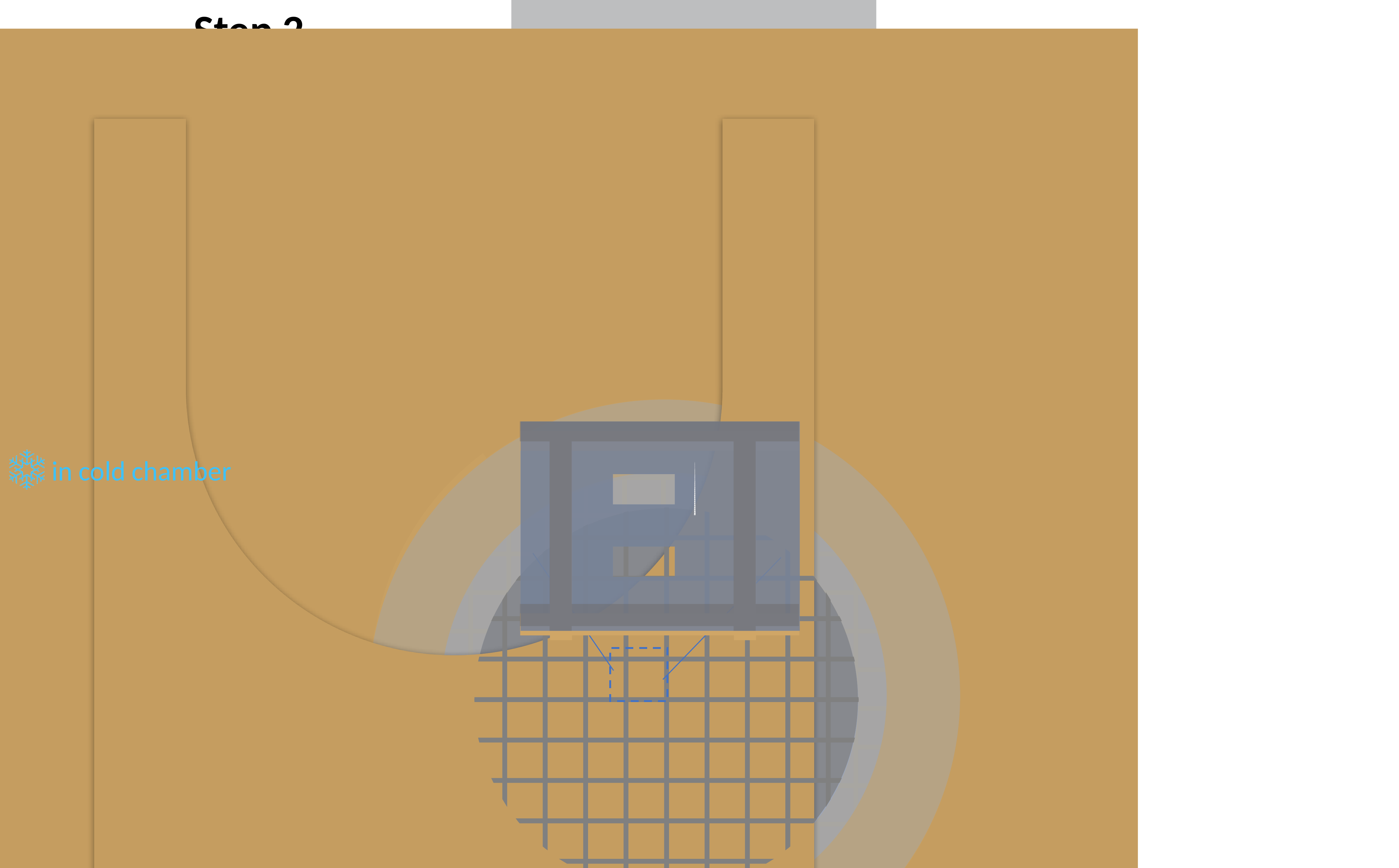

Step 2
FIB-mill trenches:
2 trenches per area of interest
(in flat holder)
in cold chamber

## Slide 103
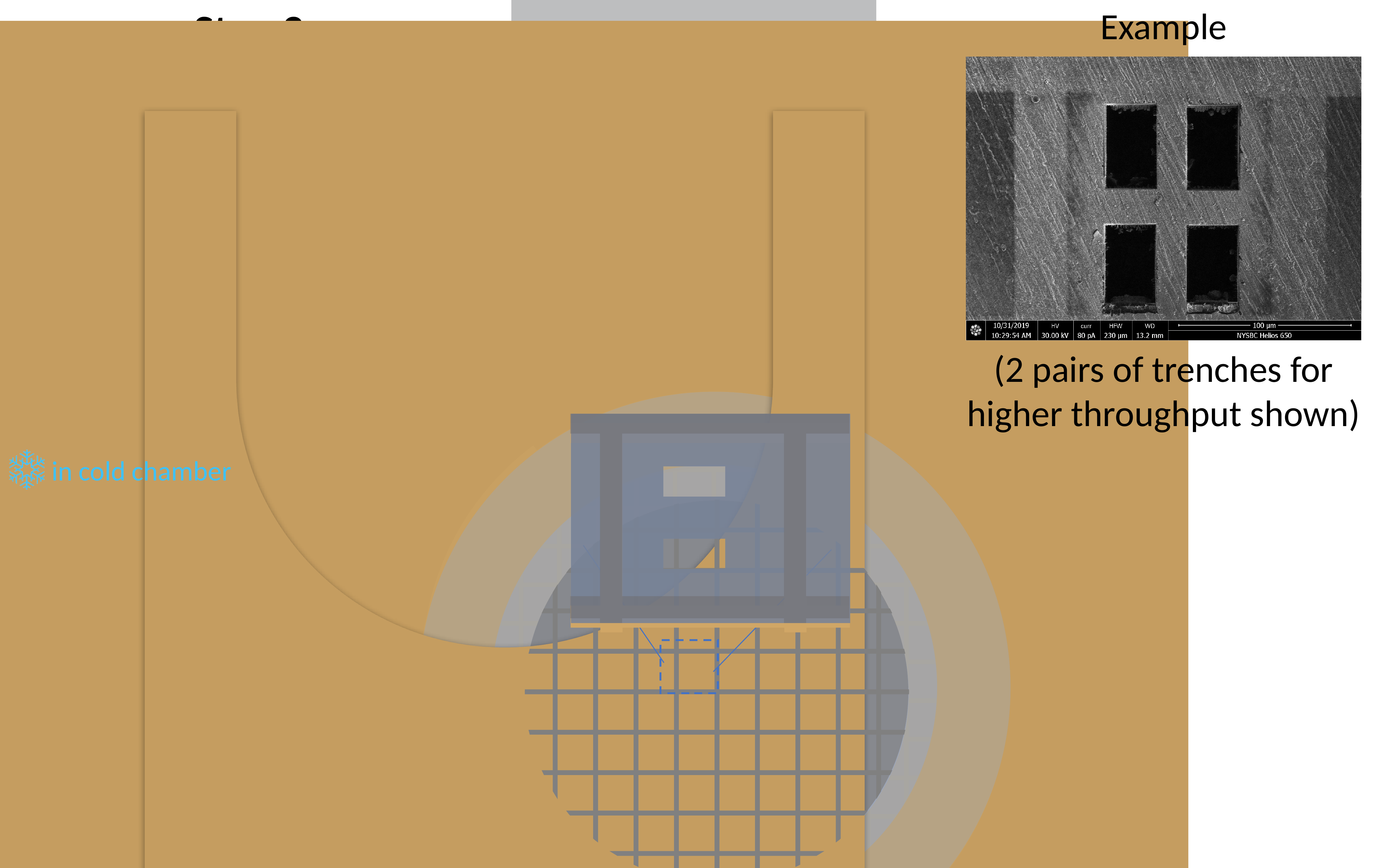

Example
Step 2
FIB-mill trenches:
2 trenches per area of interest
(in flat holder)
(2 pairs of trenches for
higher throughput shown)
in cold chamber

## Slide 104
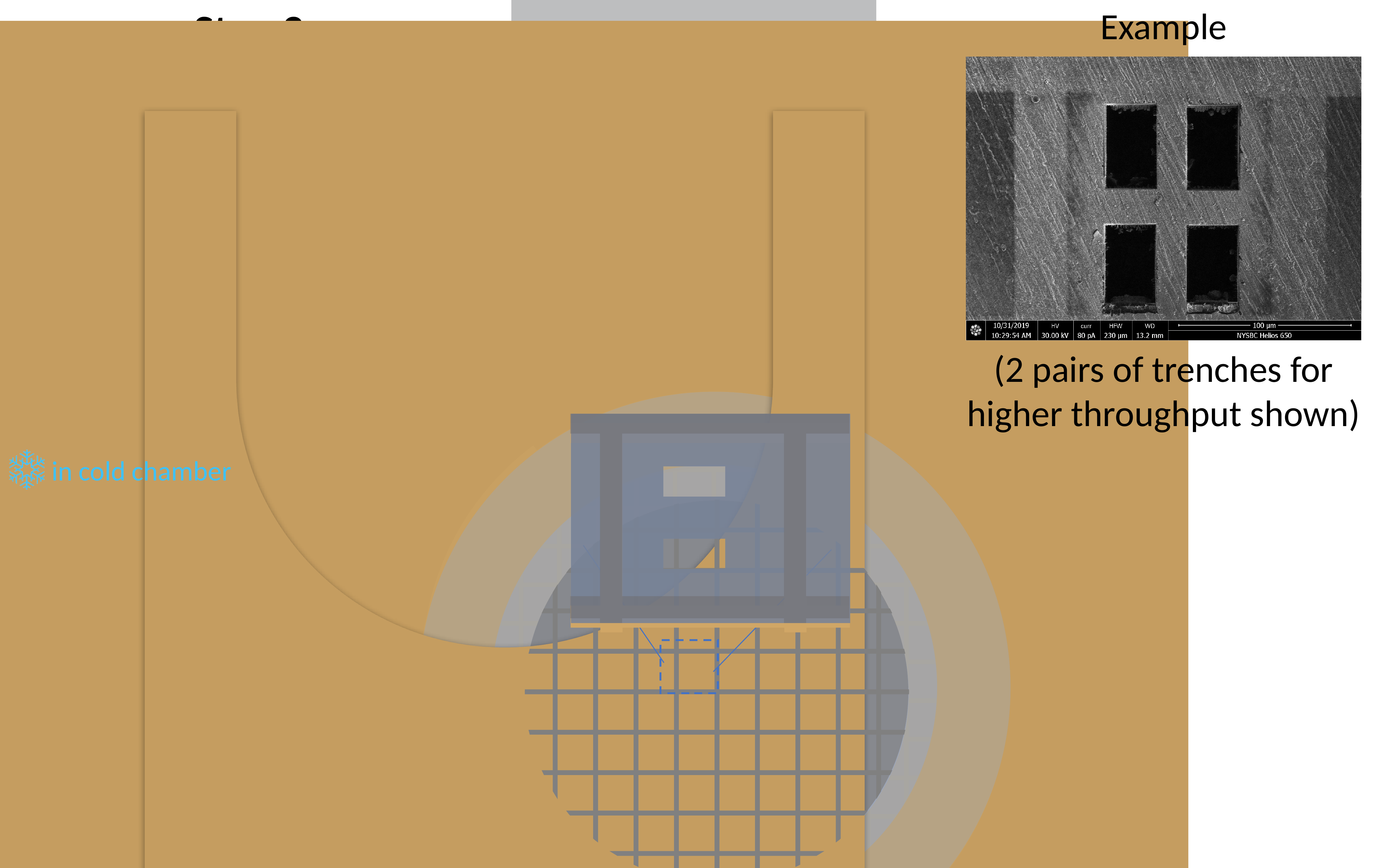

Example
Step 2
FIB-mill trenches:
2 trenches per area of interest
(in flat holder)
(2 pairs of trenches for
higher throughput shown)
in cold chamber

## Slide 105
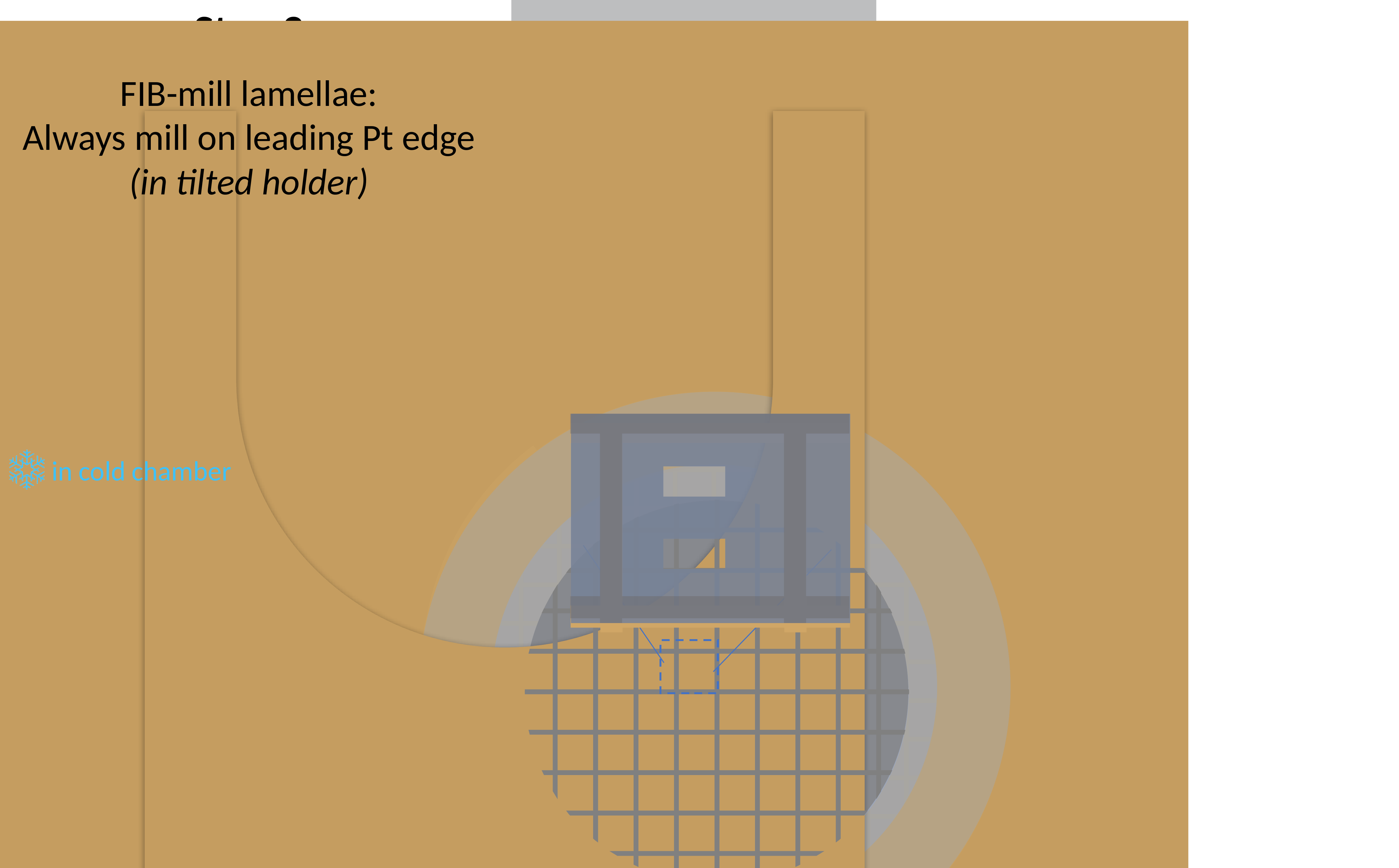

Step 2
FIB-mill lamellae:
Always mill on leading Pt edge
(in tilted holder)
in cold chamber

## Slide 106
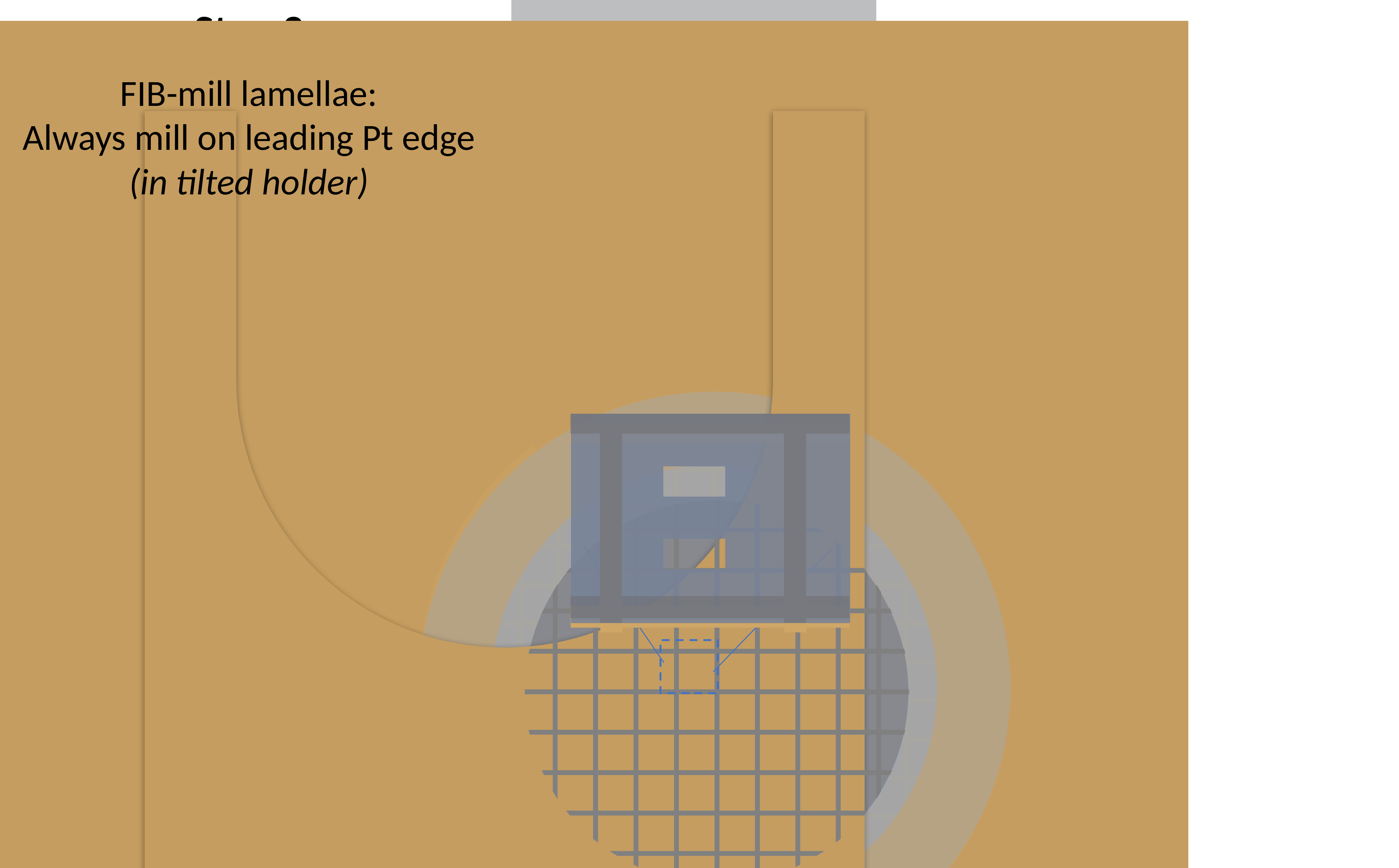

Step 2
FIB-mill lamellae:
Always mill on leading Pt edge
(in tilted holder)
in cold chamber

## Slide 107
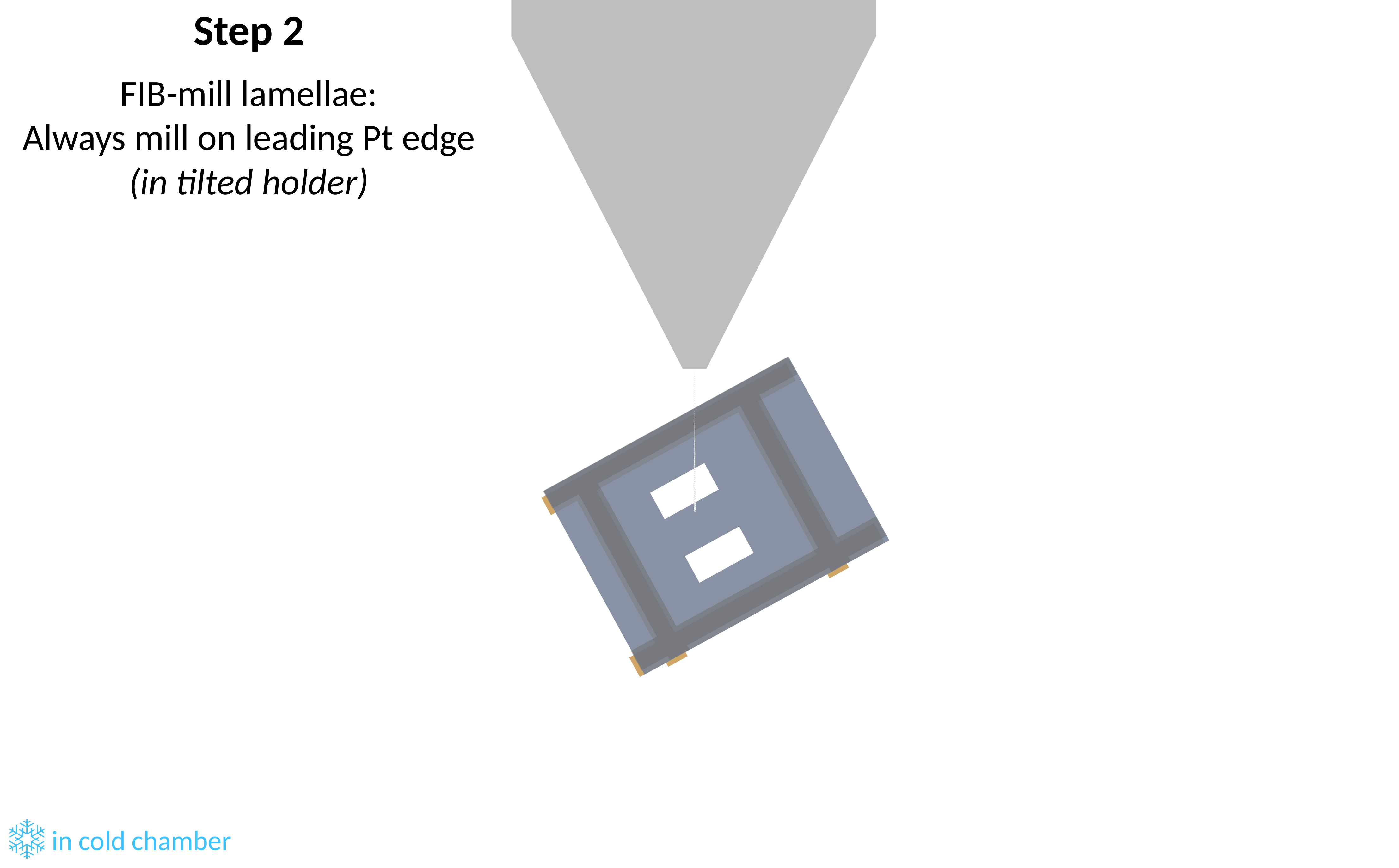

Step 2
FIB-mill lamellae:
Always mill on leading Pt edge
(in tilted holder)
in cold chamber

## Slide 108
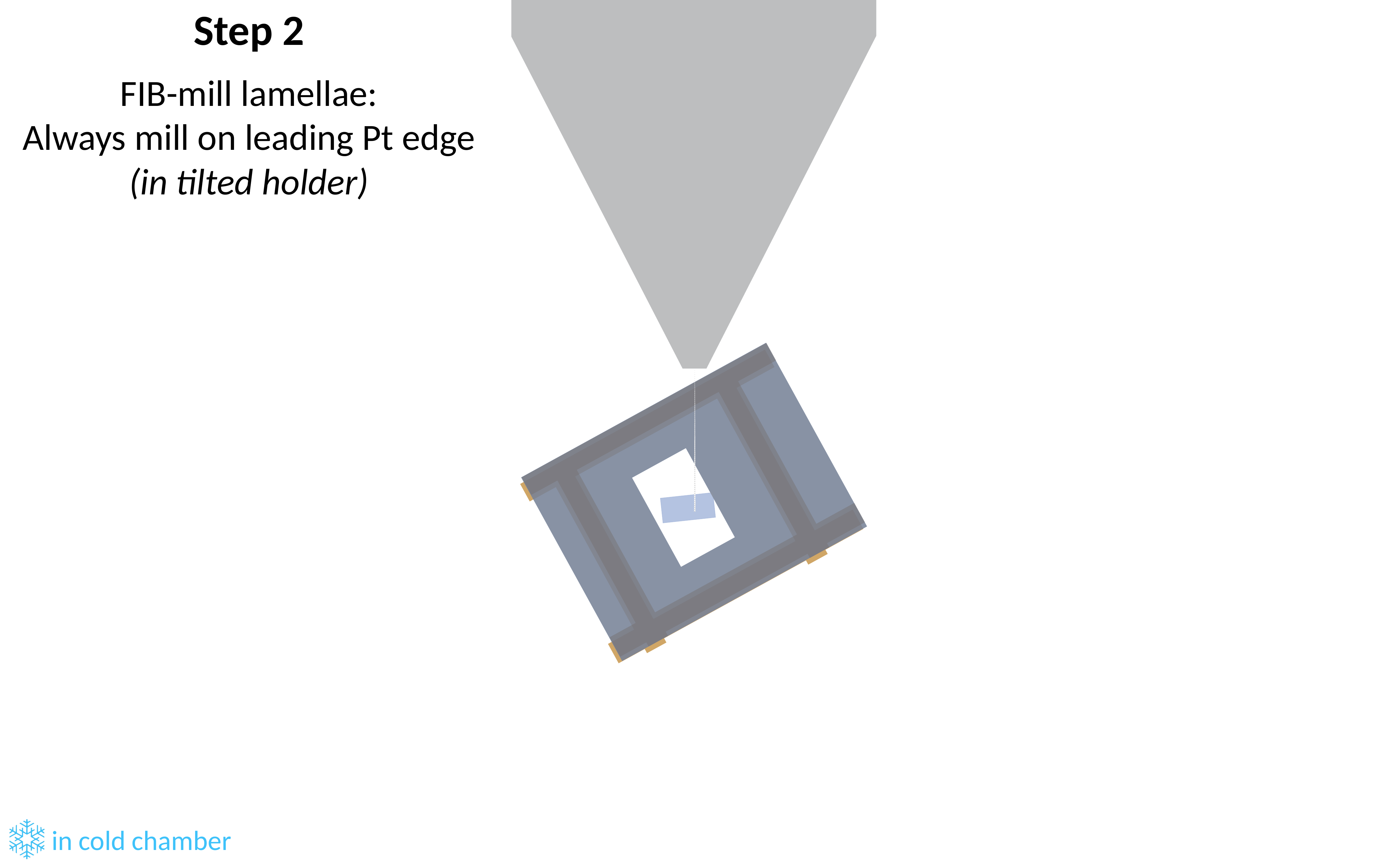

Step 2
FIB-mill lamellae:
Always mill on leading Pt edge
(in tilted holder)
in cold chamber

## Slide 109
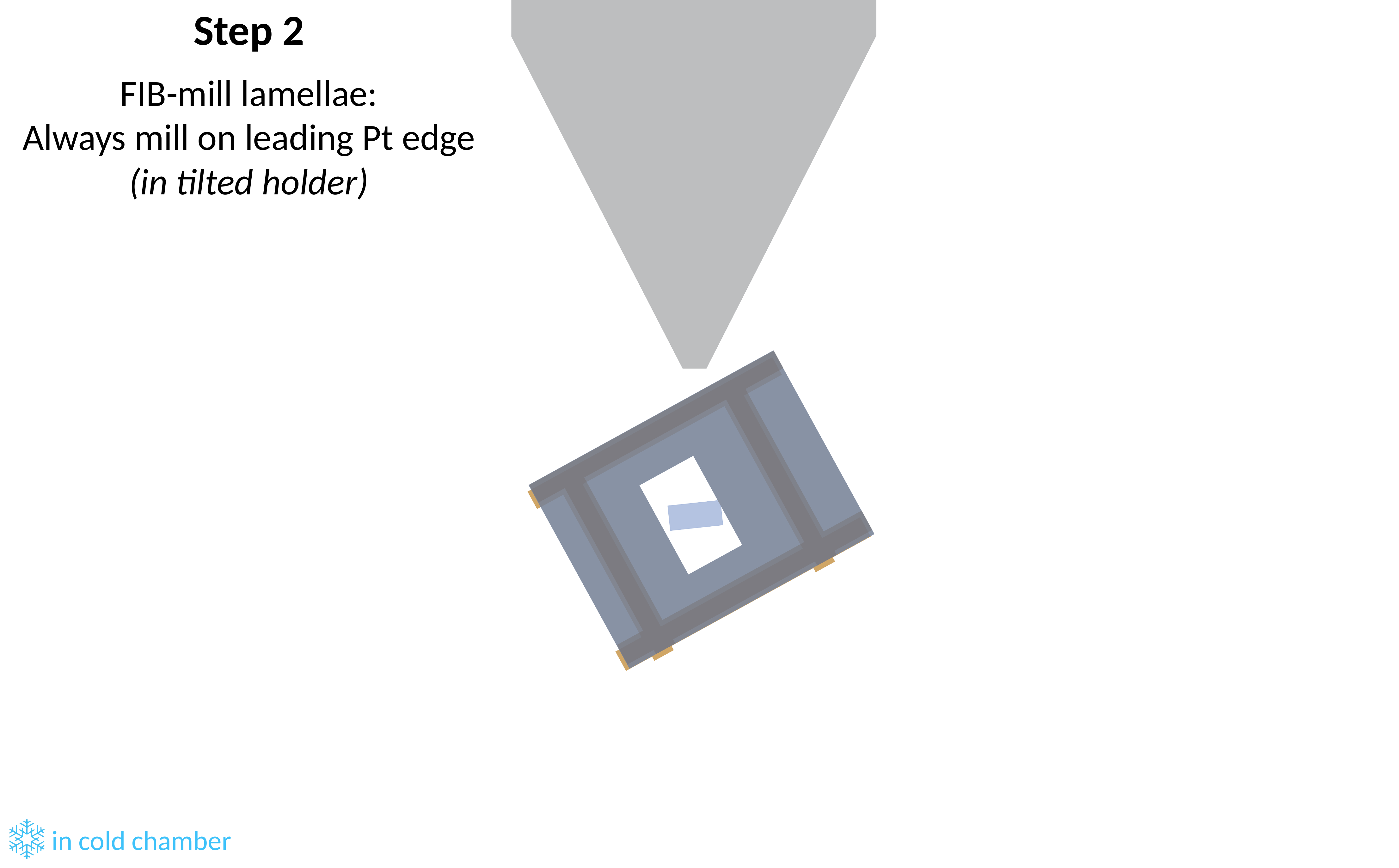

Step 2
FIB-mill lamellae:
Always mill on leading Pt edge
(in tilted holder)
in cold chamber

## Slide 110
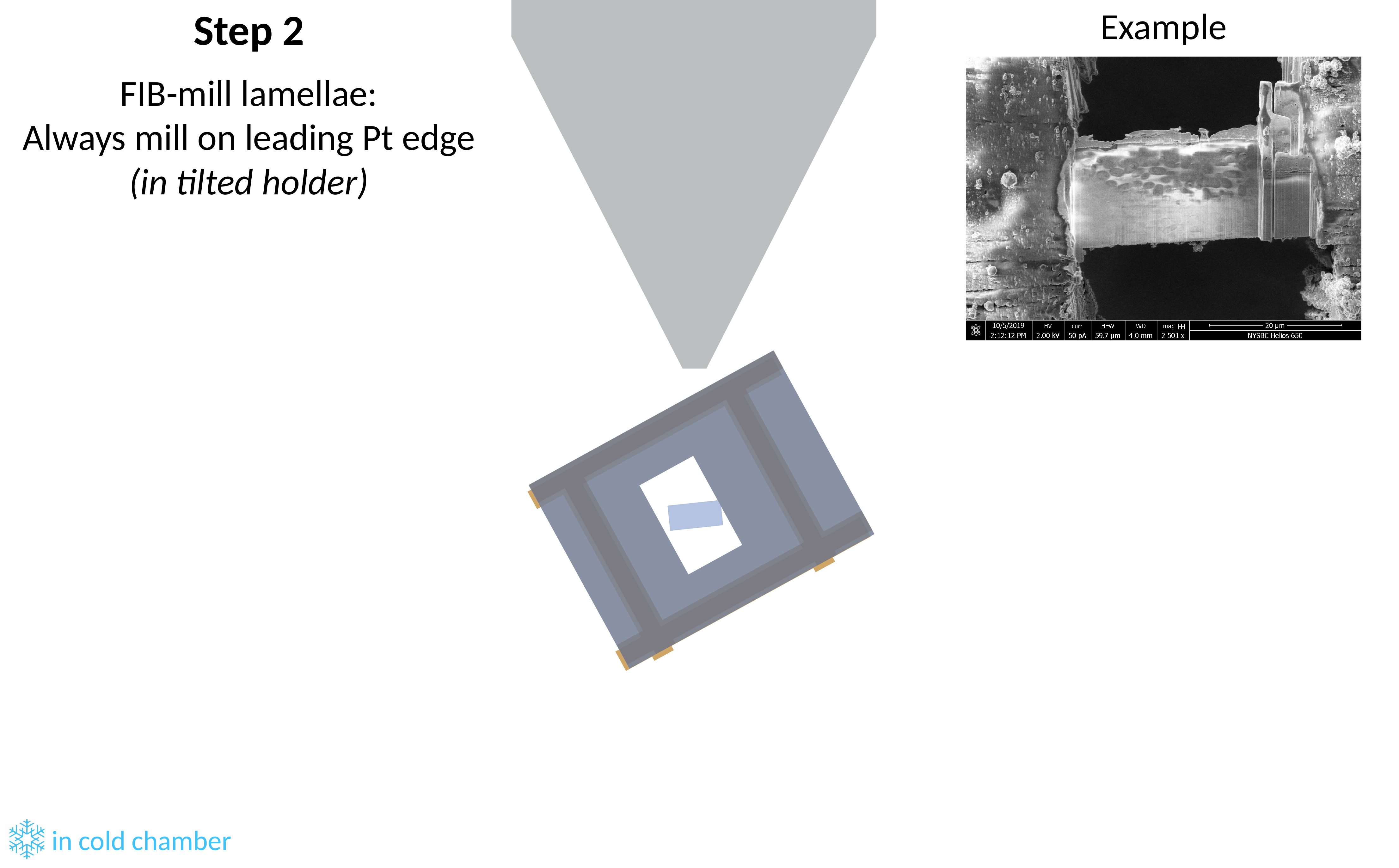

Example
Step 2
FIB-mill lamellae:
Always mill on leading Pt edge
(in tilted holder)
in cold chamber

## Slide 111
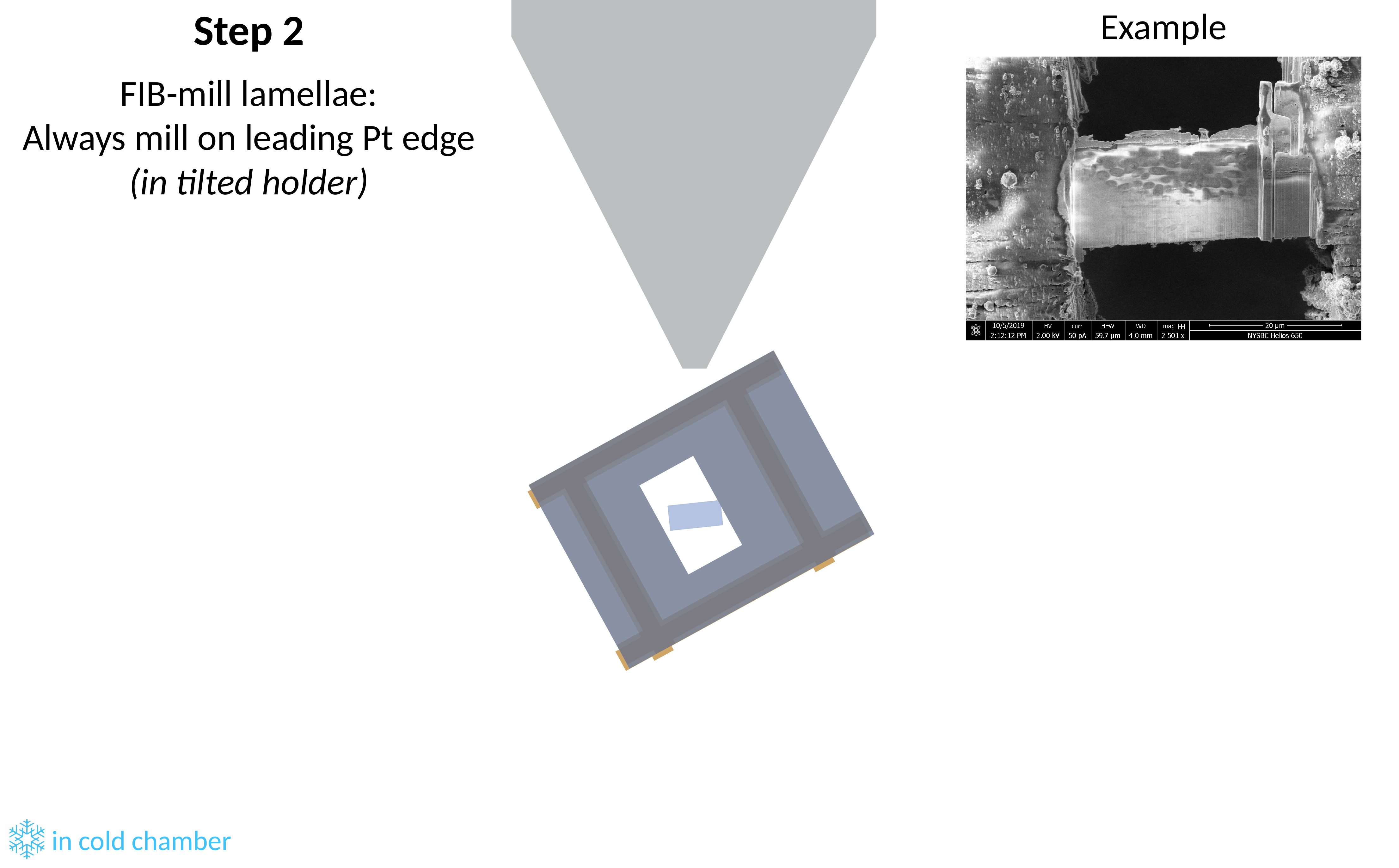

Example
Step 2
FIB-mill lamellae:
Always mill on leading Pt edge
(in tilted holder)
in cold chamber

## Slide 112
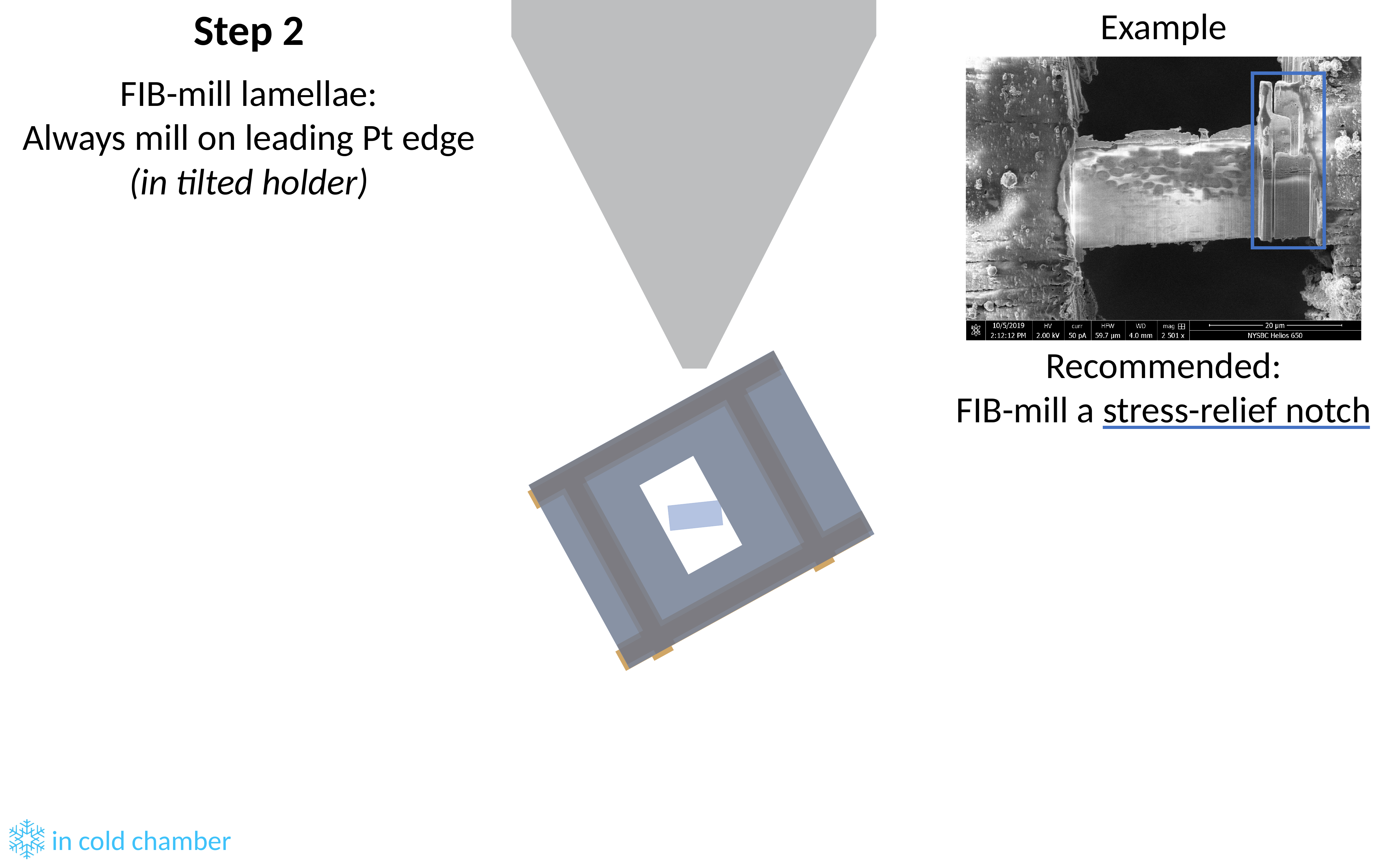

Example
Step 2
FIB-mill lamellae:
Always mill on leading Pt edge
(in tilted holder)
Recommended:
FIB-mill a stress-relief notch
in cold chamber

## Slide 113
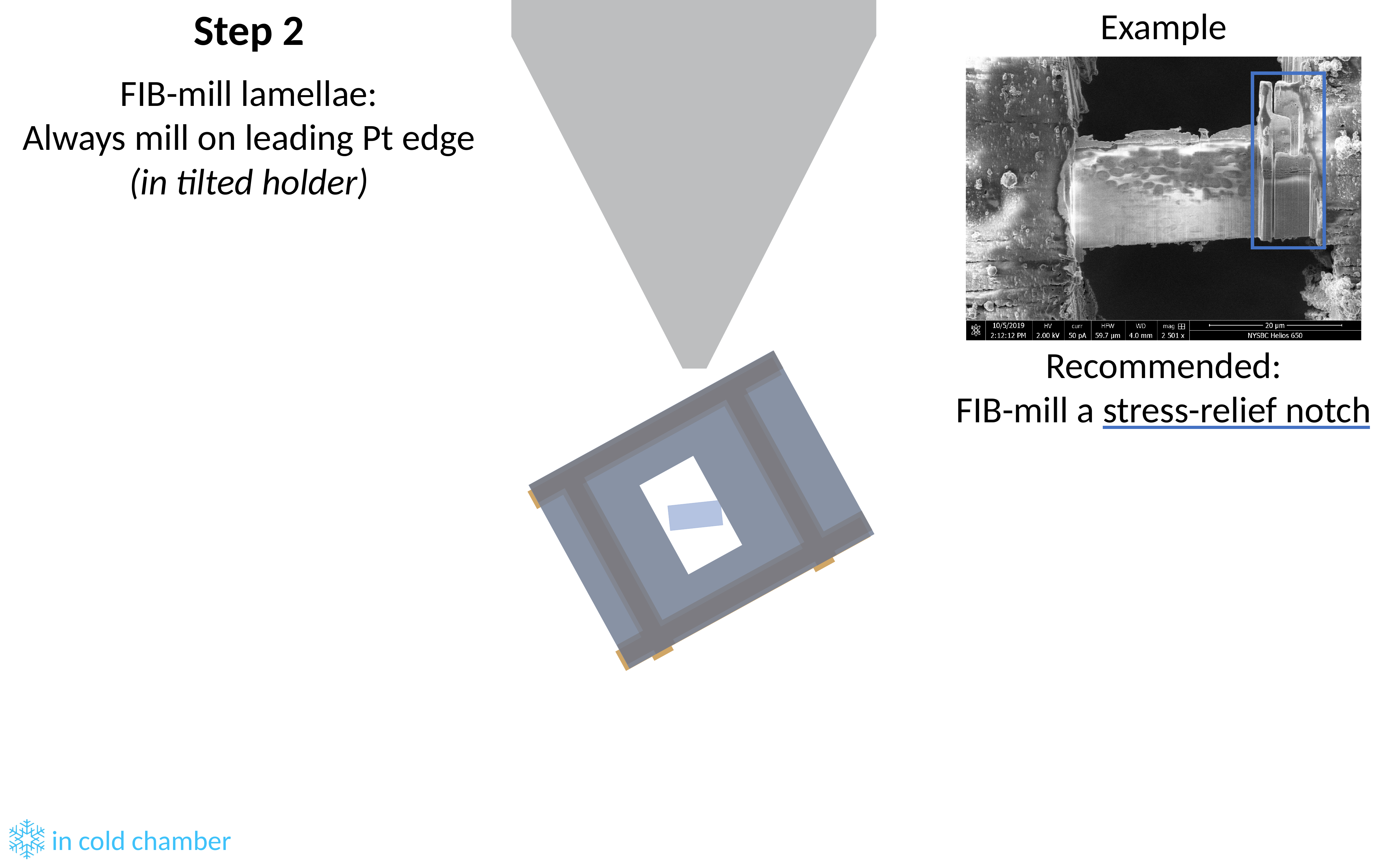

Example
Step 2
FIB-mill lamellae:
Always mill on leading Pt edge
(in tilted holder)
Recommended:
FIB-mill a stress-relief notch
in cold chamber

## Slide 114
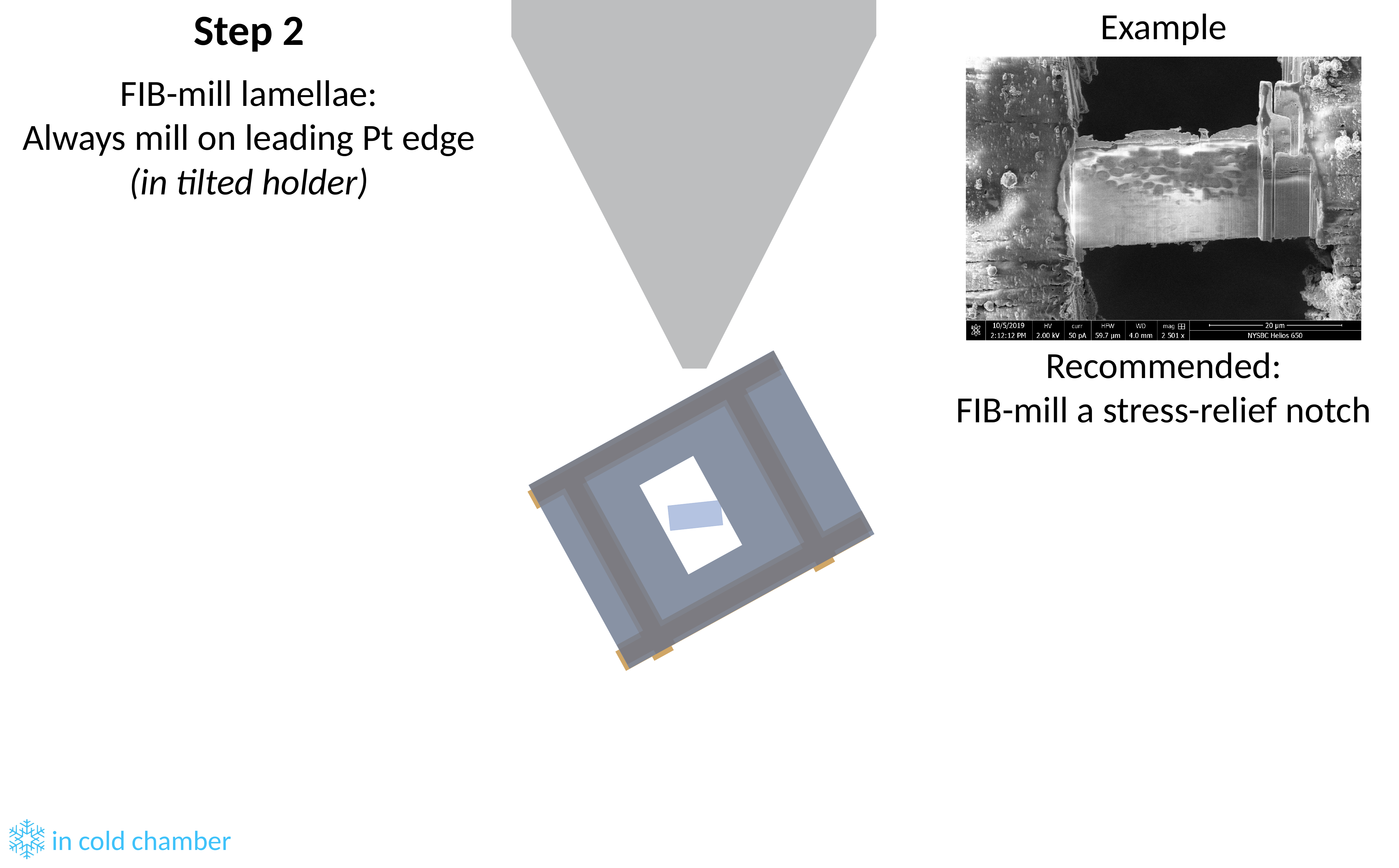

Example
Step 2
FIB-mill lamellae:
Always mill on leading Pt edge
(in tilted holder)
Recommended:
FIB-mill a stress-relief notch
in cold chamber

## Slide 115
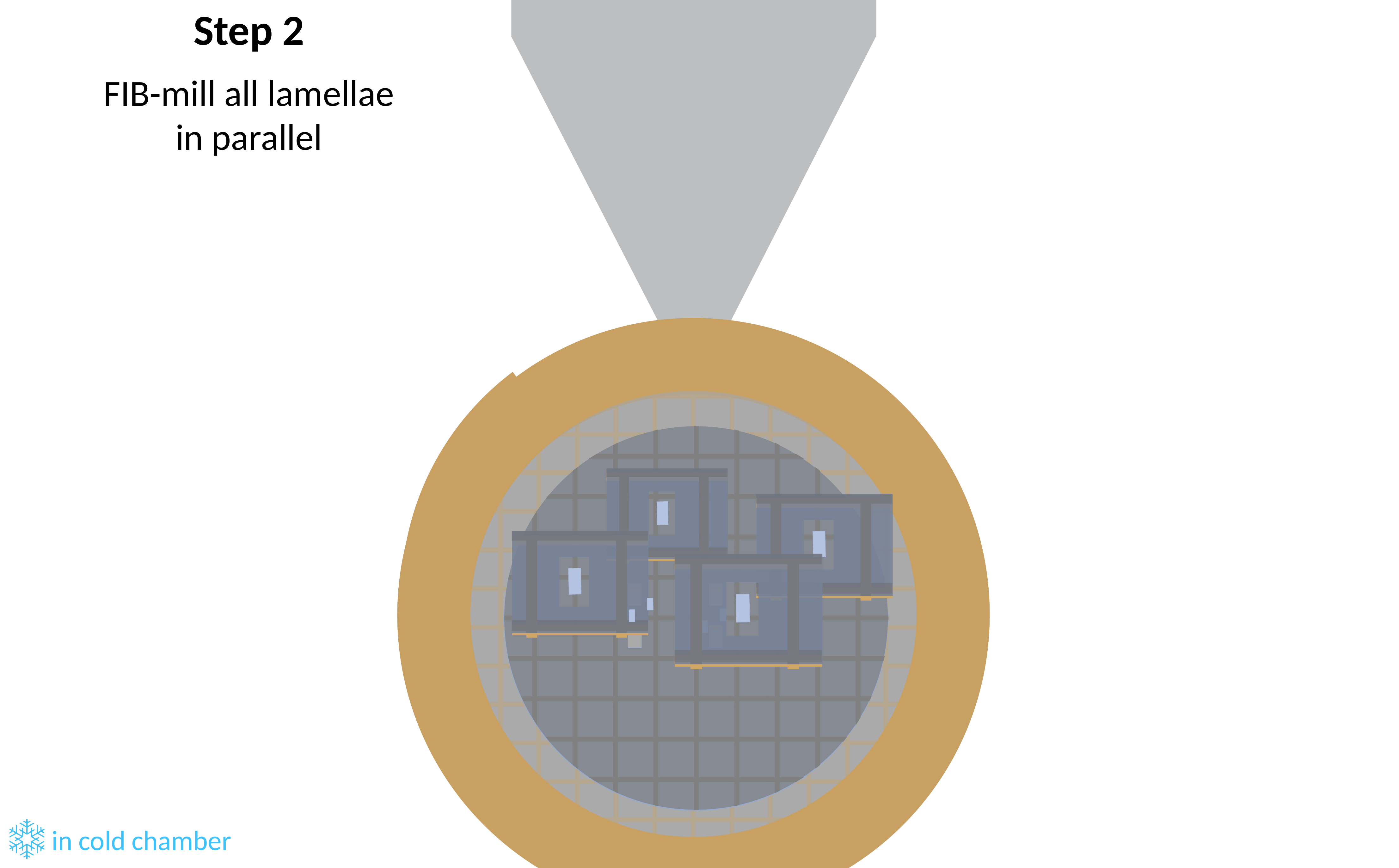

Step 2
FIB-mill all lamellae
in parallel
in cold chamber

## Slide 116
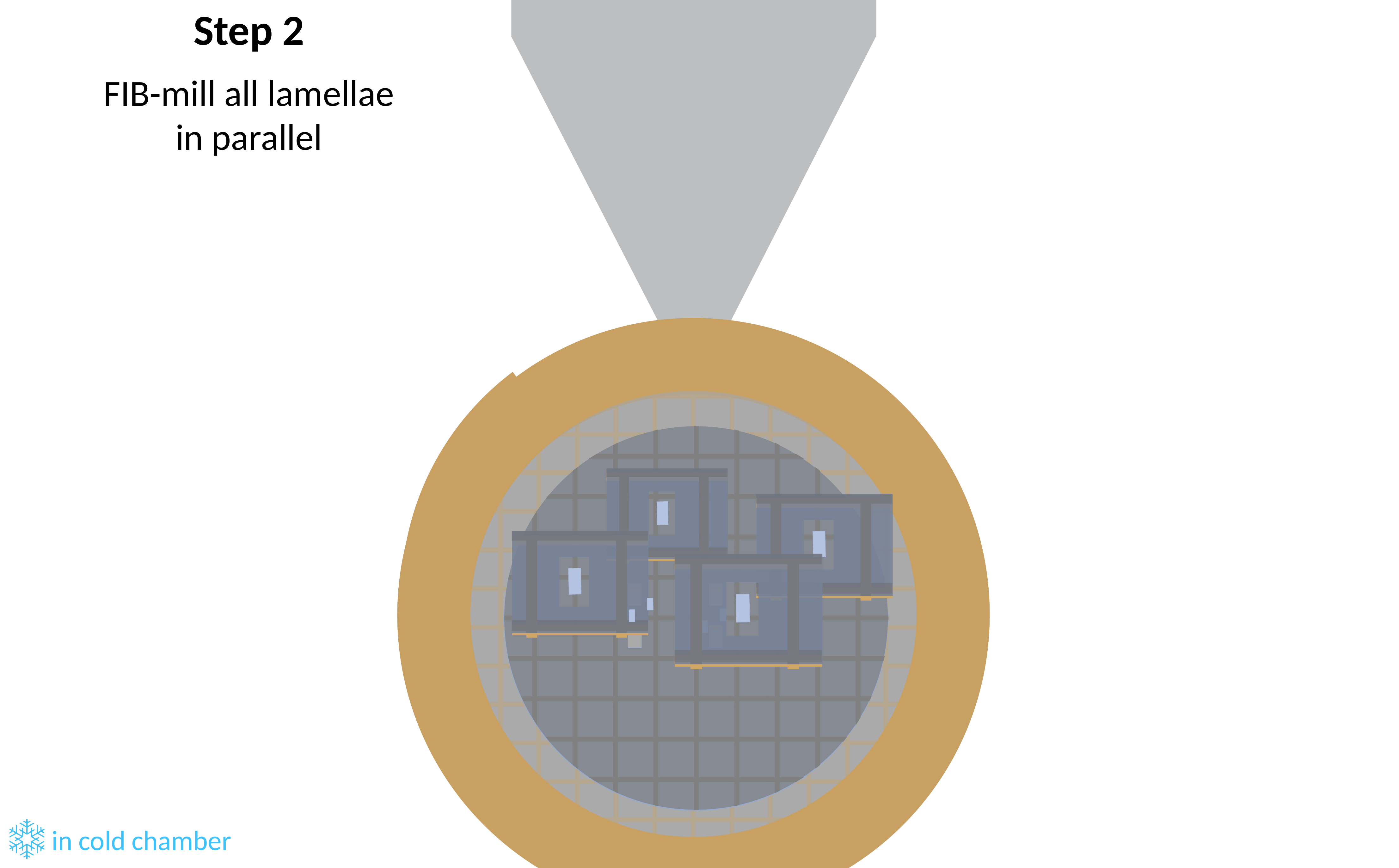

Step 2
FIB-mill all lamellae
in parallel
in cold chamber

## Slide 117
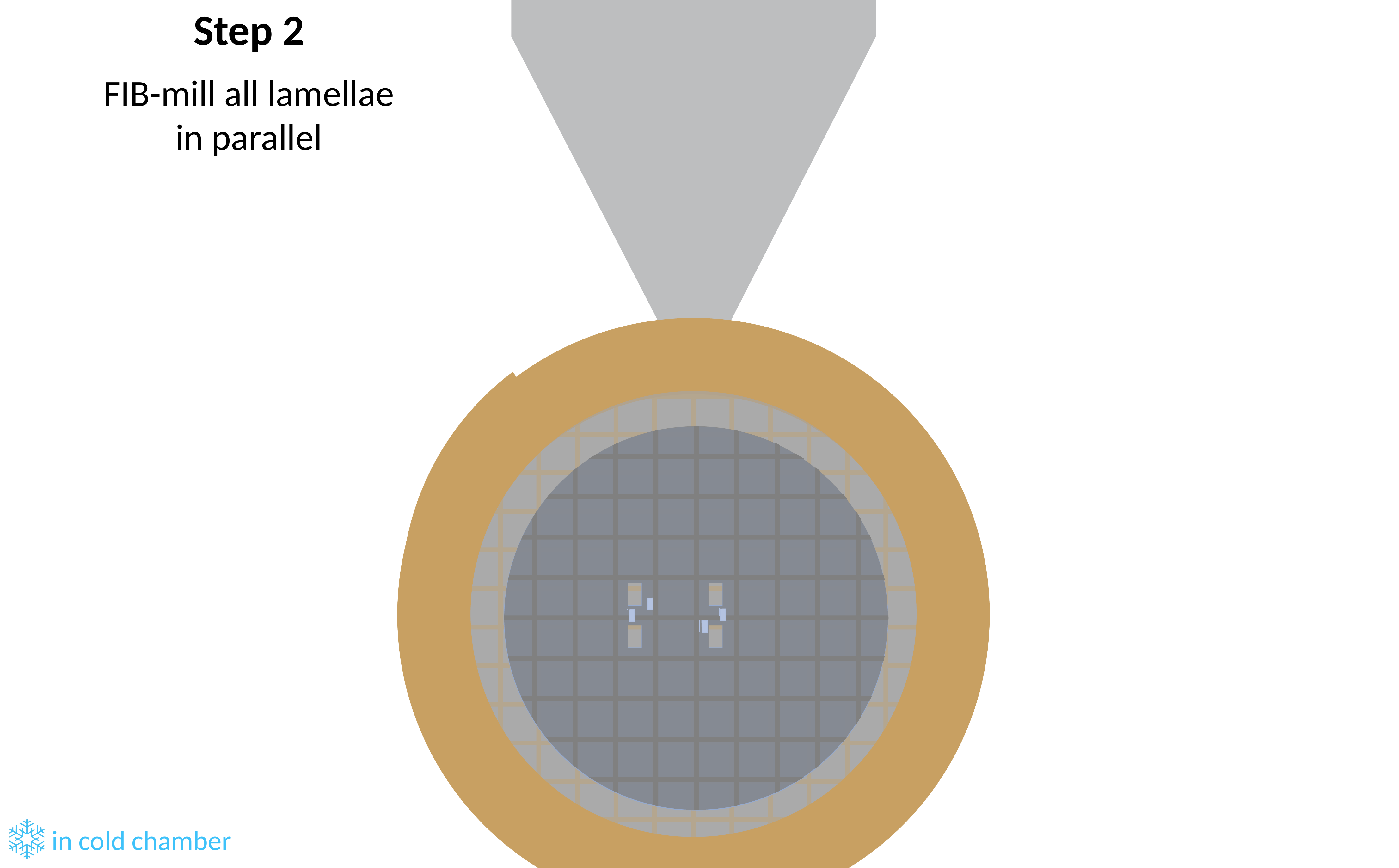

Step 2
FIB-mill all lamellae
in parallel
in cold chamber

## Slide 118
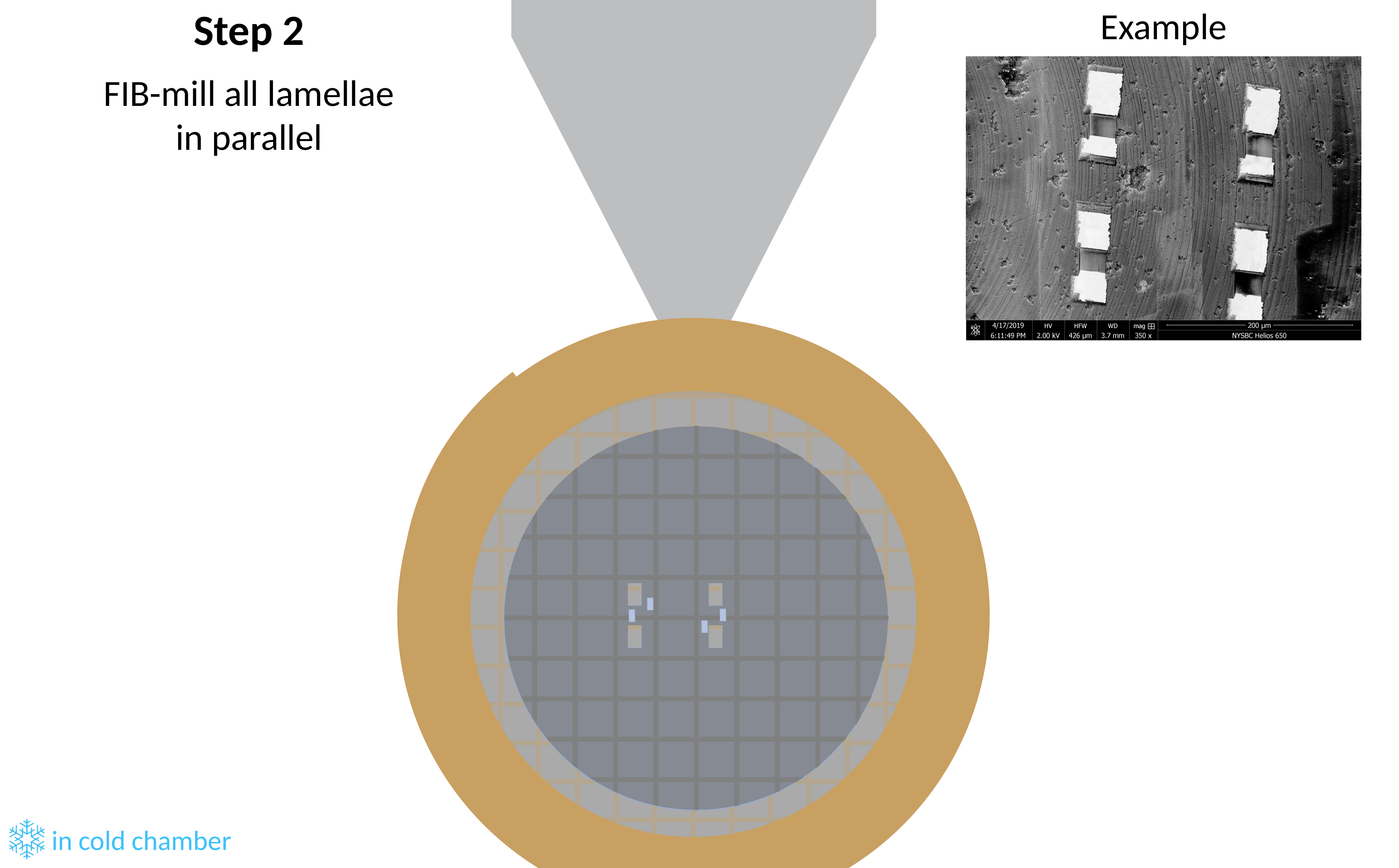

Example
Step 2
FIB-mill all lamellae
in parallel
in cold chamber

## Slide 119
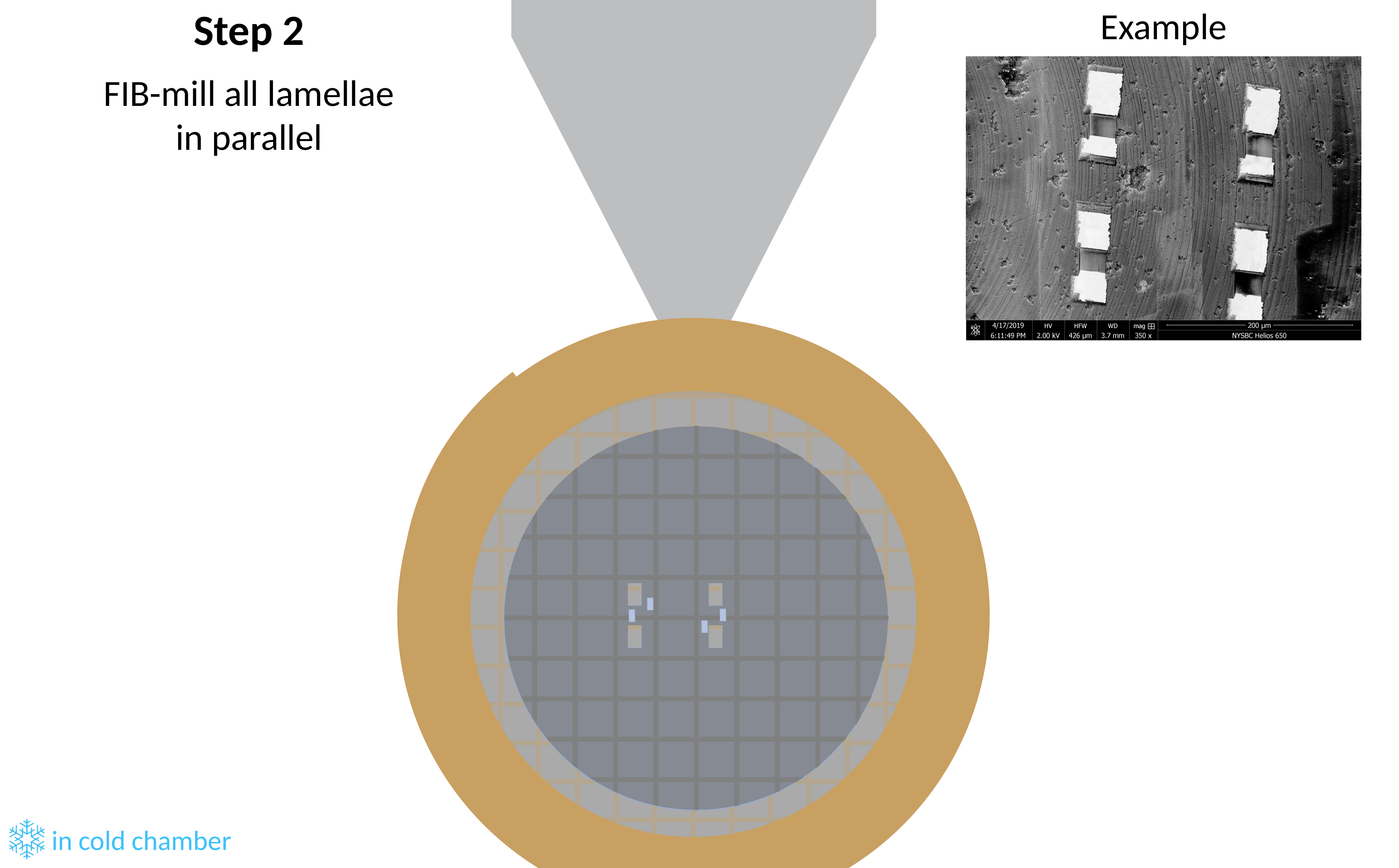

Example
Step 2
FIB-mill all lamellae
in parallel
in cold chamber

## Slide 120
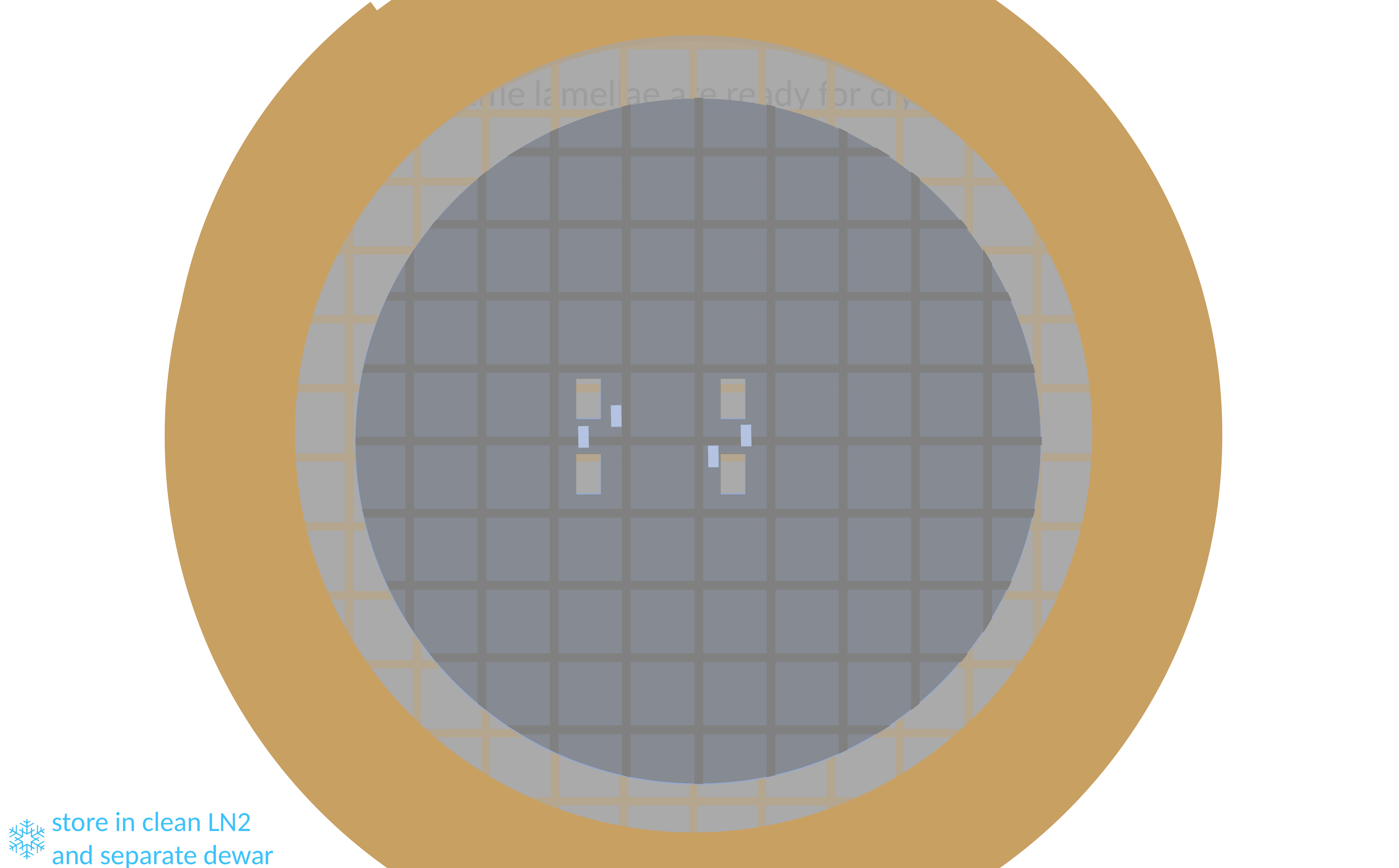

Finished!
Waffle lamellae are ready for cryoET
store in clean LN2 and separate dewar

## Slide 121
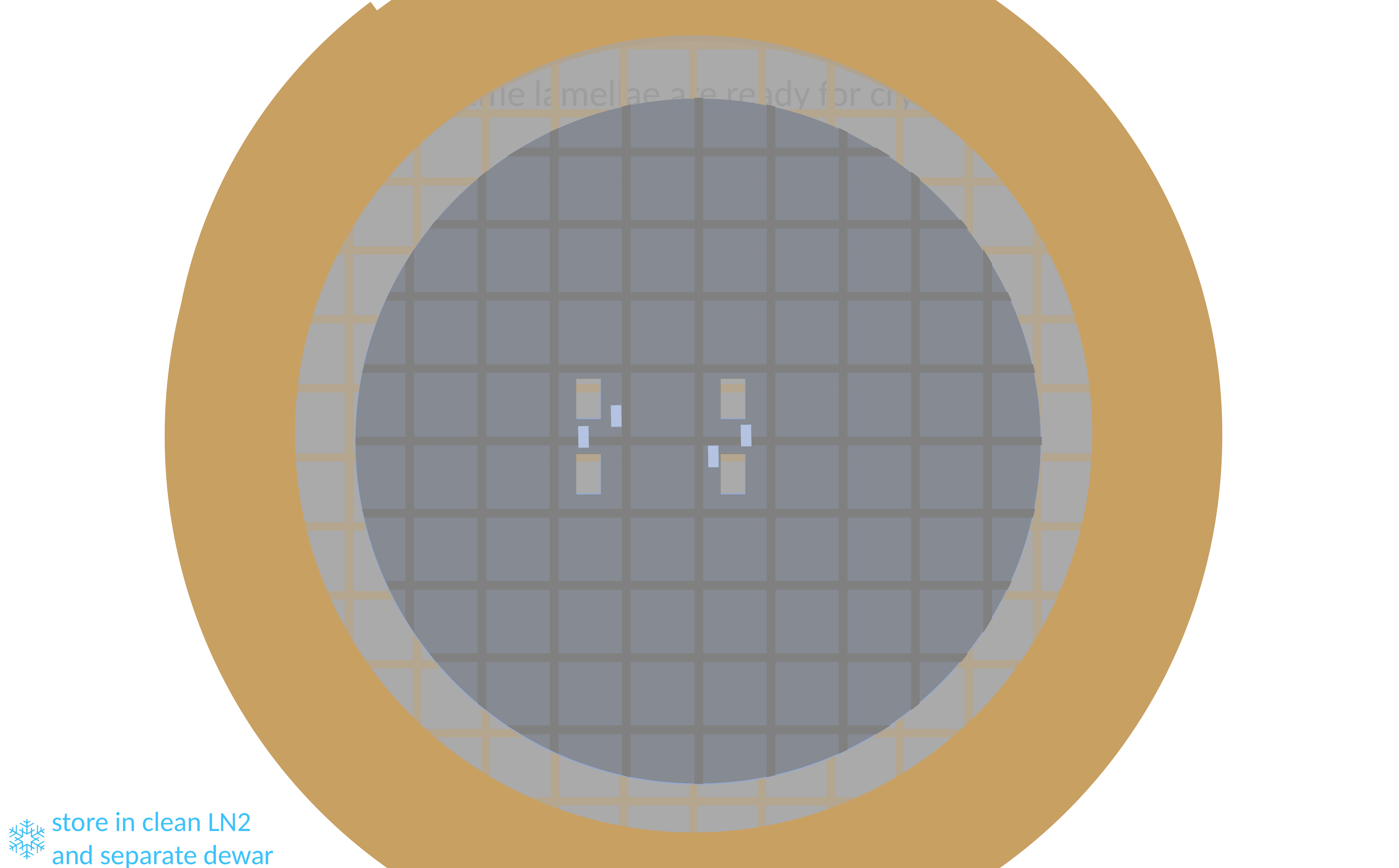

Finished!
Waffle lamellae are ready for cryoET
store in clean LN2 and separate dewar
